# Single-cell sequencing unveils key contributions of immune cell populations in cancer-associated adipose wasting

**DOI:** 10.1038/s41421-022-00466-3

**Published:** 2022-11-15

**Authors:** Jun Han, Yuchen Wang, Yan Qiu, Diya Sun, Yan Liu, Zhigang Li, Ben Zhou, Haibing Zhang, Yichuan Xiao, Guohao Wu, Qiurong Ding

**Affiliations:** 1 Department of General Surgery, Zhongshan Hospital, Fudan University, Shanghai, China; 2 CAS Key Laboratory of Nutrition, Metabolism and Food Safety, Shanghai Institute of Nutrition and Health, University of Chinese Academy of Sciences, Chinese Academy of Sciences, Shanghai, China; 3 CAS Key Laboratory of Tissue Microenvironment and Tumor, Shanghai Institute of Nutrition and Health, University of Chinese Academy of Sciences, Chinese Academy of Sciences, Shanghai, China; 4 Shanghai Jiao Tong University Affiliated Sixth People’s Hospital, Shanghai, China; 5 Institute for Stem Cell and Regeneration, Chinese Academy of Sciences, Beijing, China

**Keywords:** Mechanisms of disease, Tumour immunology

## Abstract

Adipose tissue loss seen with cancer-associated cachexia (CAC) may functionally drive cachexia development. Using single-cell transcriptomics, we unveil a large-scale comprehensive cellular census of the stromal vascular fraction of white adipose tissues from patients with or without CAC. We report depot- and disease-specific clusters and developmental trajectories of adipose progenitors and immune cells. In adipose tissues with CAC, clear pro-inflammatory transitions were discovered in adipose progenitors, macrophages and CD8^+^ T cells, with dramatically remodeled cell interactome among these cells, implicating a synergistic effect in promoting tissue inflammation. Remarkably, activated CD8^+^ T cells contributed specifically to increased *IFNG* expression in adipose tissues from cachexia patients, and displayed a significant pro-catabolic effect on adipocytes in vitro; whereas macrophage depletion resulted in significantly rescued adipose catabolism and alleviated cachexia in a CAC animal model. Taken together, these results unveil causative mechanisms underlying the chronical inflammation and adipose wasting in CAC.

## Introduction

Cancer-associated cachexia (CAC) is a syndrome with complex etiology, characterized by marked depletion of skeletal muscle and adipose tissue. Patients with CAC often show reduced food intake, elevated energy expenditure, excess catabolism and systematic inflammation^1^. CAC can compromise effects of chemotherapy and radiotherapy treatment, reduce quality of life and increase mortality^2^. According to the international Delphi consensus process in 2011^2^, CAC can occur with or without loss of fat mass. However, studies suggested that fat loss may predispose muscle loss in CAC development. For example, genetic studies using CAC animal models showed that inhibition of lipolysis in white adipose tissues (WATs) through genetic ablation of either *PNPLA2* (encoding patatin-like phospholipase-domain-containing protein 2), or *LIPE* (encoding hormone-sensitive lipase), significantly ameliorated myocyte apoptosis and proteasomal muscle degradation. These animals retained normal adipose and gastrocnemius muscle mass^3^. Another study revealed that secretion of parathyroid hormone-related protein (PTHrP) from Lewis lung carcinoma, which led to “browning” of white adipose cells, contributed to increased energy expenditure in CAC^4^. Treatment to animals developed with cachexia using an anti-PTHrP antibody inhibited adipose browning and prevented loss of skeletal muscle mass^4^. Some clinical observations also pointed out that the alteration of WAT may precede muscle wasting in some patients^5^. These findings suggested that fat loss may functionally drive muscle loss in CAC.

WAT is composed of different cellular components. Besides adipocytes and adipose stem and progenitor cells (ASPCs), there are also endothelial cells, smooth muscle cells, multiple types of resident and infiltrated immune cells in WAT. Altogether, these cells modulate the adipose microenvironment and contribute significantly to the development of metabolic disorders. In addition, the functional complexity of adipose tissues under different physiopathological conditions has also been recognized in terms of the depot-specific features of adipose tissues^6–8^. For example, distinct structural and functional differences between the subcutaneous adipose tissue (SAT) and the visceral adipose tissue (VAT) have been well characterized in obesity and relevant metabolic diseases^9–11^.

Driven by the resolving power of single-cell transcriptomics, recent studies provided new insights into the cellular complexity of adipose tissues under different physiopathologcial conditions^12–18^. The unbiased high-throughput characterization of adipose tissue fractions at single-cell level allows broader and deeper understanding of cell population structure, hierarchy and fat depot-specific nature, offering new insights into the regulatory mechanisms within adipose tissues. For example, different subsets of adipocyte progenitors with distinct functional contributions to adipogenesis have been identified^12^; adipose-resident immune cells associated with obesity have also been functionally characterized^17,19^. However, recent single-cell characterization studies of adipose tissues have mainly focused on overweight-associated metabolic syndromes, with few studies focusing on human adipose tissue^14,17,19,20^. Despite of the functional importance of WAT in the development of CAC, so far there is no study adopting the single-cell strategy to adipose samples from CAC patients.

Here we present a high-throughput single-cell expression profiling analysis of human adipose tissues derived from the visceral and subcutaneous depots of individuals with or without CAC. We characterized distinct cell types that are relevant to cachexia development and/or specific to different depots, revealing interesting depot-specific and disease-specific cellular responses to cachexia. We discovered cachexia-specific adipose progenitors in both SAT and VAT, which display clear molecular signatures of high chemokine expression; whereas macrophages showed a significant pro-inflammatory signature in adipose tissues with CAC. Remarkably, VAT displayed more immune infiltration compared to SAT. In addition, CD8^+^ T cells in VAT developed into a specific pro-inflammatory status with activated cytolitic effector pathway in cachexia patients. Further in vivo and in vitro evidences highlight a causative mechanism of adipose macrophages and CD8^+^ T cells in driving adipose catabolism during cachexia development. Taken together, our data shed light on the unique aspects of altered cellular immune responses contributing to adipose tissue wasting in CAC, which might guide the development of therapies to CAC patients.

## Results

### Identification of cell populations in the non-adipocyte fraction of adipose tissues

We generated the single-cell RNA sequencing (scRNA-seq) data from SAT and VAT adipose samples from 8 patients (4 non-cachexia vs 4 cachexia) developed with gastric cancer. Compared to the non-cachexia patients, the cachexia patients showed a clear wasting syndrome, as demonstrated by significantly decreased body weight (> 5%) and systematic inflammation (Table 1).

**Table 1:** Comparison of clinical variables between gastric cancer patients with and without cachexia.

	Non-cachexia (*n* = 4)	Cachexia (*n* = 4)	*P*
Ages (years)	59.6 ± 6.6	61.5 ± 7.4	0.74
Male (*n*)	4	4	1
BMI (kg/m^2^)	25.6 ± 1.9	21.9 ± 2.1	0.03
LY (10^9^/L)	1.64 ± 0.04	1.40 ± 0.05	< 0.01
ALB (g/L)	41.7 ± 4.5	35.6 ± 3.9	< 0.01
PA (g/L)	0.21 ± 0.01	0.18 ± 0.01	< 0.01
IL-6 (pg/mL)	4.2 ± 0.8	9.8 ± 1.3	< 0.01
TNF-α (pg/mL)	5.9 ± 1.3	11.2 ± 1.8	< 0.01
TNM stage (*n*)			0.03
I + II	4	1	
III + IV	0	3	
Proportion of weight loss in recent 6 months (%)	0.5 ± 0.1	9.5 ± 1.6	< 0.01

*BMI* body mass index, *LY* lymphocyte count, *ALB* albumin, *PA* prealbumin.

*P*-values were calculated using two-sided Student’s *t*-test. Values are means ± SD.

**Fig. 1:**
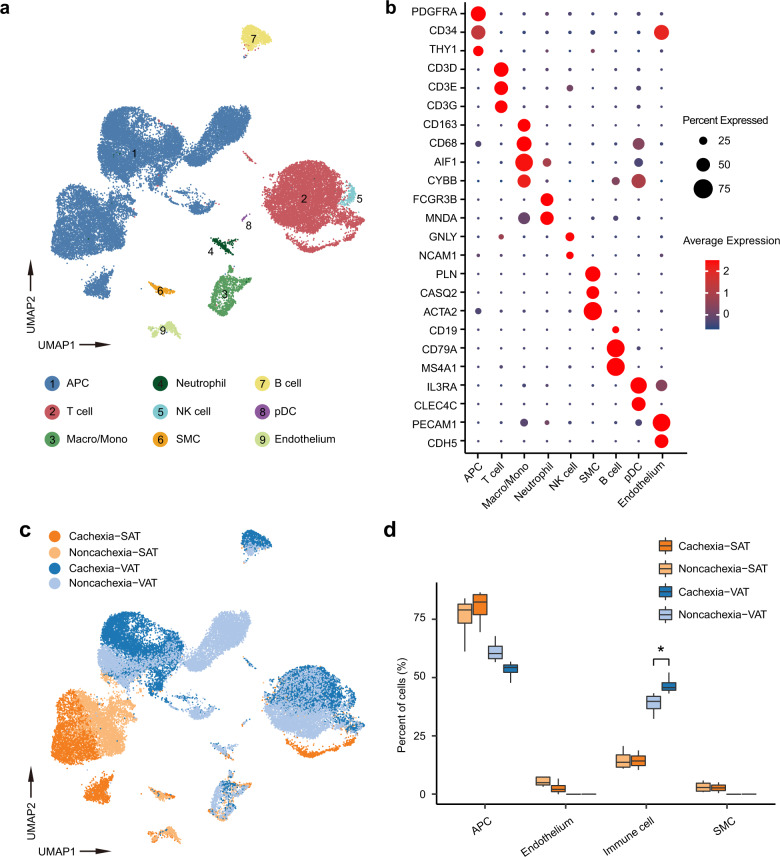
Landscape of cells in the SVF of adipose tissues from patients with or without CAC. **a** Uniform Manifold Approximation and Projection (UMAP) plot of 10X genomics-based single cells showing 9 major cell types by manual annotation. **b** Bubble heatmap showing expression levels of selected marker genes for each cell type. **c** UMAP plot showing the origins of cells. **d** Boxplot showing the proportion of major cell types from patients with (*n* = 4) or without (*n* = 4) CAC. *P*-value was determined by unpaired Student’s *t*-test. *P < 0.05. APC, adipose progenitor cell; Macro/Mono, macrophage/monocyte; NK cell, natural killer cell; SMC, smooth muscle cell; pDC, plasmacytoid dendritic cell.

scRNA-seq was then performed with the stromal vascular fractions (SVFs) from these adipose tissues. In total 42,119 cells were collected, and data from 33,856 single cells were further analyzed after quality control. The initial clustering and marker gene annotation resulted in 9 major cell populations (Fig. 1a). The progenitor cells were identified with expression of gene markers of *CD34, PDGFRA, THY1* (Fig. 1b). The immune cells were further clustered to T cells (*CD3D, CD3E, CD3G*), macrophages/monocytes (*CD163, C68, AIF1, CYBB*), B cells (*CD19, CD79A, MS4A1*), neutrophils (*FCGR3B, MNDA*), natural killer cells (*GNLY, NCAM1*), and plasmacytoid dendritic cells (pDC) (*IL3RA, CLEC4C*) (Fig. 1b). Blood vessel cells included endothelial cells (*PECAM1, CDH5*) and smooth muscle cells (*PLN, CASQ2, ACTA2*) (Fig. 1b). The distribution of different cell types between the cachexia and non-cachexia patients did not show significant difference in VAT or SAT (Fig. 1c, d). However, we observed a clear difference in the percentage of different cell types between VAT and SAT, in that there existed significantly more immune cells in VAT (32.3%–52.1% in VAT compared to 11.0%–20.6% in SAT) (Fig. 1d), suggesting more immune infiltration in VAT. SAT contained more blood vessel cells (2.0%–18.3% in SAT compared to 0–0.17% in VAT) (Fig. 1d), consistent with the observation that SAT is well-vascularized. Moreover, the fraction of immune cells in SVF was significantly higher than that in VAT of the patients with CAC (Fig. 1d).

### Distinct progenitor clusters identified in VAT and SAT

We next carried out a detailed analysis of progenitor populations in both VAT and SAT. Several recent studies applied scRNA-seq to the SVF of both mouse and human adipose tissues, providing new insights into the cellular complexity of progenitors among different mammalian fat depots^12–18^. Studies also suggested different progenitor cell types in adipose tissues between human and mouse. Although some discrepancies exist, our understanding of the heterogeneity of adipose progenitors is evolving. Summarized in a recent review^20^, the progenitor cells in SVF, which were also termed ASPCs, can be further divided into three main populations, including the adipose stem cell (ASC) population, characterized as most stemlike in nature; the preadipocyte (PreA) population, which is more committed to adipogenesis; and the adipogenesis regulator (Areg) population, which is functionally refractory to adipogenesis and can inhibit the differentiation of neighboring ASPCs. It is worthy to note that the Areg population has only been identified in mouse adipose tissues. Whether similar Areg progenitors exist in human adipose tissues is not definitive so far^20^.

**Fig. 2:**
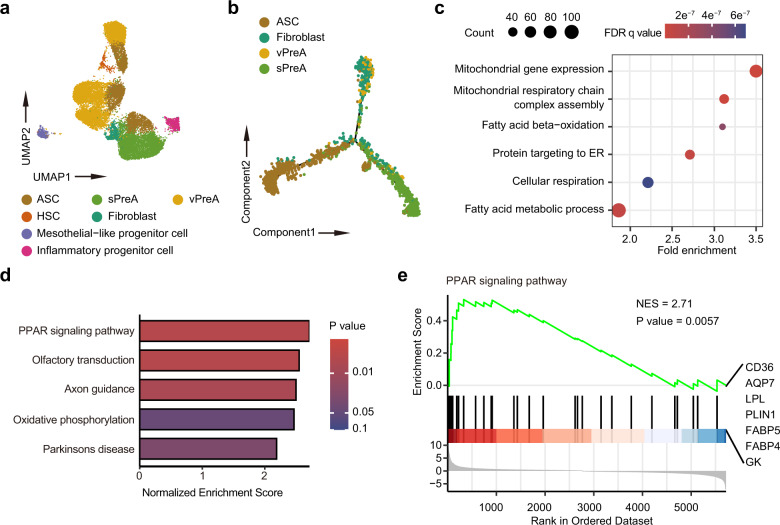
Landscape of ASPCs. **a** UMAP plot showing seven manually annotated cell populations. **b** Pseudo-time trajectory map of adipogenic progenitor cells in SAT classified by manual annotation. **c** Dot plot showing the enriched biological processes among upregulated genes in preadipocytes from SAT. **d** Bar plot illustrating top 5 enriched Kyoto Encyclopedia of Genes and Genomes (KEGG) pathways among differentially expressed genes (DEGs) between preadipocytes from SAT and visceral adipose tissue (VAT) by Gene Set Enrichment Analysis (GSEA). **e** GSEA plot showing the enrichment of genes involved in peroxisome proliferators-activated receptor (PPAR) singling pathway among DEGs between VAT and SAT. Leading edge subset of genes involving in the pathway were labeled. HSC, hematopoietic stem cell; sPreA, subcutaneous preadipocyte; vPreA, visceral preadipocyte; ASC, adipose stem cell.

The clustering of ASPCs in our study resulted in 12 groups, all of which expressed high levels of *CD34, PDGFRA*, and *THY1* (Fig. 2a and Supplementary Fig. S1a, b). Clusters 0, 1, 2, 3, 4, and 9, with higher expression of *MGP*, *CXCL14*, *APOD*, were adipogenic progenitor cells^12,14^. Moreover, we observed a depot-dependent imparity among the adipogenic progenitor cells (Supplementary Fig. S1a). Clusters 0, 2, and 3, showing relatively higher expression of *FMO2*, a maker gene for the Areg population identified in murine adipose SVF^12^, were distributed mainly in VAT and therefore labeled as visceral preadipocytes (vPreA). Clusters 1 and 4, with elevated expression of *FABP4* and *APOE*, suggesting a higher commitment to becoming mature adipocytes^12,14^, predominantly existed in SAT and therefore were annotated as subcutaneous preadipocytes (sPreA). In addition, cell cluster 9 was exclusive to SAT and showed a higher expression of genes involved in fibrosis and extracellular matrix accumulation, including *COL1A1*, *COL3A1* and *COL6A3* (Supplementary Fig. S1c), and thus was characterized as fibroblast^17^. The clusters 5, 6, and 7 were adipose stem-cell-like, as demonstrated by higher expression of *DDP4* and *CD55*^12,14^. Cluster 10 showed elevated expression of *ITLN1* and *MSLN*, indicative of its mesothelial origin^17,18,21^. Hematopoietic stem cell (HSC) markers, *PTPRC*, *CCL5* and *IL7R*, were exclusively expressed in cluster 11^17^ (Supplementary Fig. S1c). Specifically, cluster 8 exhibited a unique gene expression pattern with upregulation in several chemokines, including *CCL2*, *CXCL2*, and inflammation-related genes such as *TNFRSF12A* and *TNFAIP6*, and therefore was annotated as inflammatory progenitor cells (Supplementary Fig. S1c).

Consistent with previous studies, distinct clusters in ASPCs were found in both SAT and VAT (Supplementary Table S1a). We next performed integrated analyses to verify our manual annotations. The comparison demonstrated a consistent clustering between groups in our study and previous reports^17^. In that group P1, a subset of progenitor cells resembling adipose stem cell, was mostly overlapped with ASCs, while groups P2 and P3 mixed with sPreA, vPreA and fibroblast^12^ (Supplementary Fig. S1d). Similar results were observed when integrating our scRNA-seq data with another dataset from both human and murine subcutaneous samples^14^ (Supplementary Fig. S1d). Cell trajectory analysis of adipogenic progenitor cells in SAT identified two different transitions starting from ASC to either sPreA or vPreA and fibroblast, suggesting a bifurcated developmental trajectory underpinning an intrinsic distinction in adipogenesis between sPreA and vPreA (Fig. 2b). Along the pseudo-time, we noticed that positive regulators of adipogenesis, including members of the activating protein-1 (AP-1) family of transcription factors (*FOS*, *FOSB*, *JUN*, *JUNB*), activating transcription factor 3 (*ATF3*), and kruppel like factor (*KLF4*), were significantly upregulated in the cell fate ending with sPreA^22^ (Supplementary Fig. S2a, b). We then examined the regulatory network of preadipocytes in both SAT and VAT by using SCENIC^23^. The activity of FOS, FOSB and ATF3 regulons was remarkably higher in SAT preadipocytes (Supplementary Fig. S2c). Moreover, GO analysis indicated that compared to those in VAT, preadipocytes in SAT showed enhanced mitochondrial functions and fatty acid metabolism (Fig. 2c). Pathway enrichment analysis also suggested higher activity of PPAR signaling pathway in SAT, as demonstrated by relatively higher expression of *FABP4*, *FABP5*, *ANGPTl4*, *LPL* and *CD36*, consistent with the observation that most of the progenitor cells in SAT were preadipocytes^24^ (Fig. 2d, e). On the other hand, genes upregulated in vPreA were mostly involved in immune cell chemotaxis (Supplementary Fig. S2d).

### Cachexia-specific alterations in adipose progenitors in SAT and VAT

**Fig. 3:**
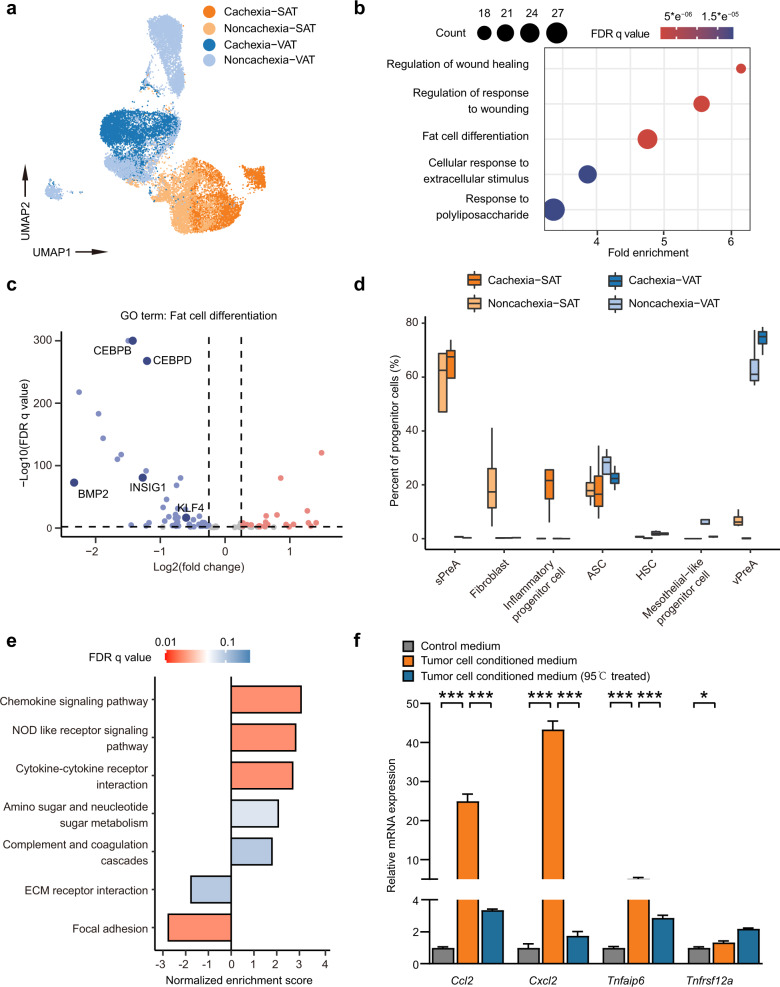
Functional characterization of cachexia-specific progenitor cell clusters. **a** UMAP plot showing the origins of all progenitor cells. **b** Dot plot showing the enriched biological processes among downregulated genes in vPreAl from patients with cachexia. **c** Volcano plot showing the differences in the expression of genes involved in fat cell differentiation in vPreA. Red dots denoting genes significantly upregulated in VAT from patients with cachexia, and blue dots denoting genes down-regulated from patients without cachexia. **d** Boxplot showing the proportion of progenitor cells derived from patients with (*n* = 4) or without (*n* = 4) CAC for each cell population. **e** Bar plot showing significantly enriched KEGG pathways in DEGs between inflammatory progenitor cell and the other progenitor cells in SAT. The color saturation of bars indicating the significance of enrichment. *P*-values were adjusted by the FDR. **f** Relative mRNA expression levels of *CCL2*, *CXCL2*, and *TNFAIP6* in white preadipocytes in each group as quantified by qPCR analyses. *P*-values were determined by the two-way ANOVA with post hoc Dunnett’s multiple-comparison tests. *P < 0.05; ***P < 0.001 (*n* = 3 biological replicates). Values are means ± SEM.

We further analyzed possible changes in progenitors in different depots between cachexia and non-cachexia patients (Fig. 3a). Interestingly, alterations in progenitor cells were observed between patients with or without cachexia. We noticed that the overall expression of genes implicated in respiratory chain reaction were much lower in sPreA when CAC developed, which might suggest a defect in mitochondrial function and lower energy supply, whereas genes involved in RNA splicing presented higher expression levels in sPreA with CAC (Supplementary Fig. S3a, b). We also observed a clear decrease in genes dominantly regulating fat cell differentiation, including *CEBPB*, *CEBPD*, *BMP2*, and *KLF4*, in vPreA in cachexia group, as compared to patients without cachexia (Fig. 3b, c). Moreover, cluster 10, annotated as mesothelium originated progenitor cells with specific expression of *ITLN1* and *MSLN*, was almost exclusively found in VAT in non-cachexia patients (Fig. 3d). Mesothelial cells were previously identified to be important in keeping the visceral cavity and adipose tissue in a normal and uninflamed state via the recruitment and differentiation of the type 2 innate lymphoid cells (ILC2s)^18,25–27^. We suspected that the loss of mesothelial cells from VAT in cachexia patients may contribute to the cachexia-associated inflammation. In addition, cluster 8 in SAT (also designated as the inflammatory progenitor cell) and cluster 0 in VAT were recognized as cachexia-specific clusters (Fig. 3a and Supplementary Fig. S1a). Interestingly, a clear molecular signature of increased inflammatory response and activated chemokine signaling pathway was noticed in these clusters (Supplementary Fig. S3c, d). For example, cluster 8 showed significantly higher expression of *CCL2*, *CXCL2*, *TNFRSF12A*, *TNFAIP6*, etc., as compared to other progenitor clusters in SAT. Further functional analysis of the upregulated genes in cluster 8 revealed significant enrichment in chemokine signaling pathway, NOD-like receptor signaling pathway and complement and coagulation cascade, suggesting an activated inflammatory response (Fig. 3e).

We hypothesized that the occurrence of cachexia-specific inflammatory progenitor cells in SAT was presumably attributed to tumor-derived factors and used marker genes identified by our scRNA-seq data including *CCL2*, *CXCL2*, *TNFAIP6*, and *TNFRSF12A* to indicate the transformation of adipogenic progenitor cells into inflammatory progenitor cells by tumor cells (Supplementary Table S2). We therefore treated immortalized adipose progenitor cells derived from the mouse SAT^28^ with conditioned medium from CT26, a mouse colon tumor cell line. Conditioned medium was filtered through a 0.22-µm filter to exclude possible contamination of tumor cells. Remarkably, we observed clear induction in the expression of marker genes of inflammatory progenitor cells after treatment with tumor cell-conditioned medium, including *CCL2*, *CXCL2*, *TNFAIP6*, and *TNFRSFL12a* (Fig. 3f). Interestingly, when tumor cell conditioned medium was boiled for 10 min at 95 °C to inactivate protein fractions, the induction of most marker genes was largely diminished (Fig. 3f). These results suggested that certain protein fractions secreted from tumor cells at least partially contributed to the activated chemokine gene expression in progenitor cells as observed specifically in cachexia patients.

### Identification of immune cell clusters in SAT and VAT

Cachexia is known to involve diverse mediators derived from cells within tissue microenvironment, especially the immune cells. The pro-inflammatory factors, either synthesized by tumor or immune cells, were considered as key mediators of cachexia^29^. We therefore performed detailed analysis of the immune cells in adipose tissues. As immune cells were known to have lower percentage of mitochondrial, we re-did the QC by discarding cells with mt % > 10%, resulting in 10,529 immune cells that were retained. Further clustering resulted in a total of 19 clusters, in which T/NK cells were further divided into 10 clusters (cluster 0, 1, 2, 3, 5, 6, 7, 8, 10, and 11). Other immune cell populations include macrophages (cluster 9), B cells (cluster 4), neutrophils (cluster 14), monocytes (cluster 13), pDCs (cluster 15), conventional dendritic cells (cDC1s, cluster 16), cCD2As (cluster 17), and cDC2Bs (cluster 12)^19^ (Supplementary Fig. S4a, b). The general components of immune cells between SAT and VAT did not show major differences (Supplementary Fig. S4c). However, VAT possessed the majority of the immune cells (86.4%, Supplementary Table S1b). In addition, VAT had a higher percentage of B cells and T cells, whereas SAT had a higher percentage of macrophages (Supplementary Fig. S4c). Compared to non-cachexia patients, cachexia patients had more adipose-infiltrating immune cells, consistent with the observed adipose inflammation^30^ (Supplementary Table S1b). Interestingly, we also noticed a unique subgroup of T cells with higher expression of cell cycle marker, such as *MKI67* and *PCNA*, indicting their vigorous capacity of proliferation (Supplementary Fig. S4b). This subset of cells is scarce yet exclusive to cachexia (Supplementary Table S1b), which might hint at the possibility of a beneficial milieus for CD8^+^ T proliferation in the context of CAC.

### Macrophages display a pro-inflammatory transition in cachexia patients and may aggravate adipose wasting

The functional contribution of myeloid cells to cachexia remains uncertain. For example, one study pointed out that macrophage depletion attenuated systemic inflammation and muscle wasting in pancreatic tumor-bearding mice^31^, whereas another study suggested that myeloid cell-mediated inflammation display a rather beneficial function in a murine HCC model in the regulation of cancer-induced fat loss^32^. Activated macrophages can be largely divided into two categories, M-like and M2-like, among which M1 macrophages are mainly involved in pro-inflammatory responses, whereas M2 macrophages are considered to be anti-inflammatory^33^. Such macrophage polarization system was extensively used to investigate the functional alterations in the context of various diseases. We initially subjected macrophages to the classification of the “classically activated” M1 and “alternatively activated” M2 polarized macrophages. However, we did not observe clear subpopulations representing classically activated macrophages (Supplementary Fig. S5a). Nevertheless, the results indicated an increased M1-preference of macrophages in VAT from cachexia patients (Supplementary Fig. S5b).

In the meantime, as suggested by previous studies^34–36^, macrophages in vivo possessed complex phenotypes and functional heterogeneity that varied well beyond denominations generalized by the M1- or M2-like in vitro polarization system. We thus next compared the gene expression profiles of each macrophage cluster to marker genes from several recent macrophage categorizations based on scRNA-seq data^19,37,38^ (Supplementary Fig. S5c, d). With this, we identified three different subtypes with unique highly expressed genes and inferred their potential pathophysiological functions (Supplementary Table S3a). Clusters 1 and 2 highly expressing *LYVE1*, *SELENOP* and *CD163* were annotated as perivascular macrophages (PVM), which have been found to reside in various tissues including both SAT and VAT^37^. Cluster 3, characterized by high expression of *MHC-II* and low expression of *LYVE1*, resembled a macrophage subtype called non-perivascular macrophages (NPVM) and was reported to occupy non-perivascular space. Cluster 4, annotated as previously reported lipid-associated macrophages (LAM) for its high expression of *TREM2*, *LGALS3* and *LIPA*, was the fewest subpopulation and mostly detected in VAT, possibly because LAM mainly relates to the obesity phenotype and functions as a homeostatic mechanism against adipocyte hypertrophy^38^.

**Fig. 4:**
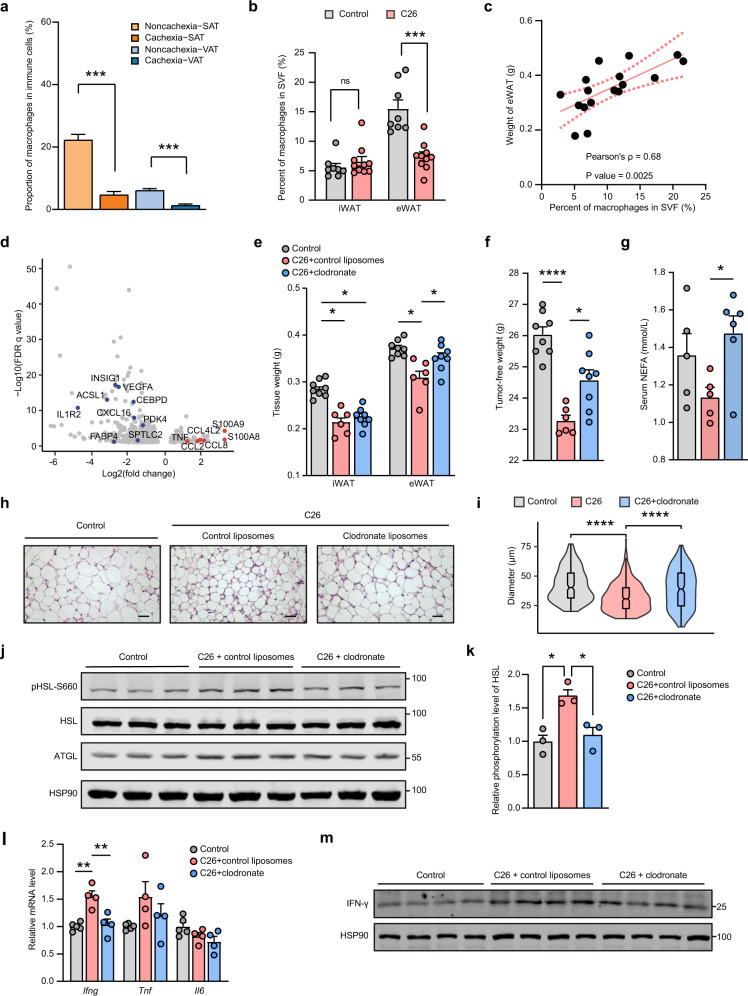
Alterations of macrophages aggravate adipose tissue catabolic activity. **a** Bar plot showing the proportion of manually annotated macrophages in all immune cells isolated from the scRNA-seq data. Bars indicate means ± SEM. *P*-values were determined by unpaired Student’s *t*-test. ***P < 0.001. **b** The proportion of macrophages in SVF of inguinal and eWAT in both control and C26 mouse model group (animals implanted with C26 colon adenocarcinoma cells) determined by flow cytometry. Bars indicate means ± SEM. ns, not significant. ***P < 0.001. **c** The scatter plot showing the positive correlation between the proportion of macrophages in eWAT and the weight of eWAT of mouse model. Linear regression and Pearson’s correlation test with 95% CI were performed and the line of best fit was shown in the plot. **d** Volcano plot showing the DEGs between macrophages from patients with or without cachexia in scRNA-seq data. Red dots represented the selected genes of interest with significant upregulation during CAC, whereas blue dots were downregulated genes. **e**, **f** Bar plots showing the tumor-free weight (**e**) and the weight of inguinal and eWAT (**f**) of mice in control group (animals with no tumor cell implanted), C26 group (animals implanted with C26 tumor cells and treated with control liposomes) and C26^+^ clodronate group (animals implanted with tumor cells and treated with clodronate-liposomes). Bars indicate means ± SEM. *P < 0.05. (*n* = 6–8/group). **g** Bar plot showing the concentration of serum non-esterified fatty acids. *P < 0.05. (*n* = 5–6/group). **h** Representative images of H&E staining for epididymal WAT sections. Scale bar: 50 μm. **i** Quantification of the area of adipocytes in three randomly selected fields from each eWAT tissue per group in H&E staining sections. The violin plot showed the density of the estimated mean diameters of each adipocyte in captured images. The inner box was bounded by the upper (75%) and lower (25%) quantiles, with the median represented by the bold horizontal line. ****P < 0.0001. **j** Western blot analysis of the expression of ATGL and the phosphorylation level of HSL in eWAT tissue (*n* = 3/group). **k** Quantification of the phosphorylation level of HSL from the results of panel **j**. *P < 0.05. Values are means ± SEM. **l** qPCR analyses showing the relative mRNA levels of *IFNG*, *TNF*, and *IL6* in eWAT tissue (*n* = 4–5/group). **P < 0.01. All *P*-values were determined by one-way ANOVA with post hoc Dunnett’s multiple-comparison correction tests. **m** Western blot analysis of IFN-γ in eWAT tissue (*n* = 4/group).

The proportion of adipose tissue macrophages (ATM) in immune cells decreased in both depots in CAC group (Fig. 4a and Supplementary Table S1b). There was also a significant decrease in quantity of both PVM and NPVM subpopulations (Supplementary Fig. S5e). Deconvolution analysis of bulk-tissue RNA sequencing data of VAT from a combination of patients with esophageal and gastric cancers also showed a reduced percentage of macrophages among tissue-infiltrating immune cells in weight loss group (Supplementary Fig. S5f). The decreasing trend in macrophage numbers in adipose tissues of CAC patients was contradictory to the previous report, suggesting that greater number of macrophages resided in VAT in a spontaneous HCC mouse model^32^. We thus further validated the results using a well-recognized experimental mouse model of CAC induced by ectopically-implanted C26 colon adenocarcinoma cells, referred to as the C26 mouse model (Supplementary Fig. S5g). We quantified CD45^+^/CD11b^+^/F4-80^+^ ATM in VAT after the development of CAC by flow cytometry. The results showed a significant decrease in the proportion of macrophages in VAT but not SAT under the condition of cachexia, consistent with the scRNA-sequencing results (Fig. 4b). Furthermore, the weight of epididymal WAT positively correlated with the abundance of macrophages in adipose tissue (Fig. 4c).

Current knowledge about the role of ATM in the context of accelerated tissue catabolism is controversial and such issue remains blurred^39^. In our study, the expression profile of macrophages in VAT from cachexia patients demonstrated a significant shift to the pro-inflammatory feature with various chemokine genes such as *CCL2*, *CCL8*, *S100A8*, *S100A9*, *TNF*^40,41^, being upregulated (Fig. 4d and Supplementary Table S3b), which was consistent with higher M1-like signature scores observed in VAT from patients developed with CAC (Supplementary Fig. S5b). On the contrary, lipid metabolism-related genes, e.g., *CEBPD* and *ACSL1*, were enriched among upregulated genes in macrophages from patients without CAC (Fig. 4d). In addition, we noticed a higher enrichment of PVM in VAT when CAC developed based on the results of *Ro/e* analysis^42^ (Supplementary Fig. S5h). Previous study indicated that PVM promoted adipose inflammation during obesity^19^. Given the clear pro-inflammatory signature of VAT macrophages under the CAC condition, we postulated that during cachexia, macrophages in VAT may also contribute significantly to the chronic inflammation in adipose tissues, which in turn promoted the lipolytic activity of adipocytes. To test this hypothesis, we treated mouse bearing CT26 colon adenocarcinoma with clodronate-liposomes to deplete the macrophages in eWAT every 2 days for 1 week before sacrificing for analyses, which resulted in around 50% depletion of macrophages in eWAT (Supplementary Fig. S5i). Interestingly, further analyses demonstrated significantly reduced weight loss, heavier adipose tissue weight and larger adipocytes in eWAT after macrophage depletion during CAC development (Fig. 4e–i and Supplementary Fig. S5j). This was in line with the previous finding that attenuated weight loss and reduced lipolysis of adipose tissues occurred in tandem with fewer macrophage infiltration after treatment of anti-IL-20 monoclonal antibodies in Lewis lung carcinoma-induced cachexia model^43^. In addition, the adipose triglyceride lipase (ATGL) expression and the phosphorylation of hormone-sensitive lipase (HSL), two key enzymes involved in lipolysis^39^, were both decreased in mouse adipose tissues treated with clodronate-liposomes, suggesting less activated lipolysis in eWAT (Fig. 4j, k). Meanwhile, the administration of clodronate-liposomes did not significantly change the tumor size and serum cytokines presumably secreted by the tumor mass (Supplementary Fig. S5k–n), which might indicate that the pro-inflammatory macrophages participate in the progression of cachexia by directly accelerating the lipolysis activity of visceral adipocytes. Among the well-known cytokines that promote tissue catabolism during cachexia, we observed an increase in both mRNA and protein expression levels of IFN-γ in epididymal WAT upon cachexia development, which was recovered to the basal level when treated with clodronate-liposomes (Fig. 4l, m). Taken together, these results indicated a pro-inflammatory transition of macrophages in VAT during cachexia development, which may significantly contribute to the adipose loss.

### The CD8^+^ T cells also present a pro-inflammatory signature and enhanced cytolytic effector activity in VAT from cachexia patients

We next turned to the analyses with lymphocytes, which comprised B cells, T cells and NK cells. Further interrogation within the B cells revealed distinct clusters identified in the VAT between cachexia and non-cachexia patients (Supplementary Fig. S6a, b). B cells from patients with cachexia showed lower expression of MHC-II molecules (*HLA-DRB5, HLA-DQA2*) and higher expression of the immunoglobulins (*IGHG2*, *IGHG3, IGKC*), and genes involved in BCR signaling (Supplementary Fig. S6c, d and Table S3c). However, the underlying biological function of these activated B cells warrants further investigation.

**Fig. 5:**
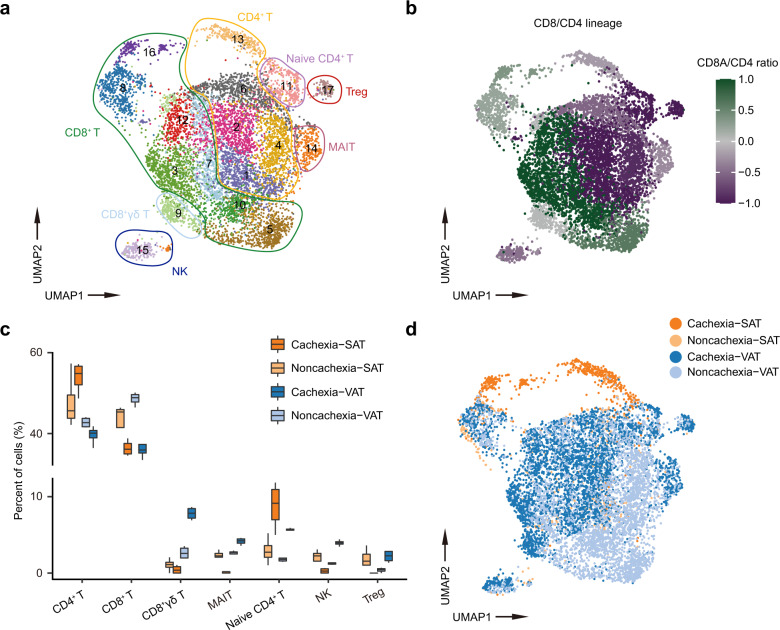
Landscape of NK/T cells in the SVF. **a** UMAP plot showing 7 major cell types identified by manual annotation. **b** UMAP embeddings of cell clusters colored by the *CD8A*/*CD4* expression ratio (cells in each cluster were assigned the same average value). **c** Boxplot showing the proportion of T/NK cells derived from patients with (*n* = 4) or without (*n* = 4) CAC for each cell population. **d** UMAP plot showing the origin of all NK/T cells.

The T/NK cells comprised the majority of immune cells in adipose tissues, we therefore did re-clustering and more detailed analyses of the T/NK cells. The clustering resulted in 17 populations, with T cells further divided into 16 subpopulations (Fig. 5a). NK cells were identified by their high expression of *GNLY*, *NCAM1* and *KLRF1* (Supplementary Fig. S6e, f). T cells contained four major cell types, including CD4^+^ T cells, CD8^+^ T cells, mucosa-associated invariant T cells (MAIT, featured by high expression of *KLRB1* and *CCR6*)^19^ and CD8^+^ γδ T cells (featured by genes encoding *TRG* and *TRD*) (Fig. 5a, b and Supplementary S6f). CD4^+^ T cells comprised three subsets, including regulatory CD4^+^ T cells (Treg) with higher expression of *IL2RA*, *CTLA4* and *FOXP3*, naive CD4^+^ T cells with higher expression of *CCR7* and *SELL*, and effector-memory CD4^+^ T cells with elevated expression of *CCL5*, a canonical marker of effector-memory T cells (TEMs)^44^ (Fig. 5a and Supplementary Fig. S6e, f). Since the majority of the captured T cells were originated from VAT (Fig. 5c, d), we next focused on the alterations of immune microenvironment in VAT during CAC.

CD8^+^ T cells have been recently implicated in the development and progression of cachexia caused by various etiologies^39,45^. After isolating the CD8^+^ T-cell subset among immune cells, further annotations based on differential gene expression showed that the subset of CD8^+^ T cells comprised seven clusters and three functional states (Supplementary Fig. S7a). Of note, all clusters of CD8^+^ T cells highly expressed *CCL5* (Supplementary Fig. S6e). Thus, all CD8^+^ T cells were divided into three categories of memory T cells (Supplementary Fig. S7a). The cluster (CD8 ITGA1) highly expressed *ITGA1* and *ZNF683*, which indicated its capacity of tissue residency^46^, and was thus annotated as tissue resident memory T cells (TRMs). Two clusters (CD8 CD16A, CD8 GNLY) highly expressed cytotoxicity-associated genes, such as *GZMB*, *PRF1*, *NKG7*, as well as activation markers including *TNFSF9*. Moreover, both clusters uniquely expressed *CX3CR1* (Supplementary Fig. S7b), indicating their preferential homing capacity for peripheral tissues, which supported that the two clusters represented the terminally differentiated effector cells (TEMRAs)^47^. The remaining four clusters represented TEMs, two of which (CD8 IFNG, CD8 GZMH) were considered as activated TEMs for high expression of activation and effector markers *TNFSF9*, *CD74*, *IFNG* and *MHC-II*^48^ (Supplementary Fig. S7b).

**Fig. 6:**
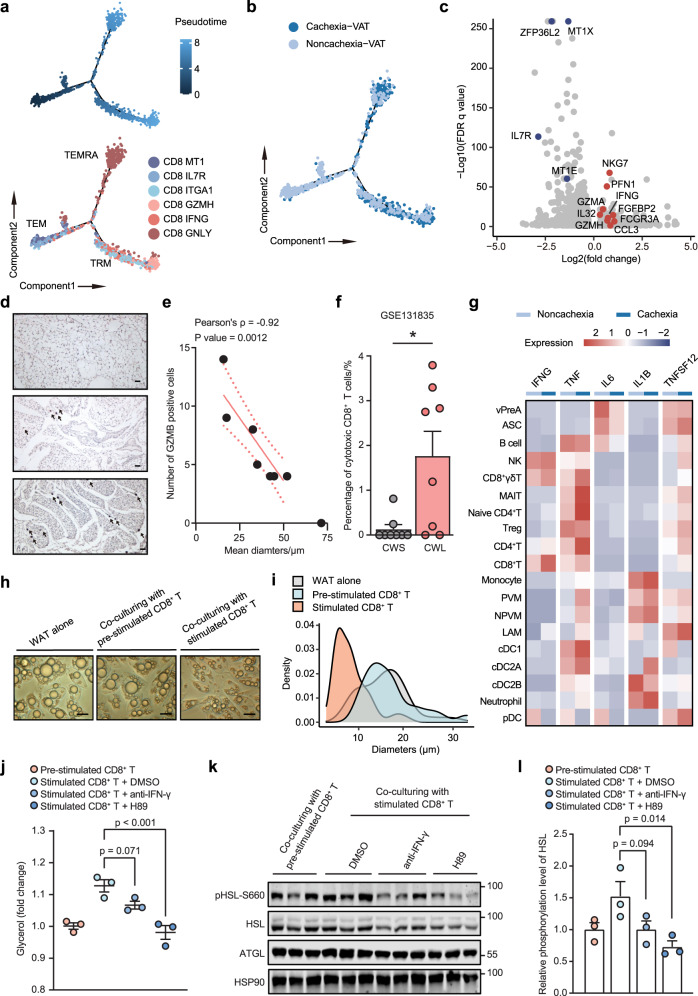
Functional analysis of CD8^+^ T cells from VAT between cachexia and non-cachexia patients. **a** Pseudo-time ordered CD8^+^ T cells from VAT overlying the inferred cell trajectories by using Monocle. Pseudo-time was depicted from dark to light blue (upper). Pseudo-time ordered cells overlying the cell trajectories were labeled by indicated CD8^+^ T-cell clusters (lower). **b** Pseudo-time ordered cells classified by the cell origin. **c** Volcano plot showing the DEGs of CD8^+^ T cells in VAT from patients with or without cachexia. Red dots represented the selected genes of interest with significant upregulation during CAC, whereas blue dots were downregulated genes. **d** Representative images of the immunohistochemistry analyses for GZMB in greater omentum (VAT) sections for another 8 patients with gastric cancer. Positive staining was pointed with arrowhead lines. Scale bars: 50 μm. **e** Scatter plot showing the negative correlation between the mean diameters of adipocytes in each section and the number of GZMB-positive cells. Linear regression and Pearson’s correlation test with 95% CI were performed and the line of the best fit was shown in the plot. **f** Bar plot showing the inferred percentage of cytotoxic CD8^+^ T cells in all immune cells for both cancer with weight stable (CWS) and cancer with weight loss (CWL) group. *P*-value was determined by Student’s *t*-test. *P < 0.05. **g** Heatmap plot depicting the row scaled expression values of indicated pro-catabolic cytokines within each cell type from VAT, separated by the disease condition. **h** Representative images of adipocytes co-cultured with murine primary splenic CD8^+^ T cells activated in vitro for 24 h. Scale bars: 25 μm. **i** Density plot showing the distribution of diameters of droplets within adipocytes in each group. The diameters were measured from three randomly selected field for each well, at 40× magnification (*n* = 3/group). **j** Supernatant glycerol levels of adipocytes under each co-culturing condition, normalized by the glycerol concentrations in medium under co-culturing with pre-stimulated CD8^+^ T cells. Bars indicate means ± SEM. *P*-values were determined by one-way ANOVA and *post hoc* Dunnett’s multiple-comparison correction tests. **k** Western blot analysis of the phosphorylation of HSL and the expression of ATGL in each co-culturing condition (*n* = 3/group). **l** Quantification of results from the penal k. Bars indicate means ± SEM. *P*-values were determined by one-way ANOVA with post hoc Dunnett’s multiple-comparison correction tests.

We next explored the dynamic immune states and cell transitions focusing on CD8^+^ T cells by inferring the state trajectories using Monocle (Fig. 6a). Pseudo-time analysis revealed that CD8^+^ T cells infiltrating VAT possessed different transition trajectories between patients with or without CAC. The transition started from CD8 IL7R and CD8 MT1 cells, which showed higher scores of resting signatures^49^ (Supplementary Fig. S7c). TEMRA with much higher scores of cytotoxicity presented at one end of the trajectory branch, while at the other branch, TRM appeared as an intermediate state, terminating as activated TEM (Fig. 6a and Supplementary Fig. S7c). Besides, along the pseudo-time, CD8^+^ T cells in CAC group emerged at a later stage (Fig. 6b), indicting their activated nature. Indeed, differential gene expression analysis of CD8^+^ T cells showed significantly higher expression of genes representing activated state in cachexia patients, including *GZMH*, *GZMA*, *NKG7*, and *IFNG* (Fig. 6c and Supplementary Table S3d). The GO analysis of differential gene expression in CD8^+^ T cells revealed a significant upregulation of genes involved in TCR-mediated activation in patients with CAC (Supplementary Fig. S7d). In addition, consistent with higher percentage of effector-memory CD8^+^ T cells in non-cachexia patients, genes relevant to the dysfunctional phenotype and the state of inactivation such as *MT1X*, *MT1E, IL7R*, and *ZFP36L2* were highly expressed in CD8^+^ T cells in non-cachexia patients (Fig. 6c). The analyses of metabolism-related pathways showed an elevated activity in glycolysis at the pseudo-time where TEMRA resided (Supplementary Fig. S7e), corresponding to the metabolic features of memory CD8^+^ T cells at early time points after receiving activation signals^50^. Moreover, along the pseudo-time, the activity of tricarboxylic acid (TCA) cycle gradually increased, suggesting the commencement of anaplerosis, which mainly functioned to fuel either biosynthetic pathways using TCA cycle intermediates as substrates or oxidative phosphorylation to fulfill increasing energy requirement during the activation of memory T cells^51^ (Supplementary Fig. S7e). To further confirm the finding that more activated CD8^+^ T cells with high cytotoxicity infiltrated into adipose tissue during CAC development, we collected adipose tissues from another 8 patients with gastric cancer undergoing surgical therapy and stained the GZMB^+^ cells by immunohistochemistry (Fig. 6d). The results showed a strong correlation between the number of stained GZMB^+^ cells and the size of adipocytes (Pearson’s correction: −0.9203, *P*-value: 0.001, Fig. 6e), indicting the relation between local tissue catabolism in adipose tissues triggered by cancer related factors and the infiltration of cytotoxic T cells. In addition, the inferred percentage of cytotoxic CD8^+^ T cells was increased in VAT of patients with weight loss by deconvolution analysis of bulk RNA-seq data from patients with esophageal or gastric cancer (Fig. 6f). Several pro-inflammatory cytokines such as TNF, IL1, IL6, and IFN-γ are previously identified to be upregulated in cachexia, and be able to stimulate the catabolic processes in experimental CAC models^29^. We observed a general increase in *TNF* and *TNFSF12* expression in multiple cell types in SVF of adipose tissues from cachexia patients, whereas no significant increase was seen in *IL6* or *IL1B* expression in these SVF cells, indicating a possible major resource from mature adipocytes, which were not included in the scRNA-seq analysis (Fig. 6g). Interestingly, we noticed a specific increase of *IFNG* expression in CD8^+^ T cells and CD8^+^ γδ T cells in adipose tissues from cachexia patients (Fig. 6g). This phenomenon, together with the observation that CD8^+^ T cells exist with different activation states between patients, led us to ask whether there were any contributions of activated CD8^+^ T cells to the adipose catabolism. To directly evaluate the effect of CD8^+^ T cells on the lipid catabolism, we co-cultured mature adipocytes with activated primary CD8^+^ T cell in vitro. Indeed, after 1 day co-culturing, we observed a significant decrease in the size of lipid droplets in adipocytes and an increase in glycerol concentration in the culture supernatant, indicating enhanced adipose lipolysis (Fig. 6h–j). Consistently, increased phosphorylation level of HSL in adipocytes was also observed after co-culturing with activated primary CD8^+^ T cells (Fig. 6k, l). qPCR analyses for genes involved in lipogenesis, lipolysis and cell death suggested that the enhanced catabolism under the co-culturing condition was unlikely to be regulated at the transcription level (Supplementary Fig. S7f). To further confirm the involvement of activated lipolysis in this phenomenon, we then used the PKA inhibitor (H89) and ATGL inhibitor (atglistatin) to block the lipolytic activity in the manner of lipase phosphorylation. The results demonstrated a significant attenuation in the co-culturing-induced glycerol increase as well as upregulation of HSL phosphorylation after H89 treatment (Fig. 6j–l and Supplementary Fig. S7g, h). We further tested whether the neutralization of IFN-γ in vitro could reverse the accelerated process of lipolysis caused by the activated CD8^+^ T cells. The addition of anti-IFN-γ antibody at 10 μg/mL could at least partially lower the release of glycerol and attenuate the activation of HSL though the changes did not reach statistical significance (Fig. 6j–l). These results altogether demonstrated a pro-catabolic function of activated CD8^+^ T cells to adipocytes in vitro, suggesting a possible function of activated CD8^+^ T cells in producing more IFN-γ and stimulating the adipose catabolic process during cachexia development.

### Cell interaction analysis predicts a dramatic remodeling of the human WAT interactome in cachexia

**Fig. 7:**
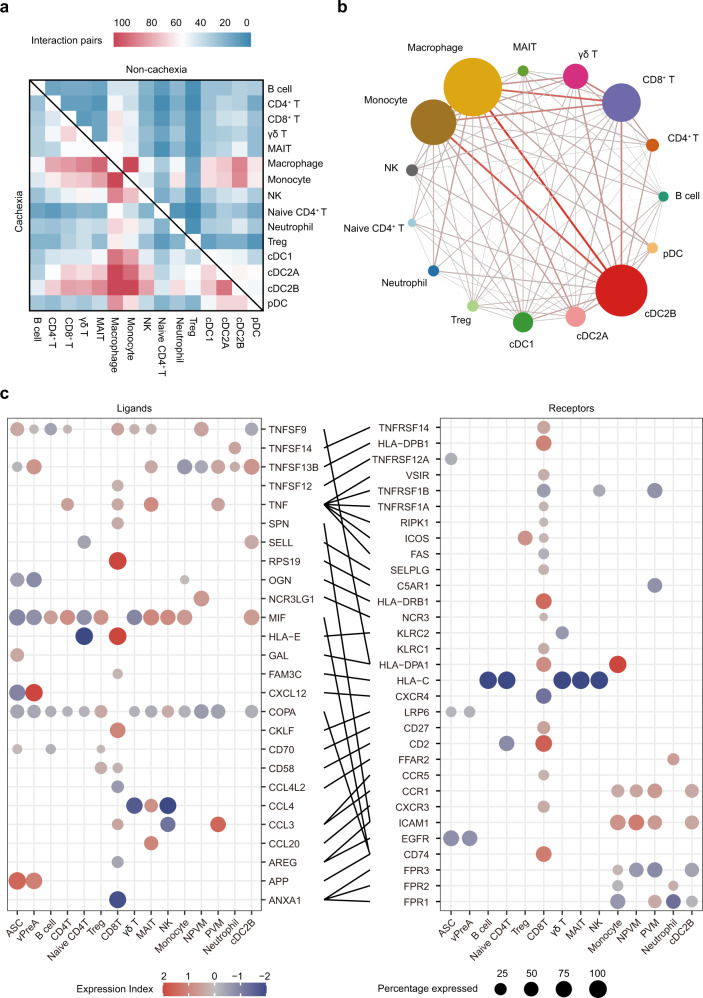
Cell ligand-receptor inference analysis of immune cells in VAT. **a** Interaction heatmap plotting the number of the ligand-receptor pairs between each immune cell type in VAT. Crosstalk of immune cells in VAT from patients without CAC is shown in the upper area, while that from patients with CAC is shown in the lower area. **b** Connectome web analysis of interacting immune cells based on the expression of paired ligands and receptors. The size of nodes denotes the number of pairs involved in each cell type, and the color saturation of the line is proportional to the amount of the pairs between two nodes. **c** Dot plots showing the expression of ligands (left) and receptors (right) implicated in the interaction pairs of CD8^+^ T cells in each immune cell type. Color saturation reflects the log_2_ fold-change of the indicated gene expressed in cells from patients with cachexia compared with patients without cachexia.

Given the conspicuous changes in the constitution and functional phenotype of certain immune cell types within VAT, we next explored to what extent such transition in activation states influenced the cell communications. By inferring the paired ligand-receptor pairs based on CellPhoneDB analysis^52^, we first depicted the overall connectivity patterns across immune cells in VAT. The number of ligand-receptor pairings between immune cells was significantly larger in the context of CAC (Fig. 7a), especially those involving macrophages. Further interrogation into upregulated ligands expressed in macrophages revealed that the macrophages might exacerbate adipose tissue inflammation in recruitment and activation of several lymphoid and myeloid cells, including CD4^+^ T cells, CD8^+^ T cells, monocytes and neutrophils, via expression of *ICAM1*, *CCL3* and *CCL3L1* (Supplementary Fig. S8a). Consistent with previous studies on the immune microenvironment in the context of obesity^19^, we found that PVM appeared to be a major inflammation mediator by producing various chemokines during CAC in a similar manner. We then applied Connectome^53^ web analysis to unveil the potential target cells of macrophages during CAC (Fig. 7b). The web analysis revealed that macrophages shared similar connectivity with monocytes and cDC2Bs, which reflected the close relatedness of these myeloid cells with regard to phenotypes and functions^19,54^. Intriguingly, CD8^+^ T cells exhibited more extensive communications with macrophages than other immune cell types apart from monocytes and cDC2Bs. We next extracted highly expressed interactions engaging CD8^+^ T cells during CAC and uncovered underlying interactions with macrophages (Fig. 7c). For example, PVM may play an important role in mediating chronic inflammation via upregulating cytokine *CCL3* and interacting with its receptor *CCR5* expressed on CD8^+^ T cells (Fig. 7c and Supplementary Fig. S8a). In summary, the interaction analyses between immune cells suggested a strong interaction between macrophages and CD8^+^ T cells during cachexia development. In addition, the recovered *IFNG* expression, which was presumably from activated CD8^+^ T cells (Fig. 6g), after macrophage depletion in the mouse cachexia model, also indicated a functional connection between macrophages and CD8^+^ cells in driving adipose catabolism. Besides, the Connectome web analysis also unveiled extensive interactions between the cachexia-specific inflammatory progenitor cells and immune cells (Supplementary Fig. S8b), highlighting a drastic remodeling of cell interactome, involving adipose progenitors, macrophages, CD8^+^ T cells, among others, in adipose tissues from cachexia patients.

## Discussion

In this study, we have performed a large and unbiased analysis of the cellular landscape in the non-adipocyte fraction of human adipose tissues from patients developed with or without CAC. We noticed significant cell type- and depot-specific responses to cachexia, especially highlighting a complex immunological involvement during cachexia development. In summary, we noticed three main transitions of cellular states in response to cachexia (Supplementary Fig. S9): first, we noticed a dynamic change of the ASPCs in cachexia patients, in which cachexia-specific progenitor population was discovered in both SAT and VAT, displaying clearly higher expression of chemokine genes, including *CCL2* and *CXCL2*; second, macrophages in VAT from cachexia patients harbored a significant pro-inflammatory feature with various chemokine genes being upregulated, such as *CCL2*, *CCL3L1*, *CCL3*, *CCL8*, and *CCL12*; third, CD8^+^ T cells in VAT from cachexia patients displayed a clear activated status with significantly higher expression of cytolytic genes and *IFNG*. These transitions discovered in adipose tissues with CAC have consistently unveiled a remarkable change of inflammatory program during CAC development, putting the immunological aspects to the upfront of the cachexia syndrome.

Further cell interaction analyses revealed significantly enhanced interaction between immune cells as well as extensive interaction of cachexia-specific inflammatory adipose progenitors and immune cells in the context of CAC (Supplementary Fig. S9). Specifically, the interaction analysis has placed macrophages with a key role in recruitment and activation of several lymphoid and myeloid cells, including CD8^+^ T cells, via expression of *ICAM1*, *CCL3*, and *CCL3L1*, etc. This is different from a previous study in obese-relevant adipose tissue inflammation, in which CD8^+^ effector T cells were suggested to contribute to macrophage recruitment^55^. In contrast to adipose tissue inflammation in obesity, in which the macrophage number in adipose tissues is significantly increased, the number of macrophages is actually decreased in adipose tissues with cachexia. However, our scRNA-seq data is somewhat incomparable with respect to the change in cell composition across diseases like obesity and aging, given the relatively low proportion of myeloid cells, which is a major limitation of our study. The sequence of events regarding activation of macrophages and CD8^+^ T cells during cachexia development warrants further detailed investigation. In addition, the functional involvement of the identified cachexia-specific adipose progenitor populations in CAC development is also intriguing. Especially this progenitor population annotated as the “inflammatory progenitor cell”, which exclusively existed in SAT from cachexia patients, and has not been discovered so far in other scRNA-seq studies with adipose tissues. The interactome analyses also suggest highly active communication of the cachexia-specific inflammatory progenitor population with macrophages, T cells, among others, which may also contribute to the immune activation of macrophages and CD8^+^ cells as observed in cachexia patients. Interestingly, our initial in vitro experimental evidences suggest that these chemokine genes highly expressed in the cachexia-specific progenitor population may be at least partly induced by some protein fractions secreted by tumor cells, suggesting a possible tumor cell—inflammatory adipose progenitor—pro-inflammatory macrophage and CD8^+^ T cascade reaction under cachexia development.

Furthermore, our studies suggest a causative mechanism of macrophage and CD8^+^ T-cell-derived pro-inflammatory cytokines to activate adipose catabolism in cachexia (Supplementary Fig. S9). Macrophage depletion in eWAT in a C26 colon adenocarcinoma-induced mouse CAC model resulted in significantly reduced weight loss and alleviated catabolism in adipose tissues; and the in vitro experiment also demonstrated a clear pro-catabolic function of activated CD8^+^ T to mature adipocytes. In addition, among all cell types identified in the SVF, the activated CD8^+^ T from cachexia patients presents as a major resource of increased IFN-γ in adipose tissues, which is previously identified to be able to stimulate the tissue catabolism process. Whether the activated macrophage or CD8^+^ T cells can be targeted to attenuate the systematic inflammation and cachexia development, especially with minimal disturbance to cancer treatment, needs careful experimental evaluation.

Taken together, we presented in this study the cellular landscape of human adipose tissues, and revealed significant transitions in cellular distribution and functional molecular signatures of both adipose progenitor cells and immune cells in cachexia patients. Our characterization of these changes and the relevant experimental evidences provide important resources that may guide new discoveries of the functional contribution of the adipose tissues in the CAC development, and hopefully new potential treatments.

## Materials and methods

### Human subject

Adipose tissues from four gastric cancer patients with cachexia (weight loss > 5% in recent 6 months) and an equal number of gastric cancer patients without cachexia (weight loss < 5% in recent 6 months) were included in the single-cell sequencing experiments, with patients’ clinical characteristics collected and listed in Table 1. Adipose tissues from another eight gastric cancer patients were used for immunohistochemistry analyses. This study was performed in accordance with the Declaration of Helsinki and approved by the Ethics Committee of Zhongshan Hospital, Fudan University (No. B2019–193R). Written informed consent was obtained from all patients. For each patient, SAT and VAT were collected from midline abdominal incision and greater omentum during surgery, respectively.

### Animals

All animals were maintained and used in accordance with the guidelines of, and under approval by, the Institutional Animal Care and Use Committee of the Shanghai Institute for Nutrition and Health (ethical committee approval No. SINH-2021-DQR-1). All animals presented a healthy status and male mice were used for all experiments. 8–10 weeks old C57BL/6J wild-type mice were utilized to harvest the primary splenic lymphocytes for further activation in vitro. To induce CAC, 7-week-old male BALB/c mice were purchased from SLACCAS and housed under specific pathogen-free conditions with ambient temperature of 22 °C, under 12 h light/dark cycles, and with free access to regular rodent diet and drinking water. After 1 week of adaptation, the CT-26 murine colonic adenocarcinoma cells (1 × 10^6^ per mouse in 100 μL PBS) were subcutaneously inoculated in the dorsal area of animals. Twenty-four days after tumor cell inoculation, mice were sacrificed for analysis. The body weight, and the weight of tumor mass, inguinal adipose tissue, and epididymal adipose tissue were recorded.

### Cell culture

CT26 cells (Cell Bank, Type Culture Collection Committee, Chinese Academy of Sciences, Shanghai) were maintained in Dulbecco’s Modified Eagle’s Medium (DMEM, Gibco, 8117254) containing 10% fetal bovine serum (FBS, Gibco, 16000044), and 1% penicillin-streptomycin. The white preadipocytes were generated as previously described^28,56^, and were maintained in DMEM with 20% FBS and 1% Penicillin-Streptomycin solution. Cells were grown in a humidified incubator with 5% CO_2_ at 37 °C. Purified primary CD8^+^ T cells were cultured in round-bottom 96-well plate pre-coated with anti-CD3 (1 μg/mL) and anti-CD28 (2 μg/mL) functional antibodies overnight at 4 °C, with complete RPMI 1640 medium (Corning Cellgro, No. 10-040-CVRC) with 10% FBS, 1% Penicillin-Streptomycin solution and 25 ng/mL recombinant mouse IL-2 (SinoBiological) for 2 days.

### Fluorescence-activated cell sorting

For isolating primary CD8^+^ T cells, 8-week-old male C57BL/6J wild-type mice were sacrificed and the spleen was removed aseptically. To obtain the single-cell suspension, the spleen was placed in between two pieces of sterile 100 μm cell strainer mesh and mashed in a petri dish containing 2 mL ice-old RPMI 1640 with 3% FBS. After red blood cell lysis, cells were filtered through a 70-μm cell strainer. For surface staining, the isolated cells were then incubated with antibodies against surface antigens in blocking buffer on ice for 30 min. The following fluorophore-conjugated antibodies were used: anti-CD3ε-APC (clone 145-2C11, MultiSciences, No. 70-AM003E05), anti-CD8α-FITC (clone 53-6.7, MultiSciences, No. 70-AM008A01), anti-CD45-PE-Cy7 (clone I3/2.3, BioLegend, No. 147704). DAPI (Beyotime) was used to exclude dead cells. Flow cytometry was performed on MoFlo Astrios EQ Cell Sorter (Beckman Coulter), with the primary splenic lymphocytes sorted as CD45^+^CD3ε^+^CD8α^+^.

### Flow cytometry

For isolation of adipose stromal vascular fraction, inguinal or epididymal adipose tissues were harvested and digested in 1 mL PBS containing 1 mg Collagenase Type II (Sigma-Aldrich, No. C6885), 2.4 mg Dispase II (Sigma-Aldrich, No. D4693) and 10 mM CaCl_2_ at 37 °C for 45 min, under constant agitation. The digested tissue was washed by high-glucose DMEM medium with 10% FBS and then filtered through a 70 μm cell strainer. Cells were then analyzed for cell-surface markers and stained with following fluorophore-conjugated antibodies: anti-CD45-PE/Cy7 (clone I3/2.3, BioLegend, No. 147704), anti-CD11b-APC (clone M1/70, BioLegend, No. 101212) and anti-F4/80-PE (clone BM8, BioLegend, No. 123110). Flow cytometry was performed on CytoFLEX LX Flow cytometer (Beckman Coulter) and macrophages (CD45^+^CD11b^+^F4/80^+^) were analyzed with FlowJo software (Tree Star).

### Macrophage depletion

For the experiment of in vivo macrophage depletion, C26 mice were randomly divided into two groups 14 days after tumor inoculation and injected intraperitoneally with 100 μL clodronate-liposomes (FormuMax, No. F70101C) or plain control liposomes (FormuMax, No. F70101-N) per mouse, respectively, every 2 days for 1 week. Mice in both C26 and control groups were euthanized on day 24 after tumor inoculation.

### Histology and immunohistochemistry

Both surgically dissected adipose tissues from mouse models and gastric patients undergoing curative surgeries were immediately fixed in formalin for 48 h and embedded within paraffin. For Hematoxylin and Eosin (H&E) staining, paraffin blocks were cut into 3 μm thick slides and stained with H&E according to the standard protocols. For immunohistochemistry, paraffin blocks were cut into 10 μm slides and underwent deparaffinization in xylene and rehydration in 100%, 90%, 70% alcohol successively. Antigen retrieval was performed by using Tris-EDTA buffer (pH 9.0) in 95 °C water bath for 25 min. Following the pre-incubation with 3% bovine serum albumin to block nonspecific sites at 37 °C for 30 min, the sections were incubated with primary antibody against GZMB (1:1000, Abcam, No. ab255598) in a humidified chamber at 4 °C overnight. After then, 3% H_2_O_2_ was used to inactivate endogenous peroxidase by incubation at room temperature for 30 min. Sections were then incubated with secondary anti-rabbit antibodies conjugated with HRP and antigens were subsequently labeled by DAB staining. Hematoxylin was used as the counterstain to enhance the tissue morphology. For HE staining, three representative images at 20× magnification of each fat pat were acquired for quantitative analysis by using ImageJ-Adiposoft plugin. Cutoff value for automated selection of adipocytes was set as 20– 300 μm in mean diameters.

### Preparation of tumor cell-conditioned medium

To collect conditioned medium, CT26 cells were plated at a 1:3 or 1:4 confluence. Two days after cells grew confluent, the medium was collected and subsequently filtered using a 0.22 μm vacuum filter. The conditioned medium was then incubated at 95 °C for 10 min or stored at 4 °C for immediate use. White preadipocytes were treated with conditioned medium for 10 days starting at day 1 after cell passaging. When white preadipocytes were treated with conditioned medium, the treatment medium was composed of 33% fresh adipocyte culture medium and 67% CT26-cell-conditioned medium, with control group incubated in adipocyte culture medium. The treatment medium was changed daily.

### Co-culture of primary CD8^+^ T cells with mature adipocytes

For culturing of mature adipocytes, we applied our previously established in vitro preadipocytes differentiation model^28^. Immortalized murine inguinal WAT preadipocytes reaching 70%–80% confluence were treated with differentiation induction medium consisting of high-glucose DMEM medium with 10% FBS, 1% Penicillin–Streptomycin solution, 5 μg/mL insulin, 1 μM dexamethosone, 0.5 mM IBMX and 1 μM rosiglitazone. After 2-days of induction, adipocytes were then cultured in maintenance medium comprising high-glucose DMEM medium with 10% FBS, 1% Penicillin-Streptomycin solution and 5 μg/mL insulin. Culture media were changed every 2 days for another 8 days until when the primary CD8^+^ T cells were co-cultured with mature adipocytes. For co-culture stimulation, adipocytes were co-cultured with primary CD8^+^ T cells in 12-well plates for 24 h in maintenance media at a ratio of 10% CD8^+^ T cells to adipocytes and adipocytes cultured alone were set as negative control. Mature adipocytes were co-cultured under the following conditions: (1) with unstimulated CD8^+^ T cells; (2) with TCR-stimulated CD8^+^ T cells; (3) with TCR-stimulated CD8^+^ T cells plus 5 μM ATGL inhibitor Atglistatin (Selleck, No. S7364); (4) with TCR-stimulated CD8^+^ T cells plus 5 μM PKA inhibitor H89 (TargetMol, No. T6250); (5) with TCR-stimulated CD8^+^ T cells plus anti-IFN-γ antibody (Clone XMG1.2, Proteintech, No. KE10001) at 10 μg/mL. Culturing media were collected and the supernatant after centrifuging 10 min at 12,000 rpm were used to test the concentration of glycerol reflecting the magnitude of lipolysis resulting from the co-culture. The lipolysis assay was performed by using the glycerol assay kit (Nanjing Jiancheng, No. F005-1-1) according to the manufacturer’s protocol.

### Quantification of adipocyte lipid content

Lipid content within cultured mature adipocytes was assessed by staining with 2 μM BODYPI 493/503 (GPLBIO, No. GC42959) in PBS solution at 37 °C for 15 min, followed by fixation via 4% paraformaldehyde at room temperature for 30 min. Cell nuclei were labeled by staining with DAPI at room temperature for 15 min. Cells were imaged on Echo Laboratories RVL-100-G Microscope (ECHO). Nucleus number and lipid content were captured by using DAPI and green fluorescent protein filters, respectively. Quantification was performed by ImageJ software.

### Western blot analysis

Proteins from cultured cells and adipose tissue were extracted by RIPA buffer (Millipore) and subjected to a regular western blot procedure. The primary antibodies used in the experiments were antibodies to HSP90 (Cell Signaling, No. 4874S), HSL (Abclonal, No. A15686), pHSL-S660 (Abclonal, No. AP1242) and ATGL (Abclonal, No. A5126). Quantification of band density was performed by Image Studio Lite Software (LI-COR Biosciences).

### ELISA assays

The concentration of cytokines (IL-6, TNF-α, and IFN-γ) in the mouse serum was measured by using commercial ELISA sandwich kits according to the manufacturer’s protocol (Proteintech).

### RNA isolation and quantitative RT-PCR

Total RNA was isolated from white preadipocytes using Trizol reagent (ThermoFisher, No. 15596018) according to the manufacturer’s instructions. Reverse transcription of isolated RNA was performed using the reverse transcription kit (Takara, RR047A). Quantitative real-time PCR was carried out on the 7900 System using SYBR Green supermix (ABI, No. 4472908). Primers used in this study were listed in the Supplementary Table S4.

### Single-cell isolation and sequencing

The single-cell isolation and sequencing were performed in Shanghai OE Biotech. Co. Ltd. After surgery, human adipose tissues were immediately placed into the tissue storage solution (Miltenyi Biotec, Gladbach, Germany), which were then dissociated into single-cell suspensions by mechanical dissociation combined with enzymatic degradation of the extracellular matrix (Miltenyi Biotec, Gladbach, Germany), to ensure the structural integrity of tissues. Briefly, the tissues were first washed with Roswell Park Memorial Institute 1640, and further cut into small pieces. The pieces were then incubated with enzymes for 3 h at 37 °C for enzymatic digestion, which were then passed through a filter with a pore size of 40 μm to remove any remaining large particles. The suspension was later centrifuged at 300× *g* for 5 min at 4 °C, and cells were collected with supernatant completely discarded. Red Blood Cell Lysis Solution (10×) (Sigma-Aldrich, St. Louis, MO, USA) was further used to remove erythrocytes. To remove dead cells, a Dead Cell Removal Kit (Miltenyi Biotec) was used to ensure a cell viability > 90%.

For single-cell sequencing, libraries were prepared using a Chromium Single-cell 3′ Reagent kit v2 (10X Genomics, Pleasanton, CA, USA), according to the manufacturer’s protocol. Briefly, single-cell suspensions were loaded on the Chromium Single-Cell Controller Instrument (10X Genomics, Pleasanton, CA, USA) to generate single-cell gel beads in emulsions (GEMs). After generation of GEMs, reverse transcription reactions were performed to produce barcode full-length cDNA, followed by the disruption of emulsions using the recovery agent. Barcoded cDNA was later cleaned up with DynaBeads Myone Silane Beads (Thermo Fisher Scientific, Waltham, MA, USA), and subsequently amplified by PCR with appropriate cycles, depending on the recovery of cells. The amplified cDNA was then fragmented, end-repaired, A-tailed, index adapter ligated, and used for library amplification. Library sequencing was performed on the Illumina sequencing platform (HiSeq X Ten; Illumina, San Diego, CA, USA) and 150 bp paired-end reads were generated.

### Single-cell data processing

Single-cell sequencing data were initially integrated and processed by Cell Ranger single-cell software suite v3.1.0 pipeline from 10X genomics. SAT and VAT from every four patients with or without CAC were mixed, respectively, resulting in four samples. Reads were aligned by using reference genome GRCh38 to generate unique molecular identifier (UMI) count matrices. Data from sequenced samples were then aggregated by the aggr pipeline in Cell Ranger. After obtaining the feature-barcode matrix originated from 42,119 cells, the R package Seurat was applied for downstream analyses^57^, including quality control, clustering, cell annotation, and identifying DEGs. The quality of cells was assessed by the total UMI counts per cell, the number of detected genes per cell, and the proportion of mitochondrial gene counts. Cells of low quality were filtered out if their library sizes or the number of detected genes were smaller than the median of all cells minus 3× median absolute deviation (MAD). Cells with a higher proportion of expressed mitochondrial genes were discarded with the threshold set as 20% for all cells, while in the case of immune cells, the cutoff value was 10%. We then applied the method previously described by Pijuan-Sala^58^ to identifying doublets. In brief, the doublet scores representing the probability of being doublet for each cell were calculated by the function computeDoubleDensity in the R package scDblFinder. Cells were clustered separately in each sample by building a Shared Nearest Neighbor (SNN) Graph and applying the Louvain clustering algorithm. Then, every cluster underwent the same clustering procedure to make smaller clusters. The median doublet scores of each small cell cluster were fit into a median-centered MAD-variance normal distribution. Cells comprising the cell cluster with a median doublet score at the extreme upper end of this distribution were labeled as doublets. In the final step, cells from all samples were clustered using the same measure as described above. The proportions of cells labeled as doublets in each cell cluster were fit into a median-centered MAD-variance normal distribution model. Cell clusters with extremely high values were considered as doublets-containing cell groups. This doublets-calling procedure identified 4810 doublets. After filtering out low-quality cells and doublets, a total of 33,856 cells were retained and put into downstream analyses.

### Unsupervised clustering and marker identification

Highly variable genes used for unsupervised clustering were identified by the function FindVariableFeatures in the R package Seurat. We used vst selection method with the default parameters to pick top 1000 highly variable features (HVF) for dimensionality reduction. Feature counts were normalized by using library size per cell and natural-log transformed. The log-normalized data were then scaled to mitigate the variability attributed to UMI count distribution, mitochondrial gene expression, ribosomal gene expression, and G2/M and S cell cycle phase scores estimated by the function CellCycleScoring. Principal Component Analysis was then performed based on the expression matrix of HVF. Top 43 principal components (PCs) were selected for graph-based clustering, the same procedure as used to call doublets, with the resolution set as 0.8. For the clustering the subset of progenitor cells, top 40 PCs were used and the resolution parameter was equal to 0.4. For all immune cells, HVF was determined by the combination of the vst method and the depth-adjusted negative binomial model from R package M3Drop^59^. The clustering procedure was based on the top 43 PCs with resolution set as 0.7. For the subset of B cells, the clustering procedure was performed with top 40 PCs used and the resolution set as 1. For the clustering of NK and T cells, top 40 PCs were used and resolution parameter equaled 0.7. The subsets for the progenitor cells, immune cells, and NK/T cells were generated from the unsupervised clustering results by Seurat function subset. UMAP plots were generated to visualize the results of graph-based clustering by Seurat function RunUMAP.

Clusters were manually annotated based on overlapping known marker genes and the expression patterns of each cell type were visualized in the form of heatmap. We manually assorted all cells into 9 groups by their marker genes, including adipose progenitor cell (*CD34*, *PDGFRA*, *THY1*), T cell (*CD3*), macrophage/monocyte (*CD163*, *CD68*, *AIF1*, *CYBB*), B cell (*CD19*, *CD79A*, *CD79B*), endothelium (*PECAM1*, *CDH5*), neutrophil (*FCGR3B*, *MNDA*)^60^, smooth muscle cell (*PLN*, *CASQ2*, *ACTA2*), NK cell (*NCAM1*, *GNLY*, *GZMB*) and plasmacytoid DC (*IL3RA*, *CLEC4C*)^61^. Adipose progenitor cells were manually grouped into 6 groups: visceral preadipocyte (*APOD*, *FMO2*)^12^, subcutaneous preadipocytes (*FABP4*, *APOE*)^14^, stem-cell like (*CD55*, *DPP4*)^14^, fibroblast-like (*COL1A1*, *COL3A1*, *COL6A1*), mesothelial-like (*ITLN1*, *MSLN*)^62^ progenitor cell, inflammatory progenitor cell (*CCL2*, *CXCL2*, *TNFRSF12A*), and hematopoietic stem cell (*PTPRC*, *CCL5*, *IL7R*)^17^.

Analysis of cluster-specifically highly expressed genes were determined by Seurat function FindAllMarkers with the Wilcox test^63^. Genes detectable in a minimal 50% cells in either of the tested cell populations with > 0.25-fold-change (on log_2_ scale) on average between groups were considered for analysis. Top 5 genes ranked by fold-change were selected for visualization in the form of heatmaps. Violin plots, heatmaps and UMAP plots were generated by applying the Seurat toolkit VlnPlot, DoHeatmap, and Dimplot, respectively.

### Differential expression analyses

To investigate the variation of gene expression level across depots and disease conditions, we applied pseudo-bulk differential expression analyses. Raw counts of genes across all cells in the cell cluster of interest within each sample were aggregated as the total counts to generate pseudo-bulk samples. DESeq2 R package was then used to compare gene expression levels between cell populations from SAT and VAT or cachectic and non-cachectic patients with default parameters. Genes with the FDR adjusted *P*-value less than 0.05 were considered as DEGs, which were then used for further analyses.

### Integration of public scRNA-seq data

To compare the gene expression of progenitor cell types identified in previous studies, we used the orthologous gene annotation from ENSEMBL 95 of the BioMart for scRNA-seq data from SVF of mouse adipose tissue. We only plotted genes for which an ortholog between mouse and human existed. Integration of data was performed by Seurat function MapQuery.

### Cell development trajectory

The cell development trajectory inference was performed on the subset of CD8^+^ T cells from VAT based on the unsupervised clustering of NK/T cells by using the R package Monocle^64^. Clusters with a tiny number of cells were discarded (Clusters 7, 9, 11), leaving 7121 cells put into the pseudotemporal analysis. Genes used to order cells were selected by the Seurat function FindAllMarkers with “Wilcox” test being applied. A total of 1106 genes were identified as significantly variable genes between clusters. In order to reduce the impact of confounding factors during dimensionality reduction, we transformed data with the linear model to subtract the effect of UMI counts per cell, mitochondrial and ribosomal gene expression, and G2/M plus S cell cycle phase scores prior to the clustering. The DDRTree method was used to perform dimensionality reduction. The plots to show the trajectory with ordered cells were generated by the Monocle function plot_cell_trajectory. Once each cell was assigned a pseudo-time value, which inferred the cell development progress, Monocle function differentialGeneTest was applied to find genes that changed steadily along the cell trajectory.

### SCENIC analysis

To evaluate the activity of transcription factor (TF) regulons in each preadipocyte cluster in both SAT and VAT, we applied SCENIC workflow with default parameters^23^. In brief, raw count matrix of progenitor cells was used as input and co-expression modules were identified by GENIE3, followed by the *cis*-regulatory motif enrichment analysis done by RcisTarget. The TF regulon was defined as the set of genes enriched with the motif of each TF and the score of regulon activity was calculated by AUCell. The AUC scores of TF regulons of interest were then visualized by Dimplot function in Seurat package.

### Definition of cell scores and signature

To further gain an insight into macrophage polarization, M1-like and M2-like gene signature for enrichment analysis were obtained from literature (Supplementary Table S5)^65^. We adopted the Gene Set Variation Analysis algorithm^66^ to calculate the enrichment score (ES) against reference gene list per cell by the R package GSVA and compared the medians of ESs between macrophages from cachexia-specific VAT and non-cachexia-specific VAT. Expression of polarization signature was summarized as the mean of zero-centered scaled count data and expression levels of DEGs in the signature gene list were illustrated by box plots. For the estimation of cytolytic and the activation/resting state of CD8^+^ T cell, we firstly filtered out genes with low expression (< 1 count) in the reference list (Supplementary Table S5), followed by computing the sum of log-normalized counts of the remaining genes.

### Pathway analysis

To generally compare the biological alterations in the transcriptional level between adipose progenitor cells from VAT and SAT, we applied GSEA^67^ to investigate the enrichment of DEGs against canonical pathways gene sets derived from the KEGG pathway database from MSigDB v7.2 (www.gsea-msigdb.org). The same method was applied when investigating the functional differences between genes highly expressed in cachexia-specific cluster 8 and that in the other clusters from SAT. In addition, Gene Ontology (GO) analyses^68^ were performed to reveal the classification of DEGs based on GO distribution, and the subset of biological process classification was visualized in the form of dot plot. The R package clusterProfiler^69^ was used to conduct GSEA and GO analyses as well as visualization of the results.

### Deconvolution of adipose tissue microarray data

To validate the changes in immune cell compositions in VAT during CAC, we performed deconvolution analyses on publicly available microarray data from omental adipose tissue of patients with gastric or esophageal cancer (GSE131835)^70^. We used online deconvolution toolkit CibersortX (https://cibersortx.stanford.edu) to generate gene expression profiles of immune cells manually annotated in our scRNA-seq data, followed by the partial deconvolution approach for the imputation of cell fractions among all immune cells^71^. The cell compositions of cytotoxic CD8^+^ T cells and macrophages were then compared between patients in the cachexia group and the weight stable group.

### Cell–cell interaction analysis

In order to investigate the relationship between the dynamic changes of infiltrating T cells in VAT and the difference in polarization preference of macrophages, we performed cell–cell interaction analyses by using CellPhoneDB v.2.0^52^, a public repository made to decode significant ligand-receptor interactions between two cell types. The interaction scores were estimated separately between cell types in VAT under cachexia or not. Briefly, the interaction score referred to the mean expression of the receptor by one cell type and corresponding ligand by the other cell type. Ligand-receptor pairs with significant cell-type specificity were considered as interactions with biologically relevance. The amounts of ligand-receptor pairs between every two immune cell types were shown in the form of heatmap plot by using the pheatmap function in R. Connectome web analysis^53^ was applied with default settings to visualize the abundance of upregulated interactions among immune cells in the context of cachexia. To determine the differential interactions involving CD8^+^ T cells in VAT between cachexia and non-cachexia group, ligands and receptors with absolute log_2_ fold-change > 0.3 and *P*-value (determined by Wilcoxon Rank test) < 0.01 were selected. Expression index was determined as the normalized expression level of the indicated gene multiplied by the sign of log_2_ fold-change.

### Statistics

Two-tailed Wilcoxon rank-sum test was applied when we compared the expression level of individual genes involved in M1-like, M2-like signature, and the signature scores between cell groups. The results of qPCR analyses were compared using the Two-Way ANOVA with post hoc Dunnett’s multiple-comparison tests as indicated in the figure legend. R v.4.0.3 was used to perform all statistical analyses.

## Supplementary information

Supplementary information

## Data Availability

The raw data and processed data of scRNA-seq in present work have been deposited at National Omics Data Encyclopedia (Project ID: OEP003500).
