# Supplementary material for: Single-cell sequencing unveils key contributions of immune cell populations in cancer-associated adipose wasting
Source: Cell Discov. 2022 Nov 15;8:122. doi: 10.1038/s41421-022-00466-3 (PMC9663454; doi:10.1038/s41421-022-00466-3)
Supplement: Supplementary file 1 — Supplementary information [file 41421_2022_466_MOESM1_ESM.pdf]

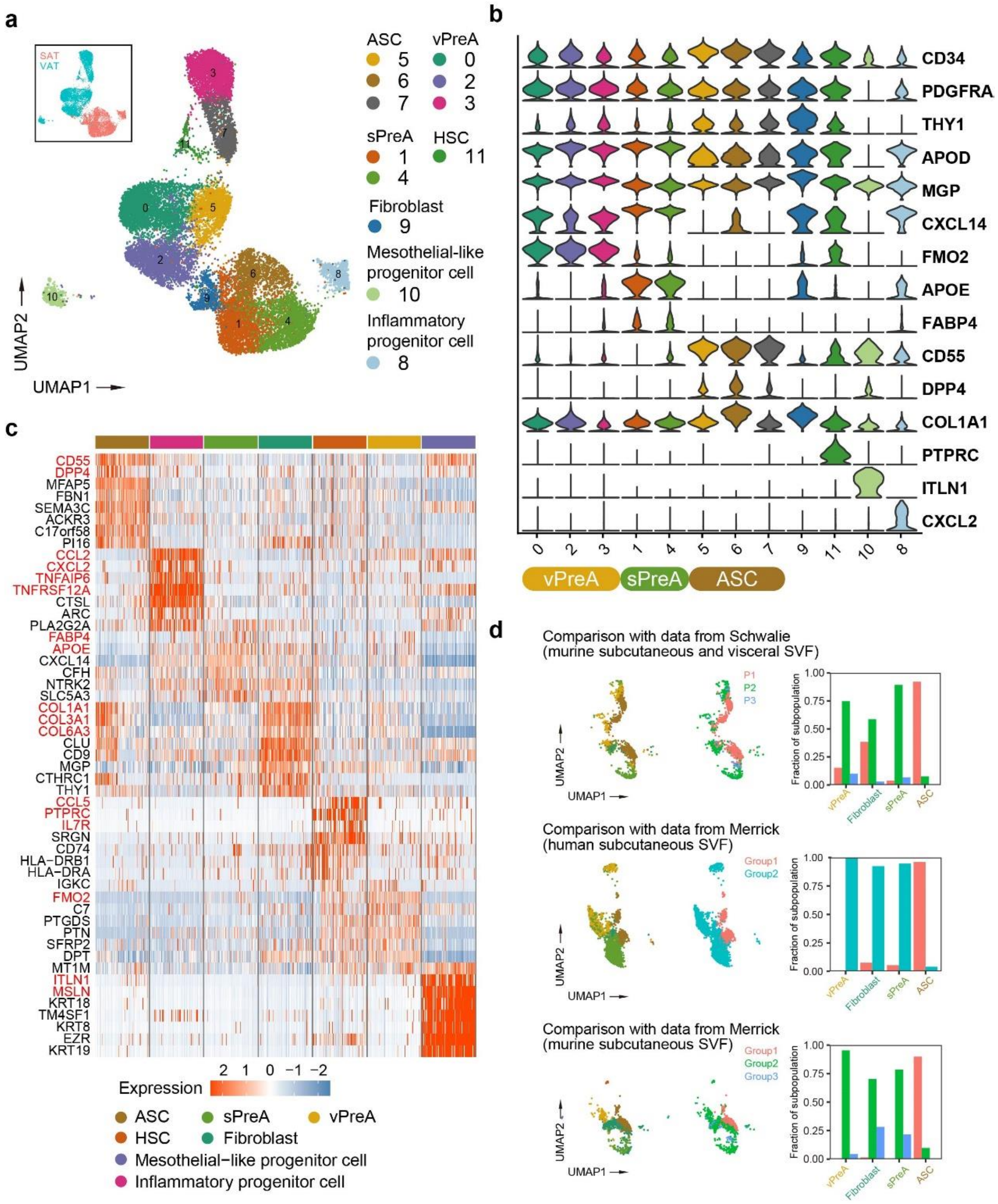

Supplementary Fig. 1 Manual annotations of progenitor cells identified by unsupervised clustering. (**a**) UMAP plot of the cell clusters identified by unsupervised clustering method. Clusters were grouped by manual annotation. (**b**) Violin plot showing the expression of marker genes within each cell type. (**c**) Heat map (blue-to-red) of scaled expression of marker genes for each cell type and top five differentially expressed genes (DEGs) for each indicated cell type. Genes shown in red were marker genes used for manual annotations. (**d**) UMAP plot showing the overlapping between progenitor cell types annotated in our study (left) and adipogenic progenitor cell groups (reference) identified by previous studies (middle). Bar plot (right) showing the fraction of reference cell groups in each cell type.

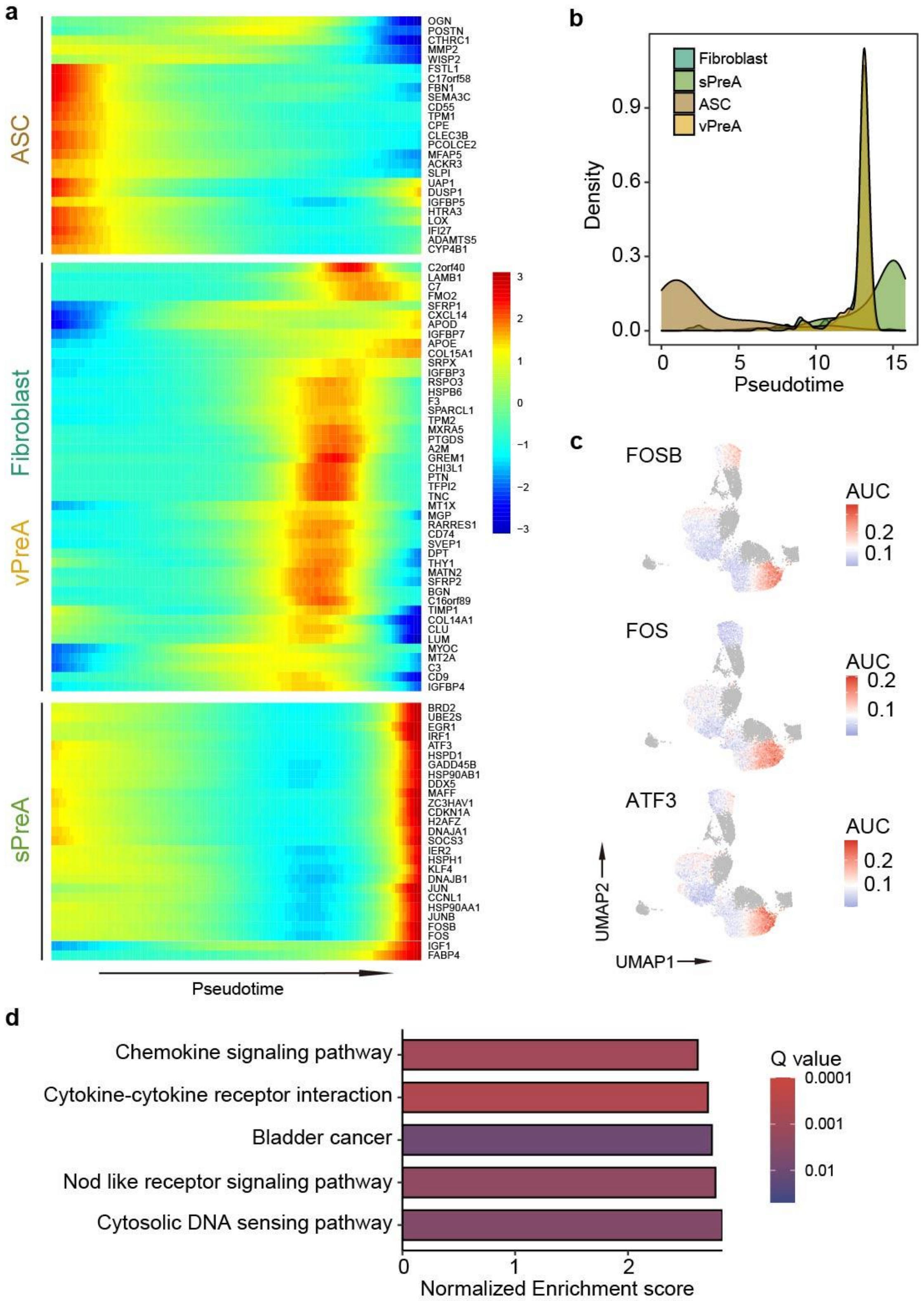

Supplementary Fig. 2 Genes differentially expressed in adipo-genic progenitor cells. (**a**) Heatmap showing the dynamic changes in gene expression along the pseudo-time. Cell trajectories towards each cell type were labelled. (**b**) Density plot showing the distribution of progenitor cell types along pseudo-time. (**c**) UMAP plot showing the AUC scores of indicated transcription factor regulons of preadipocytes. (**d**) Dot plot showing the top 5 KEGG pathways enriched among up-regulated genes in VAT preadipocytes.

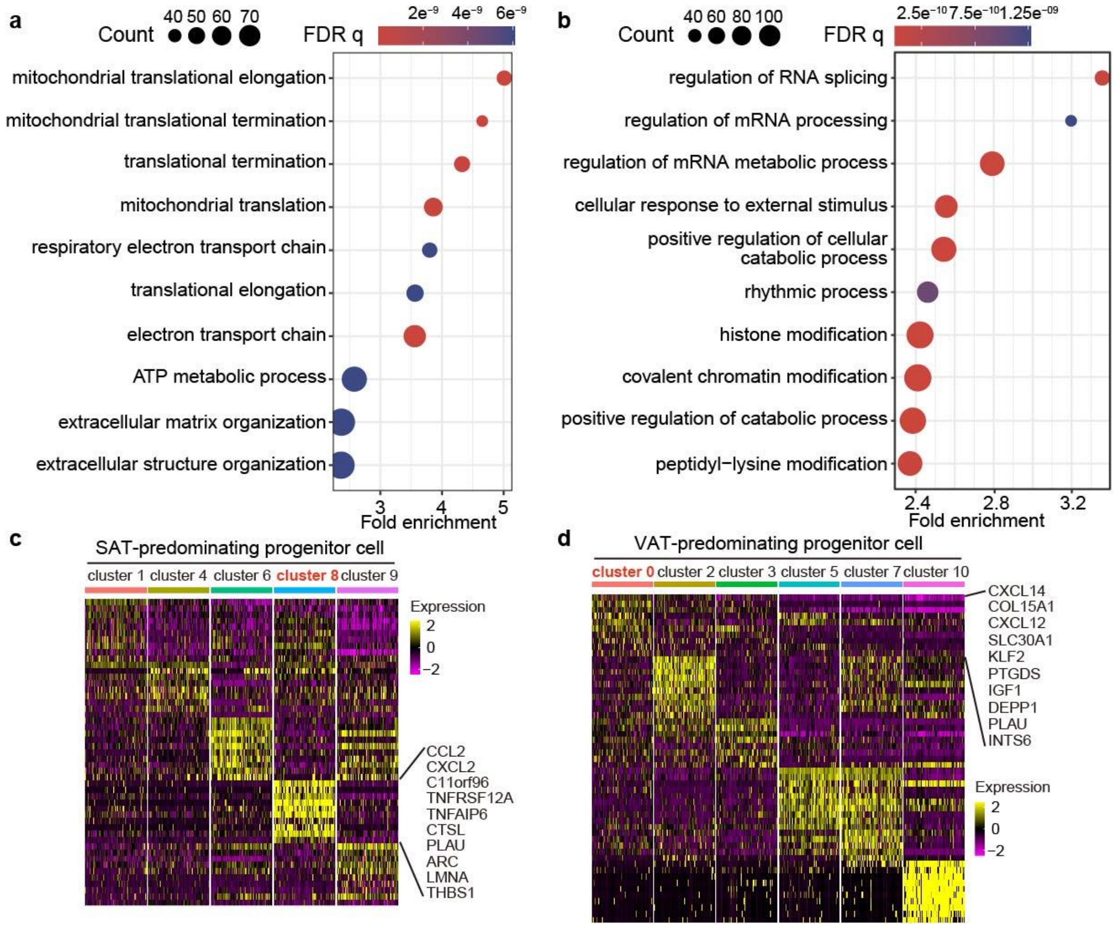

Supplementary Fig. 3 Functional annotations of disease- and depot-dependent DEGs. (**a**, **b**) Dot plots showing the enriched biological processes among down-regulated (**a**) and up-regulated (**b**) genes in SAT preadipocytes from patients with cachexia. (**c**) Heatmap showing the expression of top 10 highly expressed genes in each SAT-predominating cell cluster. Genes with higher expression in cachexia-specific cluster 8 were labeled. (**d**) Heatmap showing the expression of top 10 highly expressed genes in each VAT-predominating cell cluster. Genes with higher expression in cachexia-dominant cluster 0 were labeled.

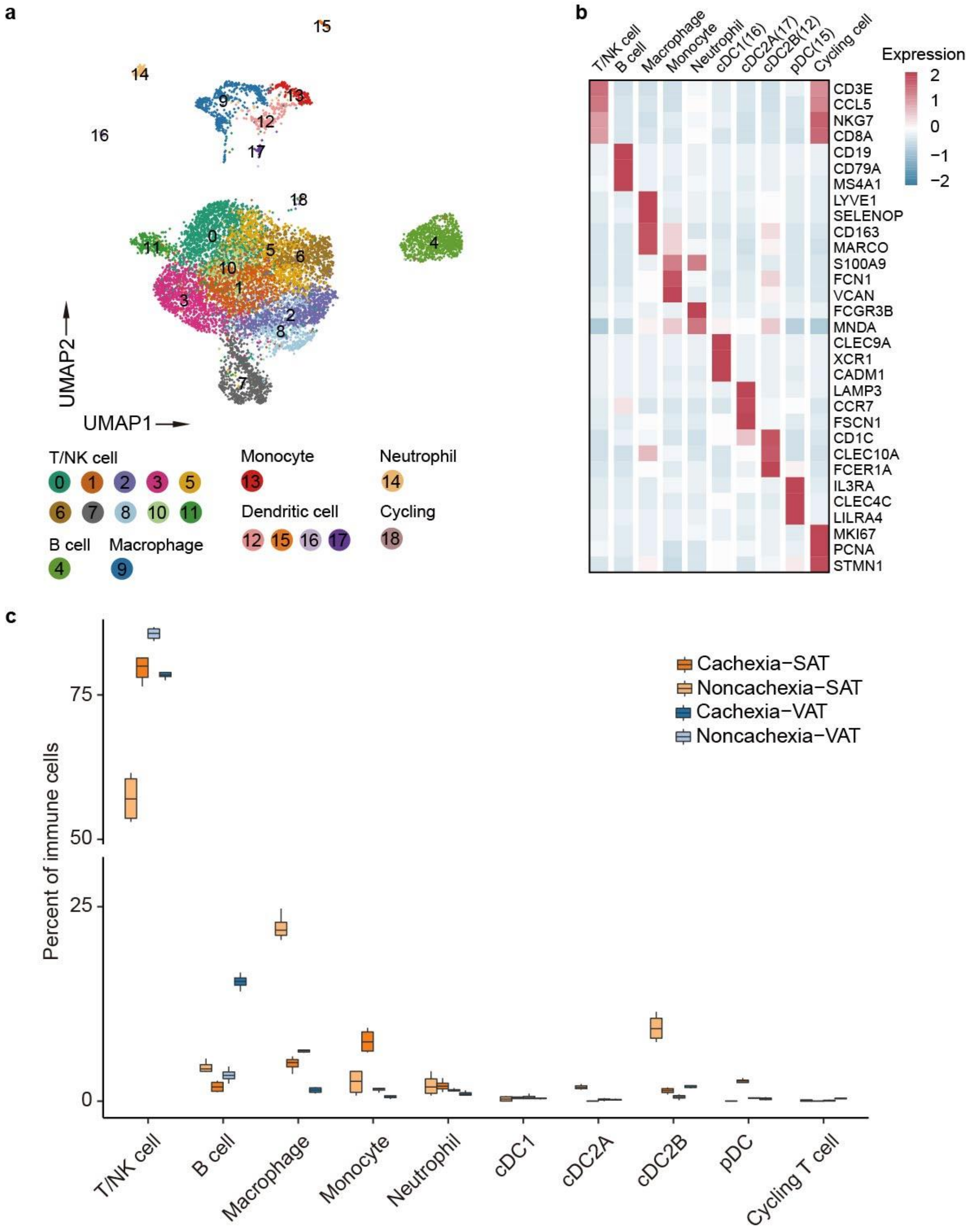

Supplementary Fig. 4 Manual annotation of immune cells. (**a**) UMAP plot showing 19 clusters identified by unsupervised clustering. Cell clusters were grouped based on manual annotation. (**b**) The heatmap showing the expression level of selected marker genes in each cell type. (**c**) Box plot showing the fraction of all immune cell types in each group. Dots representing the total number of qualified cells in each group. NK cell: natural killer cell, cDC: conventional dendritic cell, pDC: plasmacytoid dendritic cell.

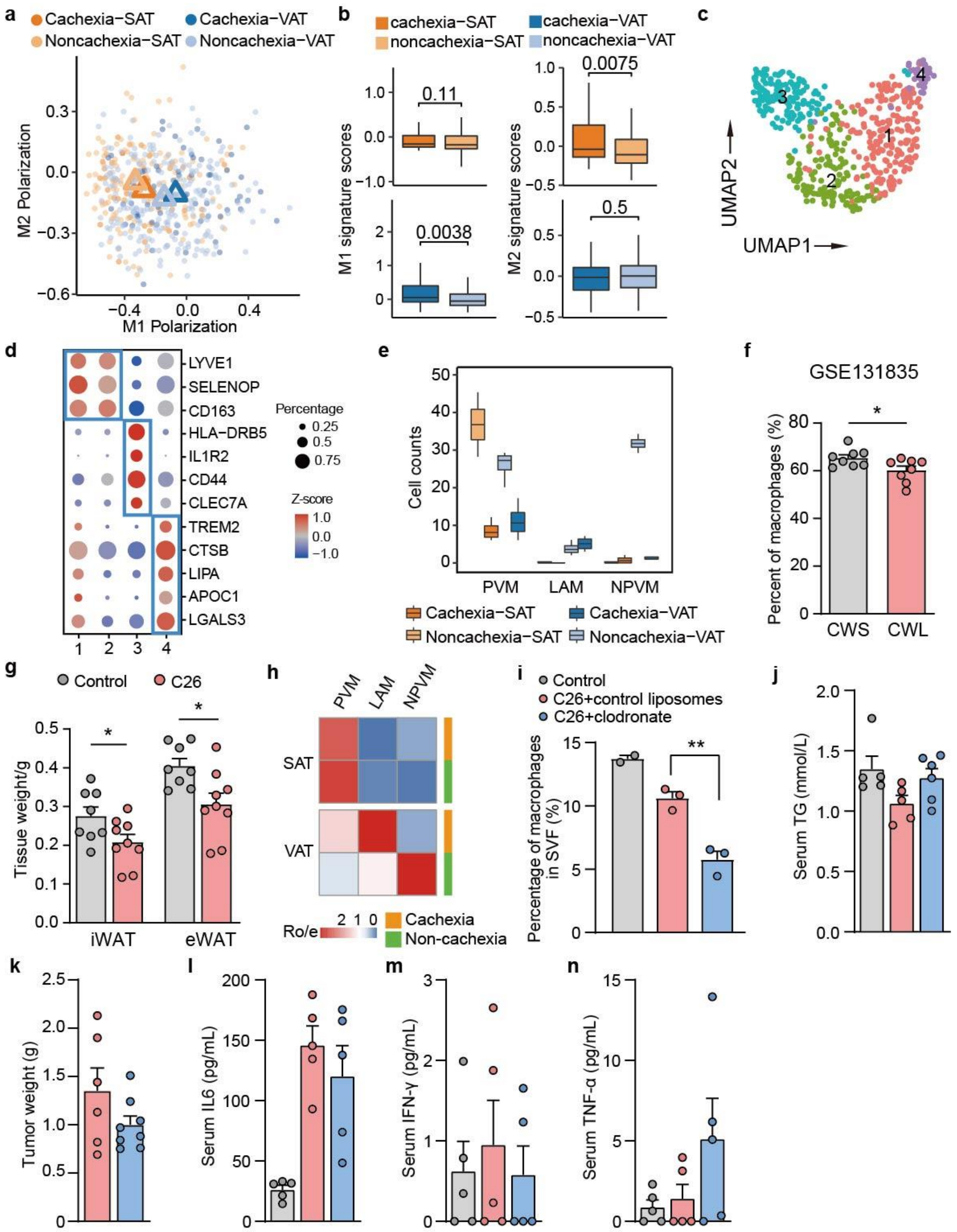

Supplementary Fig. 5 Functional analyses of adipose tissue macrophages under cachexia development. (**a**) Scatter plot of M1- and M2-like scores calculated by Gene Set Variation Analysis for the individual macrophage. The triangular sign represented the median score of M1- and M2-like signatures for cells in the corresponding group. (**b**) The mean scaled expression level of M1- (left) and M2-like (right) signature genes of macrophages from VAT and SAT were shown in the box plots. *P* values calculated by the Wilcoxon rank-sum test were labeled on the top. (**c**)UMAP plot showing unsupervised clustering of macrophages. (**d**) Bubble heatmap showing the expression of selected marker genes of each macrophage cluster. (**e**) Bar plot showing the number of cells in each macrophage subpopulation in each group. (**f**) Bar plot showing the inferred percentage of adipose tissue macrophages in both patients with stable weight (CWS) and weight loss (CWL). (**g**) The weight of inguinal and epididymal WAT of control group and C26 cachexia model. (n = 8-9/group) (**h**) The Ro/e analysis of macrophage

subpopulations. (**i**) Bar plot showing the proportions of macrophages in SVF of epididymal WAT in each group (n = 2-3/group), determined by flow cytometry. (**j**) Bar plot showing the concentration of serum triglycerides in each group. (**k**) Bar plot showing the tumor weight in C26 model. (**l**-**n**) Bar plot showing the concentrations of serum cytokines IL-6 (**l**), IFN-γ (**m**), and TNF-α (**n**) in each group (n = 5/group).

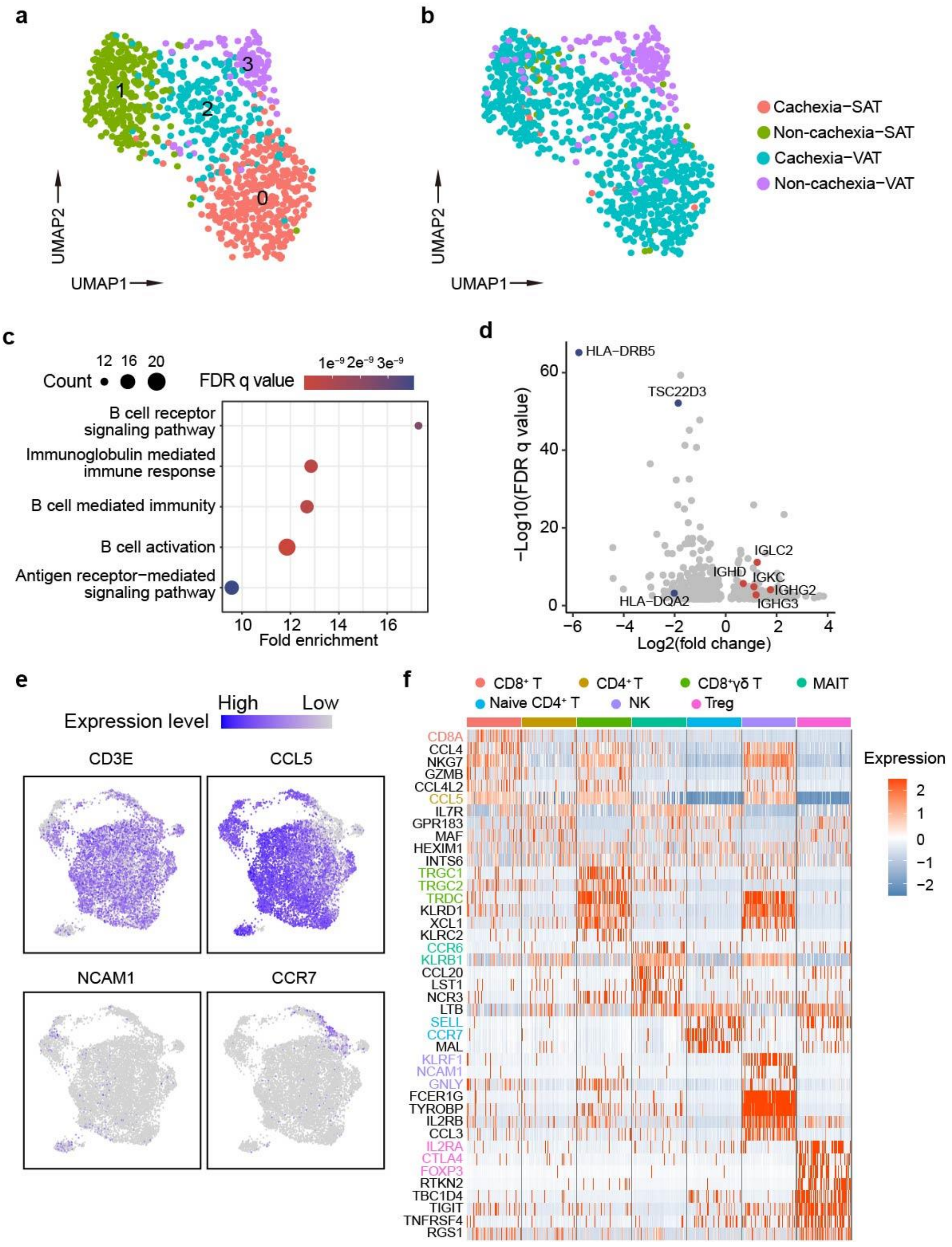

Supplementary Fig. 6 Annotations for B cell and T/NK cell. (**a**) UMAP plot showing the unsupervised clustering of B cells. (**b**) UMAP plot showing the origins of B cells by the depot and disease condition. (**c**) Dot plot showing the results of GO analysis of genes up-regulated in VAT B cells from patients with CAC. (**d**) Volcano plot showing the differentially expressed genes of VAT B cells between patient with (n = 4) or without (n = 4) CAC. Red dots denoting indicated genes up-regulated in CAC group, while blue dots denoting those don-regulated in CAC group. (**e**) UMAP plot showing the expression of selected canonical marker genes for T and NK cells. (**f**) Heat map (blue-to-red) of scaled expression of marker genes used for manual annotations and the most differentially expressed genes for each indicated cell type. Genes used for manual annotation were shown in the corresponding colors. NK cell: natural killer cell, MAIT: mucosa-associated invariant T cell, Treg: regulatory CD4^{+} T cell.

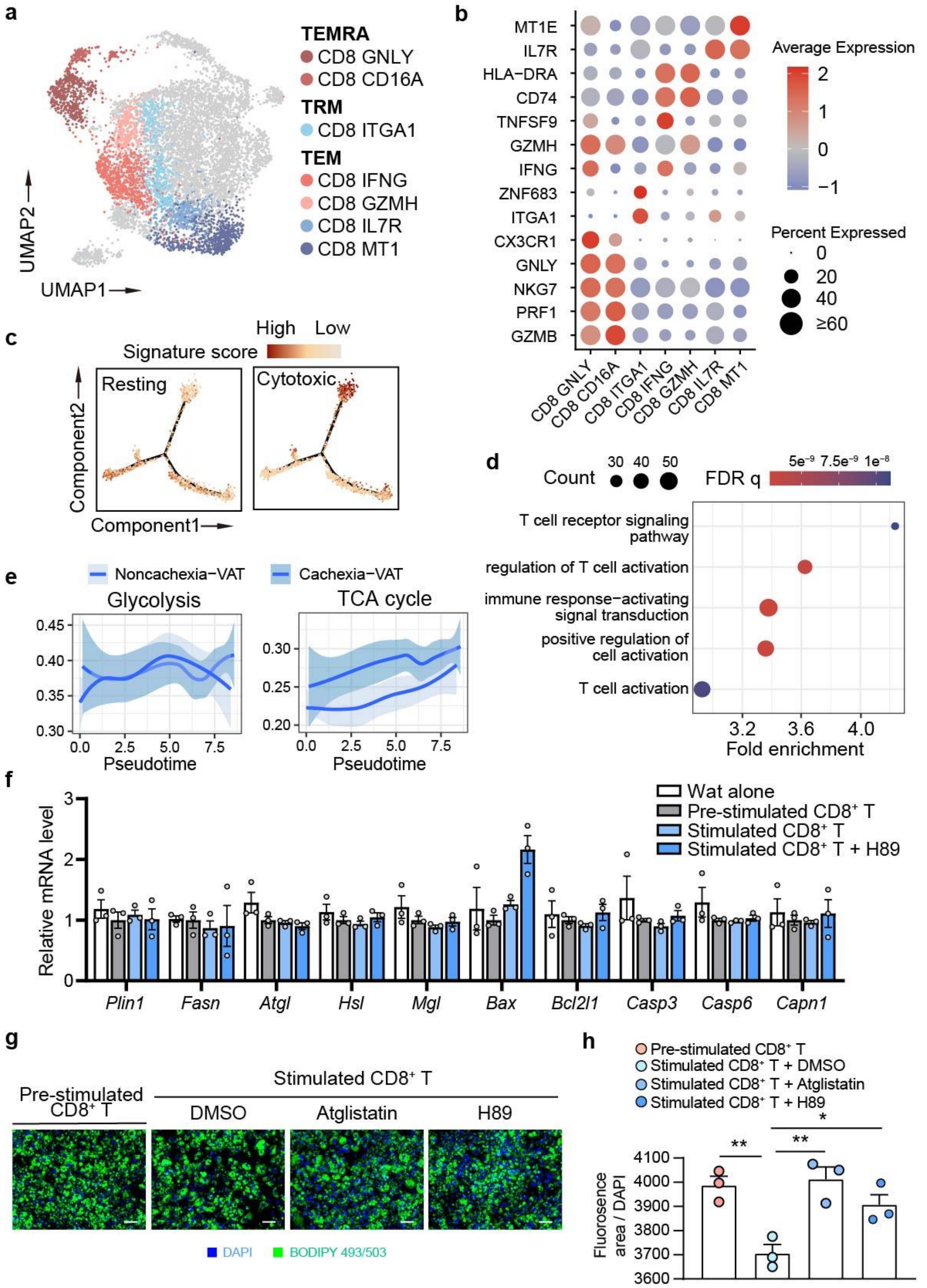

Supplementary Fig. 7 Effector CD8$^+$ T cells accelerated lipolysis via activation of PKA. (**a**) UMAP plot showing CD8$^+$ T cell clusters identified by unsupervised clustering. (**b**) Dot plot showing the expression of marker genes in each manually annotated cell cluster. (**c**) Pseudo-time trajectory map with cells colored by the scores of CD8$^+$ T cell cytotoxicity signature (left) and resting CD4/8$^+$ T cell signature (right). Color saturation indicates the magnitude of the signature gene expression. (**d**) Dot plot showing the enriched biological processes among up-regulated genes of CD8$^+$ T cells in VAT from patients with cachexia. (**e**) Two-dimensional plots showing the dynamic expression of glycolysis (left) and TCA cycle (right) related genes during the T cell transitions along the pseudo-time. (**f**) Bar plot showing the relative mRNA expression

level of lipid metabolism and cell death related genes in cultured adipocytes under different culture conditions. The expression levels are normalized by the average expression in the pre-stimulated $CD8^+$ T cells co-culturing group. Bars indicate mean ± SEM. (n = 3/group) (**g**) Representative images of adipocytes *in vitro* under different culturing conditions stained with BODIPY 493/503 for neural lipid droplets, with DAPI used to label the cell nuclei. Scale bar: 100μm. (**h**) Quantification of green fluorescence area, divided by the number of cell nuclei indicated by DAPI, in each co-culturing group. Bars indicate mean ± SEM. P values were obtained by One-Way ANOVA with *post hoc* Dunnett's multiple-comparison correction tests. *: $p < 0.05$, **: $p < 0.01$. (n = 3/group)

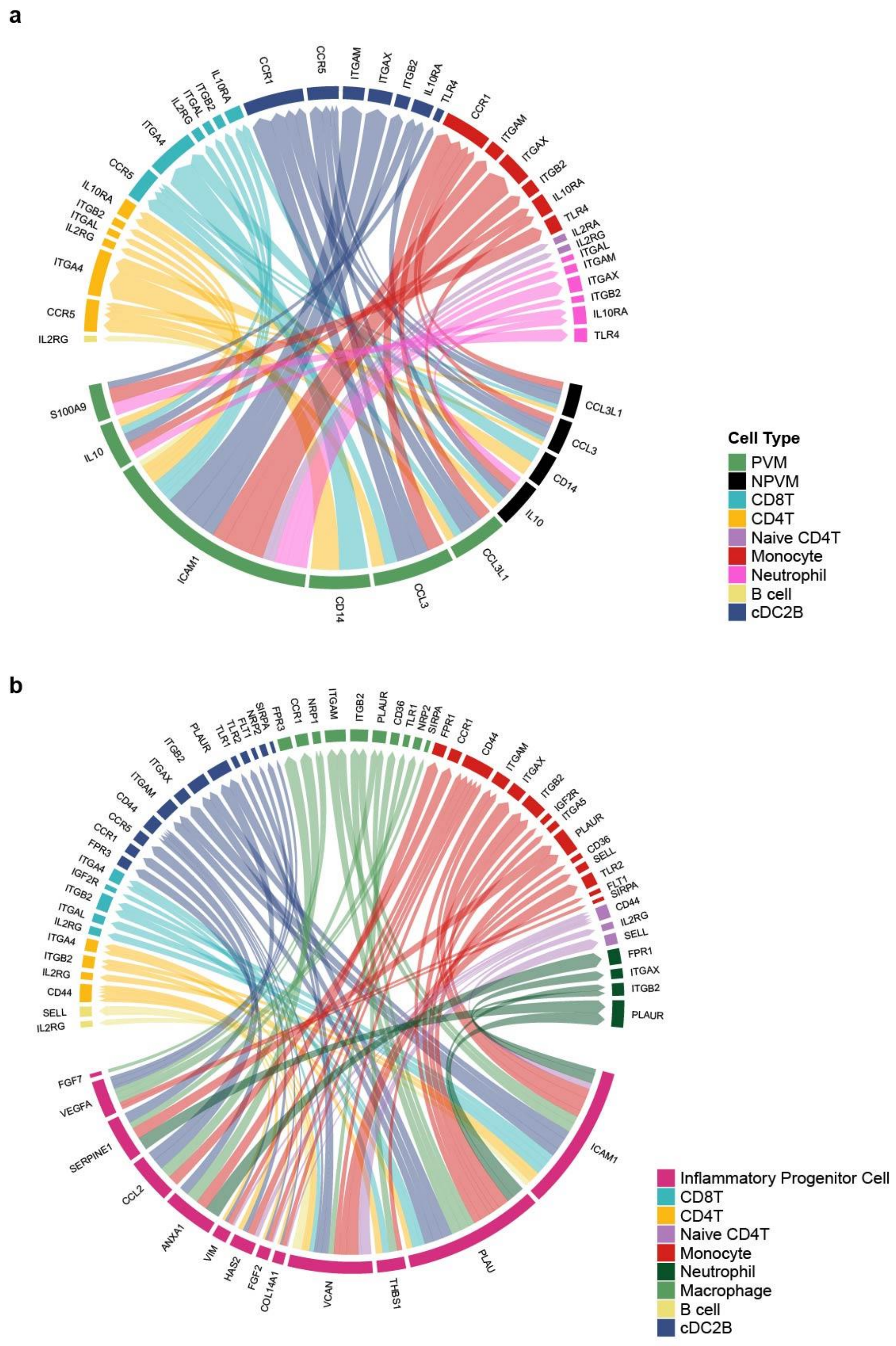

Supplementary Fig. 8 Connectome analyses of ligand-receptor interaction pairs. (**a**) Circos plot showing the extensive interactions between the macrophages and immune cells in VAT by Connectome web analysis. Arrows originate from ligands in inflammatory progenitor cells and end at receptors in the immune cells. (**b**) Circos plot showing the extensive interactions between the inflammatory progenitor cells and immune cells by Connectome web analysis.

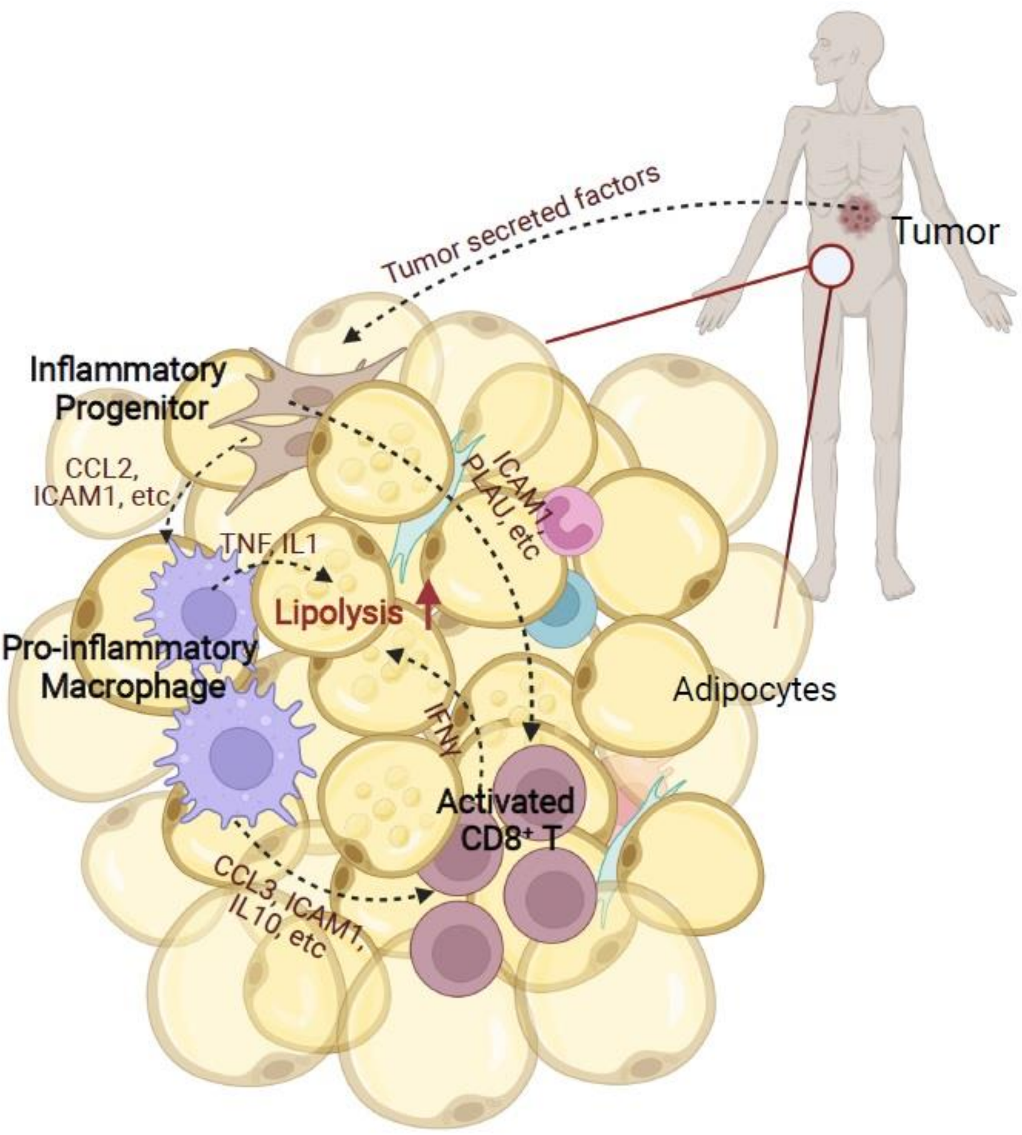

Supplementary Fig. 9 Proposed cellular alterations in adipose tissues from cachexia patients. In patients with CAC, tumor derived factors may stimulate the chemokine gene expression in a specific subset of adipose progenitors, which may cause a pro-inflammatory transition of macrophages and $CD8^+$ T cells through cytokines such as CCL2. Pro-inflammatory macrophages can also activate and recruit more $CD8^+$ T cells via CCL3, IL10, etc. Both pro-inflammatory macrophages and activated $CD8^+$ T cells can stimulate the catabolism in adipose tissues via cytokines IFN-γ, TNF-α, and IL-1, leading to severe adipose wasting. Together, these cellular transitions unveil causative mechanisms underlying the chronical inflammation and adipose wasting in CAC. Created with www.BioRender.com

Supplementary Table S1a: Distribution of progenitor cell types across different depots and disease conditions

	cachexia-SAT	noncachexia-SAT	cachexia-VAT	noncachexia-VAT
Adipose stem cell	749(16.9%)	620(17.4%)	1283(22.3%)	1595(25.4%)
sPreA	2865(64.7%)	2074(58.1%)	18(0.3%)	42(0.7%)
vPreA	5(0.1%)	227(6.4%)	4285(74.5%)	4075(64.9%)
Fibroblast	10(0.3%)	623(17.4%)	19(0.3%)	15(0.2%)
HSC	8(0.2%)	25(0.7%)	111(1.9%)	128(2.0%)
Inflammatory Progenitor Cell	793(17.9%)	2(0.1%)	0	3(0.1%)
Mesothelial-like Progenitor Cell	0	0	39(0.7%)	424(6.8%)
Total	4430	3571	5755	6282

Supplementary Table S1b: Distribution of immune cell types in each group.

	cachexia-SAT	noncachexia-SAT	cachexia-VAT	noncachexia-VAT
T/NK cell	614(79.7%)	380(57.1%)	4022(78.6%)	3401(85.6%)
B cell	14(1.8%)	29(4.4%)	782(15.3%)	131(3.3%)
Macrophage	37(4.8%)	148(22.2%)	73(1.4%)	255(6.4%)
Monocyte	58(7.5%)	17(2.6%)	28(0.5%)	59(1.5%)
Neutrophil	15(1.9%)	15(2.3%)	47(0.9%)	56(1.4%)
cDC1	3(0.4%)	2(0.3%)	17(0.3%)	21(0.5%)
cDC2A	0	12(1.8%)	10(0.2%)	8(0.2%)
cDC2B	10(1.3%)	62(9.3%)	102(2.0%)	22(0.6%)
pDC	19(2.5%)	0	16(0.3%)	17(0.4%)
Cycling T cell	0	1(0.2%)	22(0.4%)	4(0.1%)
Total	770	666	5119	3974

Supplementary Table S2: Differentially expressed genes in Inflammatory Progenitor Cells compared with other progenitor cells.

geneID	pValue	Log2FC	percent.1	percent.2	FDR q value
CCL2	0	3.364951159	0.931	0.347	0
CXCL2	0	3.280601194	0.627	0.127	0
TNFAIP6	0	3.138755626	0.871	0.268	0
TNFRSF12A	0	3.110056096	0.929	0.142	0
PLA2G2A	3.75E-221	2.968143243	0.781	0.357	8.10E-217
CTSL	0	2.861627648	0.982	0.757	0
ARC	2.66E-305	2.67355011	0.605	0.152	5.75E-301
LMNA	0	2.506438043	0.997	0.864	0
PLIN2	1.99E-128	2.501752278	0.788	0.606	4.29E-124
PTX3	2.67E-64	2.450126383	0.286	0.105	5.76E-60
CXCL8	1.45E-57	2.343496141	0.17	0.046	3.12E-53
PLAU	1.92E-92	2.336733279	0.558	0.311	4.14E-88
CXCL3	1.90E-241	2.308142927	0.213	0.02	4.09E-237
SERPINE1	0	2.240371523	0.638	0.139	0
ANXA1	1.02E-300	2.216465425	0.997	0.95	2.20E-296
UGDH	0	2.185053783	0.887	0.398	0
GPRC5A	0	2.116842775	0.929	0.325	0
HMOX1	1.99E-109	2.093068843	0.516	0.231	4.30E-105
ANGPTL4	1.33E-57	2.008261998	0.559	0.38	2.87E-53
GEM	0	1.98562683	0.772	0.267	0
C11orf96	1.10E-213	1.92704514	0.936	0.618	2.37E-209
CYTOR	2.18E-238	1.873601747	0.782	0.342	4.70E-234
NNMT	1.35E-268	1.841688264	0.975	0.769	2.92E-264
MMP3	8.84E-220	1.841316785	0.114	0.005	1.91E-215
IFI16	6.42E-275	1.834096933	0.955	0.709	1.38E-270
DDX21	0	1.799158891	0.97	0.546	0
ABL2	0	1.79344346	0.763	0.23	0
ARID5B	1.90E-260	1.779158195	0.972	0.755	4.11E-256
CD44	0	1.769548616	0.971	0.627	0
MEDAG	1.40E-284	1.752613065	0.937	0.559	3.03E-280
TUBA1C	0	1.716134261	0.815	0.241	0
IER3	4.96E-80	1.714067732	0.573	0.295	1.07E-75
DAB2	1.27E-212	1.705859691	0.917	0.666	2.74E-208
SPSB1	4.73E-297	1.691507935	0.778	0.279	1.02E-292
ICAM1	9.34E-210	1.687107954	0.544	0.155	2.01E-205
CCDC71L	4.80E-128	1.682043134	0.645	0.319	1.04E-123
KDM6B	0	1.665561505	0.835	0.295	0
IL1RL1	0	1.658352477	0.221	0.012	0
THBD	1.54E-87	1.648613693	0.272	0.08	3.31E-83
PXDC1	2.54E-275	1.619006813	0.863	0.421	5.47E-271
SLC39A14	0	1.59705414	0.729	0.126	0
FOSL1	0	1.593470285	0.707	0.085	0
HMGA1	0	1.576167238	0.536	0.063	0
GCLM	0	1.566158787	0.762	0.228	0
EMP1	2.22E-198	1.563421039	0.942	0.729	4.78E-194
SLC4A7	0	1.539574702	0.678	0.18	0
RGCC	3.90E-265	1.524948797	0.665	0.188	8.42E-261
GNPNAT1	1.27E-230	1.518354567	0.535	0.142	2.75E-226
LMCD1	3.70E-135	1.472385289	0.373	0.107	7.97E-131

RAN	1.16E-256	1.449748472	0.931	0.648	2.50E-252
NABP1	2.46E-218	1.446907457	0.791	0.338	5.32E-214
ACSL4	3.71E-295	1.441327326	0.699	0.206	8.01E-291
HAS2	1.92E-205	1.427358613	0.617	0.201	4.13E-201
HSPD1	3.53E-132	1.422915017	0.9	0.684	7.61E-128
SOD2	4.99E-105	1.41968086	0.888	0.72	1.08E-100
IL1R1	8.58E-253	1.408601502	0.912	0.544	1.85E-248
LRRFIP1	0	1.40076672	0.722	0.205	0
TXN	3.26E-129	1.355117613	0.926	0.788	7.03E-125
HSP90AB1	1.16E-225	1.318036168	0.997	0.929	2.49E-221
AKR1C1	1.08E-135	1.31429318	0.748	0.424	2.33E-131
HSPH1	3.91E-91	1.301362621	0.805	0.556	8.44E-87
ADAMTS4	3.85E-121	1.293202203	0.412	0.129	8.30E-117
GNL3	2.31E-262	1.2920874	0.712	0.246	4.99E-258
RUNX1	1.97E-299	1.292061754	0.708	0.208	4.26E-295
NAMPT	8.00E-196	1.287164216	0.952	0.603	1.73E-191
GLIS3	0	1.277727833	0.543	0.09	0
NCL	4.98E-204	1.273423334	0.95	0.741	1.07E-199
VASN	8.44E-185	1.268386423	0.689	0.278	1.82E-180
VMP1	1.44E-162	1.265469838	0.802	0.417	3.11E-158
WDR43	0	1.262816644	0.699	0.201	0
VCAN	3.86E-151	1.261728171	0.835	0.462	8.33E-147
PLAUR	2.89E-132	1.253083991	0.647	0.298	6.24E-128
CYCS	3.02E-209	1.236358091	0.876	0.464	6.52E-205
MAT2A	6.32E-177	1.231568132	0.827	0.447	1.36E-172
MLF1	4.87E-237	1.226550011	0.717	0.255	1.05E-232
PNP	0	1.225653589	0.529	0.071	0
HAS1	5.07E-124	1.225197716	0.365	0.1	1.09E-119
NRIP1	7.15E-173	1.22356441	0.665	0.274	1.54E-168
MAP2K3	4.71E-278	1.209112879	0.61	0.159	1.02E-273
METRNL	7.59E-175	1.199350456	0.867	0.524	1.64E-170
PNO1	0	1.198552723	0.648	0.126	0
EIF3J	3.15E-224	1.190947659	0.798	0.354	6.79E-220
FST	3.77E-38	1.189753554	0.554	0.363	8.13E-34
MMP19	2.09E-146	1.163531157	0.49	0.158	4.52E-142
SPHK1	0	1.161883269	0.505	0.055	0
GTPBP4	4.09E-292	1.160106831	0.692	0.2	8.83E-288
INSIG1	6.60E-106	1.136485427	0.545	0.246	1.42E-101
UAP1	9.07E-182	1.135802174	0.917	0.571	1.96E-177
HNRNPAB	7.20E-178	1.132305941	0.832	0.477	1.55E-173
TM4SF1	7.45E-21	1.122636535	0.145	0.064	1.61E-16
MAFF	5.89E-95	1.121643627	0.888	0.614	1.27E-90
SFPQ	3.77E-195	1.11764818	0.951	0.701	8.13E-191
HSPE1	2.27E-107	1.111642136	0.917	0.737	4.91E-103
CD59	1.34E-174	1.105168851	0.932	0.718	2.89E-170
NPM1	1.31E-158	1.104642965	0.969	0.864	2.82E-154
BAZ1A	1.91E-192	1.10414809	0.663	0.248	4.11E-188
S100A16	2.75E-104	1.103271847	0.678	0.396	5.93E-100
NFATC2	5.00E-287	1.100239614	0.515	0.103	1.08E-282
MIR4435-2HG	1.58E-105	1.095343197	0.536	0.245	3.41E-101
SNRPB	1.37E-171	1.094195316	0.828	0.507	2.95E-167
LDHA	4.29E-155	1.092423143	0.942	0.756	9.25E-151

WTAP	4.36E-129	1.085270423	0.764	0.438	9.40E-125
CEBPB	3.39E-127	1.070915704	0.951	0.767	7.31E-123
KLHL21	3.78E-244	1.058946573	0.491	0.11	8.16E-240
HSPA9	9.47E-183	1.057422602	0.789	0.391	2.04E-178
TOP1	4.02E-145	1.056840764	0.848	0.535	8.66E-141
EFHD2	8.26E-226	1.051540015	0.563	0.158	1.78E-221
FEM1C	8.40E-188	1.047284013	0.578	0.192	1.81E-183
FGFR1	8.64E-129	1.040034106	0.921	0.726	1.86E-124
HSPA5	2.89E-94	1.039341078	0.82	0.597	6.23E-90
LDLR	2.05E-216	1.029647061	0.586	0.167	4.42E-212
PIM3	1.02E-113	1.026561275	0.529	0.225	2.19E-109
CRYAB	5.74E-07	1.02399946	0.591	0.574	0.012391179
LRRC59	8.24E-252	1.023110214	0.659	0.203	1.78E-247
RHEB	2.46E-170	1.022741026	0.853	0.541	5.31E-166
AC108134.2	7.66E-12	1.00876071	0.201	0.124	1.65E-07
CDV3	4.23E-151	1.008068204	0.827	0.504	9.12E-147
RND3	2.05E-96	1.007322387	0.786	0.515	4.42E-92
VEGFA	1.54E-62	1.002222379	0.596	0.345	3.32E-58
CHD1	8.09E-141	0.994650312	0.816	0.458	1.74E-136
EIF5A	1.37E-141	0.994633703	0.838	0.555	2.96E-137
EIF1AX	1.10E-129	0.99324565	0.88	0.659	2.38E-125
MAP3K8	3.65E-113	0.988107665	0.6	0.279	7.87E-109
TUBB6	5.07E-157	0.986757509	0.798	0.43	1.09E-152
TAF1D	4.54E-133	0.982535177	0.83	0.54	9.80E-129
NOP16	2.48E-248	0.973707653	0.596	0.16	5.34E-244
VIM	4.43E-217	0.973361207	1	0.997	9.55E-213
PLSCR1	4.93E-119	0.967972455	0.759	0.452	1.06E-114
PDLIM4	3.30E-172	0.967903336	0.654	0.261	7.12E-168
NIFK	2.39E-155	0.962015062	0.716	0.339	5.15E-151
PPA1	2.82E-123	0.960513503	0.823	0.538	6.08E-119
JMJD1C	1.83E-108	0.955960807	0.822	0.582	3.94E-104
NXT1	2.37E-173	0.954376328	0.663	0.265	5.11E-169
CTNNAL1	2.72E-192	0.946558484	0.538	0.159	5.86E-188
PFDN2	5.82E-149	0.946362338	0.822	0.499	1.26E-144
ATP13A3	1.58E-166	0.942327327	0.614	0.225	3.41E-162
KLF6	6.30E-71	0.938933478	0.812	0.647	1.36E-66
SERTAD2	1.89E-142	0.938515068	0.489	0.167	4.08E-138
GSPT1	8.30E-156	0.938050167	0.707	0.329	1.79E-151
YWHAG	6.07E-167	0.928786406	0.716	0.312	1.31E-162
NIP7	3.22E-265	0.927606459	0.578	0.143	6.95E-261
DNAJA1	4.36E-123	0.927220603	0.955	0.782	9.41E-119
TRIB1	5.79E-173	0.926405076	0.544	0.169	1.25E-168
IGF1	9.03E-85	0.92562529	0.743	0.461	1.95E-80
SERBP1	3.03E-136	0.921047058	0.885	0.645	6.53E-132
EIF3A	6.33E-118	0.910967052	0.763	0.45	1.37E-113
RFLNB	4.73E-186	0.907123108	0.402	0.092	1.02E-181
IPO7	1.26E-169	0.906099434	0.662	0.261	2.72E-165
NME1	1.22E-205	0.905923735	0.591	0.184	2.63E-201
DCAF13	1.81E-198	0.903135651	0.594	0.192	3.90E-194
BACH1	1.02E-178	0.903075784	0.545	0.172	2.20E-174
EIF4A1	5.62E-146	0.901461948	0.915	0.649	1.21E-141
JUND	6.82E-143	0.899497826	0.995	0.958	1.47E-138

PTMA	1.02E-197	0.898532253	0.997	0.966	2.19E-193
GPATCH4	1.14E-271	0.897239824	0.491	0.1	2.47E-267
PODN	4.93E-99	0.893694767	0.929	0.785	1.06E-94
RBM8A	9.33E-150	0.892715586	0.87	0.56	2.01E-145
XBP1	1.67E-77	0.892507152	0.682	0.422	3.59E-73
BAG3	8.22E-79	0.890573872	0.719	0.44	1.77E-74
ETS2	8.86E-167	0.885433109	0.48	0.139	1.91E-162
NEU1	8.30E-45	0.885130593	0.538	0.345	1.79E-40
RSL1D1	4.63E-130	0.882408028	0.827	0.534	1.00E-125
ENO1	6.24E-122	0.882150629	0.843	0.566	1.35E-117
PNPLA8	3.63E-122	0.880369655	0.675	0.339	7.83E-118
TMED5	1.05E-113	0.873226637	0.683	0.361	2.27E-109
ZNF593	9.44E-171	0.871121033	0.628	0.235	2.04E-166
DNAJC2	1.27E-182	0.864895249	0.58	0.192	2.74E-178
SERTAD1	4.11E-105	0.863199901	0.904	0.605	8.87E-101
NR4A3	5.75E-71	0.8627868	0.386	0.161	1.24E-66
RGS2	1.22E-60	0.858700735	0.467	0.236	2.64E-56
YBX1	5.98E-148	0.856951788	0.957	0.883	1.29E-143
NOP58	3.02E-130	0.85630089	0.714	0.353	6.52E-126
S100A10	4.43E-110	0.856254022	0.995	0.969	9.56E-106
ERRFI1	2.18E-62	0.853231626	0.744	0.547	4.70E-58
NAA50	2.77E-131	0.842970728	0.63	0.286	5.97E-127
MSC	5.66E-181	0.841711693	0.355	0.075	1.22E-176
TUBB4B	6.09E-94	0.841491651	0.927	0.728	1.31E-89
RRAD	5.29E-17	0.841168045	0.218	0.125	1.14E-12
TXNRD1	7.86E-87	0.84064746	0.5	0.23	1.70E-82
ETF1	2.27E-129	0.840176771	0.726	0.377	4.89E-125
MIR222HG	1.57E-197	0.837943786	0.411	0.09	3.38E-193
EML4	5.62E-160	0.837493064	0.484	0.145	1.21E-155
PHLDA2	8.62E-177	0.825006341	0.336	0.068	1.86E-172
DDX18	3.25E-97	0.821470037	0.757	0.476	7.02E-93
HSPA8	1.38E-87	0.821160261	0.932	0.763	2.98E-83
CCT2	2.76E-123	0.814264054	0.733	0.404	5.95E-119
SRGAP1	3.77E-79	0.812396241	0.555	0.294	8.13E-75
HSPA6	6.06E-07	0.811293021	0.236	0.172	0.013080988
DNTTIP2	1.13E-121	0.809146642	0.667	0.324	2.43E-117
ANK2	1.25E-98	0.806548837	0.709	0.391	2.70E-94
UGP2	1.34E-19	0.806089464	0.628	0.534	2.90E-15
HRH1	5.27E-178	0.803777632	0.373	0.083	1.14E-173
SEC61B	2.63E-118	0.801770606	0.901	0.726	5.68E-114
SNRPD1	8.75E-124	0.800078085	0.742	0.413	1.89E-119
CLIP1	5.76E-98	0.790813898	0.615	0.3	1.24E-93
RRP15	1.39E-164	0.787180306	0.575	0.197	3.00E-160
SLC1A5	9.78E-132	0.78515336	0.594	0.245	2.11E-127
TSC22D2	3.70E-84	0.784658868	0.578	0.297	7.99E-80
GFPT2	4.46E-125	0.784551491	0.87	0.513	9.61E-121
SGMS2	1.52E-169	0.778994681	0.426	0.11	3.29E-165
FAM241A	1.78E-138	0.77740489	0.479	0.156	3.85E-134
SYNCRIP	1.79E-96	0.776554944	0.719	0.421	3.86E-92
PGM3	8.35E-133	0.773728873	0.46	0.153	1.80E-128
COTL1	6.95E-81	0.764025827	0.574	0.306	1.50E-76
BRIX1	3.27E-138	0.762387634	0.594	0.238	7.06E-134

KPNA4	2.04E-103	0.761547172	0.657	0.34	4.40E-99
UBC	2.55E-88	0.760278419	0.994	0.987	5.49E-84
UTP3	5.71E-150	0.759235809	0.496	0.161	1.23E-145
ANKRD28	1.30E-111	0.757992792	0.53	0.211	2.80E-107
MAP1LC3B	3.10E-68	0.757421672	0.823	0.66	6.69E-64
SLC25A5	1.20E-70	0.757412842	0.718	0.487	2.58E-66
ETS1	1.92E-117	0.755222216	0.482	0.177	4.14E-113
AMD1	2.45E-94	0.755206287	0.801	0.53	5.28E-90
MYADM	2.51E-88	0.755073269	0.891	0.698	5.41E-84
3-Mar	2.33E-87	0.75037544	0.533	0.253	5.02E-83
ARL4A	1.03E-122	0.750007931	0.454	0.154	2.22E-118
FGL2	3.40E-56	0.748006184	0.563	0.296	7.34E-52
SRFBP1	2.47E-136	0.746630336	0.528	0.189	5.34E-132
RYBP	7.99E-80	0.746442543	0.54	0.271	1.72E-75
MRTO4	1.97E-165	0.743191307	0.534	0.172	4.24E-161
HLA-E	2.36E-55	0.738879626	0.961	0.914	5.08E-51
GJA1	7.00E-81	0.737065204	0.658	0.364	1.51E-76
EIF1B	7.83E-47	0.734184353	0.799	0.651	1.69E-42
SPRED1	5.45E-100	0.733025821	0.571	0.259	1.18E-95
BLOC1S2	3.20E-124	0.732939209	0.525	0.203	6.90E-120
LINC00324	2.41E-75	0.731783433	0.268	0.082	5.21E-71
KHDRBS3	2.36E-103	0.730777839	0.464	0.177	5.08E-99
FERMT2	1.06E-80	0.730212212	0.783	0.552	2.28E-76
TENT5A	9.91E-29	0.729830065	0.536	0.381	2.14E-24
THBS1	7.51E-76	0.724948745	0.773	0.488	1.62E-71
CEMIP2	8.11E-141	0.719462749	0.414	0.117	1.75E-136
NOP56	1.66E-74	0.715076742	0.634	0.376	3.57E-70
CEBPZ	1.81E-92	0.713935021	0.643	0.341	3.91E-88
ILF2	7.76E-98	0.712050381	0.667	0.37	1.67E-93
ZC3H15	7.04E-90	0.711380355	0.751	0.479	1.52E-85
NFATC1	1.32E-141	0.708047355	0.402	0.11	2.84E-137
IL6R	1.31E-131	0.707904023	0.44	0.135	2.84E-127
NOLC1	5.35E-103	0.70304316	0.595	0.277	1.15E-98
FABP4	0.128708935	0.702445359	0.274	0.254	1
CBLB	2.77E-80	0.701963807	0.685	0.4	5.98E-76
ODC1	1.62E-117	0.700236942	0.447	0.153	3.49E-113
BTG1	1.61E-63	0.700030752	0.898	0.81	3.47E-59
EIF4E	4.08E-102	0.699980747	0.523	0.221	8.79E-98
RPL36AL	1.43E-138	0.697554119	0.972	0.871	3.09E-134
SDC4	8.94E-137	0.69709813	0.409	0.119	1.93E-132
SGK1	8.31E-46	0.696729576	0.491	0.279	1.79E-41
ELOVL5	3.61E-129	0.691556971	0.501	0.176	7.79E-125
HNRNPF	5.58E-90	0.688191354	0.83	0.616	1.20E-85
HSP90AA1	8.19E-68	0.688056262	0.996	0.96	1.77E-63
PAK1IP1	1.88E-121	0.687373917	0.48	0.172	4.06E-117
GRPEL1	4.78E-127	0.682960371	0.511	0.188	1.03E-122
MPHOSPH10	1.05E-96	0.682723093	0.578	0.271	2.27E-92
MT-ND4L	1.48E-100	0.681306343	0.985	0.871	3.20E-96
METAP2	7.10E-82	0.679264251	0.685	0.412	1.53E-77
EIF5B	1.81E-69	0.674687289	0.653	0.39	3.90E-65
SQSTM1	1.16E-35	0.671665926	0.9	0.833	2.50E-31
STIP1	5.52E-104	0.666135231	0.472	0.186	1.19E-99

HNRNPA1	2.57E-99	0.665119797	0.95	0.832	5.54E-95
SMAD7	6.14E-146	0.663023582	0.338	0.079	1.33E-141
RRS1	7.02E-280	0.660482085	0.351	0.048	1.51E-275
HNRNPM	2.10E-75	0.660245312	0.716	0.451	4.53E-71
PTRH2	3.88E-151	0.657664603	0.46	0.136	8.38E-147
STRAP	4.90E-85	0.657497814	0.604	0.322	1.06E-80
RPS6KA3	6.58E-93	0.657209062	0.529	0.234	1.42E-88
PABPC4	1.17E-81	0.657138879	0.664	0.37	2.51E-77
KDM7A	5.87E-92	0.656470156	0.385	0.136	1.27E-87
CCT6A	1.14E-88	0.65621214	0.598	0.304	2.45E-84
PROCR	2.86E-24	0.652864079	0.645	0.486	6.17E-20
TUBB2A	1.95E-45	0.652518611	0.466	0.264	4.20E-41
RFX2	8.05E-138	0.650583896	0.392	0.108	1.74E-133
TRMT10C	9.65E-89	0.649127313	0.58	0.282	2.08E-84
HNRNPA3	1.88E-75	0.646863306	0.813	0.603	4.05E-71
PEA15	1.20E-74	0.645780339	0.57	0.307	2.58E-70
MT-ND5	2.41E-93	0.64351974	0.99	0.936	5.20E-89
PFKFB3	8.83E-92	0.64257466	0.362	0.122	1.91E-87
PITPNB	1.54E-76	0.642485017	0.594	0.314	3.32E-72
S1PR3	1.98E-101	0.641281918	0.328	0.097	4.28E-97
MAPKAPK2	7.84E-116	0.640788764	0.472	0.17	1.69E-111
EBNA1BP2	1.21E-102	0.639458338	0.543	0.238	2.62E-98
CCDC47	6.90E-81	0.638483164	0.659	0.377	1.49E-76
NAA15	8.34E-117	0.635798057	0.475	0.169	1.80E-112
XRN2	5.17E-82	0.635117913	0.653	0.363	1.12E-77
UPP1	8.21E-93	0.633158176	0.341	0.11	1.77E-88
SLC7A5	0	0.632668186	0.263	0.013	0
ZNF800	6.63E-107	0.632253486	0.501	0.197	1.43E-102
MRPL32	2.46E-82	0.62824178	0.663	0.371	5.31E-78
AKIRIN1	1.33E-92	0.626990107	0.637	0.322	2.87E-88
COQ10B	5.10E-73	0.626648323	0.645	0.368	1.10E-68
SERPINE2	3.79E-19	0.625882632	0.164	0.079	8.18E-15
FNDC4	1.36E-72	0.625537739	0.414	0.175	2.93E-68
SET	1.02E-71	0.624821206	0.815	0.604	2.21E-67
PAK2	1.20E-65	0.621410942	0.591	0.332	2.58E-61
MIR22HG	9.19E-72	0.620917307	0.728	0.435	1.98E-67
GNL2	1.15E-134	0.619445565	0.424	0.126	2.49E-130
AKAP12	0.18822776	0.61622869	0.534	0.556	1
EIF3B	3.13E-78	0.611963118	0.511	0.243	6.76E-74
AADAC	3.09E-106	0.611857156	0.346	0.095	6.66E-102
USP12	5.34E-74	0.611698147	0.504	0.242	1.15E-69
IRF1	3.71E-26	0.608205943	0.768	0.61	8.00E-22
SLC25A33	4.39E-103	0.607886131	0.48	0.185	9.47E-99
TFAM	2.52E-73	0.607432882	0.553	0.283	5.43E-69
SNHG15	3.26E-72	0.605078208	0.575	0.302	7.03E-68
OTUD4	2.27E-105	0.603161739	0.392	0.128	4.89E-101
FNIP2	7.84E-72	0.603001551	0.307	0.109	1.69E-67
UTP11	3.29E-100	0.602996372	0.485	0.192	7.10E-96
SUB1	7.20E-79	0.601625116	0.898	0.771	1.55E-74
GLRX	9.23E-25	0.601386171	0.42	0.274	1.99E-20
TOMM40	5.19E-107	0.601132183	0.46	0.172	1.12E-102
DDX5	4.08E-106	0.599841316	0.991	0.95	8.81E-102

PA2G4	4.38E-59	0.597555051	0.732	0.52	9.44E-55
STK40	1.77E-144	0.596408027	0.361	0.09	3.82E-140
EIF2S1	5.15E-83	0.594300555	0.551	0.266	1.11E-78
UBE2N	1.61E-77	0.594027634	0.627	0.346	3.47E-73
ELL	4.28E-140	0.592450962	0.38	0.1	9.23E-136
DKC1	2.07E-102	0.591979143	0.355	0.111	4.47E-98
MFSD2A	0	0.591755033	0.262	0.018	0
EMP3	1.52E-51	0.590962746	0.888	0.742	3.29E-47
ANXA5	8.15E-60	0.590090693	0.905	0.814	1.76E-55
FAP	3.62E-76	0.588397819	0.37	0.139	7.81E-72
BDKRB1	2.29E-45	0.587712549	0.15	0.044	4.95E-41
PPP1R15B	2.50E-49	0.586249243	0.573	0.342	5.39E-45
MSX1	1.96E-59	0.584042858	0.539	0.287	4.23E-55
ATP6V1B2	4.85E-61	0.581685055	0.429	0.207	1.05E-56
AKAP13	3.64E-46	0.578536477	0.726	0.528	7.85E-42
SAMD4A	1.30E-84	0.577841875	0.404	0.152	2.80E-80
GRWD1	1.42E-143	0.576556881	0.351	0.086	3.06E-139
BZW2	5.11E-107	0.575177745	0.47	0.174	1.10E-102
DIMT1	4.00E-84	0.572363905	0.429	0.173	8.62E-80
ARFGAP3	2.48E-77	0.57104727	0.497	0.226	5.35E-73
ABCE1	4.47E-75	0.571023787	0.494	0.231	9.64E-71
FGF7	7.89E-07	0.570201614	0.66	0.668	0.017017709
SLC16A1	2.12E-72	0.570005267	0.495	0.232	4.58E-68
BTG3	1.04E-47	0.569771759	0.588	0.367	2.25E-43
SNRPG	2.96E-69	0.568250108	0.771	0.522	6.39E-65
ZBTB21	1.16E-101	0.565765733	0.466	0.172	2.51E-97
SLC3A2	4.39E-41	0.56429215	0.63	0.443	9.48E-37
AC016831.1	4.05E-89	0.563904019	0.283	0.081	8.73E-85
HSPA1A	5.32E-14	0.561961359	0.887	0.833	1.15E-09
FAM208B	2.34E-81	0.558630886	0.452	0.189	5.04E-77
SPAG9	7.66E-55	0.556962128	0.736	0.507	1.65E-50
SLC20A1	1.98E-67	0.556366081	0.536	0.267	4.27E-63
TWIST1	1.95E-39	0.555816336	0.53	0.314	4.21E-35
RPF2	1.87E-74	0.554496613	0.508	0.242	4.04E-70
PANX1	9.02E-119	0.553467264	0.335	0.09	1.95E-114
SBNO2	4.18E-123	0.55324318	0.323	0.082	9.02E-119
TCP1	6.12E-51	0.552380537	0.69	0.484	1.32E-46
C1QBP	9.01E-56	0.552357184	0.624	0.398	1.94E-51
RIOK1	2.88E-94	0.549948649	0.401	0.142	6.20E-90
CSNK1A1	3.53E-55	0.549618691	0.771	0.578	7.62E-51
CTSC	0.000664513	0.547679869	0.281	0.238	1
AAED1	2.87E-62	0.547564414	0.525	0.277	6.20E-58
MAP4K4	1.50E-44	0.543628023	0.51	0.304	3.23E-40
HSPB8	5.51E-35	0.543512943	0.449	0.271	1.19E-30
WNT9A	5.70E-189	0.54321148	0.234	0.031	1.23E-184
ARPC5L	1.94E-105	0.542174028	0.412	0.141	4.18E-101
PPP2CB	9.16E-67	0.541378454	0.595	0.333	1.98E-62
TNFRSF1A	3.33E-38	0.540945862	0.687	0.503	7.19E-34
KIF21A	1.60E-133	0.539066775	0.256	0.05	3.46E-129
HNRNPA0	1.98E-55	0.537097889	0.737	0.533	4.27E-51
FAM180A	6.88E-52	0.536423201	0.259	0.099	1.48E-47
PSMD11	2.99E-71	0.535804134	0.585	0.314	6.45E-67

FABP5	3.73E-15	0.535136486	0.276	0.173	8.06E-11
MAPRE1	2.20E-61	0.534624605	0.553	0.306	4.74E-57
SRP19	1.56E-66	0.5324244	0.53	0.275	3.36E-62
CCT5	1.22E-63	0.530950333	0.618	0.356	2.63E-59
TSR1	1.43E-133	0.530356066	0.353	0.09	3.08E-129
SBDS	4.26E-52	0.529677747	0.768	0.583	9.19E-48
CASP4	5.31E-48	0.52534042	0.54	0.324	1.14E-43
DHX36	2.41E-48	0.524852536	0.67	0.446	5.20E-44
ARL13B	6.72E-61	0.524214569	0.46	0.225	1.45E-56
HIF1A	1.39E-37	0.524136052	0.352	0.188	3.00E-33
SNRPF	3.91E-57	0.523890594	0.703	0.457	8.44E-53
PUM3	1.69E-64	0.52364485	0.472	0.224	3.65E-60
ZSWIM6	9.80E-83	0.52255368	0.342	0.118	2.11E-78
PDLIM3	1.75E-34	0.522468361	0.308	0.156	3.78E-30
IL4R	8.21E-79	0.5217626	0.35	0.125	1.77E-74
ABCF1	4.16E-55	0.519548221	0.441	0.218	8.97E-51
UFM1	2.44E-51	0.518033452	0.608	0.378	5.27E-47
RTN4	1.18E-52	0.516738168	0.941	0.876	2.55E-48
IMP4	9.29E-75	0.51616587	0.419	0.177	2.00E-70
RPL22L1	8.96E-47	0.515932126	0.727	0.522	1.93E-42
RRP1	5.01E-124	0.514408931	0.335	0.087	1.08E-119
HNRNPK	7.49E-53	0.513845999	0.891	0.758	1.62E-48
YBX3	2.05E-75	0.509784935	0.975	0.901	4.41E-71
CDKN1A	4.74E-46	0.50907786	0.867	0.618	1.02E-41
FILIP1L	1.22E-28	0.509042836	0.698	0.523	2.62E-24
PPRC1	3.99E-101	0.507918668	0.351	0.108	8.60E-97
TUBA1A	8.71E-05	0.507762204	0.773	0.768	1
PLEKHM2	1.64E-64	0.506476614	0.424	0.19	3.55E-60
PLK3	1.36E-54	0.506335889	0.393	0.183	2.92E-50
SSB	7.91E-48	0.503412885	0.762	0.58	1.71E-43
POLR1C	2.45E-130	0.502761422	0.338	0.085	5.28E-126
PLA2G16	3.30E-45	0.500775625	0.559	0.346	7.11E-41
BCCIP	4.00E-66	0.498700987	0.351	0.14	8.63E-62
DDX3X	1.37E-48	0.49834704	0.85	0.663	2.96E-44
DNAJB9	2.03E-28	0.498276984	0.353	0.207	4.37E-24
H2AFZ	3.97E-33	0.496315948	0.823	0.683	8.57E-29
RELB	1.73E-105	0.495880233	0.308	0.084	3.73E-101
ATG101	1.24E-56	0.495341386	0.543	0.3	2.69E-52
MTHFD2	2.76E-74	0.493546255	0.396	0.155	5.96E-70
PPTC7	3.64E-120	0.493078725	0.36	0.099	7.84E-116
CRY1	2.65E-96	0.492104653	0.375	0.125	5.71E-92
PUF60	1.07E-52	0.489683231	0.535	0.312	2.30E-48
ERF	1.24E-80	0.488620151	0.326	0.109	2.67E-76
FHL1	6.66E-07	0.488296826	0.706	0.68	0.014367133
FABP3	8.77E-13	0.487686551	0.112	0.054	1.89E-08
HNRNPU	2.70E-59	0.486751391	0.89	0.707	5.82E-55
ATF4	3.81E-34	0.485733292	0.769	0.628	8.22E-30
LIF	3.23E-126	0.48542748	0.132	0.014	6.96E-122
PSMG1	1.41E-78	0.484825982	0.367	0.135	3.05E-74
COPS2	4.21E-48	0.483001899	0.602	0.387	9.08E-44
SACS	8.65E-57	0.482937337	0.325	0.129	1.86E-52
NOC3L	4.46E-47	0.481172246	0.377	0.186	9.63E-43

POMP	6.51E-49	0.479971063	0.777	0.604	1.40E-44
BLOC1S6	5.70E-40	0.479582736	0.548	0.35	1.23E-35
RLF	1.18E-50	0.478358627	0.427	0.215	2.54E-46
LIMS1	2.04E-41	0.476285768	0.615	0.419	4.41E-37
USP16	2.72E-43	0.476272331	0.588	0.364	5.87E-39
PMM2	8.26E-117	0.476128388	0.337	0.091	1.78E-112
FUBP1	1.52E-76	0.475729623	0.42	0.169	3.27E-72
PLOD2	5.33E-45	0.47523192	0.33	0.152	1.15E-40
PSMD12	7.30E-51	0.475192579	0.513	0.292	1.57E-46
MAP1B	1.95E-15	0.474951588	0.667	0.567	4.20E-11
EIF5	1.18E-45	0.47475089	0.901	0.798	2.54E-41
RUFY3	1.07E-43	0.474413358	0.499	0.285	2.31E-39
UBE2S	8.96E-36	0.472436569	0.677	0.467	1.93E-31
GADD45A	2.14E-12	0.472365879	0.455	0.356	4.62E-08
ZBTB43	1.03E-59	0.4716144	0.335	0.136	2.22E-55
WDR74	3.46E-63	0.471163282	0.386	0.166	7.47E-59
COL27A1	1.17E-61	0.470074609	0.167	0.042	2.52E-57
FAM126A	2.22E-41	0.469393641	0.49	0.288	4.78E-37
TUSC1	1.61E-27	0.468929049	0.422	0.272	3.48E-23
EIF1	3.88E-110	0.468842876	1	0.996	8.36E-106
BYSL	1.44E-165	0.468302861	0.288	0.052	3.10E-161
NTRK2	1.27E-67	0.467694329	0.747	0.402	2.74E-63
DNAJA4	8.52E-41	0.46760193	0.287	0.125	1.84E-36
CCT3	6.89E-42	0.467393274	0.575	0.374	1.49E-37
PDE12	4.49E-84	0.4666511	0.325	0.105	9.69E-80
PPP2CA	1.68E-60	0.465720789	0.501	0.254	3.62E-56
CCT4	1.71E-42	0.463657848	0.675	0.481	3.69E-38
GNG5	1.44E-42	0.46364994	0.792	0.601	3.10E-38
PPAN	1.68E-152	0.462560466	0.291	0.057	3.62E-148
DCLK1	3.09E-21	0.459685956	0.624	0.493	6.67E-17
ELK3	3.94E-47	0.459372115	0.404	0.204	8.49E-43
YTHDF2	2.74E-39	0.45596482	0.564	0.36	5.90E-35
TIMM17A	2.15E-77	0.455159341	0.391	0.151	4.63E-73
MORF4L2	2.57E-38	0.454713913	0.727	0.548	5.55E-34
DNAJB6	7.34E-34	0.454107223	0.704	0.546	1.58E-29
NFKBIA	3.51E-14	0.45388958	0.843	0.804	7.58E-10
MT-ND4	2.86E-69	0.453709762	1	0.993	6.17E-65
VAPA	5.58E-40	0.453253729	0.776	0.611	1.20E-35
HNRNPH3	3.25E-37	0.45282253	0.747	0.585	7.02E-33
PRPF40A	7.67E-42	0.452690409	0.595	0.386	1.66E-37
RSL24D1	2.73E-42	0.451804702	0.802	0.639	5.90E-38
ELOC	8.77E-32	0.451111733	0.731	0.567	1.89E-27
AKR1C2	2.83E-24	0.450579509	0.419	0.268	6.10E-20
MT-ND3	1.91E-70	0.45022222	0.997	0.993	4.11E-66
STRN3	5.03E-45	0.449196168	0.422	0.224	1.08E-40
SPRY1	0.189567086	0.448143711	0.561	0.594	1
PDLIM5	1.25E-45	0.447970851	0.442	0.236	2.70E-41
CREM	1.02E-21	0.446687094	0.337	0.207	2.21E-17
H3F3B	4.29E-58	0.445286312	0.991	0.983	9.25E-54
AGFG1	1.30E-53	0.444911114	0.427	0.208	2.81E-49
IDI1	8.87E-40	0.442902729	0.538	0.326	1.91E-35
APOL4	3.10E-68	0.440983915	0.291	0.099	6.69E-64

KLF16	1.19E-92	0.440469692	0.273	0.074	2.57E-88
IVNS1ABP	7.91E-43	0.439041835	0.442	0.237	1.71E-38
PABPC1	4.58E-38	0.438001163	0.878	0.754	9.88E-34
CCDC59	1.77E-43	0.437474818	0.69	0.468	3.82E-39
UBA2	2.97E-46	0.436304696	0.492	0.279	6.41E-42
GATAD2A	9.43E-73	0.436239812	0.313	0.109	2.03E-68
ZNF207	3.41E-39	0.436205358	0.558	0.357	7.37E-35
FLNC	1.40E-20	0.436159	0.206	0.106	3.02E-16
CHCHD2	1.50E-60	0.434584958	0.955	0.892	3.24E-56
DNAJA2	6.11E-38	0.434312315	0.524	0.328	1.32E-33
HNRNPR	2.98E-39	0.433321318	0.655	0.469	6.43E-35
CLINT1	6.57E-43	0.432279544	0.534	0.314	1.42E-38
SDC2	3.83E-31	0.430792299	0.783	0.646	8.25E-27
RAB21	8.90E-33	0.430628816	0.554	0.373	1.92E-28
PRRX1	7.73E-31	0.430322744	0.793	0.655	1.67E-26
RAB7A	3.45E-24	0.429545812	0.665	0.519	7.44E-20
ILF3	2.49E-41	0.429263741	0.499	0.285	5.37E-37
AK6	2.66E-40	0.42924524	0.513	0.306	5.75E-36
GALNT2	3.25E-55	0.427835499	0.41	0.191	7.02E-51
DDX3Y	1.27E-28	0.426348224	0.685	0.504	2.74E-24
SRM	1.18E-33	0.426260229	0.503	0.321	2.54E-29
TOMM5	5.71E-61	0.425683702	0.396	0.171	1.23E-56
SLC7A1	1.54E-167	0.42448828	0.257	0.041	3.33E-163
NDUFAF4	3.15E-66	0.422724568	0.378	0.155	6.79E-62
EIF4G1	9.13E-43	0.422468738	0.455	0.249	1.97E-38
RNPS1	1.48E-38	0.418851589	0.558	0.353	3.19E-34
MAD2L2	2.11E-56	0.41817557	0.332	0.138	4.56E-52
TCERG1	2.56E-53	0.417685466	0.371	0.165	5.53E-49
RNMT	3.87E-32	0.415771182	0.544	0.358	8.35E-28
DENND4A	4.93E-44	0.415575998	0.36	0.174	1.06E-39
EXOSC6	1.63E-51	0.41481511	0.44	0.218	3.51E-47
FOXP1	5.69E-24	0.414093943	0.55	0.397	1.23E-19
SEC61G	1.19E-38	0.413123386	0.806	0.644	2.57E-34
TMEM100	2.31E-30	0.413042392	0.266	0.13	4.99E-26
YES1	2.68E-43	0.412409819	0.383	0.195	5.79E-39
PSMC6	5.66E-37	0.411707709	0.545	0.351	1.22E-32
CHMP4B	1.57E-42	0.411670286	0.482	0.275	3.38E-38
IER5	1.62E-29	0.410821619	0.638	0.462	3.50E-25
MEG3	2.53E-34	0.410766891	0.833	0.678	5.46E-30
PCGF5	1.62E-49	0.410535009	0.395	0.19	3.50E-45
CMC2	6.66E-48	0.410464428	0.409	0.203	1.44E-43
SMG1	1.69E-41	0.409828494	0.506	0.29	3.64E-37
QTRT2	2.78E-102	0.409660217	0.273	0.069	6.00E-98
PBDC1	2.54E-32	0.407227825	0.441	0.266	5.48E-28
NOL10	5.13E-70	0.406358485	0.299	0.102	1.11E-65
IFRD2	4.61E-75	0.405732004	0.327	0.115	9.95E-71
TIMM8A	1.66E-128	0.40549945	0.243	0.047	3.58E-124
PSME4	1.06E-63	0.405310531	0.318	0.119	2.29E-59
FGF2	4.29E-17	0.404700077	0.363	0.25	9.25E-13
CYP26B1	1.07E-29	0.404561006	0.291	0.146	2.31E-25
GABPB1	3.90E-101	0.404530552	0.266	0.065	8.41E-97
HNRNPC	2.49E-37	0.404201113	0.799	0.632	5.36E-33

DOK5	8.03E-64	0.402175499	0.209	0.061	1.73E-59
TSSC4	1.35E-50	0.402042252	0.409	0.2	2.91E-46
KIF5B	4.42E-34	0.401954681	0.627	0.436	9.54E-30
DDX39A	1.67E-58	0.401934204	0.353	0.148	3.60E-54
CAMTA1	1.10E-33	0.401407041	0.531	0.347	2.37E-29
MT-ND2	2.31E-62	0.40111615	1	0.992	4.99E-58
SRSF10	7.45E-32	0.399980135	0.669	0.49	1.61E-27
DEGS1	3.65E-32	0.399728607	0.526	0.344	7.88E-28
G3BP1	1.16E-36	0.398316105	0.583	0.387	2.51E-32
NAA20	1.02E-36	0.397939776	0.494	0.304	2.19E-32
EPAS1	9.98E-29	0.397610888	0.34	0.191	2.15E-24
RRBP1	9.23E-28	0.397052077	0.793	0.642	1.99E-23
ST3GAL1	3.94E-44	0.396447597	0.336	0.156	8.49E-40
POLR3D	5.98E-76	0.395966063	0.292	0.094	1.29E-71
SEPHS2	5.72E-19	0.395938192	0.373	0.251	1.23E-14
NANS	1.19E-38	0.395887985	0.398	0.214	2.57E-34
DHX15	6.40E-36	0.39572033	0.421	0.238	1.38E-31
VCL	1.24E-25	0.395656266	0.49	0.326	2.68E-21
COL14A1	0.02915827	0.395417288	0.492	0.491	1
CRIP1	2.02E-07	0.395350217	0.515	0.443	0.004367423
HNRNPDL	1.71E-29	0.394460641	0.89	0.765	3.69E-25
PRELID1	1.30E-28	0.393704246	0.657	0.493	2.80E-24
RCL1	3.13E-104	0.393558358	0.291	0.075	6.74E-100
MFHAS1	2.71E-78	0.393067108	0.261	0.075	5.85E-74
MRPL47	5.58E-50	0.392571174	0.447	0.229	1.20E-45
MAGOH	6.04E-35	0.391889493	0.615	0.423	1.30E-30
TAF7	3.60E-29	0.390938525	0.614	0.445	7.77E-25
TXLNG	5.26E-33	0.390644525	0.469	0.281	1.13E-28
POLR1E	4.34E-49	0.390028453	0.308	0.131	9.37E-45
SERPINB1	6.79E-18	0.389753796	0.593	0.448	1.46E-13
TXLNA	1.85E-42	0.389134973	0.356	0.171	4.00E-38
HSPA1B	2.64E-09	0.389053163	0.756	0.66	5.69E-05
CHORDC1	1.04E-22	0.388872026	0.451	0.309	2.24E-18
BTBD7	2.57E-21	0.388046789	0.42	0.27	5.55E-17
AEBP1	2.18E-22	0.387871028	0.826	0.715	4.70E-18
TWISTNB	3.55E-36	0.387593868	0.375	0.201	7.67E-32
HIVEP3	1.52E-55	0.387060656	0.165	0.044	3.28E-51
PSMD14	1.03E-45	0.386916802	0.37	0.179	2.23E-41
HPRT1	8.32E-96	0.386816655	0.268	0.07	1.79E-91
ZNF787	1.36E-61	0.386481326	0.287	0.103	2.94E-57
ACBD3	7.08E-32	0.386337117	0.485	0.304	1.53E-27
IGFBP5	2.69E-32	0.386266285	0.969	0.9	5.81E-28
S100A11	7.97E-40	0.38546239	0.969	0.929	1.72E-35
GGCT	1.21E-38	0.384796699	0.365	0.187	2.62E-34
SEH1L	2.08E-72	0.384521845	0.276	0.087	4.48E-68
TAF4B	2.47E-63	0.384351872	0.263	0.086	5.32E-59
TAF9	1.27E-35	0.383973755	0.419	0.234	2.75E-31
RBM28	2.97E-50	0.383948106	0.347	0.152	6.41E-46
CEBPG	3.04E-46	0.383008724	0.342	0.156	6.56E-42
GLS	1.03E-21	0.382569792	0.531	0.384	2.22E-17
SERP1	1.23E-26	0.382243261	0.733	0.58	2.65E-22
NDUFA6	6.83E-28	0.382153736	0.561	0.387	1.47E-23

DUSP14	3.81E-43	0.382010523	0.362	0.177	8.22E-39
WISP2	4.05E-07	0.381650906	0.759	0.667	0.008739964
GABARAPL1	6.39E-25	0.380903112	0.642	0.475	1.38E-20
MAP1A	5.37E-24	0.380646683	0.372	0.225	1.16E-19
SLC9B2	3.47E-48	0.380393539	0.299	0.125	7.48E-44
BATF3	1.04E-83	0.380185652	0.185	0.04	2.24E-79
IMPAD1	2.97E-45	0.379941377	0.42	0.215	6.41E-41
CLTB	1.79E-29	0.379820803	0.679	0.507	3.86E-25
UBB	9.50E-39	0.379728374	0.956	0.934	2.05E-34
SIK3	3.80E-55	0.379710404	0.32	0.13	8.20E-51
POLE3	1.96E-42	0.379654621	0.355	0.172	4.22E-38
MTHFD2L	1.24E-116	0.378522133	0.254	0.054	2.67E-112
CUL3	1.09E-39	0.37845174	0.481	0.276	2.36E-35
ZPR1	2.01E-55	0.378116634	0.312	0.126	4.33E-51
EWSR1	9.89E-39	0.377606629	0.519	0.306	2.13E-34
NFKB1	5.26E-19	0.377330687	0.371	0.245	1.13E-14
URB2	1.49E-151	0.377157279	0.201	0.028	3.20E-147
TMEM165	1.27E-30	0.376482048	0.556	0.372	2.74E-26
DYRK3	5.54E-95	0.376477402	0.241	0.058	1.20E-90
PRRC2C	4.48E-29	0.374157263	0.783	0.635	9.66E-25
ADAM17	8.95E-32	0.37351512	0.42	0.243	1.93E-27
CD9	1.61E-27	0.372670426	0.62	0.414	3.47E-23
RRP12	2.38E-142	0.37106211	0.192	0.027	5.13E-138
FAM57A	2.94E-61	0.370766221	0.193	0.054	6.35E-57
NUP153	1.04E-43	0.369109238	0.414	0.212	2.23E-39
YRDC	1.02E-69	0.368799187	0.298	0.103	2.21E-65
PTGFR	1.34E-35	0.368687852	0.355	0.178	2.88E-31
SLTM	7.70E-29	0.368312702	0.55	0.367	1.66E-24
CDK17	1.68E-45	0.368292897	0.264	0.106	3.62E-41
HK1	1.25E-63	0.368066626	0.269	0.091	2.69E-59
ZNFX1	1.41E-35	0.367257617	0.32	0.156	3.05E-31
SURF4	1.95E-27	0.366520181	0.535	0.358	4.20E-23
LHFPL2	1.10E-37	0.366271239	0.237	0.099	2.37E-33
ITGA5	8.41E-21	0.363233565	0.323	0.195	1.81E-16
IARS	2.77E-56	0.363140835	0.298	0.114	5.97E-52
COPS3	8.70E-38	0.36310153	0.378	0.201	1.88E-33
SLIRP	4.01E-30	0.363030218	0.619	0.437	8.64E-26
TAF13	1.08E-70	0.362496304	0.296	0.099	2.32E-66
KRR1	1.65E-32	0.361779919	0.586	0.394	3.56E-28
ARHGAP5	2.42E-18	0.361693703	0.407	0.279	5.22E-14
PPIF	1.06E-33	0.361405141	0.274	0.131	2.30E-29
LARP6	3.67E-15	0.361256916	0.57	0.459	7.92E-11
RAB1A	1.47E-26	0.360841147	0.595	0.431	3.17E-22
PSMC4	4.89E-34	0.360829147	0.429	0.251	1.05E-29
PAICS	6.07E-55	0.360666025	0.286	0.109	1.31E-50
DOT1L	2.42E-136	0.360621872	0.207	0.033	5.22E-132
MID1IP1	6.85E-17	0.359604436	0.296	0.183	1.48E-12
DUSP6	3.10E-23	0.358764219	0.187	0.086	6.69E-19
LPL	3.42E-36	0.358698405	0.201	0.076	7.38E-32
SPART	4.30E-23	0.358408205	0.6	0.445	9.28E-19
IFT57	1.15E-29	0.358210121	0.514	0.339	2.49E-25
PSMA3	1.01E-31	0.357076695	0.61	0.43	2.17E-27

BZW1	2.44E-28	0.356466302	0.812	0.675	5.26E-24
DHX33	3.26E-67	0.356300255	0.227	0.066	7.03E-63
TNC	9.57E-41	0.35610319	0.104	0.025	2.06E-36
MAPK6	2.19E-41	0.356036531	0.368	0.181	4.73E-37
UCHL3	3.22E-46	0.355333088	0.301	0.13	6.94E-42
GLRX3	4.03E-38	0.355101347	0.434	0.241	8.70E-34
GLA	1.38E-12	0.354954371	0.262	0.17	2.97E-08
IGF2BP2	3.46E-60	0.354645871	0.259	0.086	7.46E-56
NOP14	3.58E-54	0.354441093	0.237	0.081	7.73E-50
RASSF8-AS1	8.96E-45	0.353391797	0.332	0.149	1.93E-40
DDX24	1.25E-24	0.352865961	0.757	0.63	2.69E-20
CHI3L2	3.88E-57	0.352480223	0.137	0.031	8.37E-53
ARID5A	3.53E-38	0.352178786	0.352	0.173	7.60E-34
ESF1	1.34E-23	0.351906096	0.462	0.298	2.89E-19
PCBP1	1.93E-22	0.35032381	0.734	0.602	4.16E-18
CRNDE	6.16E-58	0.3500627	0.404	0.167	1.33E-53
MSANTD3	3.33E-50	0.34999596	0.249	0.092	7.19E-46
ADNP2	1.16E-50	0.349584575	0.256	0.094	2.50E-46
RPL7L1	7.34E-23	0.349170437	0.533	0.386	1.58E-18
SNRPE	1.62E-27	0.348298056	0.68	0.511	3.50E-23
ID4	1.51E-12	0.348252562	0.325	0.224	3.26E-08
DUSP5	5.37E-35	0.346891697	0.34	0.173	1.16E-30
ADRM1	1.30E-26	0.34637651	0.451	0.293	2.79E-22
TMEM126A	5.31E-35	0.346108456	0.387	0.213	1.14E-30
PNRC2	3.91E-20	0.344404805	0.638	0.509	8.44E-16
TFG	5.73E-26	0.343979314	0.499	0.337	1.24E-21
USP7	6.08E-32	0.343425521	0.41	0.233	1.31E-27
UBAP1	1.09E-35	0.343188431	0.371	0.195	2.35E-31
MIDN	3.14E-22	0.342917962	0.723	0.526	6.77E-18
LRRC32	5.47E-47	0.34256975	0.214	0.075	1.18E-42
NCBP2	3.07E-34	0.342499298	0.388	0.213	6.62E-30
RBM25	5.50E-25	0.342014802	0.728	0.589	1.19E-20
RELT	5.33E-211	0.341696603	0.164	0.013	1.15E-206
ASNSD1	3.99E-25	0.34042066	0.426	0.266	8.62E-21
ZNF674-AS1	1.54E-126	0.33912731	0.159	0.021	3.32E-122
GAR1	3.51E-46	0.337530287	0.311	0.135	7.57E-42
OGN	1.02E-10	0.337281197	0.602	0.513	2.21E-06
IRF7	2.56E-37	0.335828314	0.257	0.112	5.53E-33
TP53BP2	1.12E-35	0.33547957	0.296	0.139	2.42E-31
LARP1B	7.32E-40	0.33499592	0.269	0.115	1.58E-35
TMEM167A	1.87E-26	0.334863765	0.477	0.308	4.04E-22
ATP1A1	1.40E-26	0.334413779	0.622	0.437	3.03E-22
PLEKHO1	2.35E-31	0.334281585	0.249	0.116	5.07E-27
REEP5	2.57E-19	0.333632723	0.741	0.624	5.55E-15
DPH3	2.27E-30	0.333176275	0.365	0.201	4.89E-26
CXXC5	3.48E-27	0.333108736	0.305	0.161	7.51E-23
IFITM1	2.26E-15	0.332642554	0.62	0.497	4.87E-11
SON	6.13E-29	0.332238536	0.939	0.862	1.32E-24
TXNL1	3.16E-28	0.332063016	0.584	0.406	6.82E-24
ASXL1	2.94E-25	0.331749721	0.441	0.279	6.34E-21
SRPRB	2.98E-37	0.330103843	0.322	0.156	6.43E-33
RAB27A	5.78E-75	0.329144187	0.211	0.054	1.25E-70

PSMD1	2.97E-32	0.328866575	0.386	0.214	6.41E-28
EEF1E1	1.19E-45	0.328252456	0.301	0.129	2.57E-41
CWC25	6.71E-28	0.328071186	0.385	0.226	1.45E-23
PARP14	2.05E-18	0.327882737	0.316	0.195	4.42E-14
WARS	8.43E-33	0.32767357	0.19	0.076	1.82E-28
UGCG	1.29E-19	0.325388642	0.454	0.31	2.78E-15
UBE2D1	4.63E-26	0.325116921	0.376	0.226	1.00E-21
IPO5	4.56E-25	0.325004211	0.47	0.308	9.84E-21
PRPF4B	9.36E-26	0.324328537	0.62	0.437	2.02E-21
NASP	8.32E-27	0.322632495	0.687	0.516	1.80E-22
DNAJC3	1.80E-20	0.321635916	0.525	0.371	3.87E-16
NPIPB5	1.88E-42	0.320260539	0.262	0.105	4.05E-38
UBE2A	1.80E-23	0.320054365	0.506	0.351	3.88E-19
PLIN3	1.42E-12	0.320035904	0.375	0.274	3.06E-08
SERPINB8	1.54E-83	0.319774187	0.208	0.049	3.33E-79
EEF1B2	1.12E-24	0.319607337	0.945	0.902	2.42E-20
ACTN1	1.89E-30	0.31906735	0.198	0.083	4.07E-26
FKBP4	3.46E-24	0.318855063	0.289	0.161	7.47E-20
HIVEP2	5.09E-35	0.318587641	0.286	0.133	1.10E-30
LRRFIP2	7.71E-28	0.318412887	0.351	0.199	1.66E-23
UTP4	3.20E-62	0.318160678	0.239	0.075	6.91E-58
EIF6	1.59E-21	0.318158999	0.509	0.356	3.44E-17
ALYREF	1.57E-30	0.317465114	0.341	0.185	3.40E-26
ACADVL	1.46E-17	0.317183694	0.603	0.464	3.14E-13
AHSA1	5.15E-19	0.316594353	0.459	0.329	1.11E-14
PHLDB2	3.84E-15	0.314673689	0.289	0.183	8.29E-11
MT-ATP8	6.61E-27	0.313841227	0.605	0.407	1.43E-22
REL	2.68E-19	0.313784265	0.391	0.258	5.79E-15
KPNA2	3.30E-24	0.313399516	0.308	0.171	7.12E-20
TMEM263	2.91E-27	0.313327897	0.464	0.294	6.29E-23
MEG8	4.97E-28	0.312567109	0.378	0.209	1.07E-23
CCDC86	2.43E-88	0.312412571	0.209	0.048	5.23E-84
FBL	6.66E-22	0.312237717	0.481	0.33	1.44E-17
Z97200.1	5.50E-89	0.311312298	0.172	0.033	1.19E-84
SLC19A2	2.00E-36	0.311066255	0.506	0.287	4.30E-32
ASCC3	1.35E-39	0.310483316	0.301	0.135	2.90E-35
DENND5A	7.38E-34	0.310374227	0.32	0.16	1.59E-29
YWHAE	2.81E-20	0.31023072	0.808	0.688	6.06E-16
PSMD7	1.00E-23	0.309635965	0.521	0.357	2.16E-19
NFKBIB	8.70E-50	0.308981011	0.244	0.09	1.88E-45
FAM177A1	3.52E-23	0.308161896	0.419	0.27	7.60E-19
FAM210A	4.00E-66	0.307462918	0.239	0.073	8.63E-62
CXCL14	4.22E-30	0.307437996	0.924	0.735	9.11E-26
APOL6	2.36E-07	0.307119055	0.39	0.319	0.005101398
SNRPB2	1.94E-18	0.306780554	0.618	0.486	4.19E-14
CYB5D1	3.44E-47	0.306714756	0.203	0.068	7.42E-43
SAP30BP	3.62E-26	0.30651014	0.407	0.248	7.82E-22
CTGF	0.075780795	0.306500747	0.492	0.471	1
BCL7B	2.25E-35	0.306458872	0.385	0.203	4.85E-31
PTBP1	1.19E-20	0.305737421	0.485	0.339	2.56E-16
SPTSSA	1.29E-22	0.304780354	0.439	0.288	2.79E-18
UBE2D3	5.85E-17	0.304661887	0.794	0.71	1.26E-12

TRMT6	3.72E-41	0.30453334	0.251	0.101	8.03E-37
LLPH	5.22E-30	0.304278398	0.361	0.199	1.13E-25
HMGCS1	9.41E-28	0.303474835	0.301	0.159	2.03E-23
PNN	1.51E-19	0.303363207	0.59	0.439	3.26E-15
SNU13	2.14E-21	0.303043288	0.731	0.579	4.62E-17
WDR1	4.01E-26	0.302941443	0.352	0.205	8.65E-22
UTP6	5.90E-30	0.302686391	0.35	0.191	1.27E-25
GABPB1-AS1	3.35E-25	0.302484514	0.347	0.198	7.22E-21
TRAF3IP2	3.79E-56	0.302371012	0.228	0.074	8.18E-52
NAF1	3.87E-39	0.302075976	0.276	0.12	8.35E-35
KCMF1	4.51E-29	0.301084962	0.383	0.219	9.73E-25
GADD45B	0.007274152	0.301059225	0.863	0.809	1
STX5	3.00E-27	0.300115759	0.372	0.22	6.48E-23
STAU1	7.43E-22	0.299806643	0.538	0.383	1.60E-17
BUD23	2.62E-31	0.299388313	0.425	0.246	5.64E-27
PTS	8.42E-24	0.299127025	0.38	0.229	1.82E-19
TRMT61A	7.39E-51	0.299039329	0.221	0.075	1.60E-46
BAIAP2	5.47E-49	0.298480498	0.203	0.067	1.18E-44
RABGEF1	5.51E-62	0.298023758	0.206	0.059	1.19E-57
SLC36A4	7.83E-53	0.297756555	0.223	0.074	1.69E-48
SRSF5	1.04E-19	0.297269853	0.92	0.869	2.23E-15
SRP72	2.66E-19	0.296896858	0.519	0.38	5.74E-15
HEATR1	1.41E-42	0.296489973	0.243	0.094	3.05E-38
PMEPA1	1.12E-10	0.296184651	0.264	0.181	2.42E-06
PSMC1	5.70E-21	0.296040345	0.509	0.363	1.23E-16
NSUN2	1.73E-50	0.295889656	0.222	0.075	3.74E-46
KPNB1	1.52E-17	0.29498341	0.529	0.392	3.28E-13
RASSF8	2.79E-17	0.2945178	0.414	0.283	6.02E-13
SYNJ2	1.51E-87	0.293167656	0.162	0.03	3.26E-83
FAM133B	9.09E-19	0.292597733	0.665	0.529	1.96E-14
GPC1	2.75E-35	0.292270451	0.216	0.087	5.92E-31
EIF2A	2.01E-24	0.2919051	0.526	0.356	4.34E-20
AEN	4.98E-74	0.291614528	0.174	0.039	1.07E-69
WNT2	4.49E-28	0.291418592	0.1	0.03	9.69E-24
RNF149	8.68E-24	0.291102086	0.375	0.228	1.87E-19
C9orf47	1.54E-75	0.290869249	0.122	0.02	3.33E-71
KCNE4	6.04E-09	0.290572433	0.118	0.066	0.000130264
PNPT1	3.49E-38	0.290357125	0.277	0.122	7.53E-34
GBP1	4.25E-08	0.290086244	0.248	0.18	0.000917378
C16orf91	4.79E-48	0.289309626	0.254	0.096	1.03E-43
HSPA4	5.14E-24	0.289248857	0.325	0.188	1.11E-19
GCH1	2.81E-65	0.288937793	0.179	0.045	6.06E-61
ACTN4	4.16E-16	0.288193752	0.406	0.28	8.97E-12
SAP18	3.76E-24	0.287408768	0.89	0.789	8.11E-20
TOMM22	2.08E-20	0.287360918	0.424	0.285	4.49E-16
RRP7A	2.49E-33	0.286898036	0.298	0.146	5.36E-29
GPAT3	3.34E-93	0.286849891	0.129	0.019	7.21E-89
PKNOX1	2.03E-29	0.286616218	0.271	0.133	4.39E-25
NUDT4	1.55E-21	0.286397684	0.465	0.309	3.34E-17
STAT3	1.08E-16	0.28636984	0.704	0.557	2.32E-12
PEAK1	2.67E-44	0.285949631	0.247	0.094	5.76E-40
GART	1.33E-34	0.285772553	0.234	0.1	2.86E-30

ITPKC	5.34E-31	0.285110921	0.294	0.141	1.15E-26
PSMB3	1.17E-18	0.283842304	0.658	0.509	2.53E-14
DYNC1LI1	2.71E-20	0.283456103	0.425	0.282	5.86E-16
SUN2	5.05E-21	0.283088366	0.38	0.235	1.09E-16
SRSF2	1.33E-20	0.282661893	0.799	0.658	2.86E-16
ZNF622	8.76E-25	0.282146118	0.335	0.193	1.89E-20
NDUFAF8	6.82E-19	0.282094542	0.477	0.34	1.47E-14
BCAR1	4.95E-48	0.281157904	0.202	0.067	1.07E-43
EIF4H	3.61E-21	0.280761918	0.664	0.519	7.79E-17
TARS	2.23E-29	0.279253052	0.328	0.172	4.81E-25
ATP2A2	2.68E-23	0.279028006	0.412	0.257	5.77E-19
MAFG	1.47E-17	0.278506963	0.366	0.24	3.16E-13
KIF1B	2.15E-19	0.278483924	0.382	0.246	4.63E-15
EN1	1.20E-24	0.276768119	0.168	0.072	2.59E-20
THAP2	2.78E-17	0.276659942	0.208	0.113	5.99E-13
LGALSL	2.41E-10	0.276449863	0.223	0.145	5.21E-06
SAR1B	2.31E-25	0.276273384	0.372	0.219	4.99E-21
KLF10	2.90E-12	0.276011838	0.637	0.505	6.25E-08
NOM1	1.14E-47	0.275748086	0.222	0.077	2.45E-43
CAPZA2	2.30E-19	0.275585088	0.543	0.391	4.95E-15
ADGRE5	3.56E-32	0.275180626	0.228	0.099	7.68E-28
DDX10	8.73E-45	0.274609072	0.232	0.085	1.88E-40
CALM2	1.67E-16	0.274107488	0.916	0.868	3.61E-12
ABCF2.1	1.40E-51	0.273638341	0.211	0.069	3.03E-47
CNIH1	5.83E-17	0.273129652	0.451	0.32	1.26E-12
SDE2	3.67E-49	0.273091961	0.232	0.081	7.91E-45
B4GALT5	7.65E-24	0.272267133	0.318	0.179	1.65E-19
FGF18	3.06E-15	0.272239323	0.194	0.108	6.60E-11
AHI1	3.67E-16	0.272235127	0.543	0.402	7.91E-12
UTP20	1.93E-35	0.271941933	0.221	0.09	4.16E-31
ERN1	2.96E-21	0.271666996	0.227	0.118	6.39E-17
ETV3	4.73E-31	0.271504408	0.229	0.102	1.02E-26
SAR1A	3.48E-17	0.271034802	0.54	0.4	7.51E-13
NUP98	4.32E-25	0.270982246	0.328	0.183	9.32E-21
USP10	4.66E-33	0.270835925	0.286	0.136	1.01E-28
SELENOS	8.02E-17	0.270246885	0.675	0.545	1.73E-12
BAG1	4.83E-15	0.270121624	0.525	0.408	1.04E-10
SF1	9.52E-22	0.270067754	0.657	0.487	2.05E-17
UBE2G2	2.83E-18	0.270032086	0.357	0.228	6.11E-14
CPSF6	1.06E-26	0.269814039	0.313	0.169	2.28E-22
SNW1	4.19E-15	0.269734596	0.495	0.372	9.05E-11
PRMT1	8.33E-15	0.269541919	0.546	0.422	1.80E-10
GOLGA4	1.22E-15	0.269530717	0.561	0.422	2.62E-11
FJX1	2.49E-43	0.267322944	0.143	0.041	5.37E-39
PGRMC1	7.50E-12	0.267300674	0.713	0.62	1.62E-07
MYDGF	2.72E-16	0.266847242	0.694	0.576	5.87E-12
BTF3	5.33E-29	0.266673145	0.954	0.93	1.15E-24
EGR3	1.17E-23	0.26631144	0.286	0.154	2.53E-19
MIF	1.77E-15	0.266254271	0.708	0.583	3.82E-11
DIS3	1.01E-17	0.266016787	0.367	0.239	2.18E-13
COA4	3.00E-13	0.265970606	0.424	0.31	6.48E-09
PTPN1	4.41E-24	0.265913043	0.238	0.12	9.51E-20

IWS1	3.37E-19	0.264676741	0.322	0.197	7.28E-15
WAPL	6.85E-23	0.264510937	0.333	0.194	1.48E-18
PRDX1	1.32E-11	0.263930295	0.871	0.808	2.86E-07
NFE2L2	4.66E-16	0.263704093	0.767	0.656	1.00E-11
TFB2M	1.91E-57	0.263454551	0.202	0.06	4.12E-53
FGFRL1	4.39E-87	0.261178992	0.177	0.035	9.46E-83
DYNLL2	7.53E-15	0.260839194	0.35	0.233	1.62E-10
CMTM6	9.50E-18	0.260621416	0.405	0.269	2.05E-13
USP36	4.63E-35	0.260144961	0.263	0.116	9.99E-31
CYLD	4.92E-24	0.260098105	0.322	0.183	1.06E-19
UBE2B	6.90E-14	0.259579925	0.743	0.674	1.49E-09
KLHL18	9.04E-65	0.258697853	0.201	0.054	1.95E-60
MT-ND1	3.96E-52	0.258114976	0.996	0.991	8.54E-48
RRP9	2.00E-56	0.25765106	0.202	0.061	4.32E-52
EIF4G2	1.97E-17	0.257071596	0.774	0.664	4.24E-13
PPP1R15A	1.08E-05	0.256873682	0.815	0.784	0.232802168
SNAI2	0.002913803	0.256237582	0.271	0.233	1
TOMM20	1.67E-16	0.255841002	0.798	0.707	3.60E-12
UQCRFS1	3.79E-15	0.255791031	0.501	0.38	8.18E-11
EOGT	4.33E-78	0.255740355	0.154	0.03	9.33E-74
SOCS2	4.16E-14	0.255423497	0.219	0.131	8.97E-10
CDC27	6.46E-14	0.255288453	0.347	0.235	1.39E-09
RTF1	1.96E-12	0.255165421	0.589	0.476	4.23E-08
NGDN	7.48E-24	0.25514454	0.315	0.177	1.61E-19
UBE2J1	1.43E-19	0.25510274	0.381	0.245	3.09E-15
HAT1	1.36E-21	0.254341056	0.348	0.211	2.93E-17
USP14	1.87E-21	0.253980989	0.363	0.222	4.03E-17
MAP4K5	7.03E-18	0.253900936	0.363	0.235	1.52E-13
TMEM88	6.05E-98	0.253781892	0.112	0.013	1.30E-93
MRPL3	2.14E-24	0.253507388	0.405	0.25	4.62E-20
SLC2A1	3.87E-64	0.252902305	0.128	0.025	8.34E-60
NSRP1	9.12E-11	0.252627837	0.456	0.354	1.97E-06
YWHAQ	1.63E-12	0.252157725	0.767	0.672	3.51E-08
PLPP3	2.54E-11	0.251851653	0.912	0.853	5.48E-07
OGFRL1	4.00E-09	0.251751403	0.407	0.314	8.63E-05
PDK4	0.577013257	0.25171882	0.664	0.735	1
TSPYL1	6.75E-20	0.251648297	0.422	0.275	1.46E-15
SELENOT	5.15E-16	0.251557633	0.46	0.327	1.11E-11
SPATA2L	1.06E-36	0.251464978	0.164	0.056	2.29E-32
DEK	4.22E-16	0.251451932	0.776	0.652	9.10E-12
RCC2	1.45E-34	0.251451145	0.201	0.079	3.13E-30
ARF4	5.11E-14	0.251070601	0.762	0.636	1.10E-09
MED10	4.60E-16	0.250600272	0.516	0.384	9.92E-12
IPO4	4.70E-102	0.250387302	0.138	0.019	1.01E-97
RBM7	2.65E-14	0.250305901	0.432	0.312	5.71E-10

Supplementary Table S3a: Differentially expressed genes in each macrophage cluster identified by unsupervised clustering.

Gene name	p value	log2FC	percent.1	percent.2	FDR q value	cluster	subpopulation
APOE	2.08E-08	2.2649864	0.539	0.331	0.000449546	1	PVM
APOC1	2.76E-06	1.91453002	0.34	0.179	0.059534208	1	PVM
SLC40A1	8.04E-21	1.67172369	0.681	0.338	1.74E-16	1	PVM
FABP4	7.45E-09	1.63456755	0.44	0.214	0.000160679	1	PVM
TXNIP	1.80E-24	1.36912771	0.88	0.597	3.88E-20	1	PVM
CTSD	6.65E-19	1.369098	0.885	0.718	1.43E-14	1	PVM
SELENOP	2.33E-23	1.3499832	0.916	0.685	5.03E-19	1	PVM
TTN	6.02E-06	1.28359955	0.476	0.305	0.12993037	1	PVM
MAF	2.27E-20	1.21066972	0.89	0.581	4.90E-16	1	PVM
PLD3	1.83E-11	1.19180941	0.696	0.471	3.94E-07	1	PVM
GPNMB	5.21E-14	1.18744774	0.67	0.396	1.12E-09	1	PVM
ADAP2	4.23E-14	1.18589567	0.712	0.442	9.12E-10	1	PVM
RNASE1	7.53E-14	1.15687887	0.89	0.701	1.62E-09	1	PVM
CTSC	2.02E-16	1.13672164	0.874	0.672	4.35E-12	1	PVM
MAN1A1	2.61E-08	1.12008097	0.696	0.51	0.000562289	1	PVM
CD36	3.04E-10	1.10524523	0.581	0.351	6.56E-06	1	PVM
PDK4	9.13E-16	1.07639649	0.812	0.539	1.97E-11	1	PVM
GPR34	1.27E-14	1.04714117	0.691	0.403	2.74E-10	1	PVM
ACP5	3.83E-10	1.0376612	0.529	0.282	8.26E-06	1	PVM
DAB2	9.67E-17	1.01956275	0.864	0.588	2.09E-12	1	PVM
WWP1	2.13E-09	1.01118161	0.602	0.364	4.59E-05	1	PVM
FOLR2	9.33E-15	1.01000496	0.848	0.627	2.01E-10	1	PVM
F13A1	1.49E-09	1.00190178	0.717	0.523	3.21E-05	1	PVM
SCN9A	1.29E-08	0.99506917	0.382	0.175	0.000278252	1	PVM
LGMN1	5.92E-13	0.96804655	0.869	0.623	1.28E-08	1	PVM
LILRB51	1.71E-08	0.96280099	0.644	0.429	0.000368113	1	PVM
LRP1	1.12E-14	0.95890388	0.77	0.49	2.43E-10	1	PVM
PLTP	1.93E-12	0.93842534	0.733	0.477	4.16E-08	1	PVM
STAB1	8.04E-10	0.93445518	0.791	0.584	1.73E-05	1	PVM
FUCA1	1.31E-17	0.91933569	0.555	0.192	2.83E-13	1	PVM
MCOLN1	3.02E-12	0.90448028	0.597	0.334	6.52E-08	1	PVM
NRP11	1.05E-11	0.89074621	0.66	0.37	2.26E-07	1	PVM
TPM1	2.34E-09	0.87982235	0.503	0.263	5.05E-05	1	PVM
GAS6	4.75E-06	0.87040164	0.482	0.331	0.102551324	1	PVM
HPGDS	5.41E-11	0.8624198	0.382	0.14	1.17E-06	1	PVM
SNX6	1.35E-11	0.85352052	0.733	0.549	2.92E-07	1	PVM
EGFL7	2.69E-09	0.85330506	0.45	0.221	5.81E-05	1	PVM
PMP22	7.84E-08	0.849532	0.576	0.357	0.001691053	1	PVM
C1QC	4.32E-17	0.84802719	0.921	0.766	9.31E-13	1	PVM
COLEC12	5.77E-07	0.84726841	0.529	0.328	0.012438689	1	PVM
PLXND1	2.40E-08	0.83147308	0.565	0.347	0.000518647	1	PVM
CD68	1.85E-15	0.82879274	0.901	0.769	3.99E-11	1	PVM
LTC4S	9.47E-09	0.82728844	0.618	0.406	0.000204201	1	PVM
CSF1R	1.45E-10	0.82564037	0.827	0.701	3.13E-06	1	PVM
HRH1	4.31E-12	0.81752091	0.393	0.133	9.31E-08	1	PVM
STX7	9.51E-09	0.80945115	0.545	0.328	0.00020525	1	PVM
TREM2	3.58E-06	0.80626267	0.314	0.153	0.077223165	1	PVM
HTRA1	6.70E-09	0.7946894	0.293	0.101	0.000144611	1	PVM
MPEG1	4.77E-07	0.79406862	0.707	0.552	0.010300558	1	PVM

LIPA	0.000482	0.79278675	0.503	0.38	1	1	PVM
C1QA	2.48E-16	0.79045734	0.984	0.86	5.36E-12	1	PVM
SMC3	7.32E-12	0.78174174	0.398	0.149	1.58E-07	1	PVM
CMKLR1	3.23E-11	0.77804809	0.372	0.127	6.97E-07	1	PVM
MS4A4A	5.12E-10	0.77374885	0.832	0.672	1.10E-05	1	PVM
CD14	3.24E-08	0.77325478	0.806	0.682	0.000699069	1	PVM
A2M	2.82E-09	0.77000594	0.775	0.646	6.07E-05	1	PVM
SLCO2B1	7.63E-08	0.76968418	0.634	0.432	0.001646033	1	PVM
SCARB2	2.04E-07	0.75531698	0.534	0.334	0.00441024	1	PVM
DST	9.20E-07	0.7500222	0.56	0.377	0.019837949	1	PVM
CREG1	2.68E-10	0.74516657	0.738	0.555	5.77E-06	1	PVM
MAMDC2	7.07E-06	0.73595749	0.372	0.208	0.152547243	1	PVM
TMEM37	7.84E-11	0.73561218	0.471	0.208	1.69E-06	1	PVM
MXD4	1.09E-07	0.72631949	0.492	0.308	0.002351808	1	PVM
GYPC	7.35E-07	0.72587841	0.686	0.552	0.015865809	1	PVM
AP2A2	7.17E-07	0.713284	0.513	0.338	0.015457035	1	PVM
BRI3	1.03E-13	0.70756993	0.88	0.75	2.23E-09	1	PVM
C1QB	1.48E-14	0.70067695	0.948	0.808	3.20E-10	1	PVM
SESN3	0.000199	0.6982217	0.361	0.231	1	1	PVM
IGSF6	6.47E-05	0.68798825	0.597	0.474	1	1	PVM
AKAP9	0.000262	0.66943483	0.623	0.516	1	1	PVM
SESN1	2.73E-08	0.66907731	0.461	0.244	0.000588178	1	PVM
BLVRB	1.19E-08	0.66836134	0.848	0.731	0.000256452	1	PVM
UCP2	7.26E-08	0.66726175	0.665	0.497	0.001566589	1	PVM
CXCL121	0.000727	0.66508458	0.314	0.198	1	1	PVM
EPB41L2	5.30E-06	0.66340092	0.513	0.36	0.114279874	1	PVM
LYVE11	4.56E-05	0.662548	0.764	0.656	0.984071396	1	PVM
ITSN1	1.98E-05	0.66030734	0.513	0.351	0.426835459	1	PVM
WLS	2.03E-05	0.65929604	0.356	0.198	0.437084783	1	PVM
ZNF106	2.68E-06	0.65460397	0.518	0.338	0.057841124	1	PVM
EMB	1.80E-05	0.65070748	0.565	0.409	0.389233565	1	PVM
MYO5A	2.69E-06	0.65033234	0.529	0.354	0.057934193	1	PVM
TMEM176B	1.75E-06	0.64820307	0.618	0.438	0.037693403	1	PVM
LAMP2	1.80E-06	0.6474157	0.66	0.516	0.038889482	1	PVM
FXYD6	4.39E-07	0.64176639	0.288	0.11	0.00946224	1	PVM
MRC1	2.10E-07	0.63714336	0.89	0.789	0.004532521	1	PVM
C2	2.79E-07	0.62848368	0.361	0.166	0.006008405	1	PVM
HNMT	1.65E-08	0.62619841	0.644	0.455	0.000356186	1	PVM
RCSD1	2.14E-06	0.62474404	0.414	0.244	0.046223665	1	PVM
CD84	1.83E-07	0.62437939	0.649	0.435	0.003937569	1	PVM
LAMP1	4.68E-08	0.62407856	0.728	0.581	0.001009543	1	PVM
TIMP2	9.31E-07	0.62328859	0.696	0.581	0.020091077	1	PVM
KCTD12	6.01E-10	0.61882517	0.832	0.669	1.30E-05	1	PVM
KDM5A	0.000609	0.61837982	0.393	0.289	1	1	PVM
NCKAP1L	0.000137	0.61829432	0.571	0.438	1	1	PVM
SERPINB6	0.0001	0.61593018	0.555	0.416	1	1	PVM
ATM	8.54E-05	0.61389193	0.545	0.393	1	1	PVM
ASAH1	5.00E-09	0.61241662	0.801	0.682	0.000107783	1	PVM
UACA	0.000128	0.61089305	0.272	0.136	1	1	PVM
UNC93B1	2.76E-07	0.61020117	0.576	0.38	0.005956706	1	PVM
PCMTD1	1.39E-05	0.60828279	0.435	0.256	0.29967044	1	PVM
ATRX	0.001976	0.60812143	0.576	0.461	1	1	PVM

PECAM1	2.29E-06	0.60064989	0.471	0.292	0.049316695	1	PVM
ME1	2.07E-06	0.59428223	0.366	0.195	0.044593583	1	PVM
SHPRH	0.000207	0.59311487	0.22	0.107	1	1	PVM
RNF213	0.000558	0.59277914	0.565	0.461	1	1	PVM
GTF2I	2.35E-06	0.59168115	0.539	0.36	0.050593416	1	PVM
TNFAIP21	0.000702	0.58937656	0.691	0.568	1	1	PVM
CCDC141	0.000147	0.58856349	0.23	0.11	1	1	PVM
P2RX7	0.00033	0.58692594	0.314	0.188	1	1	PVM
SNRNP200	9.18E-07	0.58667586	0.351	0.169	0.019797238	1	PVM
PYCARD	1.13E-06	0.58665992	0.665	0.545	0.024397528	1	PVM
RALBP1	0.000292	0.58530267	0.445	0.299	1	1	PVM
SP100	2.67E-05	0.58297372	0.445	0.292	0.576373402	1	PVM
TPCN1	0.000271	0.57557536	0.366	0.227	1	1	PVM
ACIN1	0.000314	0.57286075	0.408	0.279	1	1	PVM
IL10RA	2.83E-05	0.57279026	0.56	0.399	0.61056928	1	PVM
TPP1	3.03E-06	0.57203096	0.712	0.539	0.065380215	1	PVM
IGF1	0.000168	0.57050915	0.408	0.263	1	1	PVM
NINJ1	0.000102	0.56823551	0.565	0.422	1	1	PVM
LAIR1	6.11E-06	0.56635017	0.607	0.432	0.131779718	1	PVM
KMT2A	0.000188	0.56509634	0.398	0.256	1	1	PVM
EPHX1	2.18E-06	0.5631918	0.319	0.149	0.046955727	1	PVM
CIR1	3.18E-06	0.56309374	0.393	0.214	0.06866752	1	PVM
GIMAP7	1.31E-05	0.5604337	0.304	0.149	0.28232375	1	PVM
TLR4	3.12E-05	0.5574046	0.466	0.299	0.672585946	1	PVM
HIST1H4C	8.07E-05	0.5570011	0.529	0.383	1	1	PVM
CCL18	1.22E-05	0.556629	0.168	0.049	0.263428157	1	PVM
MGLL	0.000241	0.55433596	0.309	0.185	1	1	PVM
COLGALT1	0.000402	0.55340782	0.476	0.341	1	1	PVM
CD151	5.32E-05	0.55340101	0.403	0.256	1	1	PVM
RASSF4	0.0001	0.55306231	0.586	0.445	1	1	PVM
PDS5B	2.65E-05	0.55085744	0.314	0.172	0.570723047	1	PVM
ZBTB38	3.16E-05	0.5502308	0.361	0.211	0.680715072	1	PVM
CCDC170	1.05E-05	0.55013202	0.293	0.14	0.226738005	1	PVM
PDGFC	0.002549	0.54908148	0.325	0.224	1	1	PVM
ST6GAL1	2.72E-06	0.54745937	0.288	0.13	0.058598665	1	PVM
PLA2G15	1.23E-08	0.54383413	0.33	0.127	0.000265567	1	PVM
GUSB	5.30E-05	0.54292972	0.377	0.237	1	1	PVM
ABCC5	2.03E-06	0.53238606	0.366	0.182	0.043795675	1	PVM
ATF7IP	0.000804	0.52996412	0.351	0.234	1	1	PVM
HERC1	0.000167	0.52879866	0.335	0.201	1	1	PVM
MTIF3	0.000403	0.52624355	0.398	0.26	1	1	PVM
RSBN1L	1.21E-08	0.52528447	0.382	0.159	0.000261879	1	PVM
FEZ2	5.37E-05	0.52508949	0.471	0.308	1	1	PVM
ITGB5	3.74E-08	0.52461414	0.424	0.205	0.000805944	1	PVM
GIMAP4	1.23E-06	0.52250297	0.403	0.211	0.026544814	1	PVM
RPN2	3.54E-05	0.51976307	0.55	0.377	0.763574263	1	PVM
GAA	9.76E-05	0.51879349	0.487	0.37	1	1	PVM
CD59	2.17E-05	0.51699341	0.476	0.315	0.469128584	1	PVM
GLMP	4.65E-08	0.51517011	0.288	0.101	0.001003827	1	PVM
GPR155	0.001374	0.51286132	0.346	0.224	1	1	PVM
PTMS	1.92E-05	0.51068626	0.623	0.497	0.413196567	1	PVM
FRMD4B1	9.20E-05	0.51010181	0.681	0.552	1	1	PVM

SYK	5.82E-05	0.50721402	0.503	0.364	1	1	PVM
RERE	0.000637	0.50674088	0.351	0.227	1	1	PVM
SGMS11	2.39E-05	0.5065475	0.414	0.24	0.515933701	1	PVM
POLR2J	6.91E-05	0.50629074	0.424	0.266	1	1	PVM
FCHO2	0.000189	0.50553423	0.471	0.315	1	1	PVM
TSPAN4	0.003765	0.5040623	0.581	0.468	1	1	PVM
RABGAP1	6.04E-06	0.50243328	0.251	0.104	0.130307141	1	PVM
KIAA1551	0.000766	0.50229986	0.471	0.344	1	1	PVM
NAGK	0.000328	0.50220567	0.534	0.412	1	1	PVM
SNX29	0.002908	0.50140193	0.377	0.26	1	1	PVM
AKR1B1	0.000379	0.50101212	0.471	0.331	1	1	PVM
HTR2B	3.46E-09	0.50055692	0.152	0.016	7.46E-05	1	PVM
C3AR1	0.000527	0.49995641	0.602	0.49	1	1	PVM
FCER2	0.00012	0.49975187	0.178	0.071	1	1	PVM
ADCY7	0.001853	0.49740647	0.387	0.269	1	1	PVM
ZYX	0.000119	0.49722511	0.539	0.416	1	1	PVM
MARCH1	0.000162	0.4969392	0.487	0.344	1	1	PVM
VPS13C	4.18E-06	0.49544143	0.665	0.464	0.090111707	1	PVM
RNF135	2.20E-07	0.49499334	0.309	0.13	0.004737724	1	PVM
MAVS	0.000217	0.49393538	0.225	0.11	1	1	PVM
DTX3L	1.68E-05	0.49380106	0.251	0.114	0.362054836	1	PVM
HOMER3	1.54E-06	0.49369692	0.44	0.24	0.033184429	1	PVM
DNAJC13	0.000362	0.49324254	0.283	0.159	1	1	PVM
NDUFB7	0.000163	0.49188839	0.524	0.409	1	1	PVM
PKN2	0.001811	0.49164165	0.377	0.269	1	1	PVM
RBMS1	0.000453	0.49064425	0.55	0.432	1	1	PVM
CCDC112	6.01E-05	0.48989149	0.257	0.123	1	1	PVM
HGF	1.41E-05	0.48543921	0.262	0.117	0.304520992	1	PVM
MPHOSPH8	1.78E-05	0.48216734	0.518	0.338	0.383058393	1	PVM
CHD9	3.31E-05	0.48004401	0.424	0.253	0.714293016	1	PVM
NFIC	0.002397	0.47896197	0.414	0.305	1	1	PVM
ZFHX3	0.000258	0.47834561	0.623	0.487	1	1	PVM
FKBP15	6.13E-05	0.47801626	0.325	0.179	1	1	PVM
FAM13A1	0.00357	0.47316266	0.419	0.312	1	1	PVM
SMIM7	0.002358	0.47252256	0.393	0.282	1	1	PVM
RBM26	6.37E-05	0.4721912	0.319	0.179	1	1	PVM
CREBL2	0.000255	0.47006096	0.414	0.292	1	1	PVM
TNS1	0.000886	0.46980375	0.283	0.169	1	1	PVM
C9	4.99E-07	0.46966797	0.162	0.036	0.010757793	1	PVM
MGAT4A	0.000355	0.46525661	0.298	0.172	1	1	PVM
ZNF22	5.22E-05	0.46299946	0.314	0.162	1	1	PVM
SNX24	5.86E-06	0.46223241	0.251	0.104	0.126360813	1	PVM
TGOLN2	0.000142	0.4620091	0.66	0.545	1	1	PVM
RNF150	2.36E-05	0.4588026	0.157	0.049	0.508584187	1	PVM
JPX	2.97E-05	0.45722838	0.272	0.13	0.640668946	1	PVM
SPRED1	4.83E-06	0.45673969	0.513	0.318	0.104222599	1	PVM
PIK3IP1	8.60E-05	0.45501127	0.246	0.117	1	1	PVM
SMIM26	0.001757	0.45404848	0.445	0.328	1	1	PVM
HP1BP3	0.001049	0.45377392	0.581	0.455	1	1	PVM
NDUFS3	0.001085	0.45202493	0.283	0.172	1	1	PVM
DIP2A	0.000338	0.45171055	0.283	0.156	1	1	PVM
PDIA4	3.93E-07	0.4516519	0.319	0.136	0.008480713	1	PVM

DAAM1	0.002157	0.45153361	0.283	0.179	1	1	PVM
PHIP	0.00042	0.45015689	0.513	0.386	1	1	PVM
RHOBTB1	6.52E-06	0.44619706	0.168	0.049	0.140742523	1	PVM
PRKACB	0.000135	0.44476042	0.387	0.253	1	1	PVM
ASPH	8.36E-05	0.44474747	0.377	0.231	1	1	PVM
MLXIP	0.00015	0.44315225	0.351	0.205	1	1	PVM
TRIP11	0.005639	0.44130411	0.356	0.25	1	1	PVM
MORC3	0.000247	0.44067734	0.346	0.211	1	1	PVM
CXorf21	8.46E-08	0.43795412	0.241	0.075	0.001825402	1	PVM
RICTOR	0.00019	0.43696963	0.236	0.114	1	1	PVM
TUT4	0.003508	0.4365631	0.293	0.192	1	1	PVM
VAT1	1.19E-07	0.43630768	0.387	0.175	0.002565763	1	PVM
MERTK	7.92E-06	0.43595755	0.366	0.188	0.170752966	1	PVM
LYL1	5.66E-05	0.43409918	0.366	0.218	1	1	PVM
UBN2	0.000467	0.43339862	0.215	0.107	1	1	PVM
LRRC25	0.001371	0.43289958	0.377	0.26	1	1	PVM
TBC1D2B	0.001995	0.43255812	0.241	0.136	1	1	PVM
KIAA0930	0.001079	0.43174756	0.398	0.279	1	1	PVM
ARHGAP18	0.000549	0.43163359	0.665	0.542	1	1	PVM
ARID1B	0.001986	0.43107315	0.435	0.328	1	1	PVM
C20orf194	0.000776	0.43012747	0.34	0.208	1	1	PVM
BLNK	0.000141	0.42983875	0.236	0.114	1	1	PVM
SPPL2A	0.000185	0.42965806	0.461	0.321	1	1	PVM
GIMAP2	3.49E-06	0.42800049	0.246	0.097	0.075389	1	PVM
TGFBR1	1.20E-05	0.42677036	0.335	0.169	0.258146181	1	PVM
LILRB4	0.001073	0.42524888	0.382	0.253	1	1	PVM
GNG11	0.002928	0.42485087	0.251	0.146	1	1	PVM
EVL	0.001433	0.42425218	0.33	0.224	1	1	PVM
DHRS9	9.43E-05	0.42421065	0.272	0.14	1	1	PVM
DEGS1	0.000759	0.42406792	0.471	0.331	1	1	PVM
PHF3	0.00294	0.42261315	0.366	0.26	1	1	PVM
GCNT1	0.000279	0.42161653	0.209	0.101	1	1	PVM
CD2091	0.000228	0.41736357	0.319	0.182	1	1	PVM
GATM	0.000273	0.4166363	0.314	0.182	1	1	PVM
KDELR1	0.002358	0.41421406	0.45	0.338	1	1	PVM
KANSL1	0.000454	0.41385791	0.335	0.208	1	1	PVM
MGAT5	0.000104	0.4137027	0.246	0.117	1	1	PVM
HELZ	0.002615	0.41251765	0.361	0.244	1	1	PVM
KLF3	0.007083	0.41241035	0.403	0.299	1	1	PVM
P2RX4	2.06E-05	0.4116991	0.34	0.188	0.445200422	1	PVM
TCEAL4	0.003382	0.40981758	0.419	0.299	1	1	PVM
PCYOX1	3.26E-06	0.40975919	0.23	0.084	0.070222826	1	PVM
FARP1	8.98E-05	0.40965525	0.236	0.107	1	1	PVM
GCC2	0.0002	0.40844898	0.492	0.351	1	1	PVM
PLEKHA1	0.002072	0.40820865	0.236	0.133	1	1	PVM
GPR137B	0.006161	0.40792519	0.314	0.214	1	1	PVM
GIMAP1	0.000963	0.40726899	0.262	0.149	1	1	PVM
TNFRSF1A	0.000363	0.40690465	0.461	0.315	1	1	PVM
FTX	3.30E-05	0.40493287	0.304	0.153	0.710976633	1	PVM
VPS13D	0.000215	0.40450362	0.23	0.117	1	1	PVM
NECAP2	0.001082	0.4045018	0.382	0.253	1	1	PVM
NCOA4	3.03E-05	0.40403146	0.586	0.403	0.652738714	1	PVM

CTSF	0.000661	0.40403093	0.204	0.097	1	1	PVM
ENG	2.93E-05	0.40363534	0.361	0.201	0.631327482	1	PVM
PTPN18	0.000773	0.40318429	0.419	0.289	1	1	PVM
MBTPS1	0.000793	0.40268425	0.22	0.114	1	1	PVM
FRMD4A	0.000106	0.39904832	0.366	0.205	1	1	PVM
TMEM127	0.001465	0.39850687	0.272	0.166	1	1	PVM
SCAMP2	0.001366	0.39813678	0.518	0.412	1	1	PVM
ARL8A	0.002488	0.39734537	0.346	0.231	1	1	PVM
RAD21	1.93E-05	0.39695376	0.435	0.253	0.416644539	1	PVM
NAGA	0.00302	0.39689307	0.351	0.24	1	1	PVM
PAK2	0.000542	0.3963797	0.55	0.419	1	1	PVM
NCEH1	0.000984	0.39609043	0.267	0.156	1	1	PVM
TGFBR2	0.002429	0.39518815	0.455	0.338	1	1	PVM
SH3BP2	0.0003	0.39474306	0.361	0.218	1	1	PVM
ATP6V0A1	4.31E-05	0.39463967	0.382	0.221	0.930456069	1	PVM
IFFO1	0.000493	0.39413103	0.23	0.117	1	1	PVM
SPOP	0.000119	0.39339862	0.382	0.231	1	1	PVM
EPS15	0.004961	0.39280478	0.44	0.334	1	1	PVM
KIAA2026	0.000626	0.39210121	0.314	0.188	1	1	PVM
MACF1	0.001416	0.39163255	0.503	0.396	1	1	PVM
AIDA	0.000254	0.38895777	0.325	0.192	1	1	PVM
MMP14	6.69E-07	0.38837403	0.272	0.104	0.014428648	1	PVM
PARP1	0.000352	0.38798646	0.325	0.192	1	1	PVM
DICER1	0.003632	0.38766357	0.414	0.302	1	1	PVM
CCPG1	0.001973	0.38684289	0.34	0.224	1	1	PVM
SETX	0.003142	0.38533126	0.487	0.386	1	1	PVM
MBD4	0.001442	0.38446338	0.304	0.192	1	1	PVM
SCAND1	0.000358	0.38387408	0.471	0.331	1	1	PVM
CSGALNACT	0.000541	0.38353232	0.204	0.097	1	1	PVM
TRIM14	0.000198	0.383068	0.33	0.188	1	1	PVM
SLC43A2	0.003062	0.38264434	0.435	0.331	1	1	PVM
RNASEH2B	0.000741	0.38175966	0.382	0.25	1	1	PVM
ATP8B4	0.000368	0.38091522	0.283	0.156	1	1	PVM
CYTH4	0.001567	0.37957748	0.445	0.318	1	1	PVM
POU2F1	0.000998	0.37891809	0.194	0.094	1	1	PVM
LTBP2	0.001495	0.37726753	0.209	0.107	1	1	PVM
MSR1	0.000105	0.37637818	0.759	0.643	1	1	PVM
RIF1	0.005605	0.37342467	0.319	0.211	1	1	PVM
LRRC58	0.001025	0.37321044	0.267	0.153	1	1	PVM
SREK1IP1	0.006409	0.37284484	0.33	0.224	1	1	PVM
RCN3	0.001482	0.37151343	0.277	0.166	1	1	PVM
ARID4A	0.001038	0.37113938	0.429	0.299	1	1	PVM
ATRAID	0.001975	0.37007302	0.45	0.347	1	1	PVM
MGST3	0.000325	0.36957482	0.602	0.5	1	1	PVM
PNPLA6	0.000304	0.36832406	0.304	0.175	1	1	PVM
NUPR1	0.002796	0.36808848	0.251	0.146	1	1	PVM
TBC1D5	0.001274	0.36596133	0.272	0.156	1	1	PVM
LGI2	6.61E-05	0.36402534	0.162	0.055	1	1	PVM
CLSTN1	0.000397	0.36294444	0.22	0.107	1	1	PVM
EEA1	0.000596	0.36272083	0.414	0.273	1	1	PVM
HEBP1	0.004444	0.36242113	0.33	0.227	1	1	PVM
PIAS1	0.000325	0.36134868	0.366	0.224	1	1	PVM

CHD8	0.002847	0.36080649	0.262	0.159	1	1	PVM
ARHGEF2	0.001104	0.35928567	0.298	0.182	1	1	PVM
RAB13	0.002256	0.35863801	0.445	0.331	1	1	PVM
TUBB	0.000404	0.35593301	0.581	0.455	1	1	PVM
LTN1	0.002486	0.3555088	0.241	0.14	1	1	PVM
KCNE3	9.75E-07	0.35483096	0.209	0.065	0.02103198	1	PVM
ATP6V1A	0.000248	0.3542144	0.277	0.146	1	1	PVM
ACAP2	0.000792	0.35408982	0.518	0.377	1	1	PVM
PPP1R14B	0.00558	0.35393737	0.445	0.334	1	1	PVM
PINK1	1.26E-05	0.35376682	0.215	0.081	0.271489824	1	PVM
DRAM2	0.00038	0.35283271	0.524	0.373	1	1	PVM
RND3	0.003764	0.3525762	0.257	0.149	1	1	PVM
SEPT11	0.0041	0.35184261	0.429	0.318	1	1	PVM
TMEM179B	0.002721	0.34963742	0.335	0.234	1	1	PVM
GPR65	0.004345	0.34859036	0.33	0.221	1	1	PVM
GNPDA1	0.000195	0.34843965	0.424	0.266	1	1	PVM
HIST1H1E	0.00013	0.34444095	0.152	0.052	1	1	PVM
VEGFB	0.001499	0.34356613	0.419	0.305	1	1	PVM
CDKN1B	0.000958	0.34323651	0.199	0.097	1	1	PVM
WDFY3	0.001642	0.34260734	0.33	0.214	1	1	PVM
SSH1	0.000654	0.34123315	0.225	0.117	1	1	PVM
BMP2K	8.14E-05	0.34072279	0.508	0.344	1	1	PVM
ADH5	0.001098	0.33892884	0.277	0.162	1	1	PVM
RRAS	0.000813	0.338597	0.283	0.162	1	1	PVM
GATAD1	0.00536	0.33853583	0.361	0.244	1	1	PVM
TNFSF12	0.002214	0.33784884	0.377	0.25	1	1	PVM
ADAM9	0.000352	0.33754241	0.414	0.266	1	1	PVM
BLCAP	8.19E-06	0.33704063	0.22	0.081	0.176572617	1	PVM
CSNK2A1	0.000833	0.33617625	0.298	0.175	1	1	PVM
CAT	0.004887	0.33613307	0.539	0.432	1	1	PVM
DCTN2	0.002775	0.33556359	0.262	0.162	1	1	PVM
WAC-AS1	0.000292	0.33362957	0.23	0.11	1	1	PVM
MKNK1	0.00397	0.33116247	0.466	0.347	1	1	PVM
WDR11	4.36E-05	0.33112886	0.241	0.107	0.941419749	1	PVM
CYB561A3	0.001085	0.3293502	0.225	0.117	1	1	PVM
SLC35F6	0.002154	0.32905951	0.272	0.162	1	1	PVM
NUMA1	0.001464	0.32785136	0.335	0.221	1	1	PVM
SLC18B1	0.000937	0.325415	0.251	0.14	1	1	PVM
PHACTR4	0.002607	0.32539611	0.346	0.231	1	1	PVM
CTCF	0.000296	0.32485915	0.293	0.166	1	1	PVM
LMBRD1	0.000835	0.32446627	0.335	0.208	1	1	PVM
CLPB	0.000354	0.32427207	0.173	0.071	1	1	PVM
CYFIP1	0.000491	0.32394602	0.435	0.295	1	1	PVM
NSL1	0.002596	0.32363777	0.314	0.205	1	1	PVM
DMXL2	0.002555	0.32353771	0.398	0.26	1	1	PVM
MESD	0.003546	0.3234635	0.346	0.231	1	1	PVM
MRPL34	4.42E-05	0.32148932	0.33	0.179	0.952902405	1	PVM
ZDHHC7	0.000127	0.31861979	0.304	0.159	1	1	PVM
APC	0.001208	0.31854996	0.33	0.205	1	1	PVM
ASB8	0.0004	0.31748367	0.204	0.094	1	1	PVM
ABCA11	0.002224	0.31721303	0.613	0.487	1	1	PVM
YWHAH	0.009268	0.3164604	0.471	0.36	1	1	PVM

DNAJC7	0.008987	0.31556575	0.45	0.344	1	1	PVM
FCGR3A	0.00684	0.31464924	0.487	0.367	1	1	PVM
NDUFS2	0.002982	0.3144872	0.33	0.221	1	1	PVM
SLC27A1	7.38E-05	0.31438581	0.204	0.084	1	1	PVM
TM9SF2	0.008363	0.31283597	0.408	0.302	1	1	PVM
DCAF7	0.000279	0.31240656	0.262	0.13	1	1	PVM
YPEL3	0.002404	0.31087896	0.382	0.266	1	1	PVM
GPAA1	0.000837	0.31004477	0.304	0.182	1	1	PVM
VPS4B	0.001071	0.30937832	0.361	0.231	1	1	PVM
SACM1L	1.35E-05	0.30930207	0.183	0.058	0.291464292	1	PVM
SMAP1	0.001937	0.30808621	0.298	0.185	1	1	PVM
CUTA	0.004134	0.30727398	0.419	0.299	1	1	PVM
DNASE2	3.00E-06	0.30675799	0.22	0.075	0.064749867	1	PVM
PARP8	0.001863	0.30535238	0.293	0.182	1	1	PVM
MSRB2	0.00086	0.30521236	0.325	0.198	1	1	PVM
WDR73	0.000134	0.30510512	0.209	0.091	1	1	PVM
PARP4	0.005077	0.30493868	0.314	0.205	1	1	PVM
HADH	0.000709	0.30456745	0.246	0.136	1	1	PVM
TCF12	0.002488	0.30282753	0.34	0.224	1	1	PVM
OSBPL1A	0.002673	0.30221435	0.304	0.188	1	1	PVM
GLB1	4.35E-05	0.30204553	0.304	0.149	0.93932497	1	PVM
C14orf119	0.002613	0.30164677	0.288	0.179	1	1	PVM
RAB3IL1	2.94E-05	0.30095824	0.188	0.068	0.633317731	1	PVM
SLC15A3	0.003928	0.30084796	0.414	0.299	1	1	PVM
BMPR2	0.000139	0.30034702	0.23	0.107	1	1	PVM
CNPY2	0.004793	0.29986208	0.293	0.192	1	1	PVM
ARHGAP25	0.000192	0.29982906	0.178	0.071	1	1	PVM
FNTA	0.001043	0.29753456	0.298	0.179	1	1	PVM
GOPC	0.000852	0.29732429	0.241	0.127	1	1	PVM
MSL1	0.005025	0.29716379	0.293	0.185	1	1	PVM
GALK1	0.00032	0.29661835	0.183	0.078	1	1	PVM
COMMD9	5.58E-05	0.29579816	0.314	0.166	1	1	PVM
UBXN7	0.000128	0.29479732	0.22	0.097	1	1	PVM
VPS26C	0.000473	0.29477873	0.194	0.088	1	1	PVM
PTPRJ	0.001214	0.29347058	0.267	0.159	1	1	PVM
SYNGR2	0.000765	0.29286116	0.56	0.429	1	1	PVM
FAM192A	0.000834	0.2921502	0.251	0.136	1	1	PVM
GORASP2	0.000106	0.28903023	0.178	0.068	1	1	PVM
AC118549.1	0.000811	0.28793344	0.215	0.107	1	1	PVM
NSFL1C	0.003019	0.28713361	0.293	0.185	1	1	PVM
CRYL1	0.001759	0.28536511	0.361	0.231	1	1	PVM
RGS19	0.004205	0.28389176	0.34	0.231	1	1	PVM
TNFRSF11A	0.00256	0.28347221	0.236	0.133	1	1	PVM
SNAP29	0.002501	0.28335722	0.257	0.149	1	1	PVM
STAT5A	0.000167	0.28228394	0.272	0.14	1	1	PVM
CYBRD1	0.002339	0.2813563	0.335	0.221	1	1	PVM
PRDX3	0.008494	0.28126358	0.393	0.289	1	1	PVM
SLC7A6OS	3.22E-05	0.28039655	0.168	0.055	0.693719078	1	PVM
RASAL2	0.000293	0.28015604	0.204	0.091	1	1	PVM
RPN1	0.008072	0.2793965	0.408	0.299	1	1	PVM
GLG1	0.002384	0.27764391	0.335	0.221	1	1	PVM
CEPT1	0.001189	0.27631021	0.267	0.149	1	1	PVM

APOBEC3C	0.00379	0.27604845	0.246	0.146	1	1	PVM
MILR1	0.00061	0.27298802	0.236	0.12	1	1	PVM
PSMD4	0.001827	0.27212778	0.382	0.26	1	1	PVM
MED11	2.09E-05	0.27058782	0.215	0.084	0.451075671	1	PVM
HDAC2	0.002405	0.26957517	0.293	0.172	1	1	PVM
ARAP1	0.002754	0.26718044	0.408	0.279	1	1	PVM
PTBP3	0.004215	0.26497927	0.508	0.393	1	1	PVM
SNX27	0.00659	0.26459438	0.272	0.172	1	1	PVM
FAM213A	8.16E-05	0.26271708	0.194	0.078	1	1	PVM
PPCS	0.004865	0.26174889	0.372	0.266	1	1	PVM
CD180	1.86E-05	0.26152491	0.225	0.088	0.402086511	1	PVM
PPP1R21	0.000356	0.2597179	0.194	0.084	1	1	PVM
UBE2M	0.006035	0.25653898	0.314	0.208	1	1	PVM
TMEM87B	0.000269	0.25496239	0.162	0.062	1	1	PVM
CDS2	0.001035	0.25022445	0.188	0.088	1	1	PVM
ACP2	0.000583	0.25012881	0.22	0.11	1	1	PVM
CXCL8	1.09E-20	3.42745135	0.662	0.311	2.36E-16	2	PVM
CCL3	4.14E-18	2.58235154	0.662	0.311	8.92E-14	2	PVM
CCL4	1.08E-07	2.41102246	0.368	0.16	0.002321478	2	PVM
EIF4E	1.92E-17	2.26556894	0.596	0.259	4.14E-13	2	PVM
CCL2	1.38E-11	2.21085939	0.507	0.215	2.99E-07	2	PVM
HSPA6	1.00E-11	2.1806921	0.61	0.369	2.16E-07	2	PVM
HSPH1	1.75E-27	2.15810086	0.912	0.73	3.78E-23	2	PVM
BAG3	4.18E-23	2.13459848	0.728	0.331	9.02E-19	2	PVM
CCL3L1	8.03E-11	2.1244994	0.397	0.149	1.73E-06	2	PVM
HSPD1	2.57E-25	2.02477191	0.912	0.749	5.54E-21	2	PVM
CD83	7.51E-28	2.00845706	0.853	0.556	1.62E-23	2	PVM
HSPA1B	1.17E-25	1.96036465	0.926	0.769	2.53E-21	2	PVM
HMOX1	9.95E-14	1.82794443	0.831	0.711	2.15E-09	2	PVM
DNAJB1	8.39E-22	1.81249246	0.949	0.81	1.81E-17	2	PVM
KLF6	4.73E-23	1.77433453	0.904	0.782	1.02E-18	2	PVM
EGR1	1.11E-15	1.7664365	0.904	0.763	2.39E-11	2	PVM
CXCL3	3.45E-10	1.75445571	0.456	0.207	7.44E-06	2	PVM
ATF3	6.02E-26	1.74751894	0.875	0.562	1.30E-21	2	PVM
CXCL2	1.73E-11	1.66885934	0.574	0.298	3.74E-07	2	PVM
DNAJA4	7.02E-10	1.54198031	0.449	0.218	1.52E-05	2	PVM
NR4A2	1.33E-13	1.53677268	0.801	0.587	2.88E-09	2	PVM
CCL8	5.13E-11	1.53525416	0.346	0.102	1.11E-06	2	PVM
PLIN2	4.61E-09	1.46052506	0.632	0.463	9.94E-05	2	PVM
FOSB	4.38E-18	1.46029198	0.978	0.815	9.44E-14	2	PVM
RASGEF1B	1.87E-23	1.40119779	0.809	0.444	4.03E-19	2	PVM
KDM6B	1.87E-18	1.33382369	0.86	0.562	4.03E-14	2	PVM
UBE2S	2.28E-13	1.31506623	0.721	0.471	4.92E-09	2	PVM
THBD	6.86E-10	1.28752784	0.537	0.273	1.48E-05	2	PVM
IER3	4.90E-12	1.27691996	0.809	0.645	1.06E-07	2	PVM
CDKN1A	5.59E-20	1.26984584	0.831	0.551	1.21E-15	2	PVM
DNAJB6	4.41E-24	1.24601454	0.919	0.689	9.51E-20	2	PVM
SQSTM1	9.63E-14	1.2262374	0.794	0.664	2.08E-09	2	PVM
MRPL18	1.36E-12	1.21799477	0.684	0.449	2.94E-08	2	PVM
FNIP2	3.99E-13	1.21774182	0.537	0.259	8.60E-09	2	PVM
TRIB1	4.70E-12	1.20597917	0.551	0.267	1.01E-07	2	PVM
NR4A1	1.13E-12	1.20512652	0.816	0.573	2.43E-08	2	PVM

NFKB1	2.92E-11	1.19544404	0.551	0.3	6.30E-07	2	PVM
PPP1R15B	5.26E-18	1.18789945	0.625	0.275	1.13E-13	2	PVM
DNTTIP2	1.65E-14	1.18202012	0.684	0.399	3.56E-10	2	PVM
B3GNT5	2.06E-09	1.16610385	0.419	0.19	4.44E-05	2	PVM
ZNF267	3.35E-11	1.16095889	0.515	0.242	7.23E-07	2	PVM
ARC	6.12E-08	1.10764885	0.382	0.176	0.001320959	2	PVM
CHORDC1	1.44E-12	1.10214798	0.728	0.485	3.11E-08	2	PVM
SPAG9	1.05E-12	1.08031434	0.743	0.523	2.26E-08	2	PVM
IER2	4.14E-08	1.06887947	0.904	0.796	0.000893174	2	PVM
PTGS2	2.66E-08	1.06793486	0.375	0.163	0.000573412	2	PVM
DDX3Y	1.08E-11	1.0536199	0.838	0.639	2.33E-07	2	PVM
CITED2	2.77E-06	1.05273097	0.662	0.523	0.059766697	2	PVM
EIF4A3	3.65E-13	1.04147706	0.75	0.523	7.87E-09	2	PVM
HBEGF	6.23E-15	1.0337932	0.824	0.471	1.34E-10	2	PVM
SOD2	2.57E-07	1.02192657	0.743	0.59	0.005549338	2	PVM
JMJD1C	1.10E-10	1.01427036	0.846	0.708	2.37E-06	2	PVM
CSRNP1	9.26E-15	1.01118035	0.632	0.3	2.00E-10	2	PVM
PPP1R15A	1.83E-16	1.00367443	0.912	0.752	3.95E-12	2	PVM
GTF2B	3.16E-09	0.99362771	0.603	0.375	6.81E-05	2	PVM
CKS2	2.34E-10	0.99186574	0.537	0.298	5.05E-06	2	PVM
SLC25A44	2.27E-12	0.98979809	0.478	0.185	4.89E-08	2	PVM
ABL2	2.09E-07	0.98454058	0.493	0.303	0.004506911	2	PVM
GPR183	2.09E-07	0.9808267	0.794	0.573	0.004505848	2	PVM
NR4A3	3.14E-09	0.97968817	0.588	0.32	6.76E-05	2	PVM
EGR3	2.23E-12	0.97410404	0.426	0.152	4.80E-08	2	PVM
NEU1	1.11E-08	0.97291595	0.662	0.46	0.00023923	2	PVM
TNFAIP3	3.97E-06	0.96656875	0.632	0.455	0.085680038	2	PVM
CPEB4	1.21E-09	0.94048546	0.713	0.479	2.60E-05	2	PVM
EGR2	7.22E-08	0.93747634	0.316	0.121	0.001557878	2	PVM
ATF4	1.67E-13	0.92951899	0.801	0.587	3.61E-09	2	PVM
PELI1	3.32E-11	0.92547906	0.64	0.369	7.16E-07	2	PVM
MCL1	2.49E-12	0.91993129	0.934	0.796	5.37E-08	2	PVM
SNHG12	0.000306	0.90573732	0.522	0.386	1	2	PVM
RAB7A	2.41E-11	0.89707395	0.743	0.57	5.20E-07	2	PVM
ICAM1	4.49E-11	0.89703468	0.522	0.237	9.70E-07	2	PVM
SERTAD1	2.38E-10	0.89407069	0.779	0.601	5.12E-06	2	PVM
KLF4	8.54E-11	0.89340284	0.882	0.744	1.84E-06	2	PVM
MAP1LC3B	3.24E-10	0.88836784	0.816	0.667	6.99E-06	2	PVM
GPBP1	3.13E-09	0.87256178	0.684	0.477	6.76E-05	2	PVM
RYBP	5.92E-10	0.87248348	0.596	0.331	1.28E-05	2	PVM
MIDN	1.39E-10	0.86913897	0.816	0.595	2.99E-06	2	PVM
LRIF1	7.05E-06	0.86060438	0.368	0.201	0.152076773	2	PVM
ADM	1.68E-09	0.85222821	0.64	0.386	3.61E-05	2	PVM
BRD2	7.13E-10	0.85101794	0.801	0.62	1.54E-05	2	PVM
PCF11	1.65E-06	0.85080475	0.625	0.452	0.035575848	2	PVM
PNP	9.17E-08	0.84848321	0.588	0.383	0.001978521	2	PVM
ARL6IP1	6.58E-09	0.84647637	0.721	0.598	0.000142019	2	PVM
ATP1A1	9.44E-07	0.84407868	0.618	0.474	0.020361914	2	PVM
ERO1B	1.99E-06	0.83773055	0.485	0.284	0.042991978	2	PVM
CYB5D1	6.86E-14	0.83611887	0.36	0.091	1.48E-09	2	PVM
AFF4	2.47E-11	0.82822792	0.765	0.504	5.32E-07	2	PVM
GADD45B	4.81E-07	0.82266406	0.949	0.846	0.010385507	2	PVM

SFPQ	1.29E-11	0.8213751	0.882	0.722	2.78E-07	2	PVM
TANK	3.05E-06	0.81963813	0.537	0.361	0.065696969	2	PVM
AHR	1.46E-07	0.81651073	0.603	0.38	0.003154406	2	PVM
GLS	2.99E-08	0.81202201	0.618	0.405	0.000644813	2	PVM
JMY	4.66E-09	0.80939681	0.368	0.146	0.000100562	2	PVM
HEXIM1	0.000249	0.80884193	0.632	0.488	1	2	PVM
ZSWIM6	8.85E-09	0.80179888	0.559	0.328	0.000190983	2	PVM
NFKBIZ	4.17E-05	0.80014282	0.632	0.471	0.899300553	2	PVM
MAP2K3	5.12E-08	0.80001636	0.588	0.38	0.001104693	2	PVM
H2AFX	2.85E-07	0.79871351	0.537	0.331	0.006138495	2	PVM
AC007952.4	4.08E-09	0.79782539	0.478	0.218	8.79E-05	2	PVM
EIF5	7.68E-10	0.795702	0.919	0.807	1.66E-05	2	PVM
AMD1	1.63E-07	0.79541185	0.75	0.587	0.003518395	2	PVM
IER5	1.16E-07	0.78926756	0.75	0.576	0.002491731	2	PVM
FAM53C	1.76E-07	0.78466122	0.515	0.295	0.003789036	2	PVM
UBE2D1	1.73E-08	0.78439591	0.485	0.262	0.000372191	2	PVM
CD93	1.23E-07	0.77841277	0.368	0.16	0.002643549	2	PVM
WSB1	2.78E-10	0.7645196	0.853	0.694	6.00E-06	2	PVM
SLC38A2	7.59E-07	0.76063305	0.735	0.576	0.016378124	2	PVM
DYNC1H1	3.17E-06	0.76038184	0.713	0.565	0.068476593	2	PVM
ELL2	1.83E-06	0.75539345	0.647	0.424	0.039415997	2	PVM
STIP1	7.67E-07	0.74371721	0.471	0.278	0.016555338	2	PVM
RHOB	0.000186	0.74017381	0.787	0.639	1	2	PVM
HSPB1	3.18E-07	0.73967597	0.794	0.642	0.006849373	2	PVM
ELF1	3.64E-06	0.73957905	0.721	0.601	0.078508462	2	PVM
RBBP6	1.04E-07	0.73797691	0.699	0.537	0.002251192	2	PVM
PLAUR	3.60E-09	0.73644197	0.846	0.551	7.78E-05	2	PVM
PRNP	2.25E-08	0.73513548	0.713	0.529	0.000485262	2	PVM
PMAIP1	1.21E-07	0.73292228	0.625	0.394	0.002610312	2	PVM
RLF	2.28E-08	0.72918368	0.434	0.207	0.000491712	2	PVM
SLC3A2	1.86E-06	0.72280597	0.61	0.466	0.040109506	2	PVM
DUSP6	0.000629	0.7221043	0.574	0.466	1	2	PVM
ZC3H12C	4.44E-11	0.71780321	0.287	0.072	9.57E-07	2	PVM
DUSP5	5.19E-09	0.71652594	0.529	0.27	0.000112044	2	PVM
CREB5	7.12E-07	0.71320658	0.368	0.168	0.015349206	2	PVM
IRF2BP2	2.43E-07	0.71283143	0.676	0.466	0.005245818	2	PVM
PHACTR1	2.43E-05	0.70764687	0.581	0.435	0.523129277	2	PVM
NABP1	1.18E-05	0.69181411	0.647	0.463	0.253896121	2	PVM
OTUD1	0.001188	0.69039709	0.331	0.215	1	2	PVM
NAA50	3.99E-07	0.67350555	0.449	0.234	0.008615181	2	PVM
MYLIP	8.38E-08	0.67129573	0.559	0.317	0.001807994	2	PVM
ZFYVE16	7.94E-08	0.66995867	0.654	0.408	0.00171362	2	PVM
MAFF	0.000607	0.66940568	0.478	0.353	1	2	PVM
ATP13A3	4.24E-07	0.66484852	0.566	0.355	0.009141216	2	PVM
GADD45A	1.82E-07	0.66478674	0.419	0.209	0.003920842	2	PVM
IRS2	0.000385	0.65915522	0.581	0.444	1	2	PVM
DDX3X	4.64E-08	0.65755855	0.882	0.741	0.001000344	2	PVM
RUNX1	5.08E-06	0.65578045	0.574	0.394	0.109671711	2	PVM
MORF4L2	2.43E-05	0.65507902	0.566	0.427	0.524507459	2	PVM
NUFIP2	3.36E-05	0.65251917	0.787	0.62	0.724861215	2	PVM
NXF1	1.59E-05	0.65022297	0.588	0.394	0.342325225	2	PVM
ETF1	6.38E-09	0.65005683	0.699	0.477	0.00013759	2	PVM

ARIH1	2.85E-09	0.64968269	0.721	0.504	6.15E-05	2	PVM
AHSA1	1.20E-08	0.64900717	0.574	0.333	0.000259672	2	PVM
IFRD1	0.009213	0.64506535	0.581	0.477	1	2	PVM
GAS2L3	2.17E-07	0.63978753	0.324	0.135	0.004679296	2	PVM
YTHDF3	9.37E-09	0.63805602	0.449	0.218	0.0002021	2	PVM
IL1B	0.000565	0.6350776	0.331	0.19	1	2	PVM
NRP2	2.53E-07	0.63296658	0.574	0.339	0.005459991	2	PVM
SGK1	5.64E-06	0.63289157	0.801	0.656	0.121639131	2	PVM
RSRC2	1.06E-05	0.62996223	0.728	0.59	0.229325137	2	PVM
LCP2	5.12E-05	0.62650467	0.588	0.43	1	2	PVM
USP36	1.61E-06	0.62645286	0.463	0.256	0.034660024	2	PVM
GADD45G	0.006167	0.61620573	0.485	0.358	1	2	PVM
FAM133B	1.23E-06	0.61468711	0.787	0.601	0.026436352	2	PVM
DEDD2	7.61E-06	0.60990264	0.353	0.187	0.164086988	2	PVM
TSPYL2	0.007084	0.60983108	0.449	0.325	1	2	PVM
CBX4	4.05E-06	0.60744834	0.463	0.273	0.087393182	2	PVM
SRSF3	6.58E-06	0.60701976	0.904	0.793	0.141977841	2	PVM
PER1	0.000482	0.60685554	0.529	0.375	1	2	PVM
TUBA1C	0.000125	0.60349643	0.581	0.46	1	2	PVM
CEMIP2	0.000609	0.60220893	0.463	0.339	1	2	PVM
ZFAND2A	1.60E-05	0.60155403	0.596	0.399	0.345297058	2	PVM
AC016831.1	1.42E-06	0.60135234	0.338	0.154	0.03053638	2	PVM
CSGALNACT	1.02E-05	0.60087252	0.368	0.193	0.219653624	2	PVM
CACYBP	1.26E-05	0.60034466	0.632	0.482	0.271023598	2	PVM
SCML1	1.98E-06	0.59976565	0.537	0.314	0.042630039	2	PVM
DUSP2	0.001469	0.59944857	0.493	0.369	1	2	PVM
ZBTB10	1.25E-08	0.59607913	0.346	0.127	0.000269521	2	PVM
ICAM4	1.45E-09	0.59515196	0.213	0.041	3.13E-05	2	PVM
ETS2	2.18E-05	0.59199502	0.75	0.606	0.470618498	2	PVM
HOMER1	2.36E-05	0.59060043	0.191	0.069	0.509218379	2	PVM
EHD1	2.10E-06	0.58731192	0.368	0.182	0.045312926	2	PVM
NFATC1	4.36E-05	0.58729819	0.331	0.176	0.940437988	2	PVM
LONRF3	1.58E-14	0.58635567	0.272	0.039	3.41E-10	2	PVM
RAB21	2.79E-06	0.58603068	0.603	0.421	0.060261271	2	PVM
ZFAND5	5.97E-05	0.58510037	0.838	0.705	1	2	PVM
HSPA9	6.78E-07	0.5799538	0.625	0.449	0.014615366	2	PVM
IDI1	2.58E-06	0.57579995	0.647	0.463	0.055654717	2	PVM
CHKA	3.57E-06	0.57464265	0.412	0.229	0.076943263	2	PVM
YBX3	6.96E-05	0.57385852	0.801	0.686	1	2	PVM
KIF1B	2.73E-05	0.57234661	0.441	0.262	0.588664699	2	PVM
ANKRD28	0.000392	0.56965939	0.324	0.19	1	2	PVM
IL6R-AS1	4.60E-11	0.56714698	0.147	0.008	9.92E-07	2	PVM
OSER1	0.000118	0.56616214	0.471	0.311	1	2	PVM
CDC42EP3	4.96E-09	0.56595065	0.331	0.116	0.000106996	2	PVM
DNAJB4	3.16E-05	0.56519271	0.434	0.253	0.681651101	2	PVM
CNRIP1	5.21E-07	0.56352735	0.338	0.143	0.011239945	2	PVM
ANKRD37	0.000147	0.56316375	0.221	0.099	1	2	PVM
ALG13	0.00066	0.56163552	0.404	0.273	1	2	PVM
SLC16A10	0.001014	0.56126178	0.191	0.088	1	2	PVM
SAV1	2.86E-07	0.5531873	0.331	0.132	0.006171524	2	PVM
FKBP4	0.000937	0.54886795	0.412	0.295	1	2	PVM
PDPK1	9.23E-07	0.54477018	0.419	0.22	0.019906977	2	PVM

NFAT5	2.72E-06	0.54168856	0.456	0.242	0.058782436	2	PVM
TSC22D2	1.31E-05	0.53893628	0.456	0.275	0.282990199	2	PVM
RDH10	8.46E-05	0.53781504	0.199	0.08	1	2	PVM
ERF	1.78E-05	0.53757413	0.221	0.085	0.38392298	2	PVM
DNAJC8	0.000158	0.53658905	0.581	0.446	1	2	PVM
PPIF	6.59E-07	0.53637449	0.346	0.157	0.01421845	2	PVM
IL6ST	0.004371	0.53112293	0.456	0.353	1	2	PVM
TXNRD1	0.00457	0.52888315	0.39	0.267	1	2	PVM
DOT1L	6.85E-09	0.52464196	0.257	0.069	0.000147805	2	PVM
GLA	0.000237	0.52191487	0.375	0.229	1	2	PVM
BMP2	8.18E-06	0.51784723	0.176	0.052	0.176530741	2	PVM
GCLM	0.007821	0.5178212	0.441	0.336	1	2	PVM
MAPKAPK2	1.02E-05	0.51608535	0.419	0.24	0.220477775	2	PVM
FSCN1	6.10E-06	0.51273904	0.36	0.176	0.131679794	2	PVM
BCAS2	0.002457	0.51208541	0.426	0.306	1	2	PVM
SRSF6	0.000684	0.50942053	0.522	0.375	1	2	PVM
KCNQ1OT1	0.00254	0.50838996	0.522	0.399	1	2	PVM
BACH1	1.93E-05	0.50752215	0.515	0.333	0.416286287	2	PVM
PDE4DIP	0.000679	0.50680643	0.449	0.322	1	2	PVM
IVNS1ABP	0.000191	0.50577533	0.596	0.438	1	2	PVM
FOXK2	0.001496	0.50534124	0.228	0.121	1	2	PVM
ATP6V0D1	0.000171	0.50060813	0.61	0.499	1	2	PVM
RNF138	0.000176	0.49739719	0.257	0.127	1	2	PVM
ARL8B	0.000232	0.49700932	0.449	0.328	1	2	PVM
KHDRBS1	0.000276	0.49660491	0.566	0.427	1	2	PVM
TCEAL9	7.77E-05	0.49551934	0.346	0.204	1	2	PVM
CDKN2AIP	1.21E-07	0.49411191	0.434	0.204	0.002617536	2	PVM
ARHGAP5	6.68E-05	0.49372919	0.61	0.413	1	2	PVM
AFF1	0.000254	0.49325029	0.404	0.253	1	2	PVM
SLC25A33	9.71E-08	0.49319074	0.426	0.19	0.002095384	2	PVM
SH2B3	3.08E-05	0.49281269	0.625	0.463	0.664644717	2	PVM
DDX18	0.004201	0.49045172	0.551	0.446	1	2	PVM
IER5L	0.001473	0.48726776	0.5	0.369	1	2	PVM
MAFG	9.24E-05	0.48107195	0.5	0.353	1	2	PVM
SLC20A1	0.002684	0.48078572	0.529	0.402	1	2	PVM
ERN1	1.83E-07	0.47945019	0.375	0.165	0.003957499	2	PVM
SLC1A5	0.00011	0.4791212	0.346	0.193	1	2	PVM
DHX36	0.002878	0.47559386	0.493	0.383	1	2	PVM
AZIN1	0.000969	0.47398911	0.463	0.328	1	2	PVM
TBC1D15	0.000258	0.47365859	0.404	0.24	1	2	PVM
INTS6	0.008394	0.47364628	0.743	0.634	1	2	PVM
EZH2	0.000184	0.47358336	0.235	0.11	1	2	PVM
SON	1.79E-07	0.47206592	0.919	0.807	0.003861562	2	PVM
NRIP1	5.44E-05	0.47136564	0.493	0.306	1	2	PVM
CFLAR	0.005813	0.47101429	0.662	0.548	1	2	PVM
RRP12	2.69E-05	0.46921469	0.162	0.05	0.579211941	2	PVM
HSPA1L	3.88E-07	0.46433818	0.213	0.061	0.008376282	2	PVM
LUCAT1	1.10E-07	0.46410195	0.228	0.063	0.002368932	2	PVM
ABCA1	0.004969	0.46328608	0.61	0.507	1	2	PVM
C16orf72	0.000684	0.46320977	0.574	0.419	1	2	PVM
ODC1	0.001023	0.46272831	0.316	0.196	1	2	PVM
GABPB1-AS1	0.000457	0.46241404	0.235	0.116	1	2	PVM

CYLD	0.000172	0.46179575	0.5	0.35	1	2	PVM
NUDT4	0.000695	0.46144886	0.404	0.262	1	2	PVM
KANSL1L	6.39E-06	0.45920049	0.257	0.099	0.137763058	2	PVM
TOB1	0.002003	0.45916853	0.478	0.353	1	2	PVM
FUS	1.07E-05	0.45849755	0.919	0.802	0.230196793	2	PVM
DENND4A	2.37E-05	0.45673611	0.419	0.231	0.512311593	2	PVM
ARFGAP3	0.004754	0.45524112	0.375	0.267	1	2	PVM
TRIM28	0.000126	0.45489461	0.434	0.281	1	2	PVM
NUP58	0.008381	0.45385412	0.353	0.248	1	2	PVM
CDKN1C	0.001686	0.45380592	0.368	0.231	1	2	PVM
LGMN	8.84E-06	0.4528581	0.816	0.68	0.190727196	2	PVM
NKRF	0.001768	0.45195452	0.228	0.121	1	2	PVM
ZNF503	2.90E-06	0.45021095	0.39	0.187	0.062650147	2	PVM
WDR26	0.00108	0.44959833	0.471	0.342	1	2	PVM
AC092803.2	2.49E-07	0.44891989	0.169	0.036	0.005372365	2	PVM
TARS	0.000213	0.44818699	0.272	0.138	1	2	PVM
EIF2AK3	5.10E-05	0.44801208	0.279	0.135	1	2	PVM
NAMPT	0.004138	0.44795939	0.816	0.711	1	2	PVM
PDXDC1	0.000645	0.4477283	0.294	0.168	1	2	PVM
CREM	0.001318	0.44653784	0.743	0.576	1	2	PVM
ANKDD1A	1.74E-07	0.44617153	0.338	0.132	0.003748096	2	PVM
FBXO11	0.003973	0.44476125	0.441	0.333	1	2	PVM
ATP2A2	0.000133	0.4417548	0.5	0.355	1	2	PVM
PPP3R1	2.36E-05	0.44156699	0.368	0.193	0.508263846	2	PVM
MPDU1	0.000275	0.44143136	0.434	0.292	1	2	PVM
JMJD6	0.000163	0.44140462	0.478	0.314	1	2	PVM
NFKBIB	0.00084	0.44108357	0.279	0.16	1	2	PVM
STMN1	0.000301	0.44042993	0.632	0.482	1	2	PVM
MGAT1	0.00011	0.43991873	0.728	0.576	1	2	PVM
AKAP13	0.000363	0.43549914	0.89	0.785	1	2	PVM
INSIG1	0.000467	0.4339765	0.647	0.471	1	2	PVM
HSPA4	0.00028	0.43271353	0.434	0.287	1	2	PVM
SRSF2	0.000569	0.43003091	0.75	0.647	1	2	PVM
SH3BP5	0.000485	0.42599512	0.684	0.496	1	2	PVM
HIF1A	0.009844	0.42573073	0.574	0.468	1	2	PVM
ING1	4.61E-06	0.4234024	0.412	0.215	0.099509546	2	PVM
ZNF703	1.63E-08	0.42311257	0.316	0.102	0.000351346	2	PVM
YTHDC1	0.00441	0.42157867	0.449	0.344	1	2	PVM
WAC	0.000705	0.42150853	0.581	0.441	1	2	PVM
PPP2CA	0.000386	0.42099201	0.456	0.314	1	2	PVM
HNRNPM	0.001704	0.42029429	0.574	0.463	1	2	PVM
TNF	0.001557	0.4127057	0.279	0.157	1	2	PVM
C3orf58	0.000328	0.4123302	0.404	0.253	1	2	PVM
AC016831.5	0.000234	0.40724814	0.36	0.204	1	2	PVM
EIF4G2	3.61E-06	0.40643009	0.846	0.708	0.077816528	2	PVM
KLF7	0.006071	0.40552249	0.36	0.248	1	2	PVM
IPO7	0.000894	0.40490091	0.412	0.284	1	2	PVM
ADAM17	0.00234	0.40439794	0.522	0.391	1	2	PVM
WTAP	0.000915	0.40199743	0.507	0.361	1	2	PVM
SRSF1	0.00022	0.40189095	0.404	0.251	1	2	PVM
TIPARP	2.76E-05	0.40152527	0.493	0.306	0.595904	2	PVM
TENT4B	0.000563	0.40088291	0.309	0.168	1	2	PVM

FAM20A	0.000924	0.40032346	0.331	0.196	1	2	PVM
SLC45A4	7.50E-06	0.39687602	0.228	0.083	0.1617936	2	PVM
TMED5	0.002982	0.39140096	0.478	0.369	1	2	PVM
ADNP2	0.000196	0.38743405	0.316	0.171	1	2	PVM
MAFK	8.82E-05	0.38248645	0.213	0.085	1	2	PVM
EPAS1	0.002533	0.3820396	0.272	0.168	1	2	PVM
KLHL21	2.87E-06	0.38133272	0.191	0.055	0.061857363	2	PVM
SNHG7	0.001529	0.37748807	0.404	0.273	1	2	PVM
PLAU	0.003977	0.37739267	0.221	0.121	1	2	PVM
CRY1	1.16E-06	0.37718111	0.213	0.066	0.024927751	2	PVM
PHLDA1	0.009723	0.37621	0.397	0.284	1	2	PVM
LATS2	4.10E-05	0.37614622	0.36	0.182	0.884922564	2	PVM
CLN8	9.38E-05	0.37585398	0.434	0.245	1	2	PVM
WDR45B	0.009408	0.37409414	0.338	0.229	1	2	PVM
BRAF	0.00184	0.37160601	0.279	0.165	1	2	PVM
MAPK6	0.004695	0.37139591	0.294	0.187	1	2	PVM
GTF2H1	0.002692	0.3687718	0.272	0.165	1	2	PVM
LYVE1	9.51E-05	0.36757352	0.831	0.647	1	2	PVM
HMGA1	0.004754	0.3669726	0.324	0.212	1	2	PVM
ARL5B	0.004849	0.36527885	0.235	0.132	1	2	PVM
TOR1AIP1	0.001065	0.36388405	0.316	0.187	1	2	PVM
ZC3H12A	0.001325	0.36348145	0.301	0.174	1	2	PVM
HES1	0.000122	0.36293468	0.368	0.198	1	2	PVM
MAPRE1	0.00093	0.36281565	0.529	0.397	1	2	PVM
CCNYL1	5.31E-05	0.36064479	0.265	0.121	1	2	PVM
ARID1A	0.001412	0.3602578	0.36	0.223	1	2	PVM
MRFAP1	0.006574	0.35966158	0.507	0.399	1	2	PVM
YES1	2.82E-05	0.35900493	0.14	0.036	0.607354969	2	PVM
TCERG1	0.008063	0.35387849	0.272	0.168	1	2	PVM
PXDC1	0.000183	0.35237399	0.36	0.212	1	2	PVM
MIR222HG	3.33E-05	0.34852425	0.206	0.074	0.71851388	2	PVM
FAM210A	0.002426	0.34774742	0.243	0.135	1	2	PVM
EPB41L1	0.000228	0.34730615	0.206	0.085	1	2	PVM
MBNL2	0.003101	0.3463069	0.331	0.207	1	2	PVM
PIP4P1	4.21E-05	0.34557048	0.316	0.16	0.907257541	2	PVM
FRMD4B	0.006399	0.34542439	0.691	0.567	1	2	PVM
SEMA4A	0.0003	0.34533724	0.39	0.226	1	2	PVM
PURB	0.007032	0.34398815	0.375	0.253	1	2	PVM
ETV3	2.38E-05	0.34302256	0.316	0.149	0.512537487	2	PVM
RHOBTB3	0.000388	0.34270912	0.279	0.149	1	2	PVM
SKI	0.000312	0.34231913	0.324	0.174	1	2	PVM
PLCXD1	4.13E-05	0.34210111	0.206	0.077	0.890847842	2	PVM
AC135050.1	2.50E-05	0.33928662	0.162	0.047	0.539256419	2	PVM
ST3GAL6	0.000736	0.33535364	0.221	0.107	1	2	PVM
SPART	0.00087	0.33527888	0.36	0.229	1	2	PVM
ARMCX1	0.00072	0.33483745	0.213	0.099	1	2	PVM
KBTBD8	3.76E-05	0.33400695	0.176	0.061	0.811773587	2	PVM
PNPLA8	0.005785	0.33356367	0.493	0.377	1	2	PVM
BCAT1	0.002818	0.33220405	0.463	0.344	1	2	PVM
NRIP3	3.02E-06	0.33082141	0.199	0.061	0.065133136	2	PVM
P2RY14	0.002308	0.33026364	0.419	0.278	1	2	PVM
FGFR1	1.60E-05	0.327242	0.36	0.171	0.34577818	2	PVM

IFITM10	1.69E-09	0.32437651	0.154	0.017	3.64E-05	2	PVM
PLEKHG2	0.002834	0.32395697	0.294	0.182	1	2	PVM
COQ10B	0.0019	0.32355844	0.331	0.207	1	2	PVM
NCF4	0.000586	0.32178834	0.603	0.438	1	2	PVM
PITPNB	0.005018	0.31728979	0.294	0.185	1	2	PVM
GOLGB1	0.002681	0.31648426	0.662	0.537	1	2	PVM
KPNA4	0.005016	0.31481729	0.596	0.485	1	2	PVM
DYNLL2	0.001108	0.31394739	0.338	0.212	1	2	PVM
DDX6	0.00175	0.31276488	0.419	0.273	1	2	PVM
PKD1	3.62E-05	0.31230351	0.162	0.05	0.780705172	2	PVM
SERTAD3	0.005696	0.30859973	0.265	0.163	1	2	PVM
REV3L	0.000129	0.30287262	0.419	0.248	1	2	PVM
RCOR3	0.000267	0.30115322	0.265	0.127	1	2	PVM
CHML	0.002968	0.30042748	0.265	0.146	1	2	PVM
RIPK1	0.001541	0.29821621	0.206	0.102	1	2	PVM
UGCG	0.006309	0.29783583	0.309	0.198	1	2	PVM
HDLBP	0.002059	0.29723636	0.493	0.353	1	2	PVM
CXCL12	7.78E-06	0.29691835	0.397	0.185	0.16783289	2	PVM
PUM1	0.00867	0.2945912	0.441	0.333	1	2	PVM
LILRB5	4.70E-05	0.29450651	0.669	0.452	1	2	PVM
BAIAP2	0.002846	0.29155682	0.375	0.242	1	2	PVM
RFX1	0.000774	0.29103996	0.265	0.14	1	2	PVM
CD209	0.005946	0.2905011	0.324	0.201	1	2	PVM
PLEKHM2	0.004798	0.29027746	0.36	0.245	1	2	PVM
ARMC5	0.000579	0.28985452	0.176	0.074	1	2	PVM
RGL1	0.004925	0.28968347	0.537	0.383	1	2	PVM
UBAP2L	0.001975	0.28926496	0.338	0.209	1	2	PVM
EFNB2	2.02E-06	0.2888329	0.125	0.022	0.043587417	2	PVM
STX3	0.000651	0.28688212	0.257	0.129	1	2	PVM
CRHBP	4.37E-06	0.28628402	0.162	0.041	0.094210736	2	PVM
GAB1	1.77E-05	0.28515843	0.169	0.05	0.380787793	2	PVM
KLF11	0.000679	0.28506154	0.316	0.174	1	2	PVM
PER2	0.00095	0.28442627	0.221	0.107	1	2	PVM
ABCA6	8.91E-05	0.28348653	0.537	0.325	1	2	PVM
TUFT1	0.000139	0.28144289	0.169	0.061	1	2	PVM
HIVEP2	0.000955	0.27938644	0.213	0.102	1	2	PVM
NRP1	0.000314	0.27851985	0.625	0.427	1	2	PVM
DDX39A	0.003626	0.27847502	0.397	0.27	1	2	PVM
SGMS1	0.004786	0.27819172	0.397	0.273	1	2	PVM
FAM13A	0.006566	0.27647451	0.449	0.317	1	2	PVM
ATN1	0.000205	0.27561158	0.228	0.099	1	2	PVM
GNS	0.008284	0.27331745	0.5	0.386	1	2	PVM
TNFAIP2	0.001064	0.27219177	0.721	0.576	1	2	PVM
ATP6V1H	0.002332	0.2718465	0.279	0.163	1	2	PVM
SLC9A1	4.12E-06	0.2702754	0.184	0.052	0.088854384	2	PVM
PLA2G2A	5.25E-05	0.27005853	0.154	0.047	1	2	PVM
SPATS2L	0.007841	0.26559347	0.522	0.397	1	2	PVM
FOXO1	0.002846	0.26449038	0.228	0.124	1	2	PVM
IPMK	0.003113	0.261089	0.265	0.152	1	2	PVM
MFHAS1	0.000212	0.26058433	0.272	0.127	1	2	PVM
CFAP20	4.74E-05	0.26008645	0.206	0.077	1	2	PVM
PKNOX1	0.001796	0.25852221	0.338	0.198	1	2	PVM

TNFRSF25	5.58E-05	0.25697015	0.191	0.066	1	2	PVM
FOXO3	0.001175	0.25396482	0.61	0.457	1	2	PVM
SELENOK	0.008971	0.25179194	0.676	0.562	1	2	PVM
Z82244.2	1.02E-05	0.25076707	0.191	0.061	0.219061197	2	PVM
AREG	1.59E-64	3.83477677	0.846	0.099	3.43E-60	3	NPVM
HLA-DRB5	5.07E-39	2.39885673	0.816	0.237	1.09E-34	3	NPVM
IL1R2	3.47E-45	2.12079049	0.566	0.033	7.48E-41	3	NPVM
HLA-DQA1	2.85E-39	1.99866543	0.971	0.678	6.15E-35	3	NPVM
FCER1A	4.38E-31	1.98193095	0.441	0.036	9.45E-27	3	NPVM
EZR	3.66E-40	1.96924685	0.897	0.438	7.90E-36	3	NPVM
THBS1	7.37E-40	1.95974804	0.735	0.118	1.59E-35	3	NPVM
LYZ	2.55E-31	1.89220669	0.949	0.694	5.50E-27	3	NPVM
CD1C	1.30E-33	1.88298667	0.449	0.03	2.81E-29	3	NPVM
FCN1	2.66E-32	1.80821542	0.596	0.099	5.74E-28	3	NPVM
GRASP	2.11E-47	1.80031823	0.868	0.198	4.55E-43	3	NPVM
SLC2A3	6.34E-41	1.6932694	0.831	0.207	1.37E-36	3	NPVM
SYAP1	1.28E-30	1.63040591	0.809	0.311	2.77E-26	3	NPVM
RILPL2	2.32E-33	1.57812926	0.838	0.355	5.01E-29	3	NPVM
TIMP1	2.32E-19	1.56699223	0.919	0.625	5.01E-15	3	NPVM
PKP2	5.99E-46	1.56419604	0.61	0.039	1.29E-41	3	NPVM
ATP1B3	2.10E-30	1.51630276	0.919	0.562	4.52E-26	3	NPVM
HLA-DQB1	5.40E-35	1.45504206	0.985	0.791	1.16E-30	3	NPVM
MT2A	2.85E-24	1.39222994	0.985	0.865	6.16E-20	3	NPVM
ITGAX	6.64E-30	1.36535249	0.75	0.245	1.43E-25	3	NPVM
SDS	6.36E-15	1.35459734	0.397	0.113	1.37E-10	3	NPVM
SRGN	5.69E-35	1.32538229	1	0.893	1.23E-30	3	NPVM
MT1M	8.33E-08	1.32284687	0.39	0.176	0.001797714	3	NPVM
CRYBG1	1.19E-23	1.32077137	0.5	0.11	2.57E-19	3	NPVM
SAMSN1	6.93E-29	1.32023833	0.875	0.397	1.50E-24	3	NPVM
GPAT3	1.03E-31	1.32005609	0.551	0.08	2.21E-27	3	NPVM
METRNL	1.26E-35	1.31117685	0.941	0.556	2.72E-31	3	NPVM
PLSCR1	6.08E-30	1.29948343	0.809	0.342	1.31E-25	3	NPVM
VDR	2.74E-36	1.28013742	0.515	0.044	5.92E-32	3	NPVM
CFP	2.61E-24	1.26063515	0.625	0.19	5.64E-20	3	NPVM
EREG	2.26E-21	1.21991382	0.324	0.028	4.86E-17	3	NPVM
ISG20	7.69E-27	1.20528091	0.529	0.099	1.66E-22	3	NPVM
VEGFA	1.10E-27	1.2008878	0.853	0.336	2.38E-23	3	NPVM
LGALS2	3.45E-21	1.19105872	0.441	0.083	7.45E-17	3	NPVM
TYMP	9.33E-23	1.14644022	0.919	0.554	2.01E-18	3	NPVM
FOSL2	2.47E-29	1.0967872	0.728	0.198	5.33E-25	3	NPVM
SAP30	3.20E-14	1.09516063	0.485	0.179	6.90E-10	3	NPVM
PLAUR1	6.75E-23	1.09092384	0.897	0.532	1.46E-18	3	NPVM
CD86	1.32E-19	1.08896738	0.757	0.416	2.85E-15	3	NPVM
MXD1	2.59E-19	1.0882937	0.669	0.278	5.59E-15	3	NPVM
ZNF331	2.31E-18	1.07301118	0.721	0.32	4.99E-14	3	NPVM
RGCC	2.88E-16	1.06789443	0.794	0.435	6.21E-12	3	NPVM
JARID2	6.91E-17	1.06560434	0.588	0.229	1.49E-12	3	NPVM
C1orf162	4.01E-22	1.04984277	0.897	0.601	8.65E-18	3	NPVM
INSIG11	1.74E-15	1.04675759	0.765	0.427	3.75E-11	3	NPVM
BTG1	1.24E-22	1.02620718	0.993	0.796	2.67E-18	3	NPVM
CRIP1	2.24E-11	1.02156466	0.625	0.333	4.82E-07	3	NPVM
PKIB	3.08E-20	1.01645331	0.441	0.091	6.64E-16	3	NPVM

CKLF	6.98E-15	1.01549362	0.691	0.408	1.51E-10	3	NPVM
FGD4	1.19E-17	1.00999833	0.721	0.331	2.56E-13	3	NPVM
CLEC10A	1.29E-17	1.00955701	0.853	0.581	2.78E-13	3	NPVM
CREM1	1.47E-20	0.99612596	0.904	0.515	3.17E-16	3	NPVM
RGS1	1.69E-13	0.97844977	0.551	0.212	3.65E-09	3	NPVM
COTL1	7.68E-19	0.97705732	0.882	0.537	1.66E-14	3	NPVM
RPS26	6.31E-24	0.97468036	0.971	0.716	1.36E-19	3	NPVM
TREM1	3.31E-21	0.97162516	0.485	0.105	7.13E-17	3	NPVM
SNHG15	1.97E-15	0.96334648	0.478	0.16	4.25E-11	3	NPVM
VCAN	3.03E-09	0.9601769	0.294	0.091	6.54E-05	3	NPVM
IL1R1	5.68E-21	0.92188031	0.441	0.08	1.23E-16	3	NPVM
NAMPT1	1.93E-24	0.92076063	0.971	0.653	4.16E-20	3	NPVM
CD300E	2.35E-26	0.91693949	0.36	0.022	5.07E-22	3	NPVM
FGL2	4.37E-21	0.91474927	0.949	0.73	9.43E-17	3	NPVM
PPP1CB	1.46E-18	0.91227126	0.846	0.518	3.14E-14	3	NPVM
DBI	3.33E-13	0.90220682	0.875	0.642	7.18E-09	3	NPVM
MIR22HG	1.43E-17	0.89379853	0.743	0.339	3.08E-13	3	NPVM
CD52	1.34E-13	0.89208327	0.478	0.16	2.89E-09	3	NPVM
MAP3K8	5.25E-13	0.88573637	0.684	0.386	1.13E-08	3	NPVM
ACSL3	1.40E-13	0.88454449	0.61	0.298	3.03E-09	3	NPVM
ADGRE5	3.36E-10	0.87967459	0.64	0.394	7.25E-06	3	NPVM
CD48	1.30E-15	0.86955996	0.493	0.174	2.80E-11	3	NPVM
RGS2	1.60E-18	0.86824202	0.868	0.496	3.45E-14	3	NPVM
S100A10	1.17E-11	0.86767894	0.949	0.791	2.51E-07	3	NPVM
PHLDA11	7.45E-12	0.86020206	0.544	0.229	1.61E-07	3	NPVM
CAVIN2	2.63E-18	0.85517869	0.353	0.052	5.67E-14	3	NPVM
MAT2A	3.59E-19	0.85097604	0.904	0.529	7.74E-15	3	NPVM
UPP1	1.52E-12	0.84634276	0.537	0.242	3.29E-08	3	NPVM
IRF7	8.45E-12	0.8444683	0.375	0.124	1.82E-07	3	NPVM
APOBEC3A	1.24E-13	0.84439189	0.169	0.006	2.67E-09	3	NPVM
DUSP4	1.18E-23	0.84436373	0.404	0.047	2.54E-19	3	NPVM
AGO2	1.39E-13	0.83101862	0.493	0.176	3.01E-09	3	NPVM
STK17B	7.66E-19	0.8289831	0.824	0.455	1.65E-14	3	NPVM
PHLDA2	4.22E-13	0.82732428	0.412	0.129	9.10E-09	3	NPVM
ACSL1	4.61E-13	0.8253928	0.662	0.339	9.95E-09	3	NPVM
TRMT6	8.54E-15	0.82486696	0.353	0.083	1.84E-10	3	NPVM
CLEC4E	1.71E-09	0.81924474	0.419	0.174	3.68E-05	3	NPVM
JAML	8.36E-15	0.81064054	0.654	0.306	1.80E-10	3	NPVM
CD44	7.75E-15	0.79607637	0.868	0.576	1.67E-10	3	NPVM
FLT1	6.37E-17	0.79103811	0.309	0.044	1.37E-12	3	NPVM
NLRP3	1.13E-18	0.78021253	0.684	0.267	2.43E-14	3	NPVM
SELENOK1	5.66E-13	0.77999372	0.779	0.523	1.22E-08	3	NPVM
SLC31A2	2.44E-12	0.77746441	0.603	0.311	5.26E-08	3	NPVM
MT1X	5.01E-08	0.77538341	0.868	0.713	0.001081015	3	NPVM
LMNA	1.30E-16	0.77469602	0.949	0.744	2.80E-12	3	NPVM
NFIL3	2.76E-17	0.7686402	0.493	0.14	5.95E-13	3	NPVM
CSTA	8.38E-16	0.76236805	0.529	0.19	1.81E-11	3	NPVM
CYTIP	9.40E-13	0.75939039	0.39	0.127	2.03E-08	3	NPVM
G0S2	1.38E-05	0.75494037	0.279	0.121	0.297242307	3	NPVM
SLC25A37	1.26E-11	0.75403415	0.434	0.16	2.72E-07	3	NPVM
SERPINB9	9.65E-13	0.75366932	0.559	0.242	2.08E-08	3	NPVM
GABARAPL1	3.80E-12	0.74821714	0.544	0.242	8.20E-08	3	NPVM

BASP1	8.54E-09	0.73307484	0.596	0.35	0.000184166	3	NPVM
RNF149	1.66E-14	0.73091355	0.728	0.402	3.57E-10	3	NPVM
PDE4A	2.33E-14	0.72893401	0.471	0.152	5.02E-10	3	NPVM
SGK11	4.18E-15	0.72683936	0.926	0.609	9.02E-11	3	NPVM
SH3BP51	9.12E-13	0.72180977	0.765	0.466	1.97E-08	3	NPVM
PDE4B	5.50E-14	0.71599377	0.529	0.179	1.19E-09	3	NPVM
AC058791.1	2.37E-12	0.71297006	0.346	0.091	5.11E-08	3	NPVM
DDIT3	1.42E-08	0.70776705	0.713	0.46	0.000306932	3	NPVM
CSF2RA	1.72E-11	0.70682917	0.544	0.245	3.71E-07	3	NPVM
TES	4.49E-13	0.70675525	0.441	0.154	9.69E-09	3	NPVM
C15orf48	3.16E-08	0.70328561	0.287	0.099	0.000681892	3	NPVM
CXCR4	9.05E-07	0.70149367	0.507	0.3	0.019513351	3	NPVM
C5AR1	3.34E-06	0.6992282	0.721	0.62	0.072026162	3	NPVM
B4GALT1	4.00E-12	0.69770186	0.684	0.377	8.63E-08	3	NPVM
CST7	3.51E-21	0.69768467	0.272	0.011	7.57E-17	3	NPVM
LST1	7.67E-13	0.69430157	0.846	0.529	1.65E-08	3	NPVM
PIM3	5.48E-07	0.69416775	0.5	0.281	0.011819384	3	NPVM
DUSP21	2.84E-08	0.69309688	0.603	0.328	0.000612343	3	NPVM
PDCL3	9.13E-10	0.68735636	0.426	0.187	1.97E-05	3	NPVM
MT1E	3.64E-09	0.68539216	0.75	0.474	7.85E-05	3	NPVM
PPA1	6.45E-08	0.68376963	0.353	0.152	0.001391606	3	NPVM
CPVL	2.50E-09	0.68038047	0.824	0.623	5.39E-05	3	NPVM
NDRG1	1.90E-06	0.67656462	0.39	0.212	0.041079387	3	NPVM
LIMS1	1.50E-10	0.67036533	0.647	0.355	3.24E-06	3	NPVM
CLEC7A	1.01E-07	0.66994698	0.522	0.295	0.002184781	3	NPVM
LDHA	1.33E-09	0.66919565	0.654	0.41	2.87E-05	3	NPVM
NAP1L1	8.26E-10	0.66648615	0.882	0.653	1.78E-05	3	NPVM
SOCS3	1.64E-12	0.66424223	0.743	0.383	3.54E-08	3	NPVM
CD1E	2.17E-12	0.66151121	0.162	0.008	4.69E-08	3	NPVM
VMO1	1.81E-07	0.65657755	0.485	0.245	0.003903539	3	NPVM
SLC30A1	8.09E-08	0.65563576	0.551	0.325	0.001745195	3	NPVM
FLNA	9.66E-10	0.65548027	0.581	0.292	2.08E-05	3	NPVM
MAP2K1	6.91E-14	0.65416316	0.61	0.292	1.49E-09	3	NPVM
SATB1	1.31E-09	0.64812548	0.441	0.193	2.83E-05	3	NPVM
ISG15	3.70E-06	0.64597349	0.441	0.251	0.079726198	3	NPVM
RAB31	1.46E-10	0.64406027	0.728	0.49	3.14E-06	3	NPVM
AVPI1	9.49E-14	0.64375993	0.382	0.107	2.05E-09	3	NPVM
SERP1	5.56E-10	0.63818432	0.816	0.617	1.20E-05	3	NPVM
SARAF	3.21E-12	0.63546755	0.868	0.65	6.93E-08	3	NPVM
SYTL3	9.00E-13	0.63211454	0.257	0.044	1.94E-08	3	NPVM
IRF1	1.79E-08	0.63195251	0.684	0.441	0.000386304	3	NPVM
IL1B1	2.30E-06	0.62917342	0.382	0.171	0.04956064	3	NPVM
COMMD6	1.90E-11	0.62549288	0.853	0.642	4.09E-07	3	NPVM
INSR	1.14E-11	0.61816425	0.353	0.096	2.46E-07	3	NPVM
AP1S2	7.14E-08	0.61645883	0.757	0.54	0.001541211	3	NPVM
PSME1	1.41E-09	0.6150622	0.75	0.46	3.04E-05	3	NPVM
IFITM2	8.58E-09	0.6078706	0.743	0.562	0.000185149	3	NPVM
SIPA1L1	2.73E-11	0.60439689	0.515	0.209	5.90E-07	3	NPVM
CXCL16	5.28E-10	0.60135963	0.824	0.579	1.14E-05	3	NPVM
SERPINA1	2.36E-08	0.60031898	0.419	0.19	0.000509826	3	NPVM
CD55	1.28E-07	0.59917772	0.684	0.474	0.002765985	3	NPVM
ADA2	2.27E-09	0.59472303	0.632	0.339	4.89E-05	3	NPVM

FILIP1L	0.00021	0.59468053	0.559	0.405	1	3	NPVM
IFITM3	2.22E-07	0.59407446	0.824	0.713	0.004792352	3	NPVM
HLA-F	1.35E-08	0.59073374	0.515	0.27	0.000291635	3	NPVM
RIPK2	3.01E-11	0.58865152	0.449	0.176	6.49E-07	3	NPVM
ARL4A	3.90E-05	0.58778279	0.581	0.388	0.840244365	3	NPVM
SULF2	4.72E-13	0.58751817	0.346	0.085	1.02E-08	3	NPVM
TNFSF13B	5.54E-06	0.58574079	0.515	0.325	0.119602299	3	NPVM
IRAK3	2.55E-13	0.58434488	0.618	0.262	5.51E-09	3	NPVM
GPR1831	5.45E-16	0.58416759	0.904	0.532	1.18E-11	3	NPVM
JOSD1	1.18E-08	0.57950935	0.588	0.32	0.000255508	3	NPVM
SLC25A6	2.62E-10	0.57946046	0.86	0.667	5.66E-06	3	NPVM
FPR1	1.09E-07	0.57651729	0.36	0.154	0.002348496	3	NPVM
AL138899.1	2.14E-15	0.57190988	0.287	0.041	4.61E-11	3	NPVM
CTSH	2.77E-07	0.56990603	0.772	0.521	0.005978352	3	NPVM
RBM3	1.45E-08	0.56790831	0.772	0.57	0.000312813	3	NPVM
LSP1	1.09E-10	0.56704734	0.522	0.22	2.35E-06	3	NPVM
SMAP2	9.77E-10	0.56326297	0.787	0.573	2.11E-05	3	NPVM
RPS5	3.04E-11	0.5632116	0.963	0.829	6.55E-07	3	NPVM
CHRNE	2.65E-19	0.56277544	0.272	0.017	5.72E-15	3	NPVM
FGR	2.89E-07	0.56262998	0.368	0.168	0.006230505	3	NPVM
MPHOSPH6	4.79E-09	0.56137245	0.382	0.154	0.00010325	3	NPVM
DNAAF1	3.40E-11	0.5574992	0.14	0.006	7.33E-07	3	NPVM
CD72	9.94E-12	0.55716243	0.397	0.129	2.14E-07	3	NPVM
AFMID	8.49E-15	0.55687509	0.309	0.052	1.83E-10	3	NPVM
GK	9.05E-09	0.55622366	0.382	0.149	0.000195282	3	NPVM
GPAT4	3.60E-10	0.55382283	0.382	0.14	7.77E-06	3	NPVM
CHD1	1.34E-07	0.55234345	0.691	0.446	0.002896389	3	NPVM
CHMP1B	1.49E-06	0.54925837	0.618	0.399	0.032209731	3	NPVM
POMP	7.88E-09	0.54804563	0.831	0.634	0.000169982	3	NPVM
DIAPH1	1.31E-06	0.54801953	0.382	0.193	0.028351667	3	NPVM
RAD51C	3.59E-11	0.54736068	0.316	0.088	7.74E-07	3	NPVM
PTK2B	2.54E-08	0.54590994	0.382	0.163	0.000548767	3	NPVM
MIS18BP1	0.000152	0.54525215	0.551	0.397	1	3	NPVM
CTLA4	8.61E-13	0.54503159	0.176	0.011	1.86E-08	3	NPVM
PABPC4	6.24E-07	0.5446133	0.787	0.612	0.013457057	3	NPVM
SH3BGRL3	2.37E-12	0.54274111	0.971	0.81	5.12E-08	3	NPVM
RNF7	2.84E-07	0.53972732	0.581	0.386	0.006125736	3	NPVM
ATP13A31	5.57E-06	0.53775352	0.574	0.353	0.12026162	3	NPVM
EIF3K	2.90E-08	0.53513161	0.801	0.595	0.000626067	3	NPVM
PSMB9	1.58E-06	0.53487157	0.493	0.292	0.033978926	3	NPVM
PSME2	1.93E-05	0.53069789	0.507	0.331	0.415273366	3	NPVM
MALT1	9.10E-12	0.53053839	0.522	0.209	1.96E-07	3	NPVM
TPM4	1.77E-07	0.53020417	0.75	0.521	0.003814002	3	NPVM
PHF20	7.08E-09	0.52880021	0.596	0.344	0.000152635	3	NPVM
RPL36A	3.72E-09	0.52499662	0.912	0.752	8.02E-05	3	NPVM
OXSR1	0.00023	0.52407761	0.272	0.149	1	3	NPVM
DDX27	5.43E-09	0.52326671	0.537	0.275	0.000117079	3	NPVM
VIM	6.05E-10	0.5229601	1	0.895	1.31E-05	3	NPVM
RNF144B	2.02E-10	0.52221553	0.493	0.209	4.37E-06	3	NPVM
RPLP0	8.57E-11	0.52146949	0.978	0.835	1.85E-06	3	NPVM
SMCHD1	8.48E-07	0.52101212	0.625	0.402	0.018298306	3	NPVM
AMPD2	3.31E-13	0.52035903	0.39	0.107	7.15E-09	3	NPVM

EMP3	1.24E-06	0.517679	0.912	0.777	0.02672148	3	NPVM
ANPEP	1.19E-05	0.51537361	0.272	0.121	0.257523206	3	NPVM
SLC11A1	4.92E-05	0.51386489	0.25	0.113	1	3	NPVM
CSF3R	2.69E-06	0.51235648	0.382	0.198	0.057944462	3	NPVM
MT1G	2.29E-06	0.5117904	0.632	0.41	0.049338656	3	NPVM
CYCS	4.72E-08	0.51140368	0.706	0.49	0.001017854	3	NPVM
EEF1B2	4.31E-11	0.5109337	0.971	0.848	9.30E-07	3	NPVM
HIGD2A	6.39E-07	0.51000124	0.669	0.471	0.013794734	3	NPVM
STAT4	2.56E-11	0.50598268	0.213	0.03	5.52E-07	3	NPVM
SNRPD2	6.53E-08	0.5036942	0.684	0.46	0.001408072	3	NPVM
HPS5	0.001075	0.50339859	0.324	0.209	1	3	NPVM
RPS17	1.39E-06	0.50184133	0.728	0.57	0.029926181	3	NPVM
ZFP36	3.90E-10	0.50045234	0.978	0.829	8.41E-06	3	NPVM
CCND2	2.32E-05	0.50043065	0.338	0.171	0.50134264	3	NPVM
TFRC	6.48E-06	0.50020178	0.581	0.375	0.139731936	3	NPVM
EPSTI1	2.41E-08	0.49237328	0.515	0.264	0.000519369	3	NPVM
REL	1.03E-09	0.49075587	0.853	0.614	2.23E-05	3	NPVM
HERPUD1	1.87E-08	0.48899426	0.89	0.752	0.00040237	3	NPVM
WARS	3.90E-06	0.48500886	0.331	0.154	0.084216276	3	NPVM
HCST	1.77E-06	0.48493447	0.824	0.625	0.038102651	3	NPVM
NET1	5.97E-10	0.48243181	0.279	0.074	1.29E-05	3	NPVM
ATP5MG	1.74E-07	0.48005128	0.897	0.697	0.003751285	3	NPVM
HMGB2	1.85E-06	0.47983959	0.529	0.328	0.039829147	3	NPVM
PFN1	2.24E-07	0.4757567	0.912	0.78	0.00483103	3	NPVM
SUB1	3.08E-06	0.47572653	0.757	0.62	0.066380228	3	NPVM
ELL21	4.45E-09	0.47509283	0.706	0.402	9.60E-05	3	NPVM
DUSP51	7.42E-07	0.47429701	0.515	0.275	0.015999507	3	NPVM
IFI30	9.86E-08	0.47426353	0.529	0.295	0.002127372	3	NPVM
GUK1	8.48E-06	0.47157648	0.721	0.537	0.182879498	3	NPVM
AL118516.1	1.61E-06	0.47131352	0.331	0.146	0.034790428	3	NPVM
NR4A31	1.05E-07	0.46928057	0.588	0.32	0.002271817	3	NPVM
OAT	4.96E-06	0.46846747	0.426	0.229	0.106946801	3	NPVM
HCLS1	6.72E-07	0.46733745	0.772	0.573	0.014500172	3	NPVM
USP53	7.34E-07	0.46661996	0.287	0.11	0.015823419	3	NPVM
ACOT9	3.13E-07	0.46637764	0.39	0.185	0.006748169	3	NPVM
ADAM28	2.52E-09	0.46603411	0.382	0.14	5.45E-05	3	NPVM
FYN	8.42E-09	0.4650209	0.221	0.052	0.000181553	3	NPVM
GNA12	3.71E-08	0.46499186	0.301	0.105	0.000799846	3	NPVM
CCNE2	5.15E-11	0.46380931	0.272	0.058	1.11E-06	3	NPVM
HPGD	2.22E-06	0.46360768	0.154	0.036	0.047951615	3	NPVM
STX11	1.08E-07	0.46109648	0.5	0.264	0.002322478	3	NPVM
GNA13	7.41E-10	0.46071371	0.662	0.372	1.60E-05	3	NPVM
XBP1	0.000153	0.45756127	0.544	0.38	1	3	NPVM
TSC22D1	0.003013	0.45749315	0.346	0.229	1	3	NPVM
CDC42SE2	8.69E-05	0.45673914	0.412	0.248	1	3	NPVM
LY6E	6.92E-05	0.45602452	0.485	0.314	1	3	NPVM
ZFAND2A1	2.06E-05	0.45521544	0.603	0.397	0.443563951	3	NPVM
SRSF21	1.75E-08	0.45410307	0.824	0.62	0.000377461	3	NPVM
MPC1	1.71E-06	0.45218511	0.449	0.251	0.036976451	3	NPVM
PTRHD1	4.50E-06	0.45094108	0.309	0.143	0.097174245	3	NPVM
PLP2	1.48E-07	0.44917263	0.294	0.11	0.003183896	3	NPVM
SLC7A5	1.74E-10	0.4486965	0.265	0.061	3.76E-06	3	NPVM

EIF5A	0.000269	0.44839063	0.559	0.405	1	3	NPVM
STAT3	2.93E-07	0.44669694	0.603	0.339	0.006320586	3	NPVM
ZBTB43	1.11E-06	0.44642888	0.434	0.223	0.023962132	3	NPVM
SERINC5	5.84E-07	0.44526561	0.449	0.22	0.01260555	3	NPVM
ITGB2-AS1	8.18E-12	0.44476626	0.206	0.028	1.77E-07	3	NPVM
RPSA	2.83E-08	0.44340235	0.919	0.799	0.000611193	3	NPVM
BIN1	0.000412	0.44230704	0.36	0.229	1	3	NPVM
TGIF1	5.19E-07	0.44187326	0.522	0.298	0.01118976	3	NPVM
LAPTM5	2.76E-06	0.44145096	0.963	0.826	0.059440195	3	NPVM
SKIL	5.40E-06	0.44111514	0.684	0.446	0.116501814	3	NPVM
PHACTR11	4.44E-10	0.44003921	0.728	0.38	9.58E-06	3	NPVM
GPRC5A	2.36E-07	0.43902796	0.125	0.017	0.00510176	3	NPVM
SRSF5	2.26E-08	0.43700188	0.919	0.725	0.00048702	3	NPVM
UQCRH	1.83E-05	0.43689188	0.743	0.606	0.394915557	3	NPVM
GMFG	1.19E-05	0.43573997	0.816	0.617	0.25676269	3	NPVM
CD69	1.87E-07	0.43567021	0.213	0.058	0.004041685	3	NPVM
TXN	0.000212	0.43377738	0.765	0.601	1	3	NPVM
HES4	2.00E-07	0.43316167	0.184	0.041	0.004307325	3	NPVM
CASP9	1.09E-07	0.43118096	0.213	0.058	0.002358536	3	NPVM
HGSNAT	3.33E-06	0.43098073	0.434	0.226	0.071766329	3	NPVM
TLR2	1.37E-07	0.42932715	0.537	0.289	0.002957933	3	NPVM
PNPLA81	1.38E-05	0.42908994	0.551	0.355	0.297476832	3	NPVM
ADAM8	1.93E-10	0.42908597	0.184	0.025	4.17E-06	3	NPVM
MAFF1	3.91E-08	0.42817255	0.588	0.311	0.000842495	3	NPVM
LILRB2	8.13E-05	0.42763794	0.551	0.369	1	3	NPVM
IL13RA1	5.58E-06	0.42743464	0.647	0.446	0.120476597	3	NPVM
RPL4	1.56E-08	0.42486818	0.912	0.774	0.000337411	3	NPVM
NRARP	8.87E-08	0.42351131	0.221	0.058	0.001913142	3	NPVM
ARHGAP26	0.0004	0.42249558	0.404	0.256	1	3	NPVM
DGKD	6.40E-06	0.42246989	0.316	0.143	0.137992231	3	NPVM
USP12	5.34E-05	0.42235062	0.36	0.193	1	3	NPVM
MYADM	5.61E-05	0.42090717	0.706	0.529	1	3	NPVM
NDEL1	4.10E-08	0.4197357	0.404	0.176	0.000884428	3	NPVM
NEDD9	1.06E-07	0.41841799	0.368	0.149	0.002290683	3	NPVM
RALA	0.000763	0.4179371	0.368	0.24	1	3	NPVM
CENPL	0.00439	0.41669502	0.324	0.209	1	3	NPVM
DDX21	1.80E-06	0.41560468	0.772	0.551	0.038927878	3	NPVM
ARF4	9.75E-08	0.41483136	0.691	0.444	0.002102828	3	NPVM
BZW1	0.000253	0.41459663	0.735	0.567	1	3	NPVM
DYNLT1	4.57E-05	0.41356332	0.471	0.295	0.985266226	3	NPVM
HAS1	7.11E-07	0.41126883	0.118	0.017	0.015334641	3	NPVM
ENTPD1	0.00019	0.4107599	0.331	0.193	1	3	NPVM
ARID5A	0.000902	0.40969844	0.353	0.223	1	3	NPVM
HIST3H2A	7.79E-06	0.40923722	0.221	0.08	0.168110369	3	NPVM
TRABD	1.03E-06	0.40914951	0.294	0.121	0.022279767	3	NPVM
TNFAIP31	4.53E-09	0.40669624	0.721	0.421	9.78E-05	3	NPVM
PLK2	0.000964	0.40584522	0.338	0.201	1	3	NPVM
ATP2B1	1.99E-05	0.40430967	0.787	0.584	0.429425691	3	NPVM
GPCPD1	0.000439	0.40400203	0.434	0.292	1	3	NPVM
IFNGR1	0.000145	0.40318963	0.596	0.444	1	3	NPVM
SPTY2D1	0.00127	0.40298474	0.404	0.267	1	3	NPVM
SERPINB8	1.68E-07	0.40178233	0.397	0.182	0.003620693	3	NPVM

CCNH	3.78E-06	0.40164214	0.537	0.333	0.081564354	3	NPVM
CEP68	6.06E-08	0.40012505	0.301	0.105	0.001307461	3	NPVM
THAP9-AS1	7.39E-05	0.39576314	0.301	0.152	1	3	NPVM
KIF13B	4.97E-05	0.39388912	0.287	0.138	1	3	NPVM
CD109	0.000217	0.39311956	0.169	0.066	1	3	NPVM
LRRFIP1	2.57E-06	0.39007892	0.875	0.705	0.055343221	3	NPVM
RASSF5	1.25E-06	0.38918618	0.368	0.174	0.026952377	3	NPVM
SLC16A3	3.41E-06	0.38912265	0.39	0.198	0.07355869	3	NPVM
UBALD2	1.04E-06	0.38881031	0.551	0.325	0.022447728	3	NPVM
STK4	0.004583	0.38616865	0.5	0.386	1	3	NPVM
SLCO4A1	3.56E-11	0.38593083	0.213	0.03	7.69E-07	3	NPVM
CD53	1.04E-05	0.38571352	0.809	0.592	0.223652046	3	NPVM
PPIA	2.70E-07	0.38567984	0.963	0.84	0.005817429	3	NPVM
SH3TC1	2.23E-07	0.38539875	0.434	0.201	0.004800507	3	NPVM
SH3BP4	9.03E-08	0.38509359	0.199	0.047	0.001947154	3	NPVM
TMEM154	5.44E-07	0.384608	0.176	0.044	0.011735116	3	NPVM
CMTM6	0.000384	0.38409473	0.691	0.565	1	3	NPVM
CHML1	0.000501	0.38264179	0.272	0.143	1	3	NPVM
SEC11A	4.75E-05	0.38255256	0.787	0.617	1	3	NPVM
ETF11	0.00038	0.38246333	0.662	0.49	1	3	NPVM
PIP4K2A	8.34E-05	0.38146907	0.39	0.226	1	3	NPVM
SESN2	1.91E-05	0.38142338	0.265	0.113	0.411882542	3	NPVM
JPT1	8.18E-06	0.38030031	0.522	0.325	0.176365169	3	NPVM
CDC42SE1	0.000225	0.38005054	0.493	0.331	1	3	NPVM
SDC2	4.01E-07	0.37950869	0.382	0.168	0.008656369	3	NPVM
TET2	0.000138	0.37905717	0.544	0.375	1	3	NPVM
SNAI1	1.66E-06	0.37899062	0.199	0.058	0.035731358	3	NPVM
GAPDH	1.46E-05	0.3787107	0.904	0.727	0.314549835	3	NPVM
PTPN1	2.97E-05	0.37841116	0.434	0.259	0.640312462	3	NPVM
IFI6	0.001003	0.37750289	0.397	0.264	1	3	NPVM
SMDT1	4.41E-05	0.37684067	0.61	0.43	0.951258446	3	NPVM
AC007613.1	4.86E-12	0.3764618	0.184	0.017	1.05E-07	3	NPVM
KLHL6	1.78E-06	0.37542617	0.294	0.116	0.038309161	3	NPVM
GSTO1	9.50E-05	0.37485304	0.684	0.468	1	3	NPVM
STAT1	0.002964	0.37377244	0.338	0.223	1	3	NPVM
RETN	9.67E-05	0.37169053	0.176	0.063	1	3	NPVM
ADGRE2	8.79E-08	0.37105213	0.316	0.118	0.001896011	3	NPVM
UBASH3B	6.69E-11	0.36891636	0.294	0.069	1.44E-06	3	NPVM
FURIN	0.000516	0.36869021	0.243	0.124	1	3	NPVM
SLC9A3R1	0.001348	0.36830221	0.257	0.138	1	3	NPVM
NDUFA6	0.002741	0.36766615	0.463	0.339	1	3	NPVM
FAM102B	1.43E-06	0.36758329	0.346	0.16	0.030807623	3	NPVM
LY86	3.96E-06	0.3664443	0.515	0.295	0.08539081	3	NPVM
CTNNB1	0.000762	0.36621523	0.625	0.501	1	3	NPVM
BCL2A1	0.00105	0.36614046	0.324	0.179	1	3	NPVM
DBF4	1.12E-06	0.36586781	0.243	0.083	0.024265187	3	NPVM
SBDS	0.000184	0.36421975	0.529	0.372	1	3	NPVM
BCL2	0.000653	0.36187035	0.301	0.165	1	3	NPVM
DNMBP	1.60E-06	0.36058365	0.331	0.138	0.034603907	3	NPVM
GCLM1	1.91E-05	0.36056279	0.515	0.309	0.411961244	3	NPVM
PAPOLG	5.24E-05	0.36011169	0.243	0.105	1	3	NPVM
C7orf50	0.000884	0.35841742	0.353	0.237	1	3	NPVM

SLC25A5	0.000873	0.35838649	0.735	0.606	1	3	NPVM
ODF3B	0.000385	0.35732294	0.235	0.118	1	3	NPVM
AOAH	1.46E-05	0.35699054	0.426	0.231	0.314804112	3	NPVM
KCTD20	2.01E-05	0.35677828	0.426	0.237	0.432615675	3	NPVM
TCF7	0.000236	0.35417051	0.25	0.121	1	3	NPVM
GLIPR2	7.38E-08	0.35342631	0.551	0.292	0.001592481	3	NPVM
C17orf107	6.56E-08	0.35245303	0.147	0.022	0.001415183	3	NPVM
MYL12A	6.57E-05	0.35102155	0.853	0.741	1	3	NPVM
SNX9	0.000344	0.35025973	0.618	0.457	1	3	NPVM
SSR4	0.000242	0.34855786	0.75	0.57	1	3	NPVM
RARA	0.003982	0.34818191	0.338	0.218	1	3	NPVM
NAF1	8.46E-07	0.34681783	0.199	0.055	0.018249168	3	NPVM
PPP1R15A1	4.10E-06	0.34618135	0.934	0.744	0.088481943	3	NPVM
C1orf56	4.48E-06	0.34592126	0.331	0.149	0.096630728	3	NPVM
KLHL5	0.000529	0.34581219	0.199	0.094	1	3	NPVM
VAPA	1.30E-06	0.34530065	0.831	0.625	0.027953225	3	NPVM
TSPYL21	6.06E-07	0.34511162	0.537	0.292	0.013079605	3	NPVM
THAP2	9.87E-06	0.34507538	0.272	0.113	0.213011961	3	NPVM
CORO1A	0.000194	0.34372235	0.478	0.311	1	3	NPVM
FMNL1	0.002952	0.34265313	0.426	0.3	1	3	NPVM
CDK2AP1	5.75E-05	0.34194103	0.272	0.129	1	3	NPVM
HINT1	0.001084	0.33929583	0.721	0.587	1	3	NPVM
TOP1	0.000191	0.33846084	0.757	0.595	1	3	NPVM
LINC01315	7.29E-09	0.33772032	0.243	0.061	0.000157212	3	NPVM
ATP5MC2	0.001144	0.33655877	0.846	0.702	1	3	NPVM
SPN	1.12E-05	0.336508	0.162	0.047	0.241070411	3	NPVM
ZNF394	0.002792	0.33576827	0.272	0.165	1	3	NPVM
TNFRSF10B	0.002065	0.33401129	0.272	0.163	1	3	NPVM
ZC3HAV1	0.001161	0.33394575	0.529	0.391	1	3	NPVM
SRGAP2	7.02E-06	0.33314058	0.559	0.347	0.151471114	3	NPVM
CDKN2D	6.16E-09	0.32926105	0.228	0.052	0.000132931	3	NPVM
BIRC3	4.78E-05	0.32924052	0.441	0.248	1	3	NPVM
ZFAND51	1.05E-06	0.32857577	0.853	0.7	0.022705529	3	NPVM
PAXX	0.000164	0.32791328	0.36	0.204	1	3	NPVM
RPL31	8.39E-05	0.32741534	0.919	0.807	1	3	NPVM
TAF10	0.000998	0.32698027	0.493	0.339	1	3	NPVM
SPTLC2	0.001553	0.32665005	0.426	0.287	1	3	NPVM
CNN2	0.000411	0.32606066	0.346	0.201	1	3	NPVM
ABHD5	0.001403	0.32446524	0.515	0.375	1	3	NPVM
PTPN2	0.001131	0.32417764	0.397	0.278	1	3	NPVM
AKNA	5.55E-05	0.32399542	0.294	0.14	1	3	NPVM
ARRDC3	6.32E-05	0.323986	0.632	0.419	1	3	NPVM
PHC2	0.004716	0.32368924	0.324	0.215	1	3	NPVM
CTTNBP2NL	0.000144	0.32352614	0.368	0.207	1	3	NPVM
EIF3I	0.002143	0.32190806	0.485	0.347	1	3	NPVM
ALYREF	0.001174	0.32148189	0.294	0.179	1	3	NPVM
HMGN1	0.002611	0.32064168	0.603	0.496	1	3	NPVM
SNHG9	2.46E-06	0.32023436	0.294	0.118	0.053016405	3	NPVM
COX7C	0.000342	0.32022738	0.853	0.727	1	3	NPVM
TTC19	0.000231	0.32017595	0.294	0.157	1	3	NPVM
ANKRD281	0.000128	0.31981211	0.346	0.182	1	3	NPVM
SSBP4	0.000527	0.31977062	0.301	0.174	1	3	NPVM

EDEM1	0.000731	0.31910832	0.331	0.198	1	3	NPVM
COX4I1	0.000107	0.31784524	0.89	0.752	1	3	NPVM
RNASE6	0.002243	0.31755095	0.662	0.532	1	3	NPVM
AC007384.1	0.000128	0.31648021	0.184	0.069	1	3	NPVM
HOTAIRM1	8.22E-06	0.31613218	0.301	0.132	0.177308866	3	NPVM
VSIR	0.000351	0.31336081	0.522	0.358	1	3	NPVM
VPS26A	0.000135	0.31234623	0.485	0.331	1	3	NPVM
CELF2	0.000158	0.31222731	0.824	0.697	1	3	NPVM
DDX24	8.42E-06	0.31175011	0.706	0.493	0.181678637	3	NPVM
EMD	3.78E-05	0.31163908	0.368	0.204	0.815685869	3	NPVM
GPR132	0.000539	0.31151731	0.228	0.11	1	3	NPVM
PLD4	1.33E-05	0.31079189	0.199	0.066	0.286099574	3	NPVM
PGLS	0.002338	0.30885882	0.603	0.446	1	3	NPVM
TUBB4B	0.000449	0.30833223	0.824	0.645	1	3	NPVM
FDX1	0.005881	0.3076009	0.346	0.242	1	3	NPVM
NBDY	0.006011	0.30714241	0.404	0.298	1	3	NPVM
CD1D	5.94E-05	0.30690438	0.162	0.052	1	3	NPVM
BCL3	3.82E-06	0.30627559	0.375	0.179	0.082366097	3	NPVM
STK17A	0.000504	0.30602725	0.316	0.185	1	3	NPVM
TNIP2	9.02E-06	0.30567158	0.287	0.127	0.19453102	3	NPVM
SGMS2	5.04E-06	0.30511488	0.265	0.102	0.108812386	3	NPVM
NECTIN2	0.000273	0.30506799	0.265	0.135	1	3	NPVM
LIMD2	0.002049	0.30498061	0.316	0.196	1	3	NPVM
WAS	0.001333	0.3047541	0.449	0.314	1	3	NPVM
AC004687.1	3.44E-06	0.30452292	0.213	0.066	0.074157861	3	NPVM
SLC7A11	2.96E-07	0.30400921	0.118	0.014	0.00638376	3	NPVM
PSMB8	0.001439	0.3035566	0.434	0.295	1	3	NPVM
AKIRIN2	0.000839	0.30340782	0.39	0.251	1	3	NPVM
EMILIN2	0.00057	0.30251596	0.566	0.408	1	3	NPVM
FLT3	4.83E-08	0.30229607	0.11	0.008	0.00104279	3	NPVM
APRT	0.00192	0.3005788	0.581	0.446	1	3	NPVM
PHLDB3	1.59E-06	0.29946935	0.169	0.041	0.034365213	3	NPVM
FAM129B	0.000563	0.2988874	0.169	0.069	1	3	NPVM
SMIM14	0.001751	0.29874606	0.309	0.193	1	3	NPVM
MBOAT7	1.72E-05	0.29762763	0.272	0.116	0.371347384	3	NPVM
GPRIN3	0.009133	0.29601113	0.346	0.234	1	3	NPVM
CHST11	0.000778	0.29427958	0.316	0.19	1	3	NPVM
MIDN1	3.65E-05	0.29390718	0.801	0.601	0.786910861	3	NPVM
MIF	0.000474	0.29344305	0.485	0.314	1	3	NPVM
ATG2A	3.01E-05	0.29313783	0.257	0.11	0.648375236	3	NPVM
TRAF5	0.001168	0.29299754	0.191	0.088	1	3	NPVM
CLIC1	8.05E-05	0.29278365	0.912	0.766	1	3	NPVM
RAPGEF1	0.000247	0.29180689	0.404	0.248	1	3	NPVM
PGK1	0.00114	0.29125049	0.588	0.457	1	3	NPVM
TXNL4A	0.001681	0.29115133	0.368	0.24	1	3	NPVM
ADD3	0.000374	0.29112403	0.36	0.209	1	3	NPVM
SNHG8	0.007177	0.29068909	0.559	0.441	1	3	NPVM
EID3	2.22E-06	0.29014377	0.228	0.074	0.047842731	3	NPVM
AC020656.1	1.35E-05	0.28970985	0.191	0.063	0.291683013	3	NPVM
DOCK10	0.002902	0.28924611	0.331	0.212	1	3	NPVM
NDUFC1	0.002663	0.2891443	0.478	0.344	1	3	NPVM
TPI1	4.47E-05	0.28913222	0.86	0.686	0.964109291	3	NPVM

EDF1	0.00131	0.28810808	0.772	0.612	1	3	NPVM
GRPEL1	0.000451	0.28805268	0.368	0.22	1	3	NPVM
ILF2	0.000167	0.2870977	0.368	0.209	1	3	NPVM
MIR3945HG	1.73E-06	0.28694272	0.191	0.052	0.037244397	3	NPVM
EFHD2	0.002166	0.28649582	0.64	0.515	1	3	NPVM
RANBP2	0.007465	0.28611206	0.478	0.375	1	3	NPVM
EIF2AK4	0.002	0.28573972	0.471	0.339	1	3	NPVM
TENT4B1	0.000333	0.2849269	0.309	0.168	1	3	NPVM
RPS4Y1	0.000937	0.28415047	0.794	0.672	1	3	NPVM
PSTPIP2	4.25E-06	0.28302414	0.25	0.094	0.091605221	3	NPVM
GSPT1	0.003406	0.28286471	0.434	0.306	1	3	NPVM
PMAIP11	0.000935	0.28252956	0.588	0.408	1	3	NPVM
RASSF2	0.001717	0.28204161	0.346	0.212	1	3	NPVM
RPL17	0.000523	0.28148823	0.654	0.49	1	3	NPVM
CCNL1	2.59E-06	0.28125591	0.941	0.755	0.055844759	3	NPVM
PTP4A1	0.000321	0.28045391	0.397	0.242	1	3	NPVM
ATP2B1-AS1	0.004025	0.27937469	0.456	0.325	1	3	NPVM
MRPL23	0.00557	0.27879376	0.287	0.182	1	3	NPVM
NMB	0.002361	0.27811879	0.206	0.105	1	3	NPVM
THUMPD3-AS	0.000104	0.27718327	0.566	0.38	1	3	NPVM
CHCHD7	2.55E-05	0.27646117	0.404	0.229	0.550091716	3	NPVM
MAP1LC3B1	3.08E-05	0.27602368	0.838	0.658	0.664954797	3	NPVM
LDLRAD4	0.002095	0.27533347	0.331	0.204	1	3	NPVM
FKBP5	2.08E-05	0.27515776	0.676	0.452	0.447905877	3	NPVM
PTP4A2	0.00149	0.27497014	0.632	0.499	1	3	NPVM
HS3ST3B1	2.34E-08	0.27362927	0.14	0.017	0.000503969	3	NPVM
CARD16	0.003271	0.27353152	0.485	0.35	1	3	NPVM
SBNO2	0.000164	0.27349717	0.213	0.091	1	3	NPVM
NDUFS6	0.001493	0.27317491	0.61	0.457	1	3	NPVM
BTF3	0.000533	0.27303918	0.934	0.815	1	3	NPVM
SPINT2	0.001605	0.27300034	0.441	0.303	1	3	NPVM
PARK7	0.001417	0.27294349	0.647	0.488	1	3	NPVM
HNRNPA3	0.001112	0.2709298	0.765	0.623	1	3	NPVM
EEF1D	0.000393	0.26994977	0.919	0.802	1	3	NPVM
PRELID1	0.004708	0.26877587	0.529	0.394	1	3	NPVM
AKAP131	3.20E-05	0.26824585	0.904	0.78	0.689752638	3	NPVM
GNAI3	0.009524	0.2675152	0.632	0.493	1	3	NPVM
COX8A	0.004101	0.26662757	0.721	0.579	1	3	NPVM
TUBB6	0.00012	0.26646656	0.463	0.284	1	3	NPVM
TPM3	0.001742	0.26525474	0.912	0.796	1	3	NPVM
CD58	0.000451	0.2646027	0.287	0.152	1	3	NPVM
CCDC6	0.003402	0.26414342	0.279	0.165	1	3	NPVM
MARCKSL1	0.006319	0.26408415	0.25	0.146	1	3	NPVM
ZNF385A	0.004405	0.2628096	0.375	0.245	1	3	NPVM
LCP1	0.002629	0.26224017	0.86	0.711	1	3	NPVM
SLC25A3	0.000615	0.26110937	0.831	0.719	1	3	NPVM
HIPK2	5.81E-05	0.26109411	0.279	0.129	1	3	NPVM
DRAP1	0.004745	0.26043901	0.61	0.49	1	3	NPVM
TMCC3	1.56E-06	0.26015424	0.235	0.077	0.033657766	3	NPVM
MRPL33	0.001939	0.25952266	0.456	0.317	1	3	NPVM
JMJD61	0.001941	0.2594947	0.471	0.317	1	3	NPVM
CHRAC1	0.001376	0.25798997	0.346	0.22	1	3	NPVM

PRPF4	0.00058	0.25796749	0.25	0.124	1	3	NPVM
MIR181A1HG	2.60E-07	0.25792828	0.243	0.072	0.005609207	3	NPVM
SEPT6	0.000243	0.25782884	0.397	0.231	1	3	NPVM
NDUFB8	0.002247	0.25706956	0.64	0.477	1	3	NPVM
FAM49A	0.003498	0.25587271	0.434	0.306	1	3	NPVM
ETS21	0.0002	0.25459317	0.772	0.598	1	3	NPVM
SLA	0.007069	0.25304366	0.478	0.372	1	3	NPVM
TRAPPC6A	0.000808	0.25282322	0.243	0.127	1	3	NPVM
S100A9	1.25E-34	4.81075447	0.972	0.199	2.70E-30	4	LAM
S100A8	1.25E-44	4.20813259	0.806	0.063	2.70E-40	4	LAM
FN1	6.47E-26	3.26614347	0.778	0.127	1.40E-21	4	LAM
MARCO	1.44E-30	3.13744424	0.944	0.199	3.11E-26	4	LAM
IFI27	1.67E-15	2.40105878	0.556	0.106	3.61E-11	4	LAM
FCGR3A1	3.00E-22	2.20723293	0.972	0.369	6.48E-18	4	LAM
S100A4	1.48E-16	1.84138177	1	0.793	3.19E-12	4	LAM
NCF1	4.12E-12	1.66503548	0.806	0.354	8.88E-08	4	LAM
ALDH1A1	5.39E-13	1.58661166	0.694	0.227	1.16E-08	4	LAM
TSPO	6.86E-14	1.53735028	1	0.657	1.48E-09	4	LAM
S100A6	3.64E-12	1.52632591	1	0.838	7.85E-08	4	LAM
VAMP8	2.83E-14	1.48372431	0.944	0.739	6.10E-10	4	LAM
PRDX1	1.71E-15	1.48234417	1	0.648	3.69E-11	4	LAM
C1QB1	2.43E-13	1.4762135	0.972	0.853	5.24E-09	4	LAM
FBP1	6.74E-12	1.38211706	0.694	0.216	1.45E-07	4	LAM
SMIM25	5.65E-27	1.37888577	0.667	0.076	1.22E-22	4	LAM
LTA4H	9.55E-16	1.36463811	0.75	0.218	2.06E-11	4	LAM
NUPR11	7.06E-14	1.33704432	0.639	0.151	1.52E-09	4	LAM
ALDH2	1.85E-10	1.20019865	0.806	0.354	3.98E-06	4	LAM
S100A11	3.53E-12	1.19812879	0.972	0.829	7.61E-08	4	LAM
EMP31	9.99E-09	1.18351515	0.972	0.801	0.000215439	4	LAM
APBB1IP	8.97E-10	1.18103664	0.694	0.268	1.94E-05	4	LAM
AP2S1	4.45E-08	1.16328258	0.889	0.568	0.000960857	4	LAM
C21	9.84E-09	1.16126285	0.611	0.212	0.000212295	4	LAM
ENHO	2.96E-11	1.15715957	0.278	0.032	6.38E-07	4	LAM
LST11	1.03E-08	1.13811011	0.917	0.592	0.000221745	4	LAM
C1QC1	3.46E-09	1.12330243	0.972	0.814	7.47E-05	4	LAM
RHOA	4.62E-12	1.1077563	1	0.76	9.96E-08	4	LAM
NOP10	1.21E-07	1.10566976	0.861	0.469	0.002619008	4	LAM
S100A101	2.88E-10	1.09218781	1	0.821	6.21E-06	4	LAM
LGALS1	1.62E-07	1.08205506	0.944	0.665	0.003505428	4	LAM
CFD	5.61E-09	1.07046788	1	0.894	0.000121063	4	LAM
CYP1B1	1.12E-10	1.03953305	0.5	0.117	2.42E-06	4	LAM
GSTO11	1.02E-11	1.03817229	0.944	0.495	2.19E-07	4	LAM
CRIP11	1.64E-06	1.03336333	0.75	0.387	0.035368091	4	LAM
TIMD4	8.57E-20	1.01980417	0.389	0.03	1.85E-15	4	LAM
VAMP5	5.35E-06	1.01274391	0.639	0.335	0.115454283	4	LAM
CALM3	2.28E-07	1.01085316	0.861	0.505	0.004925308	4	LAM
CYBB	2.48E-06	1.00543518	0.806	0.689	0.053403921	4	LAM
ABI3	3.70E-16	0.99692155	0.556	0.097	7.98E-12	4	LAM
TBC1D1	1.76E-06	0.96385999	0.611	0.246	0.037967854	4	LAM
PFN11	9.57E-11	0.95705503	0.972	0.803	2.06E-06	4	LAM
CSTB	8.73E-10	0.95701454	0.944	0.659	1.88E-05	4	LAM
ANXA2	2.21E-06	0.95135541	0.861	0.644	0.047595917	4	LAM

NDUFB2	3.14E-07	0.94697005	0.889	0.613	0.006777687	4	LAM
SH3BGRL31	7.16E-08	0.9463024	0.972	0.844	0.001543573	4	LAM
NDUFA4	2.00E-07	0.9446924	0.889	0.65	0.004313003	4	LAM
MGST31	2.48E-07	0.93486823	0.833	0.516	0.005352802	4	LAM
ISCU	3.83E-08	0.92899979	0.861	0.423	0.000825183	4	LAM
ARPC1B	4.92E-08	0.91284841	0.972	0.65	0.001060735	4	LAM
CYP27A1	4.63E-10	0.91170111	0.417	0.082	9.99E-06	4	LAM
ZBTB8OS	2.82E-09	0.91001321	0.611	0.188	6.09E-05	4	LAM
SIDT2	2.34E-10	0.90703628	0.583	0.158	5.06E-06	4	LAM
ATP5IF1	3.61E-08	0.90458846	0.861	0.503	0.000779312	4	LAM
RNF5	3.83E-05	0.89893969	0.472	0.194	0.826101931	4	LAM
COX5B	6.16E-08	0.89148396	0.889	0.609	0.001328517	4	LAM
ATP5MC3	7.56E-05	0.88946311	0.722	0.49	1	4	LAM
COX8A1	6.05E-05	0.88745106	0.861	0.598	1	4	LAM
SERPINF1	4.26E-06	0.88700633	0.5	0.194	0.09184506	4	LAM
VSIG4	9.78E-06	0.8850686	0.889	0.7	0.210987621	4	LAM
PSMB3	9.63E-07	0.88346682	0.806	0.43	0.020769249	4	LAM
UQCR11	6.73E-06	0.87621882	0.833	0.605	0.145188439	4	LAM
COX6C	2.80E-07	0.86218518	0.917	0.654	0.006031363	4	LAM
IFI27L2	1.56E-06	0.85679781	0.75	0.365	0.033658945	4	LAM
DPY30	1.57E-05	0.85627006	0.556	0.231	0.338271796	4	LAM
NPL	3.63E-05	0.85345282	0.583	0.3	0.783195782	4	LAM
COTL11	1.36E-07	0.85114615	0.944	0.607	0.002939662	4	LAM
TCN2	2.46E-09	0.85013778	0.5	0.134	5.32E-05	4	LAM
COA6	4.84E-14	0.84524301	0.694	0.164	1.04E-09	4	LAM
RPS27L	3.17E-08	0.84020917	0.917	0.577	0.00068342	4	LAM
PRR13	1.50E-08	0.83949595	0.889	0.447	0.000324169	4	LAM
RNASE11	5.62E-06	0.83342558	0.972	0.758	0.121319533	4	LAM
ARL3	1.74E-11	0.82911579	0.528	0.121	3.75E-07	4	LAM
NDUFA1	0.000851	0.82742123	0.806	0.594	1	4	LAM
PSMB2	1.30E-05	0.82673743	0.611	0.276	0.279845681	4	LAM
MNDA	9.48E-05	0.82436815	0.75	0.49	1	4	LAM
DYNLT11	1.32E-05	0.81966488	0.667	0.317	0.284292021	4	LAM
ATP5MF	0.000111	0.81859709	0.806	0.566	1	4	LAM
WDR83OS	2.33E-05	0.81672007	0.75	0.462	0.502596115	4	LAM
NDUFS7	0.003338	0.79975522	0.556	0.352	1	4	LAM
SPI1	1.48E-05	0.79538375	0.778	0.516	0.319550847	4	LAM
MPC2	4.54E-05	0.79511046	0.583	0.298	0.979981102	4	LAM
FUCA11	7.81E-05	0.79404232	0.611	0.309	1	4	LAM
SCP2	5.57E-07	0.78913053	0.778	0.423	0.012006579	4	LAM
SLC2A5	2.91E-20	0.7869022	0.361	0.024	6.27E-16	4	LAM
PSMC5	0.003006	0.78084508	0.583	0.356	1	4	LAM
MINOS1	1.64E-05	0.7788921	0.806	0.497	0.354437435	4	LAM
TGM2	2.58E-16	0.77861889	0.333	0.028	5.57E-12	4	LAM
VPS29	8.00E-05	0.77555217	0.667	0.395	1	4	LAM
BCAP31	0.000127	0.77433603	0.611	0.341	1	4	LAM
RAP1B	0.000116	0.77249643	0.806	0.559	1	4	LAM
GNG5	2.81E-07	0.77231196	0.972	0.68	0.006066407	4	LAM
C1orf1621	8.47E-06	0.77221168	0.944	0.661	0.182630675	4	LAM
LAMTOR2	2.83E-06	0.76645792	0.722	0.382	0.061009163	4	LAM
GIMAP41	4.30E-06	0.76355536	0.583	0.261	0.092757641	4	LAM
C19orf53	3.05E-05	0.7567715	0.694	0.406	0.656902957	4	LAM

SCPEP1	3.58E-05	0.75630464	0.5	0.225	0.772715133	4	LAM
ICAM2	2.65E-09	0.75083201	0.5	0.132	5.73E-05	4	LAM
PTGDS	1.56E-07	0.75002965	0.25	0.041	0.003360539	4	LAM
ARPC3	4.23E-06	0.74784282	0.972	0.832	0.091336718	4	LAM
CBR1	3.74E-07	0.73799206	0.583	0.21	0.008077528	4	LAM
ANXA4	5.71E-06	0.7349347	0.639	0.281	0.123157862	4	LAM
ELOB	0.000658	0.73458233	0.806	0.607	1	4	LAM
IRF2	8.19E-06	0.73069869	0.583	0.253	0.176612536	4	LAM
COX14	2.59E-06	0.72947129	0.667	0.296	0.055816758	4	LAM
CD681	1.54E-05	0.72880866	0.944	0.81	0.331245181	4	LAM
COX6B1	8.59E-07	0.72868372	0.944	0.661	0.018530023	4	LAM
REEP5	3.06E-06	0.72863554	0.833	0.473	0.065957102	4	LAM
YWHAH1	0.000229	0.72675319	0.639	0.384	1	4	LAM
ARL2	6.14E-06	0.7243596	0.528	0.207	0.132380749	4	LAM
UQCR10	4.34E-05	0.72150715	0.806	0.503	0.936755811	4	LAM
NDUFB1	3.65E-05	0.71826973	0.833	0.462	0.786652961	4	LAM
BMP2K1	0.000544	0.71606156	0.667	0.387	1	4	LAM
RRAS1	5.87E-07	0.71605629	0.528	0.184	0.012656177	4	LAM
NEDD8	0.000659	0.71515275	0.778	0.516	1	4	LAM
SELENOW	0.00011	0.70995285	0.667	0.389	1	4	LAM
NDUFB71	0.000442	0.70794879	0.75	0.43	1	4	LAM
NDUFA12	5.03E-05	0.70622482	0.611	0.311	1	4	LAM
NTAN1	1.04E-06	0.7056159	0.639	0.287	0.022432554	4	LAM
CARHSP1	1.44E-06	0.7050105	0.389	0.112	0.031097425	4	LAM
COX5A	2.19E-05	0.70447456	0.694	0.408	0.473306888	4	LAM
MOB1A	8.26E-05	0.70321336	0.806	0.538	1	4	LAM
LIPA1	3.28E-05	0.6995504	0.722	0.404	0.706594719	4	LAM
AKR1A1	4.67E-06	0.69912538	0.778	0.43	0.100678814	4	LAM
PSMA7	1.04E-05	0.69770683	0.889	0.691	0.224708264	4	LAM
MARCH2	6.53E-09	0.69736235	0.611	0.201	0.000140954	4	LAM
SRP14	1.56E-07	0.69202622	1	0.797	0.003368585	4	LAM
TSTA3	0.000103	0.69062003	0.306	0.099	1	4	LAM
ADI1	5.15E-06	0.68974838	0.556	0.233	0.111199281	4	LAM
ENO1	0.007941	0.68919676	0.75	0.575	1	4	LAM
NDUFB3	4.11E-05	0.68581295	0.639	0.32	0.886834489	4	LAM
STEAP3	2.38E-10	0.68343155	0.361	0.06	5.14E-06	4	LAM
SAMD9L	4.73E-06	0.68171298	0.5	0.171	0.101995091	4	LAM
POLR2L	2.91E-05	0.68170185	0.833	0.546	0.627878146	4	LAM
RPA2	0.000274	0.67588779	0.361	0.14	1	4	LAM
ACAA2	9.35E-05	0.6755022	0.5	0.225	1	4	LAM
SARS	0.001175	0.67216366	0.417	0.179	1	4	LAM
HSBP1	2.51E-05	0.67160457	0.806	0.488	0.541644498	4	LAM
MRPL52	2.20E-05	0.67016635	0.611	0.268	0.47412133	4	LAM
RBX1	8.80E-05	0.66857582	0.75	0.486	1	4	LAM
RTN3	2.19E-06	0.66590488	0.806	0.454	0.047335485	4	LAM
GBP1	0.000845	0.66157602	0.389	0.164	1	4	LAM
GTF3C6	6.30E-05	0.66111212	0.528	0.233	1	4	LAM
NME1	1.00E-07	0.65902959	0.417	0.108	0.002166147	4	LAM
LSM3	0.000303	0.65901203	0.639	0.376	1	4	LAM
GTF3A	0.000477	0.65813971	0.611	0.33	1	4	LAM
CAPNS1	4.84E-05	0.65645642	0.722	0.391	1	4	LAM
LGALS3	1.55E-05	0.65525808	0.889	0.62	0.334979131	4	LAM

TSPAN41	0.000493	0.65323425	0.75	0.492	1	4	LAM
BABAM1	1.15E-05	0.65092851	0.472	0.171	0.248987789	4	LAM
CISD3	3.56E-06	0.65032166	0.556	0.218	0.076842091	4	LAM
ATP5PD	2.14E-05	0.65008988	0.861	0.553	0.461923115	4	LAM
ATP5ME	0.000562	0.64961859	0.806	0.605	1	4	LAM
FLNA1	0.0002	0.64961298	0.639	0.35	1	4	LAM
ARHGDIB	3.28E-05	0.64691566	0.861	0.618	0.707753033	4	LAM
ATOX1	2.31E-05	0.64590338	0.694	0.387	0.499370145	4	LAM
COMMD3	1.06E-05	0.64501439	0.556	0.242	0.228476693	4	LAM
CIB1	0.002084	0.64425067	0.694	0.447	1	4	LAM
APOE1	2.75E-07	0.64190878	0.778	0.382	0.005924235	4	LAM
OSTF1	1.16E-05	0.64149097	0.806	0.454	0.250212386	4	LAM
NDUFC2	0.003256	0.64100848	0.639	0.423	1	4	LAM
HDGF	2.14E-06	0.63751173	0.556	0.214	0.046195022	4	LAM
AC026369.3	4.41E-18	0.6372909	0.278	0.013	9.51E-14	4	LAM
GNPDA11	0.000103	0.63481155	0.639	0.302	1	4	LAM
MMP24OS	0.000393	0.63190007	0.444	0.197	1	4	LAM
LAP3	0.000318	0.63039539	0.75	0.456	1	4	LAM
COMMD4	3.41E-05	0.62761446	0.5	0.197	0.736405972	4	LAM
TMIGD3	6.02E-07	0.62719269	0.278	0.056	0.012985915	4	LAM
GLRX	2.38E-05	0.62458993	0.778	0.421	0.513305936	4	LAM
MRPL41	5.52E-06	0.62363114	0.694	0.3	0.119029942	4	LAM
DST1	0.000111	0.62157483	0.75	0.423	1	4	LAM
TXNDC17	1.64E-05	0.61919095	0.667	0.328	0.353973541	4	LAM
PSME21	0.000169	0.61900047	0.667	0.356	1	4	LAM
LYPD2	8.76E-19	0.61648622	0.194	0.002	1.89E-14	4	LAM
MYOF	5.93E-09	0.61592185	0.556	0.156	0.000127958	4	LAM
SUCLG1	2.58E-05	0.61507409	0.556	0.238	0.555618386	4	LAM
COA3	2.07E-05	0.61305914	0.417	0.153	0.447201103	4	LAM
HDDC3	0.000127	0.61304495	0.25	0.069	1	4	LAM
COX7B	0.000119	0.61256226	0.833	0.577	1	4	LAM
DOK2	1.54E-05	0.61210528	0.778	0.425	0.332909569	4	LAM
DRAM1	3.78E-07	0.61141335	0.472	0.151	0.008149255	4	LAM
SERPINA11	0.006263	0.61002144	0.417	0.24	1	4	LAM
FABP3	2.52E-09	0.60796215	0.306	0.05	5.45E-05	4	LAM
TMA7	0.000212	0.60751691	0.917	0.728	1	4	LAM
COX4I11	0.001043	0.60693933	0.889	0.782	1	4	LAM
PLBD1	1.27E-07	0.60657996	0.583	0.197	0.002749666	4	LAM
CHCHD2	0.000265	0.60598682	0.889	0.713	1	4	LAM
COPS9	7.31E-05	0.60328173	0.778	0.469	1	4	LAM
TREM21	1.11E-06	0.60186594	0.556	0.188	0.023993963	4	LAM
RFC2	0.004036	0.600877	0.222	0.082	1	4	LAM
DNAJC10	0.000565	0.60020658	0.389	0.149	1	4	LAM
TIMM8B	4.65E-05	0.59538918	0.583	0.274	1	4	LAM
BTF3L4	9.76E-05	0.59229845	0.611	0.305	1	4	LAM
EFHD21	5.02E-05	0.59056388	0.861	0.525	1	4	LAM
SEM1	0.000128	0.5903322	0.778	0.538	1	4	LAM
ECHS1	0.000501	0.58842002	0.583	0.302	1	4	LAM
MRPS21	0.001409	0.58417153	0.611	0.367	1	4	LAM
RAB11A	0.000213	0.58321373	0.639	0.333	1	4	LAM
CNDP2	3.55E-06	0.58105189	0.639	0.27	0.076658909	4	LAM
IL18	0.000229	0.58098133	0.667	0.365	1	4	LAM

PA2G4	0.000405	0.58048804	0.611	0.365	1	4	LAM
CHCHD1	0.000292	0.57991044	0.5	0.246	1	4	LAM
UQCRFS1	4.26E-07	0.57939238	0.694	0.283	0.009188564	4	LAM
VTI1B	3.00E-05	0.57856213	0.611	0.283	0.648089481	4	LAM
PPIA1	3.10E-05	0.57818355	0.972	0.866	0.668046145	4	LAM
AL035446.1	2.33E-06	0.57781685	0.306	0.071	0.050319111	4	LAM
RAC1	0.001443	0.57645868	0.944	0.786	1	4	LAM
GBP3	7.82E-05	0.57625466	0.361	0.123	1	4	LAM
AP1S1	0.000679	0.57601808	0.306	0.112	1	4	LAM
PTGES2	2.85E-05	0.57506765	0.306	0.089	0.614352237	4	LAM
FMNL2	0.00011	0.57362224	0.472	0.199	1	4	LAM
VASP	0.000626	0.57300925	0.722	0.462	1	4	LAM
BLVRA	0.000124	0.57257408	0.694	0.352	1	4	LAM
NDUFA13	0.000242	0.57004369	0.75	0.495	1	4	LAM
GDI2	0.000223	0.57001774	0.833	0.501	1	4	LAM
CD141	0.000257	0.56742038	0.889	0.717	1	4	LAM
XRN2	0.002275	0.56693506	0.639	0.402	1	4	LAM
SLC11A11	1.13E-08	0.56554304	0.5	0.123	0.000243538	4	LAM
NAA38	0.001799	0.56454092	0.583	0.352	1	4	LAM
RGS191	3.39E-05	0.56181459	0.556	0.251	0.73069387	4	LAM
SNX61	0.000259	0.56035953	0.861	0.6	1	4	LAM
EIF2S2	0.000275	0.5598276	0.722	0.413	1	4	LAM
ACTR3	0.00153	0.55974595	0.722	0.523	1	4	LAM
DARS	0.000247	0.55580394	0.528	0.244	1	4	LAM
PHYKPL	0.002153	0.55438628	0.361	0.162	1	4	LAM
ARHGEF10L	0.000907	0.55413095	0.417	0.177	1	4	LAM
MRPS33	3.78E-09	0.5525937	0.556	0.151	8.14E-05	4	LAM
LINC01094	1.17E-17	0.55256259	0.389	0.035	2.53E-13	4	LAM
MYL12B	0.000389	0.55236011	0.889	0.693	1	4	LAM
LARS	0.000212	0.55185049	0.556	0.255	1	4	LAM
CTSL	0.002244	0.55176246	0.889	0.676	1	4	LAM
TCEAL41	0.000151	0.55121454	0.639	0.322	1	4	LAM
SELENOT	0.00017	0.55041667	0.722	0.4	1	4	LAM
FSTL1	0.000353	0.5501005	0.222	0.06	1	4	LAM
CORO1B	0.000606	0.54824224	0.611	0.315	1	4	LAM
ATP6V0E1	0.000609	0.54783754	0.833	0.689	1	4	LAM
CD81	0.002811	0.54629838	0.778	0.616	1	4	LAM
BLOC1S1	0.000571	0.54502184	0.75	0.492	1	4	LAM
DMAC1	3.42E-05	0.54482327	0.417	0.153	0.736835674	4	LAM
ECM1	3.27E-07	0.54268304	0.417	0.112	0.007060322	4	LAM
DECR1	5.12E-06	0.54097127	0.611	0.251	0.110380288	4	LAM
ACP21	6.82E-07	0.54027326	0.444	0.13	0.014719537	4	LAM
TNFRSF1A1	0.003549	0.53985904	0.611	0.352	1	4	LAM
CCDC137	0.001184	0.53914172	0.25	0.084	1	4	LAM
ABRACL	0.000508	0.53847728	0.556	0.289	1	4	LAM
MRPL55	0.001514	0.53684992	0.417	0.188	1	4	LAM
SNRPD1	0.000221	0.53529495	0.611	0.309	1	4	LAM
MIEN1	0.00076	0.53396057	0.528	0.244	1	4	LAM
ATL3	0.002199	0.53186836	0.389	0.194	1	4	LAM
PSMC2	0.003896	0.5307257	0.444	0.222	1	4	LAM
LAMTOR5	8.78E-05	0.52908909	0.722	0.428	1	4	LAM
CALR	0.005276	0.52532892	0.806	0.639	1	4	LAM

FIS1	0.003964	0.52333092	0.667	0.406	1	4	LAM
H2AFV	0.00343	0.52251463	0.667	0.462	1	4	LAM
CAPZA1	0.000721	0.52240879	0.722	0.473	1	4	LAM
MT-CO1	0.00314	0.52161638	0.833	0.996	1	4	LAM
TIMP11	0.000283	0.52009223	0.917	0.689	1	4	LAM
RAB5IF	1.50E-05	0.51983568	0.556	0.238	0.323762612	4	LAM
NDUFAF3	0.000249	0.51966828	0.611	0.3	1	4	LAM
ANAPC13	0.001571	0.51936733	0.417	0.199	1	4	LAM
PSMA4	0.000387	0.51920327	0.667	0.4	1	4	LAM
EHBP1L1	0.000942	0.51861989	0.556	0.296	1	4	LAM
IGKC	6.06E-07	0.51778588	0.444	0.125	0.013064585	4	LAM
DYNLRB1	0.000552	0.51772372	0.694	0.406	1	4	LAM
MIF1	0.007124	0.51693614	0.556	0.346	1	4	LAM
GLUL	0.000283	0.51676501	0.972	0.836	1	4	LAM
PSMC3	0.000408	0.51659618	0.472	0.225	1	4	LAM
SERPINB1	0.000812	0.5157964	0.806	0.527	1	4	LAM
PSENEN	1.44E-05	0.51317216	0.5	0.192	0.311066291	4	LAM
TMEM14B	0.004883	0.51296279	0.667	0.395	1	4	LAM
PARP11	0.000419	0.51281167	0.5	0.222	1	4	LAM
COLEC121	0.000153	0.51278392	0.722	0.38	1	4	LAM
LUM	0.000693	0.51185782	0.417	0.171	1	4	LAM
NCK1	2.87E-09	0.51075673	0.472	0.112	6.19E-05	4	LAM
GAPDH1	0.001926	0.50991436	0.972	0.76	1	4	LAM
AURKAIP1	0.000189	0.50918038	0.667	0.397	1	4	LAM
CAPZB	0.000841	0.50888704	0.833	0.618	1	4	LAM
SMPDL3A	1.30E-05	0.50758544	0.278	0.069	0.280715357	4	LAM
SNX3	0.0032	0.50676392	0.806	0.577	1	4	LAM
NSMCE1	0.000703	0.5051247	0.361	0.147	1	4	LAM
PSMA3	0.000548	0.50494638	0.639	0.333	1	4	LAM
PSMA1	0.000503	0.50431227	0.667	0.363	1	4	LAM
PTTG1IP	0.00365	0.50339534	0.694	0.43	1	4	LAM
RNASE2	1.17E-10	0.50294041	0.25	0.026	2.52E-06	4	LAM
SCIMP	0.001869	0.50205483	0.444	0.233	1	4	LAM
EPB41L21	0.000837	0.50046406	0.667	0.4	1	4	LAM
RTRAF	0.000373	0.49815073	0.639	0.374	1	4	LAM
SULT1A1	5.40E-05	0.49759535	0.5	0.201	1	4	LAM
NR1H3	1.51E-06	0.49551214	0.333	0.082	0.032597754	4	LAM
DDT	0.000545	0.49491781	0.694	0.369	1	4	LAM
SPCS1	0.000571	0.4943922	0.722	0.451	1	4	LAM
SLC25A20	2.10E-06	0.4939609	0.25	0.052	0.045239401	4	LAM
SF3B2	0.001696	0.49390659	0.556	0.27	1	4	LAM
STARD3	0.009082	0.49192889	0.361	0.19	1	4	LAM
TPMT	1.30E-05	0.49096127	0.472	0.164	0.280710657	4	LAM
MED4	2.06E-05	0.49008246	0.5	0.184	0.443911014	4	LAM
LAMTOR1	0.003162	0.48957885	0.694	0.469	1	4	LAM
NDUFB81	0.000802	0.48881695	0.806	0.499	1	4	LAM
IFT74	7.58E-06	0.48879618	0.306	0.08	0.163616749	4	LAM
AP1S21	0.000523	0.48860643	0.861	0.579	1	4	LAM
C1orf21	1.17E-10	0.48685582	0.222	0.019	2.52E-06	4	LAM
MS4A4A1	0.006989	0.48681032	0.889	0.721	1	4	LAM
ZNHIT1	0.000101	0.48600555	0.667	0.339	1	4	LAM
PGK11	0.0036	0.48499778	0.75	0.473	1	4	LAM

IGFBP6	0.000824	0.48472221	0.417	0.181	1	4	LAM
EMC7	0.003406	0.48356377	0.472	0.255	1	4	LAM
CALM2	0.000284	0.48191088	0.972	0.86	1	4	LAM
GGCT	3.49E-05	0.48179957	0.389	0.132	0.752342461	4	LAM
TALDO1	0.000868	0.4816105	0.75	0.467	1	4	LAM
NUCB1	0.000314	0.48077107	0.611	0.326	1	4	LAM
TMEM208	0.005007	0.47831506	0.472	0.259	1	4	LAM
MDH1	0.002982	0.47656314	0.528	0.307	1	4	LAM
ANAPC16	0.000768	0.47628278	0.667	0.391	1	4	LAM
MRPL28	0.000302	0.47587584	0.417	0.175	1	4	LAM
OAS1	0.001705	0.47554988	0.5	0.259	1	4	LAM
COX7C1	0.002701	0.47546892	0.861	0.754	1	4	LAM
TMEM251	0.007405	0.4743619	0.278	0.123	1	4	LAM
AES	0.002569	0.47409121	0.778	0.527	1	4	LAM
NDUFS1	0.00081	0.47388732	0.417	0.177	1	4	LAM
ZNHIT6	1.38E-05	0.47294038	0.306	0.078	0.296900982	4	LAM
COPZ1	0.002213	0.47267345	0.444	0.225	1	4	LAM
MCTS1	0.000194	0.47167437	0.5	0.205	1	4	LAM
TMBIM4	0.001863	0.47139593	0.833	0.648	1	4	LAM
CHMP2A	0.00042	0.47051857	0.722	0.387	1	4	LAM
OSTC	2.32E-05	0.46978487	0.694	0.333	0.500389795	4	LAM
PPCS1	0.000268	0.46951015	0.583	0.285	1	4	LAM
VDAC3	0.002898	0.46928084	0.472	0.27	1	4	LAM
SRI	7.67E-05	0.46886624	0.611	0.266	1	4	LAM
EHD4	6.43E-05	0.4683483	0.556	0.238	1	4	LAM
SHKBP1	0.000397	0.46613411	0.389	0.156	1	4	LAM
SPG21	3.40E-05	0.4661261	0.556	0.244	0.734235381	4	LAM
C4orf3	0.002329	0.46212606	0.722	0.464	1	4	LAM
SLFN5	2.95E-05	0.46120046	0.361	0.112	0.636990558	4	LAM
LGALS9	0.002327	0.45771554	0.75	0.43	1	4	LAM
MRPL16	0.001334	0.45692746	0.361	0.149	1	4	LAM
DHRS4	1.41E-05	0.45564331	0.389	0.125	0.303661072	4	LAM
UBE2L6	0.004583	0.45548441	0.389	0.197	1	4	LAM
PPP1R7	0.0002	0.45459395	0.472	0.201	1	4	LAM
CCDC124	0.003777	0.4543838	0.333	0.151	1	4	LAM
UBE2D4	4.18E-09	0.45428285	0.333	0.058	9.01E-05	4	LAM
HOXB2	0.001574	0.45335832	0.25	0.084	1	4	LAM
ARMC10	0.003237	0.45034257	0.306	0.127	1	4	LAM
TMCO1	0.000208	0.45012345	0.667	0.33	1	4	LAM
FYB1	0.001141	0.45002028	0.806	0.536	1	4	LAM
NDUFB11	0.000565	0.44911033	0.75	0.531	1	4	LAM
ALOX5	0.001806	0.44789835	0.528	0.259	1	4	LAM
PSMA5	0.001093	0.44787582	0.583	0.296	1	4	LAM
AKIP1	0.000223	0.44762434	0.306	0.099	1	4	LAM
LSM10	0.000525	0.44711412	0.444	0.203	1	4	LAM
CLEC12A	0.004521	0.44600731	0.417	0.22	1	4	LAM
TOR1B	0.000825	0.44584206	0.167	0.041	1	4	LAM
ERH	0.003043	0.44579318	0.639	0.395	1	4	LAM
DHRS4L2	1.25E-05	0.44521554	0.472	0.173	0.269359971	4	LAM
COMMD8	4.45E-06	0.4447888	0.472	0.153	0.095949245	4	LAM
PSMD8	0.007771	0.44457986	0.611	0.395	1	4	LAM
ARPC5	0.000332	0.44395581	0.972	0.734	1	4	LAM

EIF4G3	0.000161	0.44383954	0.5	0.218	1	4	LAM
FKBP3	0.000118	0.44352991	0.528	0.225	1	4	LAM
ISCA2	0.001892	0.44331673	0.25	0.091	1	4	LAM
COQ2	0.006272	0.44249997	0.25	0.104	1	4	LAM
PHPT1	0.009515	0.44140511	0.639	0.408	1	4	LAM
CLIP4	0.001488	0.44073455	0.25	0.089	1	4	LAM
UQCRB	0.003335	0.44057874	0.917	0.767	1	4	LAM
DNPH1	0.007464	0.43904979	0.444	0.235	1	4	LAM
TCEAL8	8.28E-06	0.43846417	0.556	0.21	0.17867615	4	LAM
IDH1	0.000418	0.43789105	0.417	0.179	1	4	LAM
CAPZA2	0.001144	0.43780468	0.806	0.581	1	4	LAM
EMC10	0.00044	0.43762616	0.528	0.242	1	4	LAM
LSM5	0.003857	0.43673466	0.5	0.276	1	4	LAM
ECH1	0.001087	0.43623399	0.611	0.32	1	4	LAM
EPRS	0.00227	0.43619384	0.444	0.212	1	4	LAM
PDCD6	0.000141	0.43610526	0.639	0.313	1	4	LAM
GORASP21	0.006992	0.43581353	0.25	0.099	1	4	LAM
ATP5MG1	0.001273	0.43388172	0.944	0.737	1	4	LAM
DBNL	0.000314	0.43366829	0.611	0.317	1	4	LAM
SIGIRR	0.000485	0.43284078	0.444	0.199	1	4	LAM
DNMT3A	0.000717	0.43150886	0.306	0.106	1	4	LAM
ZDHHC20	0.001701	0.42926427	0.389	0.175	1	4	LAM
CREG11	0.001569	0.4282234	0.861	0.607	1	4	LAM
RPA3	0.00028	0.42710123	0.417	0.171	1	4	LAM
PPIC	0.000266	0.42673078	0.194	0.048	1	4	LAM
MLH3	4.08E-08	0.42530291	0.361	0.076	0.000879214	4	LAM
HSD17B10	0.00292	0.42470916	0.333	0.143	1	4	LAM
GIMAP71	7.12E-06	0.42329077	0.528	0.184	0.153527095	4	LAM
CD2BP2	0.000388	0.42277213	0.389	0.153	1	4	LAM
AGTRAP	0.000187	0.42223328	0.528	0.235	1	4	LAM
ETFA	1.56E-05	0.42221478	0.556	0.227	0.336238095	4	LAM
TRMT112	0.004793	0.42157882	0.722	0.514	1	4	LAM
MGST2	0.008017	0.42141066	0.583	0.333	1	4	LAM
CLECL1	0.00533	0.42138336	0.25	0.102	1	4	LAM
SRSF9	0.005075	0.42106903	0.694	0.419	1	4	LAM
SGTB	0.001373	0.42067177	0.306	0.114	1	4	LAM
PDIA3	0.001942	0.42064952	0.833	0.54	1	4	LAM
BTBD6	0.000836	0.42034006	0.278	0.097	1	4	LAM
KYNU	0.001768	0.42012749	0.556	0.287	1	4	LAM
SH3BGRL	0.007817	0.41967518	0.917	0.702	1	4	LAM
MRPS34	0.000229	0.41928246	0.5	0.212	1	4	LAM
ACOT7	5.16E-05	0.4190804	0.167	0.03	1	4	LAM
TAF12	5.59E-05	0.41892398	0.361	0.123	1	4	LAM
NLRC4	0.003777	0.41871772	0.139	0.037	1	4	LAM
UBE2Q2	0.000291	0.41770513	0.389	0.153	1	4	LAM
TRIR	0.005415	0.41768895	0.833	0.59	1	4	LAM
MYO1G	1.58E-05	0.41759748	0.25	0.058	0.340005216	4	LAM
GPBAR1	4.43E-05	0.41690973	0.278	0.071	0.95473821	4	LAM
ARPC4	0.000596	0.41481602	0.861	0.527	1	4	LAM
RECQL	0.001118	0.4137493	0.5	0.235	1	4	LAM
TAGLN2	0.005029	0.41203598	0.917	0.717	1	4	LAM
SQOR	0.000422	0.41193409	0.444	0.203	1	4	LAM

NXPE3	1.13E-05	0.41161148	0.25	0.056	0.243633836	4	LAM
TARBP2	6.96E-05	0.41154607	0.167	0.03	1	4	LAM
FUCA2	0.00245	0.41093741	0.444	0.222	1	4	LAM
WASHC3	0.000457	0.41059995	0.472	0.205	1	4	LAM
RARRES3	5.70E-05	0.41056366	0.444	0.16	1	4	LAM
MED16	0.008118	0.41047404	0.25	0.108	1	4	LAM
ZDHHC12	0.001314	0.40944857	0.361	0.147	1	4	LAM
HMGN3	0.002631	0.4093747	0.694	0.449	1	4	LAM
PIN1	2.48E-05	0.40886602	0.583	0.242	0.53563069	4	LAM
TIMM10	0.001285	0.40862947	0.444	0.201	1	4	LAM
THAP3	0.00095	0.40843235	0.194	0.054	1	4	LAM
FBXL15	0.000551	0.4080583	0.361	0.14	1	4	LAM
TSPAN31	0.000656	0.40699893	0.472	0.207	1	4	LAM
CMC1	0.000541	0.40693132	0.417	0.175	1	4	LAM
TMEM173	7.23E-05	0.40595573	0.5	0.201	1	4	LAM
FFAR4	2.23E-08	0.40520164	0.222	0.028	0.000480801	4	LAM
TXNDC12	0.000452	0.40493512	0.583	0.294	1	4	LAM
CLIC4	0.000495	0.40419083	0.444	0.179	1	4	LAM
ANKRD49	0.003204	0.4039935	0.222	0.076	1	4	LAM
PSMB6	0.007239	0.40358857	0.639	0.432	1	4	LAM
DNASE21	0.000166	0.4034297	0.333	0.114	1	4	LAM
PPIL3	0.000507	0.40324111	0.333	0.125	1	4	LAM
HMMR	1.04E-05	0.4031025	0.139	0.017	0.223385212	4	LAM
ATP5MPL	0.00443	0.40279026	0.694	0.508	1	4	LAM
TMEM19	0.003204	0.40206761	0.278	0.108	1	4	LAM
LILRA2	4.47E-07	0.40184599	0.278	0.052	0.009639814	4	LAM
COX18	0.002219	0.40109159	0.167	0.045	1	4	LAM
DIAPH11	0.000178	0.40010005	0.5	0.225	1	4	LAM
HOXB6	8.62E-09	0.39802227	0.333	0.058	0.00018604	4	LAM
CD226	1.37E-05	0.39545664	0.222	0.045	0.295340357	4	LAM
POLR2K	4.31E-06	0.39405589	0.611	0.229	0.092945637	4	LAM
PFDN5	0.004868	0.39388368	1	0.898	1	4	LAM
PRELID11	0.003444	0.39328582	0.667	0.413	1	4	LAM
TAF9	0.005434	0.39316262	0.417	0.212	1	4	LAM
PYGL	3.09E-05	0.39258286	0.389	0.127	0.665711307	4	LAM
TMEM230	0.002288	0.39212226	0.583	0.341	1	4	LAM
CCDC69	1.08E-05	0.38849797	0.278	0.069	0.233776738	4	LAM
CCDC90B	0.000298	0.38706296	0.417	0.166	1	4	LAM
CCND1	0.003265	0.38649321	0.25	0.095	1	4	LAM
PSME11	0.001974	0.38603967	0.75	0.523	1	4	LAM
MZT1	0.002913	0.38552734	0.278	0.112	1	4	LAM
GSTK1	0.007338	0.3846282	0.667	0.41	1	4	LAM
RPL26L1	0.003724	0.38390572	0.389	0.197	1	4	LAM
ECHDC2	0.000531	0.38313188	0.167	0.039	1	4	LAM
FDX2	0.000203	0.3824104	0.25	0.073	1	4	LAM
PCK2	1.78E-06	0.38178809	0.25	0.05	0.038358036	4	LAM
BAK1	3.84E-05	0.38121989	0.278	0.073	0.827405663	4	LAM
S100A13	0.002356	0.38121287	0.389	0.177	1	4	LAM
GABARAP	0.006645	0.38108402	0.639	0.389	1	4	LAM
TMEM104	0.000354	0.38017372	0.194	0.05	1	4	LAM
STUB1	0.001193	0.37908483	0.556	0.281	1	4	LAM
PPIE	0.003636	0.37798076	0.333	0.14	1	4	LAM

PIGM	2.46E-05	0.37795505	0.194	0.037	0.529888132	4	LAM
NDUFS5	0.003342	0.37749727	0.778	0.527	1	4	LAM
ACP1	0.008646	0.3762744	0.417	0.238	1	4	LAM
MRPL11	0.005112	0.37601196	0.444	0.231	1	4	LAM
COPRS	0.002072	0.37570492	0.444	0.214	1	4	LAM
LSM4	0.006342	0.37478037	0.5	0.279	1	4	LAM
CAP1	0.004326	0.37218124	0.917	0.65	1	4	LAM
PLGRKT	9.96E-05	0.37189751	0.417	0.151	1	4	LAM
CR1	0.008476	0.37175284	0.444	0.227	1	4	LAM
GLO1	0.001942	0.37065015	0.472	0.229	1	4	LAM
MRPL51	0.008693	0.37037183	0.611	0.363	1	4	LAM
PSMB91	0.002745	0.36980984	0.611	0.326	1	4	LAM
PHB	0.009233	0.36795144	0.5	0.289	1	4	LAM
RXRA	1.58E-06	0.36791896	0.528	0.173	0.034095125	4	LAM
TP53	0.006946	0.36716319	0.25	0.099	1	4	LAM
STXBP2	0.003034	0.3671411	0.444	0.214	1	4	LAM
PYCARD1	0.007329	0.36659023	0.75	0.579	1	4	LAM
TMED1	0.000279	0.36538544	0.361	0.127	1	4	LAM
SEC11C	0.001652	0.36512456	0.472	0.24	1	4	LAM
MPZL2	1.24E-05	0.36511003	0.111	0.011	0.268034401	4	LAM
ERAP2	5.26E-07	0.36446872	0.389	0.099	0.011354035	4	LAM
NDUFA11	0.003263	0.36399576	0.722	0.467	1	4	LAM
CYB5A	0.002249	0.36227282	0.417	0.186	1	4	LAM
CXADR	5.45E-12	0.36022438	0.194	0.011	1.18E-07	4	LAM
SSPN	0.001281	0.35912171	0.167	0.043	1	4	LAM
PRSS23	3.18E-08	0.35890112	0.222	0.028	0.000686319	4	LAM
NCK1-DT	0.000403	0.35879574	0.222	0.063	1	4	LAM
PDLIM1	6.31E-06	0.35831279	0.306	0.076	0.136199137	4	LAM
THYN1	0.001893	0.35828503	0.333	0.136	1	4	LAM
PSMB1	0.007033	0.35777337	0.75	0.54	1	4	LAM
CPPED1	0.000219	0.3565463	0.389	0.145	1	4	LAM
BST1	2.80E-05	0.35477054	0.222	0.05	0.604597841	4	LAM
SMIM37	0.0001	0.35454244	0.472	0.188	1	4	LAM
RABL3	0.007671	0.35276619	0.167	0.056	1	4	LAM
MRPL44	1.32E-08	0.35251404	0.361	0.073	0.000285272	4	LAM
MPST	0.000119	0.35249852	0.361	0.123	1	4	LAM
STX8	0.001424	0.35226364	0.444	0.216	1	4	LAM
CCDC167	7.56E-07	0.34918305	0.361	0.091	0.016310965	4	LAM
NTPCR	0.004593	0.34903447	0.278	0.119	1	4	LAM
ROMO1	0.007571	0.34882782	0.583	0.339	1	4	LAM
NRBP1	0.002685	0.34871711	0.444	0.214	1	4	LAM
GLTP	0.001016	0.3480866	0.389	0.162	1	4	LAM
SMIM71	0.005123	0.34762746	0.528	0.309	1	4	LAM
YIPF4	0.002186	0.34745513	0.444	0.212	1	4	LAM
CLOCK	0.005181	0.34735384	0.278	0.117	1	4	LAM
GANC	6.99E-06	0.3468071	0.306	0.076	0.150724694	4	LAM
PGM2	0.000401	0.34554804	0.306	0.104	1	4	LAM
ITLN1	0.002012	0.34507827	0.278	0.102	1	4	LAM
SUMF2	0.00101	0.34452526	0.528	0.24	1	4	LAM
NCEH11	0.001256	0.34343227	0.417	0.181	1	4	LAM
ASAP2	6.33E-05	0.34300054	0.222	0.052	1	4	LAM
CYB561D2	0.001058	0.34290551	0.417	0.177	1	4	LAM

CHMP5	0.000918	0.34259002	0.583	0.298	1	4	LAM
NDUFAF8	0.002296	0.34257071	0.472	0.229	1	4	LAM
TYMP1	0.009015	0.3416915	0.917	0.633	1	4	LAM
MCRIP1	0.008251	0.34125746	0.583	0.328	1	4	LAM
MMADHC	0.003122	0.34086329	0.444	0.229	1	4	LAM
UGGT1	0.002467	0.33993343	0.306	0.125	1	4	LAM
GDE1	0.008412	0.33946037	0.389	0.212	1	4	LAM
HIGD2A1	0.006243	0.33853783	0.778	0.505	1	4	LAM
TMEM69	0.000217	0.33806399	0.222	0.058	1	4	LAM
NHLRC2	0.005934	0.33708339	0.194	0.067	1	4	LAM
MRPL35	0.00029	0.33640646	0.333	0.114	1	4	LAM
NDUFS61	0.002299	0.33547134	0.778	0.477	1	4	LAM
HERPUD2	0.002225	0.33509045	0.333	0.13	1	4	LAM
SLC25A24	4.70E-05	0.33499237	0.5	0.192	1	4	LAM
GEMIN6	7.11E-06	0.33487401	0.222	0.043	0.153341309	4	LAM
SSU72	0.004069	0.33464941	0.611	0.352	1	4	LAM
MED25	0.003215	0.33428048	0.306	0.121	1	4	LAM
NOC3L	0.00991	0.33389401	0.25	0.104	1	4	LAM
CD40	0.000809	0.33304263	0.333	0.125	1	4	LAM
MTAP	1.59E-05	0.33254132	0.389	0.119	0.342509741	4	LAM
S1PR1	3.00E-06	0.33207135	0.194	0.03	0.064813007	4	LAM
PTPRO	2.63E-06	0.32974274	0.278	0.058	0.05669576	4	LAM
NCKAP1L1	0.007378	0.32701635	0.694	0.473	1	4	LAM
NIT2	0.003042	0.32680725	0.333	0.14	1	4	LAM
RAB9A	0.000254	0.32680672	0.361	0.123	1	4	LAM
KNOP1	0.000171	0.32630376	0.306	0.095	1	4	LAM
MGLL1	0.000161	0.32627711	0.528	0.21	1	4	LAM
CCDC125	0.004625	0.32302032	0.139	0.037	1	4	LAM
SNAP291	9.21E-05	0.32273891	0.444	0.171	1	4	LAM
BDH2	4.53E-05	0.32190141	0.25	0.06	0.97784534	4	LAM
REXO2	0.002002	0.32123097	0.361	0.162	1	4	LAM
DCTN21	0.00114	0.32115067	0.417	0.184	1	4	LAM
AAED1	0.005428	0.3205631	0.417	0.21	1	4	LAM
MRPL22	0.000937	0.32042126	0.361	0.143	1	4	LAM
PRR34-AS1	0.000158	0.32042017	0.194	0.045	1	4	LAM
TMEM141	0.000332	0.32023678	0.417	0.171	1	4	LAM
HSD17B4	0.003746	0.31979979	0.528	0.27	1	4	LAM
CD1801	0.00292	0.3186311	0.306	0.127	1	4	LAM
CYSTM1	0.003056	0.31808222	0.472	0.225	1	4	LAM
G6PD	0.003771	0.31794502	0.278	0.117	1	4	LAM
XPO5	0.005938	0.31734317	0.139	0.039	1	4	LAM
HSD17B12	0.004373	0.31693407	0.472	0.248	1	4	LAM
GBA	0.001669	0.31594844	0.361	0.149	1	4	LAM
APOC11	2.24E-08	0.31581264	0.639	0.21	0.000482642	4	LAM
ZBTB7B	9.02E-05	0.31515149	0.25	0.067	1	4	LAM
TMEM242	6.44E-05	0.31491475	0.306	0.091	1	4	LAM
NDUFAB1	0.009974	0.31479216	0.556	0.335	1	4	LAM
HADHB	0.005613	0.31398126	0.611	0.328	1	4	LAM
SNX17	0.005168	0.31388067	0.444	0.229	1	4	LAM
RPS19BP1	0.000879	0.31374062	0.611	0.298	1	4	LAM
RMDN1	0.001515	0.31348195	0.306	0.117	1	4	LAM
H2AFY	0.005446	0.31315543	0.833	0.609	1	4	LAM

MAP4	0.007365	0.31298913	0.361	0.173	1	4	LAM
TTC1	0.003199	0.3129545	0.5	0.238	1	4	LAM
PTPRJ1	0.001791	0.31219289	0.417	0.184	1	4	LAM
DEFB1	4.00E-13	0.31046035	0.139	0.002	8.63E-09	4	LAM
PIGH	2.08E-06	0.31036679	0.278	0.058	0.044874735	4	LAM
CEP55	2.51E-07	0.31023139	0.111	0.006	0.005407668	4	LAM
LSM8	0.003021	0.30965221	0.528	0.276	1	4	LAM
RNF20	0.002537	0.30887283	0.278	0.102	1	4	LAM
TMEM134	0.006643	0.30645707	0.472	0.229	1	4	LAM
SIRT2	0.008926	0.30645425	0.389	0.199	1	4	LAM
TMED3	0.007098	0.30620783	0.25	0.102	1	4	LAM
RPS6KA4	0.003431	0.30609337	0.333	0.145	1	4	LAM
RBM42	0.009005	0.30608884	0.444	0.222	1	4	LAM
PDZD11	0.00125	0.30450568	0.306	0.117	1	4	LAM
TFDP2	0.004415	0.30443259	0.306	0.127	1	4	LAM
POP7	0.001536	0.30406947	0.25	0.089	1	4	LAM
COL6A1	0.000303	0.30380542	0.278	0.084	1	4	LAM
SKA2	0.000902	0.30220119	0.222	0.067	1	4	LAM
PRR5L	1.26E-10	0.30098303	0.222	0.019	2.72E-06	4	LAM
PWP1	3.74E-05	0.30076636	0.389	0.125	0.807669984	4	LAM
UXT	0.002937	0.29974305	0.778	0.484	1	4	LAM
MLXIPL	3.53E-10	0.29970506	0.194	0.015	7.63E-06	4	LAM
CDK2AP2	0.00037	0.29954081	0.444	0.184	1	4	LAM
LYZ1	0.000368	0.2994771	0.972	0.747	1	4	LAM
OSCAR	7.30E-06	0.29930253	0.25	0.054	0.157535952	4	LAM
TMEM126B	8.12E-05	0.29806607	0.472	0.177	1	4	LAM
METTL7A	0.002337	0.29746298	0.583	0.328	1	4	LAM
KDM1B	0.00466	0.29730373	0.25	0.099	1	4	LAM
UQCRC1	0.008377	0.29583004	0.444	0.24	1	4	LAM
RALB	0.000373	0.2956773	0.556	0.246	1	4	LAM
NMT1	0.00485	0.29558649	0.444	0.216	1	4	LAM
EEF1AKMT2	4.39E-05	0.29552106	0.222	0.05	0.948047951	4	LAM
AP3M1	0.002158	0.29511922	0.25	0.086	1	4	LAM
OTUB1	0.009589	0.29428406	0.389	0.194	1	4	LAM
PRDX5	0.009938	0.29402326	0.694	0.445	1	4	LAM
STMP1	0.003659	0.29387283	0.528	0.281	1	4	LAM
NARS	0.006622	0.29373634	0.5	0.268	1	4	LAM
ZNHIT3	0.004177	0.29324492	0.361	0.164	1	4	LAM
SLC36A1	0.000108	0.29310079	0.194	0.043	1	4	LAM
ALDH9A1	0.003613	0.29260763	0.389	0.186	1	4	LAM
KIN	0.000963	0.29208391	0.361	0.143	1	4	LAM
UCHL3	0.003035	0.29191993	0.278	0.112	1	4	LAM
CARD6	0.00247	0.29187608	0.278	0.106	1	4	LAM
RPP30	0.008331	0.2917966	0.278	0.121	1	4	LAM
ATP6V1D	0.006543	0.29132831	0.472	0.248	1	4	LAM
C3AR11	0.004836	0.29129498	0.806	0.512	1	4	LAM
SPTBN1	0.000237	0.29097055	0.25	0.069	1	4	LAM
PTPN6	0.000854	0.29092385	0.583	0.276	1	4	LAM
MTCH2	0.001946	0.29092358	0.361	0.156	1	4	LAM
CENPX	0.003502	0.29088017	0.417	0.19	1	4	LAM
MIF4GD	0.003328	0.29077731	0.361	0.153	1	4	LAM
SMIM30	8.78E-05	0.29074972	0.528	0.212	1	4	LAM

COMMD10	0.004907	0.29066057	0.306	0.127	1	4	LAM
NABP2	0.000324	0.29028497	0.194	0.048	1	4	LAM
NAXE	0.001511	0.28984854	0.278	0.106	1	4	LAM
GIMAP11	0.000703	0.28944231	0.417	0.175	1	4	LAM
SMIM29	0.001661	0.2875942	0.278	0.102	1	4	LAM
TSNAX	0.004706	0.28727087	0.361	0.173	1	4	LAM
ARFRP1	0.005781	0.28677376	0.306	0.132	1	4	LAM
SFRP1	1.12E-05	0.28529742	0.139	0.017	0.242032899	4	LAM
STX12	0.002036	0.28507386	0.472	0.216	1	4	LAM
GIPC1	0.008095	0.28350979	0.306	0.138	1	4	LAM
HINT2	0.00961	0.28272891	0.333	0.164	1	4	LAM
CARD8-AS1	0.001031	0.28250668	0.25	0.082	1	4	LAM
HPCAL1	0.00196	0.28244416	0.472	0.227	1	4	LAM
MRPL12	9.52E-06	0.28193889	0.389	0.117	0.205432791	4	LAM
BCAP29	0.006354	0.28087592	0.361	0.166	1	4	LAM
PGAM5	2.64E-07	0.27878046	0.222	0.032	0.005699947	4	LAM
ARHGAP35	0.001496	0.2782724	0.25	0.082	1	4	LAM
ALG14	0.005588	0.27782968	0.139	0.039	1	4	LAM
GNGT2	2.29E-06	0.2776703	0.194	0.03	0.049469078	4	LAM
ATP5S	0.002509	0.27731597	0.25	0.091	1	4	LAM
SLC35F61	0.002776	0.27726351	0.417	0.188	1	4	LAM
SNX241	1.04E-05	0.27709094	0.444	0.138	0.224787565	4	LAM
MDH2	0.008093	0.27591774	0.5	0.283	1	4	LAM
UGDH	0.004495	0.27557609	0.222	0.078	1	4	LAM
RNASEL	2.35E-05	0.27521901	0.194	0.037	0.507533802	4	LAM
SSBP1	0.006827	0.27503476	0.722	0.486	1	4	LAM
HTATSF1	0.000683	0.27478802	0.361	0.136	1	4	LAM
POP4	0.000626	0.27478565	0.361	0.134	1	4	LAM
DUSP18	0.00012	0.27468565	0.222	0.054	1	4	LAM
EXOC6	0.000196	0.27429008	0.25	0.069	1	4	LAM
MRC2	0.001097	0.27401857	0.139	0.03	1	4	LAM
CC2D1A	0.000894	0.27392556	0.167	0.041	1	4	LAM
SCD	0.002586	0.2731192	0.278	0.104	1	4	LAM
SASH3	0.005746	0.27300401	0.222	0.086	1	4	LAM
SELPLG	0.006136	0.27178256	0.361	0.164	1	4	LAM
RIOK3	0.004068	0.27123676	0.556	0.279	1	4	LAM
UBXN11	0.008233	0.26900729	0.389	0.186	1	4	LAM
TNS3	0.008169	0.2687012	0.417	0.218	1	4	LAM
WRNIP1	0.009709	0.26863373	0.222	0.086	1	4	LAM
VCL	0.002448	0.26862434	0.306	0.119	1	4	LAM
TXNRD2	0.000357	0.26859018	0.222	0.06	1	4	LAM
DAXX	4.53E-05	0.26855157	0.25	0.06	0.97784534	4	LAM
RIN2	0.000374	0.26624008	0.5	0.214	1	4	LAM
CORO7	0.002263	0.26611262	0.389	0.162	1	4	LAM
ELK1	0.004329	0.26589998	0.194	0.067	1	4	LAM
IKBKE	0.001548	0.26555052	0.139	0.032	1	4	LAM
UBIAD1	7.06E-05	0.26534164	0.194	0.041	1	4	LAM
HYAL2	0.006297	0.2646432	0.167	0.054	1	4	LAM
P2RY11	0.000563	0.26459306	0.139	0.028	1	4	LAM
PEX3	0.000179	0.26332055	0.194	0.045	1	4	LAM
APOO	0.000571	0.26326318	0.139	0.028	1	4	LAM
GRIN2C	1.28E-07	0.26297154	0.139	0.011	0.002757364	4	LAM

MSRB1	4.28E-11	0.26239133	0.25	0.024	9.23E-07	4	LAM
ECSIT	0.0006	0.26141227	0.25	0.078	1	4	LAM
RAMMET	0.000647	0.26063773	0.389	0.158	1	4	LAM
DENR	0.0041	0.2604306	0.444	0.216	1	4	LAM
GNPTAB	0.000385	0.26018094	0.444	0.181	1	4	LAM
RWDD2B	5.05E-06	0.25954348	0.194	0.032	0.108933471	4	LAM
C4orf48	0.00728	0.25891337	0.556	0.326	1	4	LAM
RBMS2	0.001774	0.25854892	0.222	0.071	1	4	LAM
TNFAIP8L2	0.009171	0.25843572	0.25	0.106	1	4	LAM
SNRPC	0.006882	0.25835211	0.444	0.229	1	4	LAM
MRPL27	0.000749	0.25786806	0.417	0.175	1	4	LAM
PML	0.005208	0.25778621	0.278	0.114	1	4	LAM
LINC02432	2.55E-06	0.2570305	0.167	0.022	0.054921584	4	LAM
TSN	0.003223	0.25698461	0.333	0.143	1	4	LAM
POGK	0.003919	0.25695951	0.306	0.127	1	4	LAM
C3orf14	0.001405	0.25684928	0.222	0.071	1	4	LAM
CMKLR11	0.00427	0.25650958	0.417	0.205	1	4	LAM
CSTF3	0.004919	0.25542129	0.222	0.08	1	4	LAM
FAM118B	9.72E-08	0.25468348	0.222	0.03	0.002096867	4	LAM
LRRC2	7.88E-11	0.25458197	0.167	0.009	1.70E-06	4	LAM
DHFR	0.005588	0.25447305	0.139	0.039	1	4	LAM
ZMAT3	0.001692	0.25425188	0.25	0.084	1	4	LAM
CNOT10	0.001269	0.2532665	0.194	0.056	1	4	LAM
MRPS35	0.003631	0.25137171	0.306	0.123	1	4	LAM
DPM2	2.22E-05	0.25081232	0.361	0.106	0.478813346	4	LAM

Supplementary Table S3b: Differentially expressed genes in VAT macrophages between patients with or without CAC by pseudo-bulk analysis.

	baseMean	log2FC	lfcSE	stat	pvalue	padj
S100A9	339.029555	3.316957526	0.665524	4.9839766	6.23E-07	3.03E-05
S100A8	159.589091	3.314853728	0.93288	3.5533543	0.0003804	0.007731766
IGKC	16.3492844	2.994359805	0.54974	5.44687	5.13E-08	3.30E-06
FN1	193.627726	2.412657366	0.728061	3.3138114	0.0009203	0.015685067
SMPDL3A	5.47938077	2.380100924	0.717425	3.3175623	0.0009081	0.015628513
PLAU	7.92641965	2.37546262	0.563617	4.2146775	2.50E-05	0.000765811
ENHO	8.8214463	2.255993992	0.574272	3.9284414	8.55E-05	0.002212816
CCL8	10.5895481	2.175778978	0.65283	3.3328396	0.0008596	0.015091878
ARHGAP25	5.74563664	2.156210288	0.646428	3.3355765	0.0008512	0.014996537
CMKLR1	11.5227863	2.155049939	0.505299	4.2649005	2.00E-05	0.000635542
LINC01094	5.93342116	2.114487379	0.63762	3.3162202	0.0009124	0.015636031
SLC2A5	5.78566666	2.104348718	0.65061	3.2344244	0.0012189	0.019490465
PLXNC1	5.57497467	2.05543968	0.683794	3.0059334	0.0026477	0.035450537
CCL4L2	71.5200886	1.975382278	0.560218	3.5260936	0.0004217	0.008402959
CCL2	43.2609865	1.823589	0.574168	3.1760528	0.0014929	0.022853781
GBP1	29.0029511	1.738498175	0.477873	3.6379951	0.0002748	0.005870694
DNASE2	9.45514117	1.669106194	0.487113	3.4265272	0.0006114	0.01141885
GBP4	14.2987304	1.629156725	0.497509	3.2746257	0.001058	0.017360525
C3	17.6420592	1.628077814	0.401739	4.052578	5.07E-05	0.00141303
P2RX7	10.6117735	1.61029417	0.448719	3.5886466	0.0003324	0.006868212
ABI3	9.36656368	1.46923121	0.493596	2.9765885	0.0029147	0.037621998
MRPL27	9.14413344	1.459853466	0.489737	2.9808956	0.0028741	0.037192513
CALHM6	18.818524	1.378405947	0.398696	3.4572826	0.0005457	0.010497011
FMNL2	12.6498561	1.376507034	0.415431	3.3134431	0.0009215	0.015685067
NUPR1	18.3001625	1.367725804	0.425136	3.2171481	0.0012947	0.020378607
TCN2	12.7478469	1.361531053	0.46841	2.9067073	0.0036525	0.045507299
GIMAP7	12.8456313	1.314902789	0.440875	2.982486	0.0028592	0.037109455
GNPDA1	18.2588562	1.281451096	0.340022	3.7687322	0.0001641	0.003814073
RAB32	20.7681401	1.28102525	0.338902	3.7799284	0.0001569	0.00366458
SCPEP1	16.9556142	1.280510751	0.41846	3.0600525	0.002213	0.030950922
TNF	14.2056536	1.270551309	0.401283	3.1662259	0.0015443	0.023425897
FKBP15	13.3409517	1.264078106	0.397941	3.1765431	0.0014904	0.022853781
GIMAP4	20.6732845	1.216609146	0.357761	3.4006158	0.0006723	0.012320555
CD14	156.880819	1.211848572	0.180893	6.6992496	2.09E-11	2.77E-09
MYO1F	15.7480005	1.173885648	0.35929	3.2672352	0.001086	0.01770447
PIK3AP1	15.3358557	1.148751544	0.394921	2.9088137	0.003628	0.045314273
CUX1	15.1253811	1.097749515	0.370219	2.9651329	0.0030255	0.038951661
NCF1	40.4402027	1.086232183	0.28678	3.7876816	0.0001521	0.003618441
PLEK	80.0095836	1.04575011	0.28044	3.7289638	0.0001923	0.004428355
NTAN1	17.1382664	1.032053133	0.356832	2.8922652	0.0038247	0.047184433
VAMP5	39.3158522	1.030095273	0.29882	3.447206	0.0005664	0.010813602
FBP1	23.1142026	1.019710765	0.347575	2.933784	0.0033486	0.042565004
IL10RA	23.9417121	1.005454218	0.345455	2.9105213	0.0036083	0.045179784
APBB1IP	24.1191134	0.979634765	0.294642	3.3248265	0.0008847	0.015478251
PRDX1	98.3957668	0.955515498	0.207258	4.6102791	4.02E-06	0.000164154
TUBB	43.3191915	0.952584298	0.257033	3.7060827	0.0002105	0.004744822
PLTP	61.3563837	0.949643089	0.291211	3.2610088	0.0011102	0.0180393
BMP2K	27.4212946	0.943840446	0.300265	3.1433574	0.0016702	0.024797261
FTL	6184.46987	0.930410879	0.1878	4.954275	7.26E-07	3.44E-05

FCGR3A	93.6197674	0.928766091	0.172228	5.3926671	6.94E-08	4.36E-06
NCKAP1L	30.3605211	0.911687982	0.265878	3.4289774	0.0006059	0.01141885
EPB41L2	31.5810356	0.896600002	0.285378	3.141794	0.0016792	0.024797261
FYB1	61.419508	0.781269884	0.226899	3.4432486	0.0005748	0.010931528
PSAP	411.467552	0.759869238	0.172392	4.4077888	1.04E-05	0.000374535
CTSZ	169.576936	0.735200209	0.182088	4.0376125	5.40E-05	0.001497921
CYBB	122.176887	0.729769599	0.145691	5.0090356	5.47E-07	2.72E-05
LGALS1	234.256624	0.702413312	0.156728	4.4817355	7.40E-06	0.000285959
MNDA	63.7866726	0.673748255	0.213046	3.1624537	0.0015645	0.023588989
C1QC	323.43973	0.649169742	0.130044	4.9919128	5.98E-07	2.94E-05
C1QB	624.319473	0.61636627	0.153234	4.0223874	5.76E-05	0.001555192
MARCKS	182.563508	0.60144189	0.191382	3.1426195	0.0016744	0.024797261
VAMP8	119.690427	0.59911051	0.16075	3.7269658	0.0001938	0.004443212
CSTB	94.6120845	0.596114897	0.206056	2.8929815	0.003816	0.047184433
TXNIP	150.136319	0.592483107	0.141292	4.193312	2.75E-05	0.000821624
NPC2	266.367139	0.486376997	0.119207	4.0800938	4.50E-05	0.001284278
MT-ND5	248.491619	0.44937089	0.155473	2.8903432	0.0038482	0.047357555
MT-ND1	750.079	0.414948912	0.123242	3.3669431	0.0007601	0.013727627
MT-CYB	874.855816	0.404918775	0.109188	3.7084686	0.0002085	0.004737383
MT-ND4	790.551421	0.372461245	0.121787	3.0582929	0.002226	0.030960778
HLA-B	512.267712	0.366320625	0.093141	3.9329486	8.39E-05	0.00218297
MT-ATP6	1664.97728	0.362941894	0.093183	3.8949264	9.82E-05	0.002441612
MT-CO1	1652.18294	0.349190954	0.11507	3.0345901	0.0024086	0.03290789
RPL21	586.744026	-0.350890455	0.111732	-3.140468	0.0016868	0.02483039
RPS15	430.542144	-0.35710621	0.119266	-2.994198	0.0027517	0.036167915
RPL37	495.101979	-0.374171326	0.12462	-3.002509	0.0026776	0.035720466
RPL14	312.350511	-0.375414273	0.117124	-3.205262	0.0013494	0.021041355
MALAT1	6868.79391	-0.390111364	0.11504	-3.391098	0.0006961	0.012710122
RPS9	534.59618	-0.396257449	0.110215	-3.595315	0.000324	0.006722309
RPL35A	316.398262	-0.399615359	0.126252	-3.165213	0.0015497	0.023436818
RPS27	727.251914	-0.399967464	0.123205	-3.24637	0.0011689	0.018871
RPL39	552.930702	-0.400780058	0.127969	-3.131842	0.0017371	0.025281595
PABPC1	195.332941	-0.404609672	0.134891	-2.999535	0.0027039	0.035821596
RPS15A	441.182089	-0.409069929	0.128538	-3.182484	0.0014602	0.022489414
RPL11	569.967518	-0.414931724	0.126073	-3.291208	0.0009976	0.016585599
TYROBP	413.416865	-0.419489682	0.110621	-3.792147	0.0001494	0.003587978
RPL41	843.167083	-0.420293575	0.097205	-4.323793	1.53E-05	0.000509978
RPL36	337.278872	-0.422685226	0.124012	-3.408411	0.0006534	0.012017747
FAU	428.73873	-0.4237521	0.115321	-3.67456	0.0002383	0.00524694
RPS24	696.533566	-0.427682196	0.106123	-4.030043	5.58E-05	0.001530852
RPL38	271.695791	-0.433942553	0.129945	-3.339431	0.0008395	0.014842064
RPS29	443.543355	-0.435734476	0.115278	-3.779858	0.0001569	0.00366458
LAPTM5	175.546505	-0.438093102	0.139759	-3.13464	0.0017206	0.025114481
PTMA	654.019643	-0.43955225	0.143225	-3.068955	0.0021481	0.030381826
RPS3	335.809456	-0.448600057	0.136633	-3.283254	0.0010262	0.016948557
CD74	1516.18955	-0.451412245	0.115437	-3.910478	9.21E-05	0.002324134
RPL30	604.843836	-0.469502214	0.108552	-4.32512	1.52E-05	0.000509978
RPS13	448.416339	-0.477477698	0.113822	-4.194934	2.73E-05	0.000821624
HLA-DRB1	1583.07189	-0.481774902	0.135336	-3.559832	0.0003711	0.007592221
CLIC1	125.901387	-0.490657709	0.164129	-2.989462	0.0027947	0.036542047
RPL34	568.408615	-0.4927071	0.112428	-4.38244	1.17E-05	0.000412064
DDX5	251.800343	-0.504906563	0.116197	-4.345273	1.39E-05	0.000475125

TIMP1	170.733689	-0.51150888	0.162584	-3.146128	0.0016545	0.024650217
VIM	646.346588	-0.525887689	0.099466	-5.28713	1.24E-07	7.52E-06
RPL26	578.102001	-0.526853437	0.110353	-4.774249	1.80E-06	8.01E-05
HLA-DMA	190.93517	-0.553074879	0.142051	-3.893491	9.88E-05	0.002444017
TAGLN2	84.2195154	-0.555117286	0.179522	-3.092196	0.0019868	0.028421106
FTH1	3230.2997	-0.569748135	0.143207	-3.978494	6.94E-05	0.001842444
FCGR2B	70.9945012	-0.571368461	0.185843	-3.074472	0.0021088	0.029909814
EIF1	588.321255	-0.572087099	0.102756	-5.567434	2.59E-08	1.78E-06
JUND	255.635319	-0.58696494	0.193928	-3.026717	0.0024723	0.033549104
SERP1	65.7756182	-0.588776018	0.201381	-2.923687	0.0034591	0.043748788
RPS16	466.066532	-0.590944603	0.116499	-5.072514	3.93E-07	2.12E-05
SRSF5	88.9940056	-0.595988537	0.171462	-3.475927	0.0005091	0.009946098
SRSF3	92.3188961	-0.620450958	0.170717	-3.634373	0.0002787	0.005928547
DNAJB6	63.0914299	-0.638062943	0.201094	-3.172962	0.0015089	0.023028299
RPS21	198.882595	-0.64718545	0.148046	-4.37151	1.23E-05	0.000429107
COMMD6	71.0809026	-0.655834106	0.18507	-3.543713	0.0003945	0.007987731
LYZ	517.140438	-0.657890424	0.17753	-3.705789	0.0002107	0.004744822
SELENOP	349.97004	-0.659423621	0.156667	-4.209068	2.56E-05	0.000780313
ZFP36L2	144.906009	-0.677459171	0.152741	-4.435359	9.19E-06	0.000339357
REL	79.3192292	-0.681442826	0.230032	-2.962377	0.0030527	0.039101467
DDX3X	70.2923249	-0.693862749	0.199382	-3.480073	0.0005013	0.009831697
CD44	76.651985	-0.69786121	0.212281	-3.28744	0.001011	0.016753663
KLF4	94.2659746	-0.700087675	0.2047	-3.420075	0.000626	0.011583858
PABPC4	56.6492056	-0.703685898	0.213322	-3.298697	0.0009713	0.016203073
CST3	1778.83044	-0.710717127	0.161642	-4.396861	1.10E-05	0.000388342
HLA-DRA	1936.2328	-0.718273584	0.130545	-5.502105	3.75E-08	2.51E-06
MRC1	108.495453	-0.728653153	0.185621	-3.925488	8.66E-05	0.002228653
H3F3A	305.573436	-0.739582316	0.119125	-6.208452	5.35E-10	5.60E-08
RSRP1	45.100744	-0.745632204	0.241403	-3.088742	0.0020101	0.028671894
LITAF	56.7950387	-0.746849085	0.229311	-3.256933	0.0011262	0.018241326
ELF1	42.1333799	-0.754537826	0.252999	-2.98237	0.0028603	0.037109455
RPS10	246.957423	-0.761049214	0.161534	-4.711388	2.46E-06	0.000105585
H3F3B	374.556008	-0.780240229	0.127654	-6.112155	9.83E-10	9.87E-08
SERTAD1	51.1320115	-0.784965511	0.231865	-3.385439	0.0007106	0.012881409
TUBB4B	76.2925965	-0.789943688	0.214087	-3.689823	0.0002244	0.005007838
SARAF	63.604707	-0.790794627	0.218366	-3.62142	0.000293	0.006155244
BTG1	178.810563	-0.792473029	0.159054	-4.982414	6.28E-07	3.03E-05
UBC	423.657797	-0.796595617	0.131913	-6.038778	1.55E-09	1.39E-07
GADD45B	175.470599	-0.807707952	0.165722	-4.873881	1.09E-06	4.99E-05
HLA-DPB1	715.922326	-0.816453302	0.161013	-5.070716	3.96E-07	2.12E-05
BNIP3L	36.5783355	-0.832023734	0.273594	-3.041092	0.0023572	0.032426245
TOP1	41.6609613	-0.833034583	0.247619	-3.364173	0.0007677	0.01380936
CPVL	81.4063236	-0.835890073	0.193825	-4.312593	1.61E-05	0.000529506
ZFP36	222.500285	-0.839219837	0.133787	-6.272824	3.55E-10	3.96E-08
C1orf162	119.020137	-0.841167927	0.188558	-4.461068	8.16E-06	0.000307875
HCST	74.9805394	-0.841854147	0.191355	-4.399442	1.09E-05	0.000386473
ATF4	33.5949895	-0.843124753	0.267829	-3.147996	0.0016439	0.024566122
HMOX1	105.528872	-0.8439674	0.196542	-4.294084	1.75E-05	0.000566781
IQGAP2	41.1744803	-0.852627393	0.266222	-3.202696	0.0013615	0.021163999
MS4A7	156.395697	-0.854027159	0.169581	-5.036093	4.75E-07	2.43E-05
HLA-DQB1	380.594348	-0.85723513	0.152781	-5.61089	2.01E-08	1.46E-06
MIDN	61.2773093	-0.866076764	0.205336	-4.217858	2.47E-05	0.000759724

SGK1	104.866675	-0.877736745	0.198521	-4.421375	9.81E-06	0.000354572
HCLS1	43.7132024	-0.885384467	0.240179	-3.686355	0.0002275	0.005054073
HNRNPH1	52.437671	-0.89003675	0.221951	-4.010062	6.07E-05	0.001629889
VAPA	48.6602401	-0.915138505	0.225282	-4.062186	4.86E-05	0.001371336
SNU13	35.9074118	-0.917706722	0.289747	-3.167271	0.0015388	0.023412559
MFSD1	36.2792411	-0.918269614	0.276422	-3.321984	0.0008938	0.015582495
EIF4A3	39.4514516	-0.919869853	0.295983	-3.107842	0.0018846	0.02703575
CKLF	42.6582085	-0.922980403	0.286707	-3.219248	0.0012853	0.020293545
PTPRE	39.2094279	-0.926169643	0.25431	-3.641898	0.0002706	0.005807093
DDX21	45.7939771	-0.93495733	0.232319	-4.024461	5.71E-05	0.001555192
FABP5	61.0857883	-0.936044735	0.321236	-2.913881	0.0035697	0.045016747
HBEGF	66.6858817	-0.938919247	0.270333	-3.473197	0.0005143	0.010008864
CHD1	39.2416145	-0.947874664	0.258346	-3.669011	0.0002435	0.005300331
HLA-DPA1	856.097258	-0.952623719	0.163404	-5.829878	5.55E-09	4.72E-07
RAB31	43.6853821	-0.954788939	0.258618	-3.691887	0.0002226	0.004989528
CELF2	62.9528927	-0.962999329	0.206088	-4.67275	2.97E-06	0.000125396
CDC42SE1	23.3496235	-0.963553835	0.334156	-2.883546	0.0039323	0.048273484
SAT1	418.736067	-0.973907386	0.174225	-5.589926	2.27E-08	1.60E-06
FCGR2A	78.6830141	-0.99162444	0.195648	-5.068416	4.01E-07	2.12E-05
SRSF7	92.1055169	-0.995002591	0.231198	-4.303676	1.68E-05	0.000547704
MAFF	24.6531202	-0.996796354	0.312102	-3.193811	0.0014041	0.021758918
DNTTIP2	22.8954058	-0.998547726	0.339137	-2.944382	0.003236	0.041238444
LGMN	95.7434977	-0.999221154	0.172379	-5.796667	6.76E-09	5.57E-07
PMAIP1	33.8369087	-0.999930673	0.302725	-3.303096	0.0009562	0.016057715
ANXA1	190.198544	-1.007735486	0.166061	-6.068481	1.29E-09	1.25E-07
SRSF2	50.0455392	-1.012945696	0.251356	-4.029924	5.58E-05	0.001530852
KMT2E	30.9778829	-1.018302576	0.297799	-3.419429	0.0006275	0.011583858
CTNNB1	35.4770112	-1.029218765	0.272285	-3.77993	0.0001569	0.00366458
RBM39	66.6288861	-1.040642285	0.203974	-5.10184	3.36E-07	1.85E-05
FCN1	51.6546441	-1.068322683	0.304305	-3.510692	0.0004469	0.008869949
CEBPB	145.732517	-1.079138806	0.181334	-5.951125	2.66E-09	2.31E-07
SOCS3	44.0165969	-1.084737387	0.262463	-4.132916	3.58E-05	0.001045624
SELENOK	49.1275685	-1.090210495	0.251143	-4.34099	1.42E-05	0.000477979
RAB21	20.8231899	-1.100640466	0.366635	-3.002007	0.0026821	0.035720466
NR4A3	35.369964	-1.103702289	0.300784	-3.669418	0.0002431	0.005300331
CDKN1A	50.4208969	-1.106311798	0.253127	-4.370574	1.24E-05	0.000429107
SRGAP2B	19.1093822	-1.107809852	0.362162	-3.058882	0.0022216	0.030960778
RGS2	93.0100319	-1.116744831	0.246941	-4.522323	6.12E-06	0.000238068
IRS2	25.461342	-1.117823595	0.33832	-3.304041	0.000953	0.016057422
CD86	42.2457257	-1.121529648	0.263674	-4.253469	2.10E-05	0.000664681
ADGRE5	37.1540143	-1.12827922	0.276116	-4.086253	4.38E-05	0.001265051
MAN1A1	24.0974053	-1.131783949	0.350367	-3.230281	0.0012367	0.019712391
RGS1	38.8291377	-1.136306585	0.376328	-3.019457	0.0025323	0.034178994
IFITM3	99.2023036	-1.141919146	0.196589	-5.808674	6.30E-09	5.27E-07
TNFRSF1B	52.8711659	-1.143321074	0.232813	-4.910905	9.07E-07	4.21E-05
RDX	18.0325136	-1.151846461	0.375989	-3.063515	0.0021875	0.030766427
CSF2RA	19.8331019	-1.160948549	0.354909	-3.271119	0.0010712	0.017520009
RHOB	78.3549891	-1.162814735	0.270938	-4.291807	1.77E-05	0.000566781
MYADM	38.5379695	-1.16710444	0.267939	-4.355866	1.33E-05	0.000455816
TTN	20.7407782	-1.168227333	0.369825	-3.158867	0.0015838	0.02373863
PDK4	84.9185729	-1.171794841	0.20517	-5.711349	1.12E-08	8.66E-07
MCL1	121.327081	-1.181476504	0.174941	-6.753582	1.44E-11	1.96E-09

ZFAND5	83.2873949	-1.19727027	0.21183	-5.652034	1.59E-08	1.17E-06
TSPYL2	21.5785969	-1.20203709	0.394047	-3.050494	0.0022847	0.031601222
SRGAP2	19.5138819	-1.202257313	0.362419	-3.31731	0.0009089	0.015628513
CHMP1B	31.7687732	-1.203717748	0.311115	-3.869045	0.0001093	0.002676127
SLC2A3	51.0071471	-1.204206833	0.26171	-4.601305	4.20E-06	0.000170006
TBC1D9	14.8406174	-1.218949412	0.418715	-2.91117	0.0036008	0.045179784
KDM6B	57.9615811	-1.222898069	0.230674	-5.301417	1.15E-07	7.04E-06
HLA-DQA1	400.022199	-1.229556761	0.141358	-8.698203	3.37E-18	7.70E-16
ETS2	48.2700237	-1.243791859	0.247918	-5.016941	5.25E-07	2.64E-05
SRGN	758.088353	-1.250758868	0.122006	-10.25165	1.16E-24	6.49E-22
DBI	121.838104	-1.251911709	0.206045	-6.075924	1.23E-09	1.21E-07
MT1X	468.530179	-1.254404441	0.133305	-9.410011	4.96E-21	1.66E-18
CD9	27.9081626	-1.258011511	0.353453	-3.559208	0.000372	0.007592221
JAML	29.0086683	-1.27180344	0.362611	-3.50735	0.0004526	0.008946725
SMAP2	47.4640608	-1.275163575	0.245661	-5.19075	2.09E-07	1.21E-05
ATP6V1B2	21.4545896	-1.281312845	0.354062	-3.618894	0.0002959	0.006189728
ZNF331	68.2014246	-1.303210281	0.233194	-5.58852	2.29E-08	1.60E-06
PELI1	19.6953051	-1.303822998	0.377254	-3.456091	0.0005481	0.010503287
GPR183	84.1915834	-1.30864275	0.248709	-5.261737	1.43E-07	8.53E-06
HEXIM1	28.5053102	-1.309717006	0.33278	-3.935684	8.30E-05	0.002169496
GPCPD1	14.8941328	-1.320332902	0.43504	-3.034972	0.0024056	0.03290789
PNP	22.7080812	-1.326608435	0.350077	-3.789475	0.000151	0.003609519
PLAUR	107.91468	-1.328750938	0.182755	-7.270687	3.58E-13	5.61E-11
JDP2	16.34816	-1.335341182	0.388051	-3.441148	0.0005793	0.010975192
TLR2	19.3549392	-1.337039796	0.366282	-3.650303	0.0002619	0.00564445
DUSP5	20.2869391	-1.337767741	0.368352	-3.631763	0.0002815	0.005945925
SMCHD1	26.4894359	-1.337811812	0.334427	-4.000311	6.33E-05	0.001689495
PIM3	25.4787561	-1.347025317	0.393038	-3.427213	0.0006098	0.01141885
PDCL3	14.8660172	-1.356774186	0.410278	-3.306964	0.0009431	0.015944275
DDX27	18.2033404	-1.363211837	0.376964	-3.616296	0.0002988	0.006226215
HERPUD1	94.2470653	-1.369593581	0.209895	-6.525131	6.79E-11	8.12E-09
SATB1	14.3903143	-1.370980013	0.438987	-3.123052	0.0017899	0.025973672
CTTNBP2NL	12.210062	-1.373605709	0.471509	-2.913212	0.0035773	0.045016747
AKAP13	78.0156088	-1.373857232	0.20614	-6.664673	2.65E-11	3.41E-09
GLIPR2	20.4745029	-1.374199209	0.362004	-3.796091	0.000147	0.003548394
MAP2K1	19.3422837	-1.374825663	0.369911	-3.716634	0.0002019	0.004607782
AXL	14.033232	-1.39281585	0.466325	-2.986792	0.0028192	0.036767012
RASD1	16.5853789	-1.397013942	0.391406	-3.569219	0.000358	0.007367843
RGCC	83.5899096	-1.406440794	0.464538	-3.027611	0.002465	0.033540752
HGSNAT	11.6747069	-1.41133484	0.466524	-3.025214	0.0024846	0.033625458
RNF149	33.7385729	-1.413659356	0.317775	-4.44862	8.64E-06	0.000321432
ARL4C	39.711478	-1.416625623	0.300373	-4.716221	2.40E-06	0.000104291
TGIF1	17.9750983	-1.416680286	0.439009	-3.226999	0.001251	0.019876793
PQLC1	13.0020337	-1.423764352	0.442908	-3.214586	0.0013063	0.020497119
MT1H	15.365766	-1.430674147	0.474544	-3.014842	0.0025711	0.034610377
PER1	21.818913	-1.433274068	0.366159	-3.914353	9.06E-05	0.002303868
PID1	11.5963247	-1.433477242	0.468352	-3.060686	0.0022083	0.030950922
B4GALT1	32.0155312	-1.436904307	0.307472	-4.673292	2.96E-06	0.000125396
NFIL3	14.1286085	-1.45218953	0.437322	-3.320643	0.0008981	0.015593305
SPTLC2	14.760917	-1.455402962	0.429743	-3.38668	0.0007074	0.012869747
FGR	12.6267409	-1.468571903	0.45545	-3.224442	0.0012622	0.019991868
AC016831.5	9.7212788	-1.476514032	0.508492	-2.903713	0.0036877	0.045831031

TREM1	15.3002339	-1.496290787	0.428821	-3.489312	0.0004843	0.0095353
BCAT1	10.7821084	-1.50427839	0.501825	-2.997614	0.002721	0.035953302
PHF20	19.7480994	-1.507960861	0.385292	-3.913809	9.09E-05	0.002303868
CCND2	10.4110884	-1.512764506	0.515986	-2.931796	0.0033701	0.042730172
PHLDA2	16.6966493	-1.514292846	0.48696	-3.109689	0.0018728	0.026995715
GNA13	25.9922124	-1.541006196	0.344609	-4.47175	7.76E-06	0.000297358
CPM	36.641077	-1.561546898	0.292548	-5.337751	9.41E-08	5.83E-06
FILIP1L	35.0525008	-1.567864192	0.311943	-5.02612	5.01E-07	2.54E-05
PPP1CB	50.9655573	-1.569626855	0.255465	-6.144203	8.04E-10	8.24E-08
SLA	23.0701226	-1.571025743	0.351593	-4.468302	7.88E-06	0.000299902
ADM	33.6589474	-1.576531043	0.323336	-4.875836	1.08E-06	4.99E-05
RHOU	8.88800126	-1.602187275	0.55362	-2.894021	0.0038034	0.047153068
PTK2B	10.718736	-1.613043917	0.522965	-3.084423	0.0020395	0.029009086
PLSCR1	38.7185903	-1.614311366	0.294582	-5.48001	4.25E-08	2.81E-06
GPAT4	9.45171165	-1.615099067	0.538277	-3.0005	0.0026954	0.035802733
MXD1	28.802148	-1.616266498	0.310393	-5.207159	1.92E-07	1.12E-05
SERINC5	13.1263274	-1.619542807	0.472674	-3.426344	0.0006118	0.01141885
SRGAP2C	16.2011269	-1.62803496	0.424428	-3.83583	0.0001251	0.003035419
USP36	9.4756999	-1.632008216	0.535306	-3.048737	0.0022981	0.031699277
MIR22HG	29.8742742	-1.635293953	0.341349	-4.79068	1.66E-06	7.45E-05
SLC25A37	12.4845191	-1.641507045	0.464891	-3.530953	0.0004141	0.008316085
STK17B	46.9522699	-1.641888123	0.263071	-6.241241	4.34E-10	4.74E-08
CHSY1	9.48856199	-1.648045388	0.530067	-3.109126	0.0018764	0.026995715
CXCL16	52.2580458	-1.648452667	0.251359	-6.558168	5.45E-11	6.67E-09
XBP1	22.7724521	-1.652495109	0.370671	-4.458124	8.27E-06	0.000309803
SLC31A2	23.1631418	-1.670090704	0.346928	-4.813941	1.48E-06	6.69E-05
CEBPD	163.448263	-1.682711928	0.210832	-7.981309	1.45E-15	2.51E-13
TES	13.733596	-1.684143395	0.457996	-3.677205	0.0002358	0.005215731
ILF2	8.65100721	-1.69091392	0.572689	-2.952587	0.0031512	0.040260398
CXCR4	32.6547608	-1.690972911	0.342956	-4.930578	8.20E-07	3.85E-05
ABL2	18.7304442	-1.708783544	0.407472	-4.193618	2.75E-05	0.000821624
FOSL2	24.7344629	-1.71778227	0.339034	-5.066701	4.05E-07	2.12E-05
FNIP2	11.793527	-1.734660222	0.491578	-3.528755	0.0004175	0.008352043
CHKA	9.4103915	-1.74728696	0.539191	-3.240573	0.0011929	0.019197259
ARRDC3	29.0967877	-1.751400547	0.319918	-5.474536	4.39E-08	2.86E-06
IFITM2	61.171827	-1.753183859	0.224472	-7.810268	5.71E-15	9.55E-13
GABARAPL1	16.6899143	-1.768537528	0.41678	-4.243333	2.20E-05	0.00069109
MAP3K8	39.4839885	-1.778611472	0.307819	-5.778113	7.55E-09	6.12E-07
NDEL1	9.36453986	-1.784338563	0.540517	-3.30117	0.0009628	0.016114514
MPHOSPH6	11.5399073	-1.808900341	0.498122	-3.631442	0.0002818	0.005945925
AGO2	14.4047206	-1.811405259	0.446574	-4.056224	4.99E-05	0.001398936
JARID2	18.1650923	-1.81769548	0.400492	-4.538655	5.66E-06	0.00022403
RNF144B	15.4441634	-1.821602427	0.430887	-4.227564	2.36E-05	0.000732184
NAMPT	150.022069	-1.851234322	0.176942	-10.46238	1.29E-25	8.07E-23
TRMT6	9.20975777	-1.85457507	0.560565	-3.308406	0.0009383	0.015916011
SH3TC1	12.7540022	-1.854775227	0.475373	-3.901724	9.55E-05	0.002385854
CCNH	26.9440458	-1.858921106	0.354056	-5.250366	1.52E-07	8.97E-06
EMP1	49.1155733	-1.8687269	0.275128	-6.792198	1.10E-11	1.54E-09
UPP1	20.2376178	-1.86884861	0.404408	-4.621194	3.82E-06	0.000157024
SNX9	24.9278072	-1.880129464	0.367636	-5.114113	3.15E-07	1.76E-05
DGKD	9.64334699	-1.892096404	0.569933	-3.319859	0.0009006	0.015593305
VMO1	21.1529739	-1.897081154	0.419348	-4.523885	6.07E-06	0.000238068

CCDC152	7.30513933	-1.903642239	0.64226	-2.963976	0.0030369	0.038998395
SERPINB9	20.6421121	-1.909003574	0.385172	-4.956233	7.19E-07	3.44E-05
AVPI1	9.46770364	-1.915049519	0.559572	-3.422345	0.0006208	0.011545213
CDK2AP1	7.10981632	-1.917658828	0.636568	-3.012496	0.0025911	0.034785741
SLC26A2	6.97986256	-1.919294649	0.667164	-2.876794	0.0040174	0.049198137
NDRG1	11.5875704	-1.920134021	0.525855	-3.651452	0.0002608	0.005643464
MT2A	1832.75599	-1.928187828	0.133028	-14.49458	1.31E-47	2.19E-44
CD300E	8.23741215	-1.936699061	0.606801	-3.191654	0.0014146	0.02185456
MT1F	9.56647703	-1.940873659	0.633425	-3.064092	0.0021833	0.030766427
FGL2	174.132802	-1.944323219	0.191915	-10.13118	4.02E-24	2.02E-21
HSPA2	11.8000986	-1.992943003	0.526926	-3.782208	0.0001554	0.00366458
RARA	8.2106802	-1.993953494	0.621404	-3.20879	0.0013329	0.020849628
C5AR1	85.34913	-2.009430047	0.202643	-9.916127	3.54E-23	1.62E-20
EZR	84.3640389	-2.02705217	0.22934	-8.838628	9.69E-19	2.43E-16
SIPA1L1	12.8678734	-2.034605004	0.493104	-4.126115	3.69E-05	0.001070785
TUBB6	14.1033975	-2.046521335	0.462889	-4.42119	9.82E-06	0.000354572
FKBP5	33.5948132	-2.061842483	0.322709	-6.389164	1.67E-10	1.90E-08
C1orf56	10.6804772	-2.096334006	0.540384	-3.87934	0.0001047	0.002577946
AC016831.1	6.28538669	-2.112364169	0.705428	-2.994444	0.0027495	0.036167915
SNHG15	14.9799509	-2.118407096	0.521147	-4.064891	4.81E-05	0.001363184
MAP4K4	7.60020703	-2.125750962	0.681521	-3.119127	0.0018139	0.026246298
MAT2A	60.9033676	-2.129871689	0.264852	-8.041747	8.86E-16	1.59E-13
IRAK3	22.4170839	-2.160154458	0.418542	-5.161143	2.45E-07	1.40E-05
PAPOLG	6.33812725	-2.162534855	0.722638	-2.992555	0.0027665	0.036268223
METRNL	76.2442398	-2.171686002	0.235234	-9.232041	2.66E-20	7.84E-18
PFKFB3	21.3700948	-2.177239947	0.402135	-5.414207	6.16E-08	3.91E-06
THBD	24.8844219	-2.185877583	0.426769	-5.121916	3.02E-07	1.71E-05
NEDD9	9.48751761	-2.206008111	0.574789	-3.837943	0.0001241	0.003024039
TSC22D3	69.6737025	-2.208270622	0.228529	-9.662989	4.33E-22	1.67E-19
P2RY14	10.3185807	-2.219773556	0.568321	-3.905846	9.39E-05	0.002357267
LGALS2	16.5545229	-2.242123528	0.530074	-4.229833	2.34E-05	0.000729338
TENT4B	7.47283831	-2.253544899	0.673051	-3.348255	0.0008132	0.014530929
ACSL3	28.1188183	-2.274931474	0.350146	-6.497092	8.19E-11	9.56E-09
ITGAX	31.6253881	-2.297080176	0.346581	-6.62784	3.41E-11	4.28E-09
CLEC4E	17.3575707	-2.308754971	0.45805	-5.040404	4.65E-07	2.40E-05
CEP68	6.12309931	-2.326544616	0.741214	-3.13883	0.0016962	0.02483039
SRGAP1	16.3939114	-2.37038894	0.464702	-5.100883	3.38E-07	1.85E-05
KLF9	21.002781	-2.378043832	0.419892	-5.663466	1.48E-08	1.13E-06
LMNA	180.053624	-2.386863679	0.195462	-12.2114	2.70E-34	3.39E-31
RAD51C	6.31223969	-2.417064245	0.764612	-3.161164	0.0015714	0.023622753
IRF7	11.5496555	-2.420638813	0.557459	-4.342274	1.41E-05	0.000477979
ATP13A3	20.4007073	-2.435829167	0.426458	-5.711769	1.12E-08	8.66E-07
GPAT3	16.3470015	-2.456563991	0.520939	-4.715648	2.41E-06	0.000104291
VEGFA	66.8488893	-2.494163332	0.273247	-9.127869	6.99E-20	1.85E-17
CRYBG1	17.6084371	-2.504539117	0.452199	-5.538576	3.05E-08	2.07E-06
BCL2	7.1246282	-2.542960795	0.719223	-3.535706	0.0004067	0.008200719
DNMBP	8.06268796	-2.549956098	0.678807	-3.756525	0.0001723	0.003986473
UBASH3B	5.82799812	-2.560085874	0.791453	-3.234667	0.0012178	0.019490465
CREM	80.5703942	-2.565466242	0.230398	-11.13491	8.48E-29	6.08E-26
PHLDA1	20.6089149	-2.574044425	0.429103	-5.998657	1.99E-09	1.75E-07
NET1	6.14297963	-2.598150431	0.792841	-3.277013	0.0010491	0.017270876
SAMSN1	64.3884255	-2.625392696	0.265113	-9.902926	4.04E-23	1.69E-20

SYAP1	50.9874395	-2.639363763	0.287854	-9.169121	4.77E-20	1.33E-17
PDE4A	11.939692	-2.661872064	0.616201	-4.319809	1.56E-05	0.000515856
SH3BP5	43.9521932	-2.662500933	0.324152	-8.213753	2.14E-16	4.14E-14
SDS	21.1719217	-2.677058044	0.442508	-6.049733	1.45E-09	1.32E-07
INSIG1	54.2910836	-2.681333963	0.288826	-9.283556	1.64E-20	5.14E-18
RILPL2	58.6884731	-2.682641271	0.279402	-9.601353	7.89E-22	2.83E-19
GNA12	6.7492606	-2.707269134	0.780758	-3.467487	0.0005253	0.010184463
FOXK2	5.15132845	-2.710890571	0.89345	-3.034182	0.0024119	0.03290789
GK	10.3516238	-2.733353644	0.679377	-4.023326	5.74E-05	0.001555192
AMPD2	8.34885287	-2.743957125	0.695979	-3.942584	8.06E-05	0.002119026
SDC2	9.12615873	-2.746853365	0.659089	-4.167649	3.08E-05	0.000903655
FABP4	17.7851791	-2.751362995	0.900178	-3.056466	0.0022396	0.031063984
VDR	12.5056768	-2.754495178	0.590155	-4.667409	3.05E-06	0.000127626
ATP1B3	117.861938	-2.762370741	0.230698	-11.97394	4.87E-33	4.89E-30
GRASP	42.8792729	-2.854536769	0.323763	-8.81676	1.18E-18	2.82E-16
HES4	5.6454431	-2.862528381	0.911879	-3.139153	0.0016944	0.02483039
MT1M	54.8593054	-2.879241986	0.634409	-4.538463	5.67E-06	0.00022403
KLHL6	6.08116315	-2.903864938	0.839036	-3.460953	0.0005383	0.010394768
CCNE2	5.65938019	-2.936354687	0.878279	-3.343304	0.0008279	0.014688128
NLRP3	30.6075355	-3.013753069	0.421185	-7.155421	8.34E-13	1.23E-10
DUSP4	10.3668675	-3.046967399	0.709828	-4.292541	1.77E-05	0.000566781
CD1C	24.6630412	-3.089467534	0.49598	-6.229017	4.69E-10	5.01E-08
ANKRD28	8.51699978	-3.114378021	0.744906	-4.180899	2.90E-05	0.000857582
ACSL1	34.5104622	-3.130808144	0.383832	-8.15671	3.44E-16	6.40E-14
AFMID	5.50665039	-3.182916796	0.946359	-3.363327	0.0007701	0.01380936
TFRC	45.1636841	-3.219934392	0.376742	-8.546792	1.27E-17	2.65E-15
CD72	10.8489507	-3.24060229	0.683468	-4.741413	2.12E-06	9.35E-05
ELL2	35.2479581	-3.286725669	0.393234	-8.358203	6.37E-17	1.28E-14
SAP30	22.3799208	-3.402898011	0.492868	-6.904276	5.05E-12	7.24E-10
FCER1A	31.2479052	-3.410550411	0.472834	-7.212996	5.47E-13	8.33E-11
ISG20	18.2934761	-3.455002023	0.570756	-6.053382	1.42E-09	1.32E-07
LINC01315	4.59995477	-3.526279919	1.054728	-3.343308	0.0008279	0.014688128
IL1R1	9.47704586	-3.718400532	0.819072	-4.539771	5.63E-06	0.00022403
INSR	8.36803123	-3.798049074	0.906854	-4.188158	2.81E-05	0.000835529
TSC22D1	17.0422826	-3.95192374	0.651708	-6.063948	1.33E-09	1.26E-07
FLT1	6.6356896	-4.057676518	1.028423	-3.945534	7.96E-05	0.002104126
CD1E	4.83864483	-4.379488877	1.193766	-3.668633	0.0002439	0.005300331
CHRNE	5.05616446	-4.444562642	1.134783	-3.916662	8.98E-05	0.002300017
EREG	9.76984567	-4.656187329	1.003615	-4.639416	3.49E-06	0.000144985
IL1R2	29.1501018	-4.73920036	0.633669	-7.478987	7.49E-14	1.21E-11
AC108134.2	6.87025713	-4.904107932	1.201369	-4.082099	4.46E-05	0.001280523
HLA-DQA2	41.6247004	-5.061160144	0.584174	-8.663783	4.56E-18	9.96E-16
MMP19	4.79061818	-5.128110143	1.15685	-4.432821	9.30E-06	0.000340871
AREG	156.638595	-5.173096874	0.332498	-15.55829	1.40E-54	7.02E-51
PKP2	18.4142343	-5.629648365	0.975955	-5.76835	8.01E-09	6.38E-07
THBS1	84.7778715	-5.713301069	0.510238	-11.19733	4.20E-29	3.52E-26
HLA-DRB5	158.802411	-5.892494102	0.403838	-14.59123	3.19E-48	8.02E-45
CAVIN2	15.0113377	-6.04074865	1.079485	-5.595955	2.19E-08	1.57E-06
APOBEC3A	10.2465832	-6.220046681	1.099048	-5.659486	1.52E-08	1.14E-06

Supplementary Table S3c: Differentially expressed genes in VAT B cells between patients with or without CAC by pseudo-bulk analysis.

	baseMean	log2FC	lfcSE	stat	pvalue	padj	genename
HLA-DRB5	70.87888	-5.78355	0.328609	-17.6	2.46E-69	1.51E-65	HLA-DRB5
RNF144B	12.67319	-4.45078	0.520642	-8.5486	1.25E-17	2.64E-15	RNF144B
SYTL3	6.073943	-4.43909	0.746452	-5.9469	2.73E-09	2.24E-07	SYTL3
CXCL8	6.195305	-4.03455	0.863471	-4.6725	2.98E-06	0.00013	CXCL8
THEMIS2	14.83238	-3.0394	0.496177	-6.1256	9.03E-10	7.94E-08	THEMIS2
LMNA	56.17127	-2.98341	0.226058	-13.198	9.06E-40	6.97E-37	LMNA
WEE1	12.63477	-2.96668	0.472459	-6.2792	3.40E-10	3.27E-08	WEE1
S100A9	6.703318	-2.95891	0.601302	-4.9208	8.62E-07	4.17E-05	S100A9
DDX43	4.201332	-2.90104	0.791889	-3.6634	0.000249	0.006049	DDX43
ADGRE5	31.92223	-2.72357	0.287469	-9.4743	2.69E-21	9.18E-19	ADGRE5
RASSF6	12.33966	-2.47724	0.394075	-6.2862	3.25E-10	3.22E-08	RASSF6
EHD3	4.191894	-2.45141	0.750752	-3.2653	0.001094	0.020694	EHD3
PRDM1	6.106991	-2.44004	0.553692	-4.4069	1.05E-05	0.000384	PRDM1
SNX9	25.00644	-2.40605	0.276749	-8.694	3.50E-18	7.97E-16	SNX9
DNMBP	4.352703	-2.40271	0.642628	-3.7389	0.000185	0.004817	DNMBP
SC5D	12.75101	-2.34116	0.429078	-5.4563	4.86E-08	2.94E-06	SC5D
CAMKK1	4.705195	-2.31197	0.708902	-3.2613	0.001109	0.020854	CAMKK1
BCL2L11	26.17445	-2.29452	0.28237	-8.1259	4.44E-16	8.03E-14	BCL2L11
NTNG1	6.506924	-2.28713	0.530484	-4.3114	1.62E-05	0.000564	NTNG1
G0S2	5.331122	-2.22604	0.606042	-3.6731	0.00024	0.005943	G0S2
ZBTB16	6.790089	-2.20398	0.571259	-3.8581	0.000114	0.003209	ZBTB16
TP53INP1	15.29317	-2.13324	0.340471	-6.2656	3.72E-10	3.52E-08	TP53INP1
CENPM	9.510721	-2.12517	0.461447	-4.6054	4.12E-06	0.000171	CENPM
GNA12	10.80448	-2.11766	0.43081	-4.9155	8.85E-07	4.25E-05	GNA12
MT1F	96.03025	-2.11674	0.26131	-8.1005	5.47E-16	9.35E-14	MT1F
FKBP5	7.81486	-2.10308	0.503801	-4.1744	2.99E-05	0.000972	FKBP5
EMILIN2	5.762193	-2.09066	0.561054	-3.7263	0.000194	0.005021	EMILIN2
UFSP2	12.39345	-2.07145	0.38404	-5.3939	6.90E-08	4.08E-06	UFSP2
CEP126	4.986654	-2.06772	0.592616	-3.4891	0.000485	0.010644	CEP126
MT1E	129.9174	-2.04619	0.443981	-4.6087	4.05E-06	0.000169	MT1E
HLA-DQA2	9.906218	-2.03879	0.501329	-4.0668	4.77E-05	0.001473	HLA-DQA2
CCDC173	4.92225	-2.02673	0.673407	-3.0097	0.002615	0.04167	CCDC173
SRGN	119.6855	-1.96009	0.157573	-12.439	1.60E-35	9.84E-33	SRGN
RFX3	7.403309	-1.93937	0.528713	-3.6681	0.000244	0.005988	RFX3
CRIP1	96.85581	-1.90394	0.17028	-11.181	5.04E-29	2.58E-26	CRIP1
TSC22D3	224.45	-1.88277	0.119595	-15.743	7.69E-56	1.58E-52	TSC22D3
PFKFB3	5.14507	-1.86583	0.632134	-2.9516	0.003161	0.048	PFKFB3
SNTA1	5.93003	-1.86358	0.553804	-3.365	0.000765	0.015281	SNTA1
AC016831.1	10.53127	-1.86304	0.403356	-4.6188	3.86E-06	0.000164	AC016831.1
ZNF250	4.256412	-1.86193	0.624634	-2.9808	0.002875	0.044644	ZNF250
LDLRAD4	9.637945	-1.85709	0.496019	-3.744	0.000181	0.00476	LDLRAD4
JADE3	5.278739	-1.84654	0.570447	-3.237	0.001208	0.022109	JADE3
AC020916.1	15.84927	-1.84598	0.324011	-5.6973	1.22E-08	8.81E-07	AC020916.1
FAM76A	11.74819	-1.83743	0.374777	-4.9027	9.45E-07	4.47E-05	FAM76A
CEMIP2	38.81472	-1.81439	0.212947	-8.5204	1.59E-17	3.26E-15	CEMIP2
ALG2	4.82816	-1.81174	0.585782	-3.0929	0.001982	0.03331	ALG2
AC004687.1	23.04538	-1.8012	0.28958	-6.2201	4.97E-10	4.56E-08	AC004687.1
HIPK2	11.59673	-1.80066	0.3808	-4.7286	2.26E-06	0.000103	HIPK2

CA5B	7.014499	-1.79985	0.531794	-3.3845	0.000713	0.014379	CA5B
CENPL	20.94528	-1.79376	0.297887	-6.0216	1.73E-09	1.46E-07	CENPL
FAM49A	20.4483	-1.79156	0.31151	-5.7512	8.86E-09	6.65E-07	FAM49A
EZR	251.6116	-1.7889	0.106599	-16.782	3.33E-63	1.02E-59	EZR
GABPB1	14.93901	-1.77159	0.346652	-5.1106	3.21E-07	1.72E-05	GABPB1
COL4A3	5.817262	-1.76681	0.524959	-3.3656	0.000764	0.015281	COL4A3
CCDC167	10.69336	-1.73593	0.437664	-3.9664	7.30E-05	0.002137	CCDC167
PBXIP1	16.63406	-1.73546	0.313818	-5.5301	3.20E-08	2.10E-06	PBXIP1
RAB3IP	7.19143	-1.70891	0.505113	-3.3832	0.000716	0.014398	RAB3IP
TOGARAM1	7.392375	-1.70324	0.48463	-3.5145	0.000441	0.009889	TOGARAM1
RAPGEF2	5.445075	-1.69926	0.55605	-3.056	0.002243	0.036793	RAPGEF2
PELI1	18.99763	-1.6927	0.304581	-5.5575	2.74E-08	1.83E-06	PELI1
HMGCR	9.280847	-1.69101	0.426973	-3.9605	7.48E-05	0.00218	HMGCR
AGO2	17.76765	-1.68626	0.321526	-5.2445	1.57E-07	8.76E-06	AGO2
MT1X	1014.597	-1.68145	0.240724	-6.985	2.85E-12	3.65E-10	MT1X
SYNGR1	10.50334	-1.67513	0.396669	-4.223	2.41E-05	0.000797	SYNGR1
MRPL23	9.032322	-1.64189	0.450394	-3.6455	0.000267	0.006387	MRPL23
AHNAK	93.80354	-1.64096	0.149856	-10.95	6.63E-28	2.91E-25	AHNAK
LRRC75A	14.91001	-1.63825	0.334072	-4.9039	9.40E-07	4.47E-05	LRRC75A
ANKRD28	8.196439	-1.62538	0.498618	-3.2598	0.001115	0.020854	ANKRD28
SFMBT1	7.297908	-1.62277	0.4687	-3.4623	0.000536	0.011478	SFMBT1
CD55	157.0309	-1.62085	0.115583	-14.023	1.12E-44	1.15E-41	CD55
HEXIM1	88.84429	-1.61315	0.183091	-8.8106	1.24E-18	2.94E-16	HEXIM1
WDR74	17.91426	-1.61131	0.318427	-5.0602	4.19E-07	2.22E-05	WDR74
ACSF3	8.166604	-1.61044	0.463812	-3.4722	0.000516	0.011179	ACSF3
AC060780.1	6.019695	-1.59784	0.527908	-3.0267	0.002472	0.04001	AC060780.1
PEG10	12.46314	-1.57818	0.456391	-3.458	0.000544	0.011599	PEG10
PRDM4	7.516138	-1.56959	0.480197	-3.2686	0.001081	0.020577	PRDM4
SNX8	14.88127	-1.56105	0.393029	-3.9718	7.13E-05	0.002099	SNX8
CSKMT	41.75848	-1.5303	0.237175	-6.4522	1.10E-10	1.15E-08	CSKMT
ARID5A	8.514974	-1.51541	0.438287	-3.4576	0.000545	0.011599	ARID5A
PHF20	62.13737	-1.50303	0.169849	-8.8493	8.81E-19	2.36E-16	PHF20
TNFRSF13C	104.2998	-1.48861	0.146346	-10.172	2.65E-24	1.02E-21	TNFRSF13C
HIVEP1	22.40819	-1.48693	0.270234	-5.5024	3.75E-08	2.43E-06	HIVEP1
LRRFIP1	71.23806	-1.48669	0.161936	-9.1807	4.28E-20	1.25E-17	LRRFIP1
PDE4B	31.1684	-1.48357	0.23939	-6.1973	5.74E-10	5.19E-08	PDE4B
TLE1	14.76711	-1.47718	0.334605	-4.4147	1.01E-05	0.000376	TLE1
EGR3	19.65472	-1.46793	0.342571	-4.285	1.83E-05	0.000622	EGR3
CSRNP2	7.554435	-1.46619	0.491287	-2.9844	0.002842	0.044462	CSRNP2
CXCR4	167.7402	-1.45039	0.116146	-12.488	8.72E-36	5.96E-33	CXCR4
ZFP36L2	209.1586	-1.44707	0.09873	-14.657	1.22E-48	1.50E-45	ZFP36L2
RALGDS	9.973056	-1.43943	0.410504	-3.5065	0.000454	0.010117	RALGDS
IFIT2	26.56315	-1.43814	0.371521	-3.8709	0.000108	0.003059	IFIT2
SYAP1	26.31159	-1.41355	0.251903	-5.6115	2.01E-08	1.40E-06	SYAP1
RILPL2	31.66967	-1.40773	0.243943	-5.7708	7.89E-09	6.02E-07	RILPL2
STIM2	27.2977	-1.39059	0.252929	-5.498	3.84E-08	2.44E-06	STIM2
GALM	10.93416	-1.38394	0.445026	-3.1098	0.001872	0.031846	GALM
GGCX	18.32972	-1.38218	0.303154	-4.5593	5.13E-06	0.000205	GGCX
IER3	13.65302	-1.37859	0.365166	-3.7752	0.00016	0.004256	IER3
SMAP2	138.2516	-1.36285	0.11938	-11.416	3.48E-30	1.94E-27	SMAP2

RASSF2	17.23143	-1.34312	0.313727	-4.2812	1.86E-05	0.000628	RASSF2
IL6	12.23234	-1.33972	0.366658	-3.6539	0.000258	0.00623	IL6
USP8	46.31533	-1.33436	0.194674	-6.8544	7.16E-12	8.47E-10	USP8
CITED2	109.9248	-1.33094	0.150649	-8.8347	1.00E-18	2.57E-16	CITED2
GRASP	90.31639	-1.31742	0.142993	-9.2132	3.17E-20	1.02E-17	GRASP
HIST1H2BG	27.41811	-1.31673	0.254987	-5.1639	2.42E-07	1.32E-05	HIST1H2BG
PIGT	9.900146	-1.31396	0.426297	-3.0823	0.002054	0.034425	PIGT
FOXO3	9.854654	-1.30916	0.434314	-3.0143	0.002576	0.041394	FOXO3
TFEC	13.9689	-1.30484	0.354669	-3.6791	0.000234	0.005829	TFEC
KLF6	130.5945	-1.30098	0.160188	-8.1216	4.60E-16	8.09E-14	KLF6
MAP3K8	25.23976	-1.28445	0.271453	-4.7318	2.23E-06	0.000102	MAP3K8
FAM43A	16.27729	-1.28001	0.312256	-4.0992	4.15E-05	0.001301	FAM43A
SIK1	9.045198	-1.26173	0.428444	-2.9449	0.003231	0.048688	SIK1
TARSL2	13.59471	-1.25873	0.380947	-3.3042	0.000952	0.018536	TARSL2
ANKRD11	48.65026	-1.255	0.186511	-6.7288	1.71E-11	1.99E-09	ANKRD11
HSPA2	41.63414	-1.25478	0.204608	-6.1326	8.65E-10	7.71E-08	HSPA2
COL9A3	11.93731	-1.25382	0.384651	-3.2596	0.001116	0.020854	COL9A3
AC016831.7	21.35475	-1.24566	0.289297	-4.3058	1.66E-05	0.000575	AC016831.7
PTPN1	37.19166	-1.24554	0.218389	-5.7033	1.18E-08	8.60E-07	PTPN1
CREM	65.50358	-1.23335	0.204839	-6.0211	1.73E-09	1.46E-07	CREM
CFLAR	31.71222	-1.23286	0.236882	-5.2046	1.94E-07	1.08E-05	CFLAR
GKAP1	12.50319	-1.21511	0.375092	-3.2395	0.001197	0.022048	GKAP1
METTL4	10.61523	-1.21078	0.405995	-2.9823	0.002861	0.044549	METTL4
KLF9	13.22098	-1.20847	0.394871	-3.0604	0.00221	0.036442	KLF9
CCDC6	19.30169	-1.20193	0.2962	-4.0578	4.95E-05	0.001508	CCDC6
AC058791.1	25.14779	-1.19624	0.263251	-4.5441	5.52E-06	0.000218	AC058791.1
NFKBIZ	46.71815	-1.1698	0.19576	-5.9757	2.29E-09	1.90E-07	NFKBIZ
TXNIP	482.1391	-1.16687	0.083822	-13.921	4.74E-44	4.16E-41	TXNIP
AL021453.1	18.94502	-1.15066	0.354189	-3.2487	0.001159	0.021474	AL021453.1
AC025164.1	18.90775	-1.14616	0.315155	-3.6368	0.000276	0.00658	AC025164.1
SNAI1	13.01507	-1.14603	0.384035	-2.9842	0.002843	0.044462	SNAI1
CMTM7	14.47019	-1.14146	0.358737	-3.1819	0.001463	0.026009	CMTM7
POLR2A	39.82659	-1.12834	0.20674	-5.4578	4.82E-08	2.94E-06	POLR2A
HMGCS1	42.46379	-1.12133	0.217946	-5.145	2.68E-07	1.44E-05	HMGCS1
CRYBG1	20.28297	-1.11717	0.311893	-3.5819	0.000341	0.007887	CRYBG1
TOB1	39.53614	-1.11335	0.225623	-4.9346	8.03E-07	3.92E-05	TOB1
MT2A	2993.157	-1.11299	0.250767	-4.4384	9.06E-06	0.000342	MT2A
YWHAH	21.9117	-1.11268	0.298937	-3.7221	0.000198	0.005083	YWHAH
EHMT1	17.31268	-1.11214	0.316922	-3.5092	0.000449	0.010052	EHMT1
BACH1	31.17504	-1.11011	0.273558	-4.0581	4.95E-05	0.001508	BACH1
SIK3	15.99527	-1.1095	0.357026	-3.1076	0.001886	0.031867	SIK3
SPTAN1	47.84272	-1.10847	0.206218	-5.3753	7.65E-08	4.48E-06	SPTAN1
TUBB6	13.03764	-1.10587	0.371161	-2.9795	0.002887	0.044727	TUBB6
ATP2B1	83.48088	-1.09831	0.157407	-6.9775	3.00E-12	3.77E-10	ATP2B1
PARP8	14.64131	-1.0907	0.335898	-3.2471	0.001166	0.021532	PARP8
ACOT13	22.71003	-1.08974	0.280788	-3.881	0.000104	0.002948	ACOT13
C9orf72	16.14965	-1.07536	0.34593	-3.1086	0.00188	0.031846	C9orf72
C3orf58	18.99057	-1.06941	0.314397	-3.4015	0.00067	0.013649	C3orf58
AC087239.1	28.12875	-1.06502	0.251785	-4.2299	2.34E-05	0.000777	AC087239.1
UCP2	61.66984	-1.0644	0.170705	-6.2353	4.51E-10	4.20E-08	UCP2
TPM4	39.33121	-1.0615	0.3078	-3.4487	0.000563	0.011906	TPM4
TUBA1C	33.74711	-1.06004	0.240158	-4.4139	1.02E-05	0.000376	TUBA1C

SAMSN1	21.03536	-1.05441	0.313789	-3.3602	0.000779	0.015499	SAMSN1
MFN1	15.12761	-1.05435	0.35016	-3.0111	0.002603	0.041586	MFN1
HNRNPH1	143.9349	-1.05215	0.137805	-7.6351	2.26E-14	3.47E-12	HNRNPH1
CD70	20.13853	-1.04879	0.291214	-3.6015	0.000316	0.007379	CD70
SGK1	39.86718	-1.04441	0.240977	-4.3341	1.46E-05	0.000514	SGK1
ISG20	93.21678	-1.0418	0.145787	-7.146	8.93E-13	1.25E-10	ISG20
BTG1	470.7306	-1.03725	0.068805	-15.075	2.36E-51	3.63E-48	BTG1
CCNL2	17.81017	-1.02903	0.320203	-3.2137	0.00131	0.023496	CCNL2
TRIM22	52.01102	-1.02657	0.182777	-5.6165	1.95E-08	1.38E-06	TRIM22
EDEM1	16.2106	-1.0251	0.343575	-2.9836	0.002848	0.044462	EDEM1
CMTM6	54.4257	-1.02355	0.186172	-5.4979	3.84E-08	2.44E-06	CMTM6
CD44	58.65548	-1.0224	0.173236	-5.9018	3.60E-09	2.91E-07	CD44
SRSF7	443.8553	-1.0209	0.102904	-9.9209	3.38E-23	1.22E-20	SRSF7
IVNS1ABP	28.18538	-1.019	0.249348	-4.0867	4.38E-05	0.001359	IVNS1ABP
PSMB4	33.76389	-1.01598	0.254392	-3.9938	6.50E-05	0.001941	PSMB4
INTS6	150.1661	-1.01497	0.110531	-9.1827	4.20E-20	1.25E-17	INTS6
MXD1	44.8144	-1.01299	0.202027	-5.0141	5.33E-07	2.71E-05	MXD1
NEAT1	129.8394	-1.00343	0.180343	-5.564	2.64E-08	1.78E-06	NEAT1
TXN	51.57115	-0.9992	0.228615	-4.3707	1.24E-05	0.000448	TXN
NABP1	40.96118	-0.99905	0.218083	-4.581	4.63E-06	0.000187	NABP1
GNG2	16.68082	-0.99829	0.316761	-3.1515	0.001624	0.028295	GNG2
TAGLN2	99.48871	-0.98931	0.157429	-6.2842	3.30E-10	3.22E-08	TAGLN2
SMCHD1	129.6339	-0.98805	0.118538	-8.3353	7.73E-17	1.49E-14	SMCHD1
LINC01781	28.13048	-0.98227	0.257446	-3.8154	0.000136	0.003683	LINC01781
SLC20A1	23.3644	-0.97842	0.268111	-3.6493	0.000263	0.006317	SLC20A1
CIR1	31.5665	-0.97709	0.229161	-4.2638	2.01E-05	0.000676	CIR1
SLC39A10	32.17475	-0.97278	0.258574	-3.7621	0.000169	0.004467	SLC39A10
SLC5A3	26.80114	-0.97039	0.274196	-3.539	0.000402	0.009148	SLC5A3
MCL1	136.58	-0.96955	0.138538	-6.9984	2.59E-12	3.39E-10	MCL1
ZC3H12A	20.75079	-0.96925	0.283282	-3.4215	0.000623	0.012867	ZC3H12A
ZBTB20	80.37208	-0.94861	0.14953	-6.3439	2.24E-10	2.26E-08	ZBTB20
MARCKS	73.75443	-0.94656	0.164052	-5.7698	7.93E-09	6.02E-07	MARCKS
AREG	125.4048	-0.9426	0.13703	-6.8788	6.04E-12	7.28E-10	AREG
MGAT5	16.84535	-0.94073	0.317286	-2.9649	0.003028	0.046432	MGAT5
IL16	23.11416	-0.91858	0.273843	-3.3544	0.000795	0.01578	IL16
MAT2A	77.25536	-0.90338	0.164409	-5.4947	3.91E-08	2.46E-06	MAT2A
SAT1	280.0824	-0.8953	0.099348	-9.0118	2.03E-19	5.67E-17	SAT1
HEBP2	21.54565	-0.88656	0.277991	-3.1892	0.001427	0.025435	HEBP2
DENND4A	32.55881	-0.8818	0.233466	-3.777	0.000159	0.004244	DENND4A
OAT	34.09419	-0.87925	0.228584	-3.8465	0.00012	0.003334	OAT
RAB11FIP1	31.74668	-0.86997	0.232577	-3.7405	0.000184	0.004805	RAB11FIP1
CDC42SE2	50.73653	-0.8632	0.185758	-4.6469	3.37E-06	0.000145	CDC42SE2
PDE7A	30.51829	-0.86055	0.272451	-3.1585	0.001586	0.027783	PDE7A
LYN	53.24297	-0.8448	0.178772	-4.7255	2.29E-06	0.000103	LYN
REV3L	20.98627	-0.84475	0.285853	-2.9552	0.003125	0.047803	REV3L
ABHD5	53.8883	-0.84175	0.205254	-4.101	4.11E-05	0.001297	ABHD5
ELL2	42.57515	-0.84128	0.210362	-3.9992	6.36E-05	0.001907	ELL2
GPR183	56.58874	-0.82599	0.200829	-4.1129	3.91E-05	0.001239	GPR183
C6orf62	49.41866	-0.82069	0.188324	-4.3579	1.31E-05	0.000472	C6orf62
SYNGR2	46.86314	-0.81769	0.193872	-4.2177	2.47E-05	0.000812	SYNGR2
MBP	33.9155	-0.81186	0.232249	-3.4956	0.000473	0.010424	MBP
FUS	231.7889	-0.80728	0.114309	-7.0623	1.64E-12	2.24E-10	FUS

ID3	86.28014	-0.80411	0.173118	-4.6449	3.40E-06	0.000145	ID3
SETD2	23.84828	-0.80385	0.264925	-3.0343	0.002411	0.039232	SETD2
KMT2E	80.27363	-0.80051	0.149444	-5.3566	8.48E-08	4.83E-06	KMT2E
THUMPD3-AS1	96.12326	-0.79921	0.145571	-5.4902	4.02E-08	2.49E-06	THUMPD3-AS1
RPA3	35.02784	-0.79815	0.230387	-3.4644	0.000531	0.011469	RPA3
KDM6B	105.2762	-0.78631	0.138363	-5.6829	1.32E-08	9.47E-07	KDM6B
SNHG9	24.89824	-0.7788	0.263766	-2.9526	0.003151	0.048	SNHG9
TSPYL2	64.9691	-0.77084	0.178528	-4.3177	1.58E-05	0.000551	TSPYL2
FAM53C	48.15052	-0.76893	0.189176	-4.0646	4.81E-05	0.001479	FAM53C
CYTH1	29.51711	-0.7569	0.24131	-3.1366	0.001709	0.029691	CYTH1
SP140L	29.46476	-0.75492	0.242764	-3.1097	0.001873	0.031846	SP140L
HERPUD1	141.5436	-0.74975	0.134484	-5.575	2.48E-08	1.69E-06	HERPUD1
NXF1	27.72319	-0.7478	0.252161	-2.9655	0.003021	0.046432	NXF1
GOLGA4	42.38536	-0.74778	0.206046	-3.6292	0.000284	0.006751	GOLGA4
IQGAP1	41.58495	-0.74314	0.243143	-3.0564	0.00224	0.036793	IQGAP1
DNAJB4	51.54485	-0.73675	0.239308	-3.0787	0.002079	0.03475	DNAJB4
KIAA1551	72.55435	-0.73314	0.167356	-4.3807	1.18E-05	0.00043	KIAA1551
LY9	29.69965	-0.723	0.237489	-3.0443	0.002332	0.03814	LY9
HLA-C	459.3933	-0.72284	0.084474	-8.5569	1.16E-17	2.55E-15	HLA-C
NR3C1	29.41725	-0.72112	0.244264	-2.9522	0.003155	0.048	NR3C1
NKTR	61.82638	-0.7136	0.171665	-4.1569	3.23E-05	0.001039	NKTR
ELF1	57.32977	-0.71356	0.180571	-3.9517	7.76E-05	0.002241	ELF1
MYH9	32.42294	-0.70785	0.230858	-3.0662	0.002168	0.03598	MYH9
PRDM2	78.97541	-0.70714	0.162965	-4.3392	1.43E-05	0.000508	PRDM2
CAST	53.97651	-0.70699	0.191449	-3.6929	0.000222	0.005544	CAST
IFRD1	85.98459	-0.6867	0.144319	-4.7582	1.95E-06	9.03E-05	IFRD1
MAP3K2	36.68688	-0.66837	0.218349	-3.061	0.002206	0.036442	MAP3K2
ZFP36	303.7501	-0.65759	0.085836	-7.661	1.84E-14	2.91E-12	ZFP36
ARID5B	71.17156	-0.65136	0.169293	-3.8475	0.000119	0.003334	ARID5B
HSPH1	228.8092	-0.65128	0.129843	-5.0159	5.28E-07	2.71E-05	HSPH1
STK4	93.05119	-0.64773	0.145203	-4.4608	8.16E-06	0.00031	STK4
TPI1	64.47709	-0.64203	0.173147	-3.708	0.000209	0.005287	TPI1
GOLGB1	56.26525	-0.6409	0.188004	-3.409	0.000652	0.013368	GOLGB1
RSRP1	173.3463	-0.63601	0.111022	-5.7287	1.01E-08	7.50E-07	RSRP1
RHOB	71.06192	-0.62865	0.178829	-3.5154	0.000439	0.009889	RHOB
ACTG1	292.9681	-0.62467	0.095217	-6.5605	5.36E-11	6.00E-09	ACTG1
HCLS1	43.45089	-0.62305	0.208452	-2.9889	0.0028	0.044035	HCLS1
RPS10	743.2867	-0.61709	0.069942	-8.823	1.11E-18	2.74E-16	RPS10
SARAF	87.49871	-0.59916	0.156846	-3.8201	0.000133	0.003647	SARAF
DDIT4	119.4342	-0.5823	0.124146	-4.6905	2.73E-06	0.00012	DDIT4
LAPTM5	161.9497	-0.58168	0.115445	-5.0386	4.69E-07	2.47E-05	LAPTM5
SKIL	72.03368	-0.57435	0.183339	-3.1327	0.001732	0.030005	SKIL
PMAIP1	264.3506	-0.57152	0.120892	-4.7275	2.27E-06	0.000103	PMAIP1
H2AFX	87.33291	-0.56606	0.175627	-3.2231	0.001268	0.022938	H2AFX
AKAP9	61.58316	-0.56437	0.174286	-3.2382	0.001203	0.022085	AKAP9
SRSF6	56.94751	-0.55653	0.178156	-3.1238	0.001785	0.030754	SRSF6
FTH1	829.4182	-0.54601	0.077673	-7.0296	2.07E-12	2.77E-10	FTH1
ACTB	691.0135	-0.54478	0.099852	-5.4558	4.87E-08	2.94E-06	ACTB
MIDN	83.78869	-0.53864	0.155972	-3.4534	0.000554	0.011738	MIDN
TUBA1A	88.02721	-0.53045	0.167789	-3.1614	0.00157	0.027589	TUBA1A
ANKRD12	127.3649	-0.52869	0.150943	-3.5026	0.000461	0.01023	ANKRD12

BRD2	64.05372	-0.52334	0.168327	-3.109	0.001877	0.031846	BRD2
EIF4A2	199.0922	-0.5231	0.107264	-4.8767	1.08E-06	5.06E-05	EIF4A2
EGR1	154.2899	-0.52231	0.121904	-4.2846	1.83E-05	0.000622	EGR1
DUSP1	275.4262	-0.5184	0.104466	-4.9624	6.96E-07	3.45E-05	DUSP1
UBB	409.4152	-0.49264	0.080493	-6.1203	9.34E-10	8.09E-08	UBB
SP100	73.12679	-0.48423	0.16165	-2.9955	0.00274	0.043426	SP100
TUBA1B	117.6013	-0.47949	0.129214	-3.7108	0.000207	0.005265	TUBA1B
IER5	202.7893	-0.47651	0.107872	-4.4173	9.99E-06	0.000375	IER5
MYL6	171.9329	-0.47336	0.11271	-4.1998	2.67E-05	0.000874	MYL6
DNAJB1	672.6542	-0.4707	0.089372	-5.2667	1.39E-07	7.84E-06	DNAJB1
PTPRC	120.5099	-0.46112	0.125764	-3.6665	0.000246	0.006	PTPRC
BIRC3	169.3144	-0.45625	0.134077	-3.4029	0.000667	0.013624	BIRC3
HSPA8	224.453	-0.44857	0.098728	-4.5435	5.53E-06	0.000218	HSPA8
CDC42	139.3162	-0.44675	0.117878	-3.7899	0.000151	0.004047	CDC42
SRSF2	141.4256	-0.44486	0.121223	-3.6698	0.000243	0.005972	SRSF2
RBM39	163.797	-0.43157	0.119333	-3.6165	0.000299	0.007036	RBM39
EMP3	169.903	-0.42682	0.11186	-3.8157	0.000136	0.003683	EMP3
FOSB	306.7229	-0.41111	0.105633	-3.8919	9.95E-05	0.002846	FOSB
JUND	1000.439	-0.39914	0.059662	-6.6899	2.23E-11	2.54E-09	JUND
NAP1L1	144.8412	-0.39625	0.119758	-3.3088	0.000937	0.018296	NAP1L1
UBC	856.0323	-0.37833	0.084408	-4.4822	7.39E-06	0.000282	UBC
PTMA	1108.787	-0.31288	0.056586	-5.5294	3.21E-08	2.10E-06	PTMA
MALAT1	18168.01	-0.31221	0.053134	-5.8759	4.21E-09	3.36E-07	MALAT1
HMGB1	253.3681	-0.29247	0.096872	-3.0192	0.002535	0.040913	HMGB1
HLA-DQB1	314.8896	-0.28831	0.088587	-3.2545	0.001136	0.021171	HLA-DQB1
RPS21	643.4064	-0.28756	0.073918	-3.8902	0.0001	0.002852	RPS21
FOS	602.479	-0.27698	0.075456	-3.6708	0.000242	0.005972	FOS
CALM1	282.1318	-0.27489	0.091917	-2.9906	0.002784	0.043992	CALM1
CD74	1159.536	-0.26962	0.052128	-5.1723	2.31E-07	1.27E-05	CD74
B2M	2363.279	-0.22066	0.057681	-3.8256	0.00013	0.003582	B2M
RPL36	852.4227	-0.20676	0.060434	-3.4212	0.000623	0.012867	RPL36
TPT1	1939.668	0.185534	0.059918	3.09647	0.001958	0.032998	TPT1
RPL13	3059.346	0.195819	0.057038	3.43312	0.000597	0.012524	RPL13
RPS27A	2029.519	0.200959	0.062298	3.22575	0.001256	0.022794	RPS27A
RPS11	1089.975	0.201996	0.061827	3.26712	0.001086	0.020623	RPS11
RPS6	1366.99	0.226872	0.06508	3.48606	0.00049	0.010728	RPS6
RPS3A	1615.918	0.226979	0.074734	3.03715	0.002388	0.03896	RPS3A
RPL15	870.1501	0.234081	0.066957	3.49601	0.000472	0.010424	RPL15
RPL29	899.4083	0.238855	0.069786	3.4227	0.00062	0.012867	RPL29
RPL12	1055.392	0.248077	0.072378	3.4275	0.000609	0.012741	RPL12
RPSA	649.6791	0.262174	0.082423	3.18084	0.001468	0.026027	RPSA
RPL21	1637.312	0.273314	0.072811	3.75374	0.000174	0.004598	RPL21
RPL18	1147.668	0.275371	0.081264	3.38862	0.000702	0.014211	RPL18
RPL8	1116.479	0.303889	0.061174	4.96759	6.78E-07	3.39E-05	RPL8
CD37	412.634	0.311353	0.093963	3.31358	0.000921	0.018041	CD37
UBA52	640.1464	0.32118	0.075919	4.23055	2.33E-05	0.000777	UBA52
MT-ATP6	1893.673	0.321857	0.050058	6.42968	1.28E-10	1.31E-08	MT-ATP6
PNRC1	248.618	0.329193	0.095073	3.46251	0.000535	0.011478	PNRC1
MT-ND1	592.1749	0.350983	0.075303	4.66094	3.15E-06	0.000136	MT-ND1
MT-CYB	958.0375	0.37062	0.082469	4.49403	6.99E-06	0.000269	MT-CYB
CD79A	238.1835	0.376449	0.104724	3.59467	0.000325	0.007538	CD79A
RPS5	815.6139	0.388902	0.078791	4.93587	7.98E-07	3.92E-05	RPS5

RPL6	715.4905	0.407136	0.07053	5.77252	7.81E-09	6.02E-07	RPL6
HSPE1	564.0707	0.432196	0.089336	4.83786	1.31E-06	6.11E-05	HSPE1
CORO1A	113.3226	0.468612	0.140236	3.34158	0.000833	0.016473	CORO1A
RPL18A	1507.963	0.46987	0.063675	7.3792	1.59E-13	2.28E-11	RPL18A
CD52	424.9258	0.475705	0.084962	5.59903	2.16E-08	1.49E-06	CD52
MT-ND3	1173.528	0.488855	0.074648	6.54877	5.80E-11	6.37E-09	MT-ND3
TMSB4X	1147.461	0.502152	0.066056	7.60189	2.92E-14	4.38E-12	TMSB4X
CREBRF	76.62176	0.508466	0.172701	2.94419	0.003238	0.048688	CREBRF
FXYD5	80.64205	0.519881	0.166266	3.12681	0.001767	0.030528	FXYD5
LINC00926	83.2566	0.545567	0.167361	3.25982	0.001115	0.020854	LINC00926
NDUFA4	69.71147	0.550428	0.176647	3.11598	0.001833	0.031495	NDUFA4
TMSB10	767.2328	0.565759	0.067701	8.35677	6.45E-17	1.28E-14	TMSB10
HLA-DRB1	845.8976	0.579116	0.071581	8.09038	5.95E-16	9.89E-14	HLA-DRB1
RIPOR2	74.56918	0.582012	0.177031	3.28762	0.00101	0.019418	RIPOR2
RALGPS2	123.2819	0.588017	0.148804	3.95163	7.76E-05	0.002241	RALGPS2
AES	70.65447	0.605574	0.181776	3.33143	0.000864	0.016977	AES
SELENOK	115.6345	0.614838	0.154222	3.9867	6.70E-05	0.001981	SELENOK
POU2F2	72.39285	0.619027	0.180652	3.42662	0.000611	0.012741	POU2F2
MRPL18	153.5001	0.628775	0.139021	4.52288	6.10E-06	0.000239	MRPL18
SNX3	56.1713	0.663116	0.203939	3.25154	0.001148	0.021326	SNX3
IGKC	1373.785	0.669489	0.124924	5.35918	8.36E-08	4.83E-06	IGKC
ERP29	73.64465	0.676975	0.176898	3.82691	0.00013	0.003579	ERP29
POMP	52.43262	0.678302	0.20979	3.23323	0.001224	0.022337	POMP
UBE2D2	50.02962	0.687356	0.212738	3.231	0.001234	0.022445	UBE2D2
TNFAIP8	50.96996	0.716873	0.237933	3.01292	0.002587	0.04144	TNFAIP8
SELENOH	49.81117	0.744188	0.219203	3.39497	0.000686	0.013931	SELENOH
CFD	108.5015	0.746405	0.16216	4.6029	4.17E-06	0.000172	CFD
ZC3H15	44.42294	0.765275	0.232368	3.29337	0.00099	0.019085	ZC3H15
C12orf57	41.29996	0.778751	0.245658	3.17007	0.001524	0.026856	C12orf57
TRBC2	40.13085	0.836948	0.253626	3.29993	0.000967	0.018762	TRBC2
LTB	350.8321	0.856599	0.110839	7.72833	1.09E-14	1.76E-12	LTB
CD40	39.29935	0.874501	0.247373	3.53515	0.000408	0.009249	CD40
SNHG15	56.2022	0.883953	0.265215	3.33297	0.000859	0.016937	SNHG15
SEC62	78.28079	0.888114	0.176321	5.03691	4.73E-07	2.47E-05	SEC62
SLC38A1	32.83958	0.88944	0.271032	3.28168	0.001032	0.01977	SLC38A1
USP12	27.90209	0.921062	0.306471	3.00538	0.002652	0.042152	USP12
HVCN1	38.2474	0.924219	0.255146	3.62232	0.000292	0.006906	HVCN1
HSPA6	104.3517	0.959624	0.258651	3.71011	0.000207	0.005265	HSPA6
PSMB9	44.47219	0.961249	0.238571	4.0292	5.60E-05	0.001687	PSMB9
ITGB1	47.22549	0.968784	0.232382	4.16892	3.06E-05	0.000991	ITGB1
ATP5IF1	43.84253	0.974797	0.235042	4.14734	3.36E-05	0.001077	ATP5IF1
PPP1R2	34.57642	1.002205	0.269533	3.7183	0.000201	0.00514	PPP1R2
OSER1	55.59481	1.002265	0.218469	4.58768	4.48E-06	0.000184	OSER1
IKZF3	39.08769	1.024856	0.261903	3.91311	9.11E-05	0.002618	IKZF3
CDC40	28.6473	1.03312	0.297351	3.47441	0.000512	0.011129	CDC40
EIF4A3	147.9808	1.062917	0.153122	6.94163	3.88E-12	4.77E-10	EIF4A3
TMCO1	19.03778	1.066262	0.361416	2.95024	0.003175	0.048099	TMCO1
DCN	55.63388	1.066378	0.226437	4.70938	2.48E-06	0.00011	DCN
IGHM	306.8251	1.081053	0.096771	11.1712	5.64E-29	2.67E-26	IGHM
IGHD	71.24574	1.089471	0.21896	4.97566	6.50E-07	3.28E-05	IGHD
CAMK2D	18.8031	1.110631	0.374587	2.96495	0.003027	0.046432	CAMK2D
TRAPPC2B	24.58572	1.116357	0.321315	3.47434	0.000512	0.011129	TRAPPC2B

VPREB3	66.53169	1.122037	0.209424	5.35774	8.43E-08	4.83E-06	VPREB3
ISCA1	33.98206	1.132676	0.2838	3.99111	6.58E-05	0.001954	ISCA1
STX7	24.11114	1.14924	0.351215	3.27219	0.001067	0.020383	STX7
QRSL1	20.67446	1.152707	0.36515	3.1568	0.001595	0.027869	QRSL1
NCF1	45.47902	1.170788	0.248047	4.72003	2.36E-06	0.000105	NCF1
IGHG3	30.68229	1.174259	0.308892	3.80152	0.000144	0.003879	IGHG3
CSK	26.03338	1.178697	0.327308	3.60119	0.000317	0.007379	CSK
LYSMD2	16.05582	1.207924	0.400765	3.01404	0.002578	0.041394	LYSMD2
LSM2	17.87683	1.209545	0.381123	3.17364	0.001505	0.026604	LSM2
IGLC2	300.9823	1.218517	0.164086	7.42607	1.12E-13	1.64E-11	IGLC2
CKS2	86.88797	1.220779	0.187795	6.50059	8.00E-11	8.63E-09	CKS2
SPIB	38.4911	1.227525	0.285367	4.30157	1.70E-05	0.000583	SPIB
SCIMP	24.53905	1.245729	0.349503	3.56428	0.000365	0.008404	SCIMP
STRBP	20.32819	1.263714	0.367432	3.43932	0.000583	0.012283	STRBP
PLAC8	49.54768	1.290266	0.23839	5.41242	6.22E-08	3.71E-06	PLAC8
UPF3A	16.98911	1.294673	0.421507	3.07153	0.00213	0.035494	UPF3A
S1PR4	16.64906	1.336753	0.417126	3.20467	0.001352	0.024174	S1PR4
TUFM	15.7181	1.354072	0.420506	3.2201	0.001281	0.023062	TUFM
FCRL3	13.55191	1.3586	0.46131	2.94509	0.003229	0.048688	FCRL3
GAPT	22.66896	1.386771	0.371504	3.73286	0.000189	0.004913	GAPT
PTPN6	30.655	1.387575	0.307608	4.51085	6.46E-06	0.000251	PTPN6
IKZF1	20.20807	1.42816	0.371954	3.83961	0.000123	0.003414	IKZF1
GSN	23.18829	1.547901	0.374959	4.12819	3.66E-05	0.001165	GSN
BCAS2	121.4387	1.550905	0.190526	8.14012	3.95E-16	7.36E-14	BCAS2
PSMC6	12.7036	1.576441	0.507064	3.10896	0.001877	0.031846	PSMC6
LYL1	23.58659	1.58451	0.364564	4.34632	1.38E-05	0.000495	LYL1
CCNG2	10.18932	1.641352	0.551714	2.97501	0.00293	0.045272	CCNG2
PLEKHF2	19.81347	1.722722	0.421206	4.08998	4.31E-05	0.001347	PLEKHF2
IGHG2	22.78211	1.73907	0.380068	4.57568	4.75E-06	0.000191	IGHG2
BLNK	12.04874	1.74551	0.529894	3.29407	0.000987	0.019085	BLNK
CSTB	22.63548	1.798641	0.389991	4.61201	3.99E-06	0.000168	CSTB
LUM	21.41796	1.837185	0.40067	4.58528	4.53E-06	0.000185	LUM
CALHM6	8.493966	1.919244	0.634007	3.02717	0.002469	0.04001	CALHM6
HES1	15.38049	1.97436	0.533113	3.70346	0.000213	0.005355	HES1
PHACTR1	41.25028	1.999431	0.309099	6.46858	9.89E-11	1.05E-08	PHACTR1
PLEK	23.93044	2.028895	0.404512	5.01566	5.29E-07	2.71E-05	PLEK
ARL5B	17.32998	2.069176	0.46948	4.40738	1.05E-05	0.000384	ARL5B
EAF2	30.30737	2.080066	0.35486	5.86165	4.58E-09	3.61E-07	EAF2
TCL1A	17.62057	2.087002	0.481139	4.33763	1.44E-05	0.000509	TCL1A
FAM96A	11.15661	2.106803	0.584584	3.60394	0.000313	0.007357	FAM96A
CD79B	105.6157	2.27401	0.213708	10.6407	1.93E-26	7.90E-24	CD79B
FBN1	7.553748	2.347465	0.76568	3.06585	0.00217	0.03598	FBN1
FBLN1	9.662059	2.413418	0.684106	3.52784	0.000419	0.009473	FBLN1
TLR10	15.64254	2.416606	0.537482	4.49616	6.92E-06	0.000268	TLR10
C3	12.95144	2.545455	0.630512	4.03712	5.41E-05	0.001639	C3
TNXB	6.657143	2.605288	0.871333	2.99	0.00279	0.043992	TNXB
GLA	9.867487	2.773806	0.749137	3.70267	0.000213	0.005355	GLA
CD180	8.602267	3.12639	0.878295	3.55961	0.000371	0.008523	CD180
HP	4.600858	3.459855	1.074531	3.21987	0.001282	0.023062	HP
DBP	5.001466	3.616331	1.060404	3.41033	0.000649	0.013346	DBP
IGFBP5	8.630642	3.662068	1.00208	3.65447	0.000258	0.00623	IGFBP5
FAM177B	5.566409	3.822515	1.075447	3.55435	0.000379	0.008663	FAM177B

Supplementary Table S3d: Differentially expressed genes in VAT CD8^{+} T cells between patients with or without CAC by pseudo-bulk analysis.

	baseMean	log2FC	lfcSE	stat	pvalue	padj
CH25H	46.13455835	-8.48141	1.03502	-8.19447	2.52E-16	1.20E-14
HLA-DQA2	33.16571696	-5.262441	0.5587	-9.41909	4.55E-21	2.97E-19
IL10	6.309451375	-3.865926	0.84908	-4.55307	5.29E-06	7.98E-05
FP565260.1	4.406726324	-3.702203	0.98288	-3.7667	0.00016542	0.001797967
ABTB2	4.46914999	-3.343736	0.89626	-3.73076	0.000190905	0.002028485
CRYBG1	330.6692643	-3.224995	0.10564	-30.5289	1.08E-204	1.28E-201
DDIT4-AS1	8.135820682	-3.151364	0.62819	-5.01657	5.26E-07	9.30E-06
IL7R	1296.144155	-3.031597	0.13019	-23.2852	6.26E-120	3.52E-117
LINC00239	19.7997179	-3.02769	0.40034	-7.56277	3.95E-14	1.66E-12
PARD6G	36.14733554	-2.991862	0.29127	-10.2718	9.44E-25	7.06E-23
AC002306.1	4.358416617	-2.951505	0.8333	-3.54194	0.000397198	0.003790874
MT1A	33.41789596	-2.93427	0.30923	-9.48893	2.33E-21	1.56E-19
ANK3	27.07699924	-2.91463	0.33341	-8.74199	2.29E-18	1.26E-16
S100A8	19.24344779	-2.872171	0.39449	-7.28071	3.32E-13	1.26E-11
AC132872.1	10.27700898	-2.745313	0.51858	-5.29388	1.20E-07	2.33E-06
FKBP5	226.3314142	-2.666089	0.11699	-22.7884	5.98E-115	3.05E-112
S100A9	32.02912701	-2.621706	0.30442	-8.61216	7.17E-18	3.72E-16
NLRP3	18.47609082	-2.575636	0.38907	-6.61996	3.59E-11	1.11E-09
ANKRD28	205.8632391	-2.551892	0.11993	-21.2787	1.79E-100	7.65E-98
TPPP	15.91809676	-2.521613	0.4178	-6.0355	1.58E-09	3.93E-08
TSC22D3	1898.049931	-2.509603	0.05436	-46.1661	0	0
MAML3	6.18355151	-2.480126	0.63774	-3.88892	0.00010069	0.001160971
SLC7A5	128.1098048	-2.475086	0.15121	-16.3686	3.20E-60	7.00E-58
AC007952.4	326.047063	-2.475032	0.10183	-24.3053	1.72E-130	1.02E-127
PFKFB3	111.0534887	-2.448664	0.15451	-15.8482	1.45E-56	2.98E-54
EFCAB2	6.273574307	-2.38489	0.65804	-3.62421	0.000289844	0.002898443
MCAM	6.391750895	-2.378161	0.65033	-3.65683	0.000255351	0.002589819
HLA-DRB5	11.66510951	-2.360693	0.46657	-5.05963	4.20E-07	7.59E-06
ELL2	254.09423	-2.347075	0.10267	-22.8602	1.16E-115	6.19E-113
CXCL8	13.88062392	-2.327834	0.42338	-5.49821	3.84E-08	8.03E-07
AC074099.1	6.15489754	-2.318856	0.62868	-3.68845	0.000225624	0.002321326
PBX4	73.19982452	-2.317098	0.1869	-12.3973	2.70E-35	3.11E-33
ZFP36L2	2521.353218	-2.306585	0.04215	-54.7237	0	0
SLC19A2	19.10540092	-2.281785	0.35358	-6.45333	1.09E-10	3.17E-09
CXCR4	2218.92502	-2.237316	0.05289	-42.3039	0	0
SC5D	91.57967701	-2.175806	0.17341	-12.5469	4.13E-36	4.91E-34
AL662844.4	17.54026269	-2.157639	0.38309	-5.63224	1.78E-08	3.95E-07
SNX9	54.40257939	-2.148454	0.20904	-10.2778	8.87E-25	6.68E-23
FAM229B	4.933068	-2.130477	0.71446	-2.98193	0.002864373	0.020542086
ICOSLG	4.842845399	-2.056618	0.68718	-2.99283	0.002764002	0.019956023
AC106739.2	8.136986117	-2.05549	0.52334	-3.92761	8.58E-05	0.001009898
ZBED4	30.05598508	-2.030578	0.27371	-7.41859	1.18E-13	4.69E-12
MILR1	9.054578997	-2.028767	0.50581	-4.01092	6.05E-05	0.000736247
SMAP2	316.0507471	-2.013375	0.09199	-21.886	3.53E-106	1.64E-103
GCNA	18.71414637	-2.012705	0.3484	-5.77692	7.61E-09	1.75E-07
FAM129A	206.1161933	-2.009163	0.11139	-18.0371	9.97E-73	2.67E-70
RASSF8	11.55747716	-1.991484	0.44727	-4.45254	8.49E-06	0.000123705
AC016831.1	98.60300722	-1.989714	0.15921	-12.4978	7.68E-36	9.02E-34

AC020916.1	149.6624572	-1.98894	0.13257	-15.0034	6.98E-51	1.33E-48
PIK3IP1	301.2172025	-1.984986	0.09653	-20.5633	5.85E-94	2.24E-91
HPGD	69.946227	-1.978059	0.1886	-10.4881	9.80E-26	7.78E-24
LMNA	745.8478683	-1.977786	0.07454	-26.5346	3.87E-155	3.19E-152
PRDM1	210.4915421	-1.975828	0.11838	-16.69	1.55E-62	3.45E-60
KANTR	12.54360691	-1.951461	0.42187	-4.62571	3.73E-06	5.85E-05
FAM118A	88.68985927	-1.938253	0.15867	-12.2158	2.56E-34	2.80E-32
KLF9	132.0255617	-1.935728	0.13853	-13.9738	2.25E-44	3.49E-42
TNFAIP3	1368.900717	-1.935527	0.06083	-31.8168	3.79E-222	5.80E-219
SKI	112.1015155	-1.92508	0.14759	-13.0433	6.94E-39	9.05E-37
AL360012.1	47.19977197	-1.916621	0.22369	-8.56801	1.05E-17	5.39E-16
FAM222A	6.276510381	-1.910838	0.59544	-3.20914	0.001331321	0.010630696
TRABD2A	9.397782146	-1.907045	0.48022	-3.97117	7.15E-05	0.000852182
AC058791.1	246.2323127	-1.860157	0.10069	-18.4742	3.33E-76	9.63E-74
FRMD4B	33.07418768	-1.856033	0.25984	-7.14304	9.13E-13	3.23E-11
MIR29B2CHG	8.20143253	-1.84527	0.51035	-3.61567	0.000299567	0.002985723
LINC00513	52.64587945	-1.842495	0.20994	-8.77628	1.69E-18	9.52E-17
AL357060.1	33.69165552	-1.822307	0.25233	-7.222	5.12E-13	1.88E-11
LINC01970	11.95337662	-1.803946	0.42381	-4.25646	2.08E-05	0.00027779
SPINK2	29.58860394	-1.791902	0.28914	-6.19728	5.74E-10	1.53E-08
IFNGR1	172.3387713	-1.789538	0.12323	-14.5224	8.74E-48	1.48E-45
PGAP1	12.97319022	-1.779241	0.40139	-4.43275	9.30E-06	0.000134751
GABPB1	49.9161019	-1.774033	0.21492	-8.25458	1.52E-16	7.39E-15
CNOT6L	534.3375793	-1.772799	0.06997	-25.3365	1.27E-141	8.48E-139
FSCN1	9.931482298	-1.76348	0.46147	-3.82142	0.000132685	0.001475807
GPAT3	6.632086123	-1.739797	0.55815	-3.11706	0.001826616	0.01402065
LSR	30.35105881	-1.731892	0.26842	-6.45215	1.10E-10	3.19E-09
ERMN	5.857072876	-1.727255	0.59241	-2.91566	0.003549322	0.024549289
ZDBF2	19.66590456	-1.723861	0.34233	-5.03573	4.76E-07	8.48E-06
USP40	5.004138633	-1.722516	0.65545	-2.62799	0.008589013	0.049277447
PIM2	85.80727082	-1.718471	0.15788	-10.8848	1.36E-27	1.21E-25
PELI1	31.99306455	-1.716612	0.267	-6.42922	1.28E-10	3.70E-09
AIM2	8.130210908	-1.714487	0.51086	-3.35609	0.000790522	0.006832461
ZNF250	25.48581612	-1.713781	0.28503	-6.01271	1.82E-09	4.48E-08
AC016831.5	136.1305413	-1.708562	0.12622	-13.5361	9.57E-42	1.35E-39
RBAK-RBAKI	11.00969411	-1.691458	0.44449	-3.80535	0.000141602	0.001557187
TCAF2	9.204081485	-1.66624	0.48902	-3.40731	0.000656076	0.005792092
ZBTB16	16.38346433	-1.652358	0.35733	-4.62421	3.76E-06	5.88E-05
IPCEF1	99.17510452	-1.649115	0.14829	-11.1206	9.96E-29	9.11E-27
GOLGA6L4	6.010183486	-1.647291	0.5771	-2.85441	0.004311711	0.028780606
SYTL3	271.7267733	-1.6449	0.09514	-17.2891	5.69E-67	1.45E-64
DOCK5	22.99836698	-1.625397	0.30223	-5.37803	7.53E-08	1.50E-06
NFKBIA	1736.245703	-1.614623	0.04897	-32.9714	2.09E-238	3.72E-235
PDZD4	9.462719199	-1.608457	0.47161	-3.41058	0.000648248	0.005737186
PLEKHG2	33.52669863	-1.6056	0.25036	-6.41317	1.43E-10	4.09E-09
BRD1	103.2586435	-1.60542	0.15185	-10.5721	4.02E-26	3.31E-24
SPDYA	7.594186098	-1.595161	0.51577	-3.09277	0.001982969	0.015058746
RGPD5	37.75998993	-1.59115	0.23935	-6.64787	2.97E-11	9.25E-10
AP006284.1	7.630013157	-1.590778	0.51296	-3.1012	0.001927406	0.014719839
AGPAT4	16.78218617	-1.584257	0.35168	-4.50484	6.64E-06	9.83E-05
G0S2	9.764716624	-1.562806	0.45706	-3.41925	0.00062794	0.005588458
AC245014.3	23.80976442	-1.560007	0.29745	-5.24467	1.57E-07	2.99E-06

PPP1CB	431.7644029	-1.557112	0.08168	-19.0628	5.14E-81	1.67E-78
AREG	420.8872985	-1.555434	0.17975	-8.65337	5.00E-18	2.64E-16
FAM43A	33.34258465	-1.55323	0.25836	-6.01196	1.83E-09	4.49E-08
SLC5A6	11.81614421	-1.552123	0.4306	-3.60456	0.000312677	0.003083542
TMEM88	10.25202481	-1.550627	0.43964	-3.52701	0.000420282	0.00398672
C1orf56	110.0361543	-1.546912	0.15368	-10.066	7.81E-24	5.64E-22
CLECL1	26.90687056	-1.545079	0.27932	-5.53151	3.17E-08	6.78E-07
GFPT2	16.30516097	-1.543965	0.35225	-4.38316	1.17E-05	0.000166214
CCNH	310.6713062	-1.531892	0.08873	-17.2642	8.76E-67	2.18E-64
LRIG1	18.36003069	-1.530294	0.33786	-4.52932	5.92E-06	8.88E-05
KMT5C	16.86155501	-1.509574	0.35911	-4.20363	2.63E-05	0.000346131
SND1-IT1	7.243738161	-1.508421	0.53207	-2.83499	0.004582667	0.030218104
Z93241.1	67.3183878	-1.503138	0.17364	-8.65651	4.86E-18	2.58E-16
SMIM3	34.07961545	-1.498759	0.25437	-5.89196	3.82E-09	9.03E-08
POLR1E	49.06429595	-1.498447	0.2154	-6.95646	3.49E-12	1.17E-10
LINC02446	42.24062959	-1.497314	0.2208	-6.78128	1.19E-11	3.82E-10
PER1	163.2513375	-1.495947	0.11741	-12.7415	3.48E-37	4.33E-35
AC016831.7	128.362222	-1.492123	0.14107	-10.5773	3.80E-26	3.17E-24
AP001157.1	14.39471394	-1.489224	0.39932	-3.72937	0.000191958	0.002035633
KLF6	1746.345053	-1.478708	0.05502	-26.8764	4.15E-159	3.70E-156
RAB9A	83.86638282	-1.472283	0.1618	-9.09968	9.06E-20	5.48E-18
KLF5	11.81902742	-1.471406	0.41815	-3.51888	0.000433376	0.004085573
MT1E	16219.35535	-1.469436	0.0866	-16.9683	1.41E-64	3.35E-62
ZFP36	3338.592953	-1.469026	0.04376	-33.5664	5.18E-247	1.11E-243
GRAMD1B	25.15039184	-1.464445	0.28205	-5.19214	2.08E-07	3.92E-06
MCL1	1237.882513	-1.463689	0.05569	-26.284	2.92E-152	2.24E-149
NFKBIZ	219.810045	-1.461118	0.10984	-13.3023	2.24E-40	3.08E-38
FAM177A1	493.623806	-1.459334	0.06995	-20.8629	1.16E-96	4.79E-94
PHKG1	24.65595668	-1.453348	0.28669	-5.06932	3.99E-07	7.25E-06
BACH2	16.60289205	-1.452511	0.34439	-4.2176	2.47E-05	0.000326974
FOXO1	112.7174096	-1.443692	0.13825	-10.4426	1.58E-25	1.24E-23
SATB1	86.77955428	-1.438968	0.15597	-9.22567	2.82E-20	1.72E-18
PELI2	6.953695033	-1.434987	0.54346	-2.64048	0.008278798	0.047908673
IL18R1	46.52127673	-1.430392	0.21194	-6.74915	1.49E-11	4.74E-10
ISG20	444.2963716	-1.416672	0.07327	-19.3348	2.74E-83	9.16E-81
CRTAM	168.0705329	-1.411404	0.18809	-7.50381	6.20E-14	2.53E-12
AC005332.8	8.584403739	-1.4068	0.49346	-2.85089	0.004359687	0.029046482
ZSWIM6	24.27049612	-1.401051	0.30228	-4.635	3.57E-06	5.62E-05
MT1X	24416.14349	-1.399461	0.04014	-34.8614	2.86E-266	7.66E-263
IL18RAP	45.99869384	-1.397044	0.21231	-6.58034	4.69E-11	1.43E-09
TXNIP	1942.9916	-1.396673	0.04981	-28.0381	5.58E-173	5.43E-170
PHLDA1	271.9233043	-1.396314	0.0947	-14.7446	3.33E-49	5.85E-47
NPIPB4	12.36230925	-1.39569	0.40477	-3.44813	0.00056448	0.005092692
CEMIP2	320.5954872	-1.393036	0.08752	-15.9166	4.86E-57	1.02E-54
SEC14L1	58.70056392	-1.390967	0.18683	-7.44495	9.70E-14	3.92E-12
KIF13B	55.10783103	-1.390523	0.19079	-7.28813	3.14E-13	1.20E-11
OBSCN	9.305246848	-1.390131	0.46235	-3.00665	0.002641409	0.019174408
CEBPB	303.9926081	-1.384743	0.15656	-8.84473	9.18E-19	5.22E-17
SYAP1	200.3625007	-1.382936	0.10859	-12.735	3.78E-37	4.65E-35
IRF4	27.71358376	-1.382734	0.26659	-5.18674	2.14E-07	4.02E-06
IL23A	19.45294537	-1.380992	0.32029	-4.31175	1.62E-05	0.000221622
CEBPD	136.9257986	-1.377879	0.12324	-11.1804	5.09E-29	4.69E-27

CENPM	23.36613975	-1.377061	0.31106	-4.42693	9.56E-06	0.000137652
THEM4	40.56032313	-1.368814	0.22357	-6.12242	9.22E-10	2.38E-08
SERPINB9	134.3848888	-1.367192	0.20885	-6.54621	5.90E-11	1.75E-09
LRRC8C	41.46879738	-1.359549	0.22107	-6.14993	7.75E-10	2.02E-08
PDE3B	66.03477266	-1.359183	0.17606	-7.71989	1.16E-14	5.15E-13
SYNJ2	23.58104444	-1.358949	0.2965	-4.58325	4.58E-06	7.01E-05
PTCH2	23.56031072	-1.352297	0.29609	-4.56716	4.94E-06	7.51E-05
NSG1	20.54736867	-1.352126	0.31472	-4.29631	1.74E-05	0.000235515
RAPGEF2	23.96416739	-1.34597	0.29253	-4.60107	4.20E-06	6.51E-05
RNF19A	238.510287	-1.330188	0.09426	-14.1114	3.23E-45	5.16E-43
TOB1	284.4046791	-1.326942	0.08656	-15.3296	4.85E-53	9.79E-51
ETS1	472.6170424	-1.325695	0.07204	-18.402	1.27E-75	3.56E-73
CASS4	17.47692256	-1.317023	0.3361	-3.91855	8.91E-05	0.001041747
ARHGAP5	43.67307439	-1.312758	0.21224	-6.18514	6.20E-10	1.64E-08
ATP2B1	226.4494886	-1.304006	0.09923	-13.1411	1.91E-39	2.56E-37
GGCX	52.3048542	-1.295864	0.19696	-6.57946	4.72E-11	1.44E-09
ZMAT1	29.85792223	-1.287972	0.2634	-4.88974	1.01E-06	1.72E-05
AC114760.2	14.94774062	-1.287905	0.37095	-3.47193	0.000516725	0.00472965
MPP7	26.48137828	-1.283967	0.2961	-4.3363	1.45E-05	0.000199795
SOCS1	270.3591404	-1.280352	0.09364	-13.6729	1.47E-42	2.13E-40
ZNF831	80.35865606	-1.275434	0.15806	-8.06923	7.07E-16	3.29E-14
U2AF1	9.996561183	-1.273415	0.44171	-2.88295	0.003939691	0.026714002
CD55	203.7763598	-1.271683	0.10349	-12.2876	1.06E-34	1.18E-32
NPC1	31.24036667	-1.270196	0.25102	-5.06009	4.19E-07	7.59E-06
TSPYL2	501.3058277	-1.263798	0.07338	-17.2223	1.81E-66	4.39E-64
ZC3H12D	11.06474144	-1.262657	0.42693	-2.95755	0.003100904	0.022031658
IL2RA	15.35816488	-1.262558	0.3725	-3.38945	0.000700322	0.00613714
AUTS2	79.77676477	-1.26138	0.16679	-7.56285	3.94E-14	1.66E-12
RASGRF2	17.50998019	-1.258527	0.33476	-3.75944	0.000170295	0.001846156
KDM6B	378.3090398	-1.254661	0.08187	-15.3246	5.24E-53	1.04E-50
MCM6	35.11326016	-1.25171	0.24957	-5.01545	5.29E-07	9.34E-06
PHLDA2	79.12306347	-1.25047	0.16109	-7.76274	8.31E-15	3.71E-13
GPM6B	39.89147304	-1.247793	0.23766	-5.25031	1.52E-07	2.91E-06
SERINC5	31.23297328	-1.246899	0.25461	-4.89726	9.72E-07	1.66E-05
GPR171	147.2051447	-1.241779	0.12589	-9.86433	5.94E-23	4.18E-21
ATP1B3	507.0807058	-1.239692	0.07163	-17.3071	4.16E-67	1.09E-64
CHD7	16.19151647	-1.239534	0.35447	-3.49684	0.000470804	0.004380525
ZNF165	10.82073775	-1.236729	0.44322	-2.79036	0.005265025	0.033573163
TP53INP2	40.0983507	-1.236297	0.22183	-5.57315	2.50E-08	5.38E-07
ABCC1	21.27382942	-1.232649	0.30673	-4.0187	5.85E-05	0.000715606
RNF115	126.5726113	-1.232005	0.13117	-9.39251	5.86E-21	3.78E-19
BCL2L11	49.71589932	-1.227609	0.19936	-6.15777	7.38E-10	1.93E-08
TGIF1	66.75630614	-1.221185	0.17217	-7.09275	1.31E-12	4.60E-11
ZNF274	22.1479016	-1.214174	0.31001	-3.91658	8.98E-05	0.001049117
MAFF	188.6512231	-1.21346	0.11311	-10.7286	7.47E-27	6.45E-25
EZR	899.4037346	-1.211992	0.06616	-18.3199	5.74E-75	1.58E-72
AC116366.1	24.42132984	-1.208787	0.28409	-4.25493	2.09E-05	0.000279343
AGO2	105.5161497	-1.205005	0.14191	-8.49115	2.05E-17	1.02E-15
AC025259.3	11.22833102	-1.203248	0.41121	-2.92615	0.003431864	0.023891314
WHRN	61.2816294	-1.197243	0.19396	-6.17278	6.71E-10	1.76E-08
CHML	17.86183179	-1.197093	0.33829	-3.53864	0.0004022	0.003828774
MAML2	43.63486761	-1.196981	0.20981	-5.7052	1.16E-08	2.63E-07

GOLM1	10.65127534	-1.195464	0.41994	-2.84674	0.004416909	0.029318193
NSUN4	10.79949042	-1.193654	0.42351	-2.81845	0.004825633	0.031409824
GSPT1	220.1339745	-1.192675	0.09947	-11.9909	3.97E-33	4.24E-31
AL121944.1	57.54579194	-1.18755	0.20324	-5.84303	5.13E-09	1.20E-07
LPIN1	85.44129042	-1.185988	0.15135	-7.83591	4.65E-15	2.08E-13
SRGN	3536.809482	-1.184895	0.03866	-30.6518	2.50E-206	3.35E-203
ZNF100	15.08647407	-1.18306	0.36797	-3.2151	0.001304005	0.010443757
PIGA	18.6164799	-1.179774	0.3322	-3.55142	0.000383156	0.00367034
ANKRD9	19.15022282	-1.179313	0.31685	-3.72201	0.000197641	0.002081455
C9orf72	19.80252133	-1.175476	0.3229	-3.64032	0.000272298	0.002743494
SETD7	14.65165358	-1.17145	0.36435	-3.21518	0.001303615	0.010443757
BRD9	100.1316614	-1.163985	0.14536	-8.00765	1.17E-15	5.37E-14
AC009404.1	12.57191416	-1.162381	0.38591	-3.01202	0.002595195	0.018915933
RNF125	220.5723163	-1.162261	0.10216	-11.3773	5.43E-30	5.14E-28
CBLB	199.2410905	-1.156849	0.10546	-10.9699	5.33E-28	4.80E-26
NFKB1	140.1059028	-1.155748	0.13195	-8.75929	1.96E-18	1.10E-16
TNFSF8	40.39406446	-1.155496	0.22625	-5.10726	3.27E-07	5.96E-06
GPCPD1	101.098852	-1.15325	0.14247	-8.09471	5.74E-16	2.71E-14
ITGAV	20.02980744	-1.152461	0.30727	-3.7507	0.000176344	0.001896358
RNF103	38.06125431	-1.151794	0.22283	-5.16898	2.35E-07	4.40E-06
POC1B-AS1	11.430841	-1.149804	0.41268	-2.78617	0.005333449	0.033908442
ERN1	134.1736602	-1.148861	0.13041	-8.80938	1.26E-18	7.12E-17
SGK1	73.44554097	-1.147973	0.16553	-6.93502	4.06E-12	1.36E-10
AEN	15.9836347	-1.147941	0.34749	-3.30349	0.000954889	0.008008568
CSRNP2	23.05457731	-1.146386	0.30104	-3.80815	0.000140013	0.00154766
SARAF	903.4418038	-1.14457	0.0552	-20.7338	1.72E-95	6.81E-93
MUC20-OT1	57.80125326	-1.143638	0.18438	-6.20256	5.56E-10	1.48E-08
AL162377.1	24.51012896	-1.139427	0.2818	-4.04337	5.27E-05	0.000648
PDZD8	49.88658922	-1.135686	0.20202	-5.62162	1.89E-08	4.18E-07
HNRNPH1	706.3702033	-1.135399	0.06033	-18.8201	5.17E-79	1.58E-76
HERPUD2	155.8913812	-1.131212	0.11633	-9.72429	2.38E-22	1.65E-20
BTG1	4653.518329	-1.128704	0.03994	-28.2632	9.80E-176	1.05E-172
RNF19B	15.77847334	-1.128126	0.3623	-3.1138	0.001846955	0.014166609
CBWD1	28.13567659	-1.123638	0.26335	-4.26671	1.98E-05	0.000266667
LPAR2	29.42718298	-1.122866	0.26493	-4.23832	2.25E-05	0.000299706
LENG8	74.56380139	-1.116704	0.16549	-6.74788	1.50E-11	4.76E-10
RUNX2	29.52859888	-1.116568	0.25652	-4.35283	1.34E-05	0.000186023
IRS2	46.55539167	-1.115709	0.20648	-5.40357	6.53E-08	1.32E-06
PARP8	348.6199871	-1.112417	0.08222	-13.5304	1.03E-41	1.44E-39
CYB5D1	20.52191931	-1.105574	0.30414	-3.63512	0.000277857	0.00279161
GPR183	185.7716242	-1.103854	0.10632	-10.3821	2.99E-25	2.29E-23
ANXA1	1706.053627	-1.100118	0.04437	-24.7939	1.04E-135	6.57E-133
DHCR7	13.50132677	-1.099333	0.37815	-2.90712	0.003647729	0.025084
NRIP3	13.19217721	-1.098328	0.39335	-2.79223	0.005234614	0.033439025
NELL2	11.92773817	-1.09785	0.39594	-2.77279	0.005557841	0.035126341
PHF20	194.557066	-1.097035	0.10519	-10.4295	1.82E-25	1.41E-23
FOXP1	233.8471087	-1.095771	0.10362	-10.575	3.89E-26	3.23E-24
APBA2	33.76067877	-1.0915	0.25068	-4.35418	1.34E-05	0.000185123
PERP	87.47895999	-1.090951	0.15255	-7.15135	8.59E-13	3.06E-11
GNAO1	18.78253635	-1.090348	0.31561	-3.4547	0.000550905	0.004991267
SKIL	290.4773686	-1.08728	0.08751	-12.424	1.94E-35	2.25E-33
ADGRE5	356.4954254	-1.085098	0.08239	-13.1697	1.31E-39	1.78E-37

MRPL1	103.8936067	-1.077561	0.14746	-7.30756	2.72E-13	1.05E-11
B4GALT1	133.4462946	-1.077173	0.12839	-8.3899	4.87E-17	2.39E-15
IER3	112.3924196	-1.076749	0.14136	-7.6171	2.59E-14	1.11E-12
POLR3E	36.07959748	-1.076548	0.2307	-4.66639	3.07E-06	4.87E-05
IL4R	40.56313638	-1.074765	0.22145	-4.85323	1.21E-06	2.04E-05
BTN3A2	89.70390459	-1.07085	0.14667	-7.30121	2.85E-13	1.10E-11
SDC4	13.56999415	-1.069501	0.40595	-2.63456	0.008424549	0.048620647
SMCHD1	361.3431985	-1.067157	0.08161	-13.076	4.52E-39	5.97E-37
ARID5B	219.112746	-1.066252	0.10166	-10.4888	9.72E-26	7.78E-24
FYN	402.3228938	-1.063201	0.0772	-13.772	3.76E-43	5.58E-41
PDE4D	175.3197012	-1.063051	0.11497	-9.24641	2.32E-20	1.43E-18
CDKN1A	148.6141271	-1.060283	0.11537	-9.18998	3.93E-20	2.39E-18
DUSP16	34.69356382	-1.058419	0.24228	-4.36856	1.25E-05	0.000175852
HDAC4	19.56209285	-1.056719	0.31497	-3.35497	0.000793731	0.006847659
SPAG1	13.03477097	-1.056014	0.38021	-2.77742	0.005479169	0.034670082
NBL1	31.22977708	-1.054955	0.25361	-4.15968	3.19E-05	0.000409367
NR3C1	240.3096625	-1.05493	0.09798	-10.7664	4.96E-27	4.32E-25
MALT1	75.47755302	-1.04994	0.16835	-6.23672	4.47E-10	1.20E-08
MAP3K8	114.413966	-1.047421	0.13889	-7.54124	4.66E-14	1.95E-12
MAF	161.6109961	-1.044018	0.1124	-9.28857	1.56E-20	9.73E-19
LSMEM1	14.39505388	-1.038456	0.36879	-2.81581	0.004865417	0.031628169
DDIT4	1118.844823	-1.03772	0.05485	-18.9179	8.12E-80	2.55E-77
AL121603.2	24.97379347	-1.033262	0.28599	-3.61294	0.00030275	0.003002249
PPP2R5C	411.2100441	-1.033096	0.08139	-12.6933	6.44E-37	7.83E-35
FTH1	4662.270425	-1.032813	0.04038	-25.5764	2.79E-144	1.99E-141
ODC1	171.8051287	-1.029736	0.11133	-9.24956	2.25E-20	1.39E-18
SEC24B	24.43134591	-1.027887	0.29093	-3.53309	0.00041074	0.003903125
USP36	105.116882	-1.026525	0.14025	-7.31932	2.49E-13	9.70E-12
AC108673.3	13.02332681	-1.026335	0.37622	-2.72798	0.006372258	0.039051069
MX1	42.66893581	-1.02572	0.20897	-4.90838	9.18E-07	1.58E-05
DENND4A	105.973093	-1.02189	0.14161	-7.21615	5.35E-13	1.96E-11
SLA	165.2349494	-1.021758	0.11488	-8.89386	5.90E-19	3.40E-17
AC004520.1	17.8859877	-1.014857	0.33148	-3.06163	0.002201382	0.016414484
AHR	57.36038704	-1.01094	0.18084	-5.59023	2.27E-08	4.89E-07
CDC14A	76.58885559	-1.010296	0.17162	-5.88671	3.94E-09	9.31E-08
MAGIX	24.15583721	-1.009487	0.28731	-3.51358	0.000442115	0.004153323
TMEM39A	19.88061353	-0.99786	0.32271	-3.09217	0.00198699	0.015078575
OGT	121.7840488	-0.99628	0.14265	-6.98399	2.87E-12	9.65E-11
PCIF1	38.41402908	-0.995807	0.23733	-4.19581	2.72E-05	0.00035741
WDR74	65.58899691	-0.994792	0.17407	-5.71488	1.10E-08	2.49E-07
PATJ	43.8776016	-0.993692	0.21671	-4.58543	4.53E-06	6.96E-05
SUPV3L1	39.33758916	-0.987691	0.22464	-4.39686	1.10E-05	0.000156686
CDK5RAP1	29.94161163	-0.986509	0.25665	-3.84377	0.000121158	0.001364617
FOSB	1738.63727	-0.986148	0.05005	-19.7041	1.99E-86	7.09E-84
RBM12	45.16477163	-0.97839	0.20388	-4.79893	1.60E-06	2.64E-05
AL049840.1	18.02923338	-0.977637	0.32507	-3.0075	0.002634053	0.019139311
CEBPZ	157.6951364	-0.975495	0.11341	-8.6015	7.87E-18	4.07E-16
SEMA4A	40.56064201	-0.975463	0.21415	-4.55504	5.24E-06	7.92E-05
MT2A	62351.18926	-0.974433	0.04575	-21.3005	1.12E-100	5.01E-98
CSNK1D	127.735035	-0.974062	0.12321	-7.90577	2.66E-15	1.21E-13
POLR2A	180.3377601	-0.973402	0.10451	-9.31352	1.24E-20	7.78E-19
ITK	120.1328321	-0.973115	0.13519	-7.19826	6.10E-13	2.22E-11

PSME4	68.93051995	-0.972717	0.17305	-5.62104	1.90E-08	4.18E-07
GPR132	32.07647487	-0.969782	0.24883	-3.89731	9.73E-05	0.001125135
CRIP2	21.91125579	-0.96974	0.29574	-3.27904	0.001041592	0.008626188
LEPROTL1	471.1559893	-0.968134	0.06912	-14.0072	1.41E-44	2.22E-42
ARID5A	119.3448327	-0.968111	0.12878	-7.51782	5.57E-14	2.30E-12
MYADM	396.4270368	-0.966365	0.0745	-12.9715	1.78E-38	2.29E-36
AKAP13	364.3132684	-0.96413	0.07779	-12.3937	2.83E-35	3.22E-33
TIMM23B	16.09658961	-0.962361	0.34145	-2.81843	0.004825958	0.031409824
MAT2A	206.2105281	-0.955583	0.11009	-8.68005	3.96E-18	2.13E-16
ATXN7.1	14.09556857	-0.954461	0.36062	-2.64671	0.008127869	0.047265324
RILPL2	81.81287244	-0.951441	0.15214	-6.25364	4.01E-10	1.08E-08
FAM83D	17.08010539	-0.950908	0.32875	-2.89252	0.003821592	0.026012109
EMP1	19.20921911	-0.948061	0.30938	-3.06438	0.002181201	0.016309472
HEXIM1	272.183398	-0.947991	0.08998	-10.5352	5.95E-26	4.86E-24
PLIN2	159.5002059	-0.944038	0.11224	-8.41064	4.08E-17	2.01E-15
GOLGB1	273.7220086	-0.941776	0.08783	-10.7224	7.99E-27	6.84E-25
TGIF2	40.02709692	-0.941688	0.22086	-4.26382	2.01E-05	0.000269463
XRRA1	45.5515521	-0.941477	0.20294	-4.63912	3.50E-06	5.53E-05
PLXND1	30.37125471	-0.936236	0.24954	-3.75189	0.000175509	0.001893095
NDRG1	79.70705236	-0.935796	0.15625	-5.98921	2.11E-09	5.13E-08
AC005261.1	34.25572585	-0.935633	0.23848	-3.92339	8.73E-05	0.001024366
AC103591.3	66.54770104	-0.935294	0.18931	-4.94064	7.79E-07	1.35E-05
GLUL	117.1247371	-0.933578	0.13103	-7.12492	1.04E-12	3.67E-11
LINC01871	218.9881126	-0.932316	0.09782	-9.53139	1.55E-21	1.05E-19
RELB	63.66294459	-0.931647	0.17256	-5.39885	6.71E-08	1.35E-06
AC007384.1	21.63695213	-0.931086	0.2976	-3.1287	0.001755832	0.013545351
PDE4A	55.60843298	-0.929944	0.18176	-5.11638	3.11E-07	5.72E-06
NCK2	92.93307688	-0.928098	0.157	-5.91132	3.39E-09	8.11E-08
RFX3	17.88861705	-0.92469	0.32233	-2.86881	0.004120248	0.027727453
TLE4	258.3479295	-0.924387	0.09744	-9.48697	2.38E-21	1.57E-19
AC092683.1	48.49099446	-0.924153	0.20017	-4.61688	3.90E-06	6.08E-05
CYTIP	264.550884	-0.923329	0.08772	-10.5264	6.53E-26	5.29E-24
HECA	127.2118991	-0.923018	0.12552	-7.35384	1.93E-13	7.58E-12
ACSL6	30.95820243	-0.922905	0.24422	-3.77903	0.000157443	0.001724299
UBL3	75.28716123	-0.920728	0.16965	-5.42707	5.73E-08	1.17E-06
GAN	15.85375266	-0.920396	0.33789	-2.72395	0.006450699	0.039424838
AL035071.1	20.51785275	-0.919178	0.30687	-2.99535	0.002741315	0.019818965
ERO1B	84.11199715	-0.918672	0.15065	-6.09819	1.07E-09	2.72E-08
MYBL1	144.379377	-0.917283	0.12322	-7.44412	9.76E-14	3.93E-12
PLK3	101.969668	-0.91609	0.13977	-6.55444	5.58E-11	1.66E-09
PCNX1	92.61470252	-0.915424	0.14498	-6.31421	2.72E-10	7.49E-09
CAMK4	84.75481819	-0.91378	0.15502	-5.89465	3.75E-09	8.95E-08
MKNK2	128.1843529	-0.912813	0.12278	-7.43463	1.05E-13	4.17E-12
CTNNB1	111.7354757	-0.912041	0.14307	-6.37499	1.83E-10	5.21E-09
AAED1	80.02945961	-0.910671	0.15554	-5.855	4.77E-09	1.12E-07
MAP2K1	48.67440622	-0.909986	0.19532	-4.65902	3.18E-06	5.04E-05
OXNAD1	134.797006	-0.905918	0.12836	-7.05778	1.69E-12	5.84E-11
RIPK2	21.44657639	-0.903362	0.29287	-3.08452	0.002038832	0.015439136
SIK3	73.4978407	-0.903184	0.16126	-5.60086	2.13E-08	4.61E-07
N4BP2	43.16955589	-0.902976	0.21454	-4.20897	2.57E-05	0.000338889
DDX27	119.646497	-0.900675	0.1299	-6.93349	4.11E-12	1.37E-10
PABPC4	102.7160531	-0.900017	0.14172	-6.35074	2.14E-10	6.02E-09

PTGER4	186.9573561	-0.8997	0.11141	-8.07561	6.71E-16	3.14E-14
AKT3	45.16263484	-0.898906	0.20519	-4.38087	1.18E-05	0.000167749
BICDL1	51.54768057	-0.895943	0.19558	-4.58088	4.63E-06	7.08E-05
SDR42E2	29.05541633	-0.895478	0.24866	-3.60121	0.000316742	0.003117462
SRSF6	227.9659065	-0.893027	0.09457	-9.44331	3.61E-21	2.37E-19
KHNYN	21.15762659	-0.890901	0.30012	-2.96845	0.002993076	0.021364852
ST3GAL1	90.36032268	-0.888359	0.14645	-6.06576	1.31E-09	3.31E-08
LDLR	41.0725593	-0.888217	0.22168	-4.00682	6.15E-05	0.000748288
SLC16A3	34.18909283	-0.886401	0.23064	-3.84326	0.000121412	0.001366041
SLA2	71.53717546	-0.885907	0.16545	-5.35449	8.58E-08	1.69E-06
MAPKAPK2	74.13772713	-0.884839	0.16394	-5.3975	6.76E-08	1.36E-06
HIF1A	92.87315898	-0.884593	0.15072	-5.86923	4.38E-09	1.03E-07
FBXO34	64.45522616	-0.884179	0.1712	-5.16462	2.41E-07	4.48E-06
CREM	865.6561624	-0.88395	0.06114	-14.4571	2.26E-47	3.78E-45
TP53INP1	17.7581837	-0.882926	0.319	-2.76776	0.005644358	0.035505371
TMEM181	47.38752372	-0.881195	0.1984	-4.44143	8.94E-06	0.000129916
MXI1	73.33171423	-0.880939	0.15937	-5.52771	3.24E-08	6.90E-07
RLF	75.26969651	-0.880415	0.15937	-5.5245	3.30E-08	7.02E-07
ATL2	27.37177181	-0.879284	0.26475	-3.3212	0.000896318	0.007581507
CFH	27.04053017	-0.878181	0.26345	-3.33341	0.000857886	0.007296808
IL6ST	86.18439483	-0.875667	0.14949	-5.85776	4.69E-09	1.10E-07
SMARCA2	137.1518311	-0.874717	0.12673	-6.90246	5.11E-12	1.69E-10
ZBTB10	29.1604346	-0.868929	0.2516	-3.4536	0.000553157	0.005003195
BACH1	38.36637732	-0.868676	0.23159	-3.75099	0.000176135	0.001896016
PPP1R16B	54.8172568	-0.868404	0.18906	-4.59336	4.36E-06	6.73E-05
RCAN3	115.1816722	-0.86802	0.13008	-6.67281	2.51E-11	7.85E-10
RUNX3	615.9322144	-0.864957	0.06124	-14.1231	2.74E-45	4.44E-43
TP53BP2	56.54956117	-0.863747	0.18453	-4.68092	2.86E-06	4.58E-05
B4GALT4	42.8997586	-0.863726	0.21301	-4.05495	5.01E-05	0.000621
MAN2A1	64.31225859	-0.862444	0.17948	-4.80529	1.55E-06	2.57E-05
UBXN11	23.45527873	-0.858904	0.28518	-3.01175	0.002597422	0.018919277
ZFYVE27	18.11982772	-0.855567	0.31683	-2.70036	0.006926381	0.041706404
YBX3	134.2731036	-0.854389	0.11864	-7.20151	5.95E-13	2.17E-11
LRRC75A	42.50931227	-0.854056	0.21021	-4.06296	4.85E-05	0.000600756
SLC1A5	69.23560966	-0.853339	0.16887	-5.05327	4.34E-07	7.82E-06
GABARAPL1	260.2654349	-0.853329	0.09454	-9.02647	1.77E-19	1.05E-17
EXOSC6	101.1939574	-0.852591	0.13769	-6.1921	5.94E-10	1.58E-08
DNM1L	71.20212114	-0.850825	0.16872	-5.0428	4.59E-07	8.19E-06
CTH	21.71261419	-0.850785	0.29991	-2.83681	0.004556632	0.030077708
MFSD14C	35.93132158	-0.848854	0.23773	-3.5706	0.000356163	0.003451942
TBC1D15	66.92412783	-0.847931	0.17007	-4.98564	6.18E-07	1.08E-05
NPHP3	25.82439173	-0.844218	0.26762	-3.15448	0.001607824	0.012557456
ELF1	442.0313474	-0.84396	0.07062	-11.9512	6.40E-33	6.78E-31
THBS1	45.62333362	-0.842674	0.21876	-3.85203	0.000117142	0.001323568
CORO7	92.05484585	-0.8417	0.15566	-5.40745	6.39E-08	1.30E-06
STAT4	167.2032905	-0.841661	0.11213	-7.50607	6.09E-14	2.50E-12
SAMSN1	421.9248804	-0.839829	0.07154	-11.7394	8.00E-32	7.86E-30
HGSNAT	34.86441135	-0.839467	0.22781	-3.685	0.0002287	0.00235071
ATP8A1	60.6848536	-0.836881	0.17889	-4.67827	2.89E-06	4.63E-05
AC243829.1	25.20355308	-0.833511	0.28596	-2.91474	0.003559881	0.024606416
MT1F	1298.960368	-0.83341	0.09524	-8.75027	2.13E-18	1.19E-16
CPEB2	23.17358189	-0.82801	0.28169	-2.93946	0.003287893	0.023099447

AL355075.4	22.72993449	-0.827338	0.29654	-2.78996	0.005271476	0.033594278
ANKRD36B	39.63031427	-0.827326	0.22268	-3.71524	0.000203008	0.002131686
CD44	537.4084759	-0.827298	0.06424	-12.8791	5.90E-38	7.52E-36
JUND	5222.372419	-0.826817	0.04205	-19.662	4.56E-86	1.58E-83
CELF2	351.7835968	-0.825406	0.07709	-10.7066	9.47E-27	8.05E-25
BLMH	23.15143464	-0.825397	0.28172	-2.92989	0.003390802	0.023713454
PABPC1	940.5501669	-0.824731	0.05401	-15.2699	1.21E-52	2.36E-50
SLC35D1	23.47547045	-0.823136	0.28604	-2.87772	0.004005608	0.027092289
OFD1	157.583331	-0.819041	0.11316	-7.2376	4.57E-13	1.70E-11
HIST1H1D	69.14642171	-0.81818	0.17923	-4.56493	5.00E-06	7.58E-05
TMEM71	41.21717642	-0.815641	0.21388	-3.81347	0.000137031	0.001518295
HNRNPUL1	185.880344	-0.814336	0.10576	-7.70019	1.36E-14	5.93E-13
SPRY1	44.43825537	-0.814226	0.20542	-3.96378	7.38E-05	0.000877083
AP3M2	23.43679397	-0.813097	0.27933	-2.91092	0.003603631	0.024844618
IKZF1	245.0737423	-0.812427	0.09031	-8.99586	2.34E-19	1.37E-17
SRSF2	915.9212024	-0.812085	0.05451	-14.8977	3.41E-50	6.29E-48
NUTM2A-AS1	22.82282891	-0.812077	0.29452	-2.75727	0.005828654	0.036471693
PTPN1	101.2012367	-0.811548	0.137	-5.92391	3.14E-09	7.55E-08
SH3YL1	26.5891253	-0.811361	0.27147	-2.98879	0.002800805	0.020153741
MIR22HG	104.2593411	-0.81096	0.13937	-5.81857	5.94E-09	1.38E-07
JMJD1C	203.879945	-0.809246	0.10489	-7.7153	1.21E-14	5.31E-13
MIR155HG	40.2853619	-0.807215	0.22679	-3.55937	0.000371749	0.003583528
AKNA	232.0345043	-0.806013	0.0932	-8.64803	5.24E-18	2.75E-16
ATF7IP	153.8958975	-0.80436	0.11226	-7.16485	7.79E-13	2.81E-11
DYNLT1	131.5004554	-0.80377	0.12154	-6.61347	3.75E-11	1.16E-09
CLU	82.21900505	-0.799637	0.15283	-5.23209	1.68E-07	3.19E-06
LINC00685	25.66275562	-0.798727	0.27592	-2.89481	0.003793858	0.025872706
ZFAND5	323.448933	-0.796408	0.08542	-9.32369	1.12E-20	7.11E-19
MAPK8	39.2134839	-0.795987	0.21861	-3.6411	0.000271471	0.002737743
AC044849.1	152.0167787	-0.794789	0.11621	-6.83952	7.95E-12	2.59E-10
GOLGA8A	49.99011671	-0.794282	0.19746	-4.02244	5.76E-05	0.000705165
MGAT4A	124.9275125	-0.793045	0.12612	-6.28782	3.22E-10	8.74E-09
PBXIP1	140.5826166	-0.792831	0.11622	-6.8219	8.98E-12	2.91E-10
POR	28.77231115	-0.792061	0.25192	-3.14406	0.001666198	0.012947214
JMY	133.4481996	-0.787283	0.12009	-6.5556	5.54E-11	1.66E-09
AC097376.2	50.61188699	-0.786024	0.19719	-3.98614	6.72E-05	0.000806497
FOS	4049.758141	-0.781425	0.04196	-18.6226	2.11E-77	6.27E-75
ERRFI1	37.49909171	-0.780888	0.22694	-3.441	0.00057957	0.005215643
KIAA1147	22.76571426	-0.779985	0.2884	-2.70454	0.006839958	0.041278935
PIEZO1	47.02876097	-0.779771	0.21176	-3.6824	0.000231045	0.002368806
NFKB2	100.3280119	-0.778511	0.13872	-5.61228	2.00E-08	4.36E-07
FAM107B	289.2373392	-0.777717	0.08691	-8.94809	3.62E-19	2.09E-17
SPOCK2	234.5247895	-0.776988	0.10075	-7.71238	1.23E-14	5.42E-13
PDCL3	151.944611	-0.775741	0.1162	-6.67586	2.46E-11	7.71E-10
METRNL	421.1892492	-0.775519	0.07394	-10.4879	9.82E-26	7.78E-24
TRPS1	38.00191947	-0.773511	0.23102	-3.34826	0.000813195	0.006994524
PTAR1	35.33400021	-0.772887	0.23377	-3.30613	0.000945925	0.007950827
HERPUD1	584.5725075	-0.771602	0.06542	-11.794	4.19E-32	4.21E-30
STAT3	177.4323378	-0.768882	0.10702	-7.18463	6.74E-13	2.44E-11
SLC12A2	45.61622001	-0.768508	0.2073	-3.70725	0.000209523	0.002182953
RSRP1	726.2621723	-0.766974	0.06491	-11.8156	3.24E-32	3.34E-30
PCNX4	54.73961405	-0.766288	0.1852	-4.13762	3.51E-05	0.00044701

ABCG1	27.19901754	-0.765584	0.25989	-2.94583	0.00322091	0.022731272
JOSD1	245.2062883	-0.765141	0.09723	-7.86969	3.56E-15	1.61E-13
NBPF19	37.95950535	-0.76469	0.22062	-3.46611	0.000528057	0.004808687
CCDC6	41.60718229	-0.762287	0.21092	-3.61413	0.000301355	0.002993959
FKBP11	182.5722129	-0.761976	0.10325	-7.38025	1.58E-13	6.24E-12
EGR1	527.5454657	-0.76109	0.07686	-9.90242	4.06E-23	2.88E-21
OSBPL8	170.4234922	-0.759198	0.10854	-6.99435	2.66E-12	9.02E-11
CNOT6	24.3220016	-0.758895	0.28005	-2.70987	0.006731028	0.040759478
XCL1	292.4885425	-0.757638	0.24754	-3.06065	0.002208579	0.016456682
SYTL2	95.89025336	-0.757163	0.14589	-5.18991	2.10E-07	3.96E-06
KCTD9	38.1714186	-0.756816	0.22477	-3.36706	0.00075975	0.006587783
MRPL23	29.70039761	-0.75527	0.24567	-3.07435	0.002109589	0.015907397
ITGA6	27.60247076	-0.754826	0.26422	-2.85677	0.004279761	0.028620901
GLIPR1	238.043784	-0.753728	0.09263	-8.13732	4.04E-16	1.91E-14
ID1	76.64766348	-0.75363	0.17265	-4.36499	1.27E-05	0.00017818
USP11	39.45477587	-0.752894	0.22171	-3.39581	0.00068426	0.00601609
PLXNA3	37.81109013	-0.752854	0.22161	-3.39727	0.000680608	0.0059889
HIPK1	106.1117543	-0.752788	0.13392	-5.62137	1.89E-08	4.18E-07
RGCC	893.7131712	-0.751178	0.05267	-14.2626	3.74E-46	6.16E-44
MT1G	2667.305798	-0.750466	0.18201	-4.12317	3.74E-05	0.000473208
BRAF	95.08970223	-0.747586	0.14222	-5.25661	1.47E-07	2.83E-06
SEC22C	47.26442569	-0.746349	0.19608	-3.80629	0.000141068	0.001554512
PRKAR2A	75.58395139	-0.744219	0.1567	-4.74931	2.04E-06	3.32E-05
SIPA1L1	42.91411423	-0.74236	0.20776	-3.57324	0.000352594	0.003420447
EML4	299.2361489	-0.741426	0.0871	-8.51267	1.70E-17	8.54E-16
ADNP2	39.13603362	-0.741207	0.21733	-3.41059	0.000648215	0.005737186
TUBD1	26.99647615	-0.738152	0.26524	-2.78296	0.005386578	0.034205572
KLF13	228.0045425	-0.737431	0.09694	-7.60707	2.80E-14	1.19E-12
TUBA4A	658.2231076	-0.736885	0.07767	-9.48733	2.37E-21	1.57E-19
COG3	40.02055045	-0.736376	0.21518	-3.42217	0.000621228	0.005539284
MT1M	249.1090639	-0.735251	0.22886	-3.21268	0.001315021	0.0105241
LRRFIP1	304.0204696	-0.735091	0.08411	-8.74014	2.33E-18	1.28E-16
SGK3	39.48516993	-0.734921	0.21557	-3.40913	0.000651695	0.005762921
RASSF2	32.50265997	-0.733538	0.24211	-3.02982	0.002446994	0.017981643
CCL3L1	162.2172425	-0.733139	0.1979	-3.70467	0.000211663	0.002198831
AHNAK	579.9381553	-0.731504	0.06203	-11.7936	4.21E-32	4.21E-30
FAS	78.85187818	-0.730848	0.15591	-4.68767	2.76E-06	4.45E-05
MGAT5	32.51803764	-0.730499	0.24258	-3.0114	0.002600445	0.018928406
PDE4B	141.4117346	-0.730362	0.12322	-5.92744	3.08E-09	7.43E-08
EPC1	214.6544059	-0.729894	0.0996	-7.32851	2.33E-13	9.09E-12
SPTY2D1	242.6510978	-0.729355	0.09785	-7.45384	9.07E-14	3.69E-12
FOXN2	51.24660411	-0.72926	0.19624	-3.71614	0.000202286	0.002126193
GALNT11	52.05230972	-0.727662	0.19122	-3.80543	0.000141561	0.001557187
RBMS1	165.817076	-0.721714	0.11166	-6.46329	1.02E-10	2.98E-09
MALAT1	83758.94755	-0.720795	0.03648	-19.7571	6.98E-87	2.57E-84
ZNF10	38.9229126	-0.720619	0.2153	-3.34697	0.000816988	0.007005369
MIGA1	26.83413864	-0.719855	0.27016	-2.66456	0.007708831	0.045321149
KAT6B	103.5740903	-0.718452	0.13715	-5.23825	1.62E-07	3.09E-06
NCOA2	61.20498421	-0.718162	0.1737	-4.13454	3.56E-05	0.000451971
VAV3	32.29146249	-0.716512	0.25592	-2.79978	0.005113763	0.032863219
TMF1	122.3331899	-0.716	0.13012	-5.50253	3.74E-08	7.87E-07
PLCB1	27.38371094	-0.714715	0.26936	-2.65343	0.007967807	0.046511477

IFT57	35.02261699	-0.713496	0.2417	-2.95193	0.003157953	0.022392374
HIVEP2	45.68150673	-0.712166	0.20209	-3.52395	0.000425162	0.004022311
CENPC	143.7685068	-0.711497	0.12206	-5.82928	5.57E-09	1.30E-07
RARA	37.97861984	-0.711231	0.23241	-3.06023	0.002211696	0.016468439
CD28	52.62387144	-0.710706	0.1936	-3.67108	0.00024153	0.002463651
MT-ND2	4009.671907	-0.70924	0.04203	-16.875	6.88E-64	1.57E-61
PIM3	222.2331673	-0.707856	0.09856	-7.18197	6.87E-13	2.48E-11
RPS6KB1	45.2395736	-0.707141	0.20637	-3.4265	0.000611412	0.005468812
DPP4	39.13609065	-0.705921	0.23634	-2.9869	0.002818188	0.020265196
LITAF	407.5109657	-0.70457	0.07373	-9.5565	1.22E-21	8.30E-20
SOS2	42.16803361	-0.704438	0.21371	-3.29628	0.000979738	0.008177224
RPGR	26.50540348	-0.703842	0.26827	-2.62359	0.008700761	0.049758493
MT-ND5	1245.607877	-0.703628	0.05117	-13.7511	5.02E-43	7.35E-41
CRTC3	26.60670666	-0.703627	0.26032	-2.7029	0.00687377	0.041459604
ZBTB21	57.57684017	-0.702647	0.17815	-3.94417	8.01E-05	0.000947823
PPP3CA	114.0411673	-0.702647	0.12794	-5.49213	3.97E-08	8.28E-07
CYTH1	138.5736497	-0.701082	0.11654	-6.01589	1.79E-09	4.40E-08
RBM39	748.9986568	-0.700517	0.05733	-12.2182	2.48E-34	2.74E-32
VIM	2313.016791	-0.699764	0.04678	-14.959	1.36E-50	2.55E-48
SENP5	38.86029138	-0.699538	0.22116	-3.16305	0.001561271	0.012265493
CUX1	41.96070921	-0.699516	0.21359	-3.27503	0.001056519	0.0087228
ARHGEF7	29.34597379	-0.698386	0.25192	-2.77228	0.005566467	0.035146541
TERF2IP	267.8528355	-0.697729	0.08883	-7.85447	4.01E-15	1.80E-13
DDX24	689.9184986	-0.697489	0.06553	-10.6432	1.88E-26	1.58E-24
CASK	69.55791681	-0.69629	0.16993	-4.09743	4.18E-05	0.000525264
DDX3X	727.7122518	-0.696251	0.06429	-10.8302	2.48E-27	2.19E-25
TRA2A	249.3625721	-0.695282	0.09238	-7.52652	5.21E-14	2.16E-12
CREBBP	68.46069578	-0.69442	0.16604	-4.18221	2.89E-05	0.00037762
PRDM2	179.976266	-0.694181	0.1052	-6.59866	4.15E-11	1.27E-09
IVNS1ABP	188.915959	-0.692034	0.10605	-6.52558	6.77E-11	2.00E-09
MAP3K5	39.92213329	-0.691692	0.21868	-3.16306	0.001561185	0.012265493
QRICH1	37.45277781	-0.691562	0.22323	-3.09795	0.001948659	0.014840321
BTG2	1649.907544	-0.689342	0.05457	-12.6318	1.41E-36	1.70E-34
PLEKHA2	103.0069899	-0.686906	0.14055	-4.8874	1.02E-06	1.74E-05
CFAP20	57.08563572	-0.685539	0.18995	-3.60903	0.000307348	0.003039398
RB1CC1	117.1594167	-0.683926	0.1373	-4.98122	6.32E-07	1.10E-05
CHPT1	33.21677396	-0.680158	0.23272	-2.92268	0.003470371	0.024096674
MT-ND6	55.00403066	-0.678929	0.18235	-3.7232	0.00019671	0.002073688
PDCD4	377.0003939	-0.678344	0.08022	-8.45603	2.77E-17	1.38E-15
IDS	336.926024	-0.676178	0.0803	-8.42016	3.76E-17	1.86E-15
SLCO3A1	44.06898573	-0.673095	0.20292	-3.3171	0.000909555	0.007669216
AC026979.2	86.30583455	-0.670753	0.14986	-4.47579	7.61E-06	0.000111631
RBM38	240.978238	-0.670073	0.09371	-7.15036	8.66E-13	3.08E-11
TIMM44	29.57494779	-0.668576	0.24739	-2.70248	0.006882509	0.041488927
CLDND1	146.0382979	-0.668177	0.12182	-5.48511	4.13E-08	8.59E-07
CLK4	34.10325597	-0.666842	0.23021	-2.89672	0.003770834	0.025768708
OAT	123.889498	-0.666616	0.13735	-4.85323	1.21E-06	2.04E-05
TTPAL	32.16875904	-0.666179	0.23836	-2.79485	0.005192315	0.033248218
ETFDH	30.12750412	-0.665838	0.25042	-2.65891	0.007839327	0.045932196
DARS	71.69324756	-0.662483	0.1695	-3.90848	9.29E-05	0.001079034
RALGAPA1	104.438641	-0.661371	0.13636	-4.85027	1.23E-06	2.06E-05
PRKX	53.02562131	-0.659581	0.18426	-3.57971	0.000343981	0.003352096

TNFRSF1B	231.5155401	-0.659137	0.0952	-6.92364	4.40E-12	1.46E-10
NAP1L4	162.4461725	-0.658232	0.10892	-6.04322	1.51E-09	3.78E-08
FAM102A	66.89142772	-0.657596	0.17713	-3.71257	0.000205166	0.002148025
NAP1L1	461.5695792	-0.656903	0.06799	-9.6622	4.36E-22	3.01E-20
C3orf58	51.45121199	-0.656376	0.19559	-3.35581	0.000791334	0.006833957
MSI2	64.41843285	-0.656337	0.17511	-3.74818	0.000178122	0.00191164
FUS	795.7740498	-0.655825	0.05742	-11.4211	3.28E-30	3.13E-28
ELK3	33.37901217	-0.655785	0.24134	-2.71732	0.006581364	0.040011702
CDK17	55.40926766	-0.654151	0.18687	-3.50063	0.000464157	0.004326202
FTX	51.84217144	-0.652687	0.20664	-3.1585	0.001585823	0.01243099
DDX3Y	506.4647644	-0.652151	0.08123	-8.02834	9.88E-16	4.58E-14
ZNF506	56.4209865	-0.649568	0.18715	-3.47092	0.00051868	0.004738985
ZNF407	37.03750327	-0.648299	0.22578	-2.87133	0.004087476	0.027539906
TSPYL1	152.0838188	-0.647498	0.11409	-5.67527	1.38E-08	3.11E-07
SLC38A2	291.9198494	-0.646606	0.08871	-7.28925	3.12E-13	1.20E-11
USP8	148.0357534	-0.646164	0.11412	-5.66194	1.50E-08	3.34E-07
TARSL2	53.98904216	-0.646091	0.18897	-3.41909	0.00062831	0.005588458
6-Mar	91.03737697	-0.645018	0.14789	-4.3615	1.29E-05	0.000180201
SNHG9	96.16946805	-0.644466	0.14266	-4.51763	6.25E-06	9.31E-05
ATP9B	30.69414428	-0.643535	0.24109	-2.66928	0.007601356	0.044787725
DUSP1	2030.502293	-0.643421	0.04671	-13.7754	3.58E-43	5.40E-41
MED13L	45.27495172	-0.643314	0.20346	-3.16186	0.001567675	0.012306768
CPD	49.37174101	-0.643261	0.20038	-3.21016	0.001326612	0.010601008
KIAA1551	416.1164161	-0.641127	0.07431	-8.62751	6.27E-18	3.27E-16
TMX4	182.8618399	-0.640868	0.10468	-6.12192	9.25E-10	2.38E-08
EMB	269.8364663	-0.640538	0.08842	-7.24396	4.36E-13	1.63E-11
PAF1	63.35942899	-0.639858	0.19449	-3.28989	0.001002256	0.008345634
TUBB4B	866.5401221	-0.638931	0.05752	-11.1084	1.14E-28	1.04E-26
SH2D2A	136.1798235	-0.638646	0.13182	-4.84479	1.27E-06	2.12E-05
MTO1	35.05520112	-0.638624	0.23177	-2.75546	0.005860984	0.036588406
SORL1	77.71881371	-0.638543	0.15783	-4.04566	5.22E-05	0.000642452
PPP1R15B	101.8319556	-0.638069	0.14148	-4.50994	6.48E-06	9.62E-05
DYNLL2	137.7826133	-0.637887	0.12009	-5.31162	1.09E-07	2.12E-06
CCSER2	149.5607711	-0.637771	0.1161	-5.4935	3.94E-08	8.23E-07
ZNF644	173.228117	-0.63756	0.10556	-6.03992	1.54E-09	3.85E-08
CASP8	150.7028592	-0.636998	0.11572	-5.5046	3.70E-08	7.79E-07
AKAP9	260.2933228	-0.634091	0.0961	-6.59843	4.16E-11	1.27E-09
MXRA7	64.23099917	-0.633298	0.17064	-3.71139	0.000206124	0.002155941
GOLGA4	186.5486213	-0.632848	0.11267	-5.61694	1.94E-08	4.26E-07
KLF2	726.8738819	-0.631936	0.07251	-8.71569	2.89E-18	1.57E-16
TAGLN2	406.253159	-0.631751	0.07225	-8.74403	2.25E-18	1.25E-16
FOSL2	277.5957464	-0.630996	0.08669	-7.27837	3.38E-13	1.28E-11
XCL2	472.8978126	-0.630958	0.14904	-4.23338	2.30E-05	0.00030599
HMGB2	313.8158101	-0.630472	0.08717	-7.23257	4.74E-13	1.76E-11
RANBP2	220.4003608	-0.628844	0.09647	-6.51846	7.10E-11	2.09E-09
INO80D	79.34289862	-0.628229	0.1565	-4.0143	5.96E-05	0.000727437
TMEM41B	40.12825618	-0.627819	0.22243	-2.82255	0.004764341	0.031141387
OGFRL1	36.70392605	-0.627135	0.22832	-2.74668	0.006020139	0.037407369
GINM1	45.06492552	-0.626084	0.21076	-2.97063	0.002971917	0.021256361
MDM4	133.6515143	-0.624975	0.12453	-5.01862	5.20E-07	9.22E-06
SHLD2	34.20647213	-0.620024	0.23267	-2.66487	0.00770181	0.045304766
PGRMC1	48.66802152	-0.619641	0.19934	-3.10853	0.001880182	0.014411138

RERE	41.03450871	-0.617885	0.21335	-2.89617	0.003777491	0.025790414
ARIH1	228.3725243	-0.617433	0.09699	-6.36594	1.94E-10	5.49E-09
ADSS	110.5225331	-0.616299	0.13194	-4.67115	3.00E-06	4.78E-05
CRELD2	66.8460528	-0.615743	0.16798	-3.66554	0.00024682	0.002512824
KCTD20	39.36114785	-0.615221	0.21763	-2.82695	0.004699322	0.030811075
RASGEF1B	107.8832312	-0.614375	0.13593	-4.5197	6.19E-06	9.24E-05
TLE3	69.52161964	-0.614136	0.16261	-3.77676	0.000158882	0.001736502
AKIRIN2	85.26459269	-0.614134	0.15964	-3.84707	0.000119539	0.001347807
RBBP5	39.25643301	-0.614006	0.21514	-2.85397	0.004317692	0.02880256
MED29	64.72859634	-0.613706	0.1689	-3.63363	0.000279465	0.002805141
PNPLA2	79.38493735	-0.612914	0.1528	-4.01126	6.04E-05	0.000736025
ELOVL5	95.62308559	-0.612599	0.13909	-4.40448	1.06E-05	0.000151887
PGGHG	64.80573571	-0.611114	0.17145	-3.56436	0.000364744	0.003525528
STX2	34.87638319	-0.609945	0.23264	-2.62188	0.008744621	0.049955922
SLC35A3	35.90943638	-0.608545	0.22367	-2.72068	0.006514709	0.03965153
RASA2	123.8851311	-0.608455	0.13126	-4.63535	3.56E-06	5.62E-05
CCNT1	88.76907917	-0.607731	0.1468	-4.13976	3.48E-05	0.000443398
NDFIP1	150.4404221	-0.606971	0.11131	-5.45294	4.95E-08	1.02E-06
ABHD5	144.536819	-0.606609	0.11613	-5.22357	1.76E-07	3.32E-06
CMIP	66.52223509	-0.605853	0.18641	-3.25012	0.001153563	0.009415047
VEZT	43.26513161	-0.605633	0.2056	-2.94565	0.003222742	0.022731272
SMURF2	60.633114	-0.605216	0.17287	-3.50105	0.000463435	0.004323239
DDX60L	34.81290499	-0.605058	0.22611	-2.676	0.00745077	0.04404599
CAMK2N1	61.26547003	-0.604734	0.18743	-3.22646	0.001253322	0.010113531
DCTN6	69.80852723	-0.604259	0.16321	-3.7024	0.00021357	0.002216486
KDSR	35.32330845	-0.60337	0.22523	-2.67891	0.007386142	0.04376064
KLF10	138.398418	-0.60246	0.12121	-4.97031	6.68E-07	1.16E-05
IQGAP2	253.341331	-0.602129	0.09008	-6.68422	2.32E-11	7.31E-10
ZBTB25	42.01090745	-0.601911	0.21285	-2.8279	0.004685434	0.030782324
MTFP1	49.2511871	-0.601723	0.19588	-3.07192	0.002126899	0.016004091
SCML4	67.35888463	-0.601713	0.16645	-3.61502	0.000300321	0.002986465
WDR47	40.8740507	-0.601421	0.2112	-2.84767	0.004404033	0.029250872
ZNF267	80.63974616	-0.600237	0.1626	-3.69147	0.000222957	0.002300525
TENT4B	56.93495705	-0.599943	0.1794	-3.34418	0.000825272	0.00705868
CCL4	3963.173492	-0.597935	0.19524	-3.0626	0.002194207	0.016383822
TSC22D2	135.1649248	-0.597532	0.12232	-4.88493	1.03E-06	1.75E-05
FBXO21	47.05990934	-0.597511	0.19821	-3.01452	0.002573865	0.018798881
TUBB2A	103.9540439	-0.597452	0.13933	-4.28797	1.80E-05	0.00024361
TNFRSF10B	44.97387596	-0.597174	0.20117	-2.96854	0.002992135	0.021364852
SNTB2	68.61113827	-0.597169	0.16439	-3.63261	0.000280574	0.002813629
MTHFD2	44.6091284	-0.59629	0.19934	-2.99133	0.00277769	0.020030266
NKAP	85.29783504	-0.594958	0.15929	-3.73495	0.00018775	0.002000921
ALG13	182.8922643	-0.594159	0.11467	-5.18167	2.20E-07	4.12E-06
ETV3	140.1407231	-0.593541	0.12866	-4.61315	3.97E-06	6.16E-05
ANKH	40.24058308	-0.593184	0.21196	-2.79854	0.005133422	0.03294998
MYH9	308.8298789	-0.592674	0.08389	-7.0651	1.60E-12	5.56E-11
ATF3	251.2138661	-0.591259	0.16026	-3.68947	0.000224723	0.002314278
SF3A1	61.10942418	-0.590672	0.1774	-3.3296	0.000869713	0.007373952
GNA13	83.97951441	-0.590023	0.14794	-3.98822	6.66E-05	0.000801237
BTAF1	36.86811482	-0.589644	0.22026	-2.67702	0.007427956	0.043935392
DUSP10	77.30869777	-0.589456	0.15449	-3.81551	0.000135903	0.001508464
EVI2A	122.960492	-0.587665	0.12407	-4.73656	2.17E-06	3.52E-05

MT-ND4L	410.3360743	-0.587471	0.07261	-8.09084	5.93E-16	2.78E-14
NR1D2	85.13967874	-0.586732	0.15537	-3.77647	0.000159067	0.001736757
MBNL2	55.22353661	-0.586343	0.18076	-3.2438	0.001179453	0.009582499
NASP	289.6400679	-0.585332	0.09117	-6.41998	1.36E-10	3.92E-09
ARL4A	203.5527461	-0.585279	0.10215	-5.72962	1.01E-08	2.30E-07
DCUN1D1	41.69092643	-0.585042	0.20847	-2.80631	0.005011259	0.032359969
MARCKSL1	167.7650951	-0.583577	0.11484	-5.08163	3.74E-07	6.81E-06
GALM	57.16921837	-0.583434	0.1768	-3.3	0.000966849	0.008090626
SYNE2	395.4889152	-0.583342	0.07346	-7.94128	2.00E-15	9.15E-14
UBR5	54.95690633	-0.582956	0.1842	-3.16474	0.0015522	0.012212163
IPO7	43.96339849	-0.582309	0.21208	-2.74568	0.006038482	0.037499568
TGFB1	347.9090146	-0.581314	0.07817	-7.4362	1.04E-13	4.14E-12
MT-ND4	3483.228899	-0.581181	0.03912	-14.8557	6.39E-50	1.16E-47
CSRNP1	182.7112809	-0.580839	0.10582	-5.48892	4.04E-08	8.42E-07
DUSP5	264.6702826	-0.580714	0.08808	-6.59297	4.31E-11	1.32E-09
NANS	48.92429627	-0.580197	0.19676	-2.94875	0.003190609	0.022579042
ANTXR2	52.45532123	-0.579629	0.18455	-3.14085	0.001684579	0.013080545
CD226	97.33975297	-0.579534	0.14076	-4.11707	3.84E-05	0.000484754
PGK1	283.0515126	-0.578962	0.08828	-6.55846	5.44E-11	1.63E-09
G3BP1	120.219465	-0.578827	0.12671	-4.56827	4.92E-06	7.48E-05
CFLAR	173.557392	-0.57867	0.1064	-5.43876	5.37E-08	1.10E-06
SAMD4B	39.56963399	-0.578469	0.21988	-2.63082	0.008518021	0.049080682
SESN1	41.60229097	-0.577867	0.21151	-2.73205	0.006294089	0.038727288
HEBP2	110.5235963	-0.576592	0.13109	-4.39829	1.09E-05	0.000155869
CASC4	56.91714164	-0.576255	0.1789	-3.22113	0.001276865	0.010264802
SERINC1	201.9858667	-0.575717	0.10436	-5.51688	3.45E-08	7.30E-07
ARGLU1	421.8798827	-0.575207	0.07604	-7.56415	3.90E-14	1.65E-12
SMC5	86.3192044	-0.573879	0.14595	-3.9319	8.43E-05	0.000994234
RYBP	92.06069398	-0.57366	0.148	-3.87613	0.00010613	0.001213237
ZC3H18	68.10296678	-0.572698	0.1631	-3.5113	0.000445915	0.004181678
FAM53C	124.5226354	-0.570762	0.12927	-4.41544	1.01E-05	0.000144778
EIF4A3	339.7665156	-0.56884	0.08364	-6.8008	1.04E-11	3.36E-10
CD69	1848.415636	-0.568345	0.04906	-11.5847	4.92E-31	4.79E-29
NUDT4	98.06909673	-0.567883	0.14468	-3.92499	8.67E-05	0.001019227
KMT2E-AS1	90.68110282	-0.567773	0.14889	-3.81339	0.000137074	0.001518295
HERC1	88.57846	-0.567629	0.14731	-3.85333	0.000116523	0.001317966
STX16	60.10332756	-0.567494	0.1744	-3.254	0.001137922	0.009308687
REL	661.4793841	-0.566966	0.06535	-8.67631	4.09E-18	2.19E-16
PAN3	60.23373817	-0.566757	0.17294	-3.27718	0.001048497	0.00866326
IFRD1	284.2938122	-0.565589	0.08459	-6.68625	2.29E-11	7.23E-10
KAT6A	87.81949633	-0.563215	0.14507	-3.88226	0.000103491	0.001188147
CNBD2	48.7689965	-0.562861	0.19463	-2.89201	0.003827846	0.026038114
RHOB	187.3405038	-0.562713	0.11774	-4.77918	1.76E-06	2.89E-05
MCUB	98.03353192	-0.562151	0.14003	-4.01437	5.96E-05	0.000727437
OXCT1	65.32060034	-0.561747	0.17275	-3.25187	0.001146471	0.009364302
DUSP4	103.5986791	-0.561488	0.1542	-3.64132	0.000271246	0.002737743
GPATCH8	70.29174341	-0.561366	0.16067	-3.49387	0.000476071	0.004418008
ID2	1059.329482	-0.561178	0.05368	-10.4533	1.41E-25	1.11E-23
SEMA4D	128.3321266	-0.559964	0.12212	-4.5854	4.53E-06	6.96E-05
CUL3	89.7426044	-0.559365	0.14377	-3.89072	9.99E-05	0.001153632
ATF4	194.7650326	-0.559074	0.10289	-5.43362	5.52E-08	1.13E-06
H1FX	254.525606	-0.558767	0.08792	-6.35569	2.07E-10	5.84E-09

PCBP2	259.9956294	-0.558679	0.08801	-6.34804	2.18E-10	6.09E-09
PIK3R1	464.6706608	-0.558578	0.07273	-7.68053	1.58E-14	6.86E-13
CSKMT	74.20259962	-0.556049	0.16272	-3.41716	0.000632788	0.005623614
MAP3K12	49.24248483	-0.555988	0.19392	-2.86711	0.004142423	0.027841663
GCC2	325.9775434	-0.555289	0.08468	-6.55727	5.48E-11	1.64E-09
CHD1	232.0943049	-0.553396	0.09175	-6.03176	1.62E-09	4.01E-08
FAM13B	56.02128448	-0.553133	0.18918	-2.92384	0.00345738	0.024022059
PCGF5	95.46883041	-0.552613	0.14092	-3.92142	8.80E-05	0.001030512
RPS10	2083.334874	-0.552487	0.04082	-13.5358	9.61E-42	1.35E-39
TTTY15	72.97415748	-0.552078	0.16486	-3.3488	0.000811619	0.00698658
CDC42SE2	354.8366974	-0.550645	0.07694	-7.15662	8.27E-13	2.97E-11
RC3H1	51.07946413	-0.549346	0.18768	-2.92707	0.003421704	0.023851615
SF1	278.0912874	-0.549205	0.08675	-6.33057	2.44E-10	6.79E-09
DIP2A	88.73143158	-0.548828	0.15324	-3.58152	0.000341594	0.00333187
TOMM5	70.99507282	-0.548315	0.16582	-3.30672	0.000943956	0.007940507
GNL3	52.56934107	-0.547634	0.1891	-2.89601	0.00377938	0.025790414
MT-CO1	6208.329466	-0.546761	0.03715	-14.7196	4.82E-49	8.33E-47
CAST	296.7539698	-0.545279	0.08299	-6.57063	5.01E-11	1.51E-09
CCDC88B	54.05786687	-0.544943	0.18509	-2.94414	0.003238557	0.022797737
ODF2L	128.2925316	-0.544409	0.12149	-4.48107	7.43E-06	0.000109159
GABPB1-AS1	82.77137016	-0.544301	0.15371	-3.54103	0.000398561	0.003797508
UAP1	76.50107201	-0.544123	0.16091	-3.38159	0.000720686	0.006294163
SLFN11	61.38581105	-0.544049	0.1812	-3.0024	0.002678591	0.019417969
COMMD5	53.29570914	-0.541981	0.19567	-2.76991	0.005607253	0.035313481
TIPARP	195.6222624	-0.541165	0.10704	-5.05561	4.29E-07	7.74E-06
HNRNPH2	61.93691975	-0.540961	0.17856	-3.02963	0.002448528	0.017981643
CD7	387.5812271	-0.540165	0.07687	-7.02722	2.11E-12	7.18E-11
HSD17B11	84.07745983	-0.540074	0.15099	-3.57684	0.000347772	0.003385955
FBXO32	69.40833814	-0.538241	0.16797	-3.2043	0.001353896	0.010802896
ARFGEF1	65.95520257	-0.537658	0.18025	-2.98283	0.002855926	0.020495242
GPS2	55.7379934	-0.534937	0.18134	-2.94986	0.003179217	0.022513318
DCP1A	58.49415658	-0.534561	0.18208	-2.93583	0.003326618	0.023310293
SNHG7	139.6888563	-0.532227	0.1226	-4.34128	1.42E-05	0.000195574
ZCCHC2	46.24409798	-0.530745	0.19735	-2.68942	0.007157632	0.042761953
PPIL4	76.67783271	-0.529383	0.15861	-3.33755	0.000845198	0.00721588
SRRM2	498.5102947	-0.529101	0.07469	-7.08355	1.41E-12	4.90E-11
PRKAA1	56.86184497	-0.529028	0.18593	-2.84535	0.004436214	0.029372425
SUCLG2	67.19645971	-0.527347	0.1659	-3.17876	0.001479062	0.011688303
SRRT	98.98338586	-0.527017	0.13853	-3.80446	0.000142112	0.001561189
HLA-C	3534.549101	-0.526893	0.03785	-13.9223	4.63E-44	7.08E-42
ZRANB1	45.44866497	-0.526276	0.19837	-2.65299	0.007978272	0.046547171
ATXN1	120.5907908	-0.526183	0.12605	-4.17431	2.99E-05	0.000387185
NLRP1	66.08974872	-0.525681	0.16749	-3.1386	0.001697594	0.013172046
NR4A3	179.4510831	-0.524142	0.1068	-4.90755	9.22E-07	1.58E-05
STIM2	81.23094845	-0.523872	0.14963	-3.50106	0.000463408	0.004323239
C16orf54	73.72239051	-0.522617	0.16192	-3.22769	0.001247956	0.010093065
LUZP1	105.0790321	-0.521095	0.13422	-3.88253	0.000103376	0.001188107
SAP30	77.20669362	-0.521048	0.1606	-3.24441	0.00117693	0.009569262
SRSF5	1210.639021	-0.520772	0.05143	-10.1251	4.27E-24	3.15E-22
PSMA3-AS1	117.8993582	-0.515423	0.12622	-4.08342	4.44E-05	0.000554732
RNF114	105.4715785	-0.513893	0.13499	-3.80703	0.000140646	0.001551457
ZC3HAV1	409.871914	-0.513646	0.07423	-6.91931	4.54E-12	1.50E-10

S100A10	1542.175273	-0.512324	0.04508	-11.365	6.25E-30	5.86E-28
TNRC6B	247.2153246	-0.509254	0.09064	-5.61836	1.93E-08	4.24E-07
SNIP1	54.836063	-0.508775	0.18647	-2.7285	0.006362241	0.03903439
OGA	123.9911262	-0.508675	0.12362	-4.11469	3.88E-05	0.000488626
ARID4B	299.3505625	-0.508233	0.08425	-6.03239	1.62E-09	4.00E-08
CHRAC1	82.76399243	-0.507805	0.14787	-3.43421	0.000594288	0.005330156
SAT1	876.5480791	-0.507339	0.06321	-8.02659	1.00E-15	4.62E-14
TXLNG	73.43481508	-0.506913	0.16096	-3.14931	0.001636577	0.012754097
SVIP	128.3901014	-0.506194	0.12363	-4.09457	4.23E-05	0.000531165
NKTR	295.847236	-0.506115	0.08278	-6.11365	9.74E-10	2.48E-08
R3HDM2	92.31869859	-0.505842	0.14624	-3.45889	0.000542411	0.004918473
ZBTB1	278.2428498	-0.505684	0.09473	-5.33809	9.39E-08	1.84E-06
COX20	126.8960026	-0.505351	0.12388	-4.07947	4.51E-05	0.000562262
ATP1A1	119.732904	-0.505269	0.12642	-3.99674	6.42E-05	0.000776878
CD96	226.3089913	-0.504914	0.09761	-5.1729	2.30E-07	4.31E-06
QKI	70.63981983	-0.502991	0.16372	-3.07225	0.002124526	0.01599749
DDX5	2627.489411	-0.502345	0.04265	-11.7787	5.03E-32	4.98E-30
RELA	62.17900527	-0.502285	0.17805	-2.82109	0.004786005	0.031225763
BCLAF1	268.5582547	-0.50181	0.09015	-5.56638	2.60E-08	5.57E-07
WSB1	228.7105421	-0.50141	0.09222	-5.43732	5.41E-08	1.11E-06
SLC39A10	106.5101116	-0.499015	0.13889	-3.593	0.000326897	0.003203112
PLEC	62.39022119	-0.498577	0.17642	-2.82603	0.004712839	0.030861309
NAMPT	207.4346756	-0.495783	0.09707	-5.1073	3.27E-07	5.96E-06
CMTM7	71.64797717	-0.495593	0.16302	-3.04001	0.002365664	0.017505261
MACO1	58.6709907	-0.495587	0.17942	-2.76223	0.005740812	0.036048529
CYP20A1	78.30198244	-0.494768	0.15657	-3.16001	0.001577649	0.012375986
DYRK1A	62.28165879	-0.494598	0.1728	-2.86221	0.004207035	0.02822274
CITED2	276.7211646	-0.49366	0.0869	-5.68068	1.34E-08	3.02E-07
AC087239.1	53.82263152	-0.493538	0.18459	-2.67365	0.00750306	0.044281712
EPS15	112.2424343	-0.49309	0.13193	-3.73755	0.000185822	0.001982352
POLR2J3.1	80.09315802	-0.491787	0.15727	-3.12696	0.001766215	0.013605833
STAU1	111.2577503	-0.491723	0.13376	-3.67627	0.000236673	0.002423348
SAFB2	95.13023694	-0.491035	0.14223	-3.4524	0.000555631	0.005021169
PHF1	116.3486858	-0.490838	0.13111	-3.74384	0.000181227	0.001939125
GLS	235.437127	-0.488603	0.09124	-5.35523	8.54E-08	1.69E-06
FAM133B	345.066291	-0.487886	0.08532	-5.71816	1.08E-08	2.45E-07
XPC	60.1928625	-0.485727	0.17958	-2.70473	0.006836061	0.041278698
RPS6KA3	91.56961712	-0.485571	0.14573	-3.33202	0.000862174	0.007321639
TMEM123	122.008624	-0.485134	0.12497	-3.88189	0.000103649	0.001188686
ACOT13	63.15544694	-0.484921	0.17247	-2.81165	0.004928736	0.031942745
DYNLT3	97.29983449	-0.484665	0.14006	-3.46052	0.000539131	0.004897031
SOCS3	135.1801983	-0.484353	0.1235	-3.92196	8.78E-05	0.001029348
SERINC3	111.9135744	-0.484074	0.12967	-3.73321	0.000189058	0.002010853
SRSF7	1655.79446	-0.483715	0.05083	-9.51678	1.79E-21	1.20E-19
JUN	3750.112265	-0.483607	0.04645	-10.4105	2.22E-25	1.71E-23
TMEM107	80.73773959	-0.482023	0.15798	-3.05109	0.002280137	0.016919186
SLC38A1	274.4913767	-0.481753	0.08591	-5.60751	2.05E-08	4.46E-07
IRF2BP2	116.7804053	-0.481453	0.12814	-3.75734	0.000171728	0.001859807
SUPT5H	96.04083541	-0.480184	0.13737	-3.49556	0.000473062	0.004397707
MYLIP	177.8206084	-0.479902	0.10365	-4.62982	3.66E-06	5.74E-05
DHRS3	71.02084698	-0.478903	0.15937	-3.00493	0.002656383	0.019270032
MAPK1IP1L	117.1792823	-0.477443	0.13026	-3.66528	0.000247071	0.002512988

APLP2	75.39486116	-0.477211	0.15989	-2.98457	0.002839796	0.020393168
TCF7	120.526713	-0.476645	0.12862	-3.70597	0.000210579	0.002189699
PABPN1	100.9241366	-0.476117	0.13853	-3.43704	0.000588107	0.00528358
ORAI1	126.0610041	-0.475726	0.12853	-3.70119	0.000214587	0.002224881
SMARCA5	209.3233997	-0.474633	0.10482	-4.52794	5.96E-06	8.93E-05
ZNF292	138.4480006	-0.47453	0.12045	-3.93954	8.16E-05	0.000965242
C6orf62	202.1621555	-0.474273	0.09823	-4.82832	1.38E-06	2.29E-05
PCM1	142.7052689	-0.474044	0.11706	-4.04956	5.13E-05	0.000633627
SERTAD1	543.2363041	-0.469285	0.06628	-7.08041	1.44E-12	4.99E-11
EIF1	6399.911412	-0.469226	0.03973	-11.809	3.51E-32	3.57E-30
PPP1R15A	1071.60563	-0.4665	0.06356	-7.33913	2.15E-13	8.43E-12
SDCBP	237.223876	-0.465887	0.09523	-4.89238	9.96E-07	1.70E-05
MT-CYB	5504.926053	-0.464653	0.03828	-12.1387	6.59E-34	7.12E-32
HMGCS1	93.59497166	-0.464394	0.14331	-3.24053	0.001193083	0.009685881
RORA	260.8761969	-0.464022	0.08829	-5.25586	1.47E-07	2.84E-06
PKN2	86.47921094	-0.463426	0.15352	-3.01873	0.002538394	0.018577846
DDX18	260.7259936	-0.462553	0.09739	-4.74945	2.04E-06	3.32E-05
GLUD1	67.57761403	-0.462453	0.17253	-2.68036	0.007354388	0.043645011
ESYT2	67.70101762	-0.462139	0.17196	-2.6874	0.007200958	0.042935317
CMTM6	101.0851873	-0.459085	0.13891	-3.30494	0.000949975	0.007978599
MT-ND3	4989.107375	-0.458117	0.03958	-11.5747	5.54E-31	5.34E-29
N4BP2L2	416.6791623	-0.458034	0.07192	-6.36892	1.90E-10	5.40E-09
ANKRD36C	86.54869208	-0.457653	0.14901	-3.0712	0.002131994	0.016031154
MAP3K2	134.0513893	-0.456647	0.12596	-3.62529	0.000288639	0.002890214
PNISR	259.9218158	-0.454783	0.09015	-5.04494	4.54E-07	8.13E-06
ZNF331	922.4259278	-0.454037	0.06285	-7.22371	5.06E-13	1.87E-11
HSPA5	543.4835699	-0.453802	0.06618	-6.85685	7.04E-12	2.30E-10
RAB21	148.1643976	-0.452319	0.11377	-3.97576	7.02E-05	0.000838723
USP15	188.3133134	-0.452101	0.1021	-4.42801	9.51E-06	0.000137517
KMT2E	366.2293909	-0.449527	0.079	-5.6899	1.27E-08	2.87E-07
DYNC1H1	199.8886683	-0.449284	0.0987	-4.55207	5.31E-06	8.01E-05
DBF4	113.773986	-0.449214	0.1339	-3.35481	0.0007942	0.006847659
HNRNPL	97.57048291	-0.449143	0.13826	-3.24858	0.001159839	0.009459055
PHF3	139.1176563	-0.448656	0.12401	-3.61791	0.000296989	0.002967118
DOCK10	127.8484533	-0.448531	0.12211	-3.67329	0.000239452	0.002444782
MSL2	67.9639894	-0.44845	0.16708	-2.68397	0.007275399	0.043272244
FNBP1	263.7563991	-0.44818	0.08678	-5.16483	2.41E-07	4.48E-06
IER5	470.9892976	-0.447719	0.06819	-6.56556	5.18E-11	1.56E-09
TRIM22	154.8902324	-0.446632	0.11553	-3.86585	0.000110705	0.001260152
PPP1R10	270.4387823	-0.446168	0.09158	-4.87191	1.11E-06	1.87E-05
PTBP1	109.9179537	-0.445696	0.13427	-3.31928	0.000902492	0.007627694
ITLN1	72.92979844	-0.444852	0.16554	-2.68732	0.0072027	0.042935317
F2R	71.4434088	-0.444812	0.16353	-2.72006	0.006527082	0.039704251
ASH1L	141.6937131	-0.444115	0.1231	-3.60776	0.000308855	0.003051474
G3BP2	312.8684578	-0.442352	0.08403	-5.2642	1.41E-07	2.72E-06
DDHD1	72.91986532	-0.442314	0.16137	-2.74105	0.006124349	0.037926234
ARPP19	109.398244	-0.441737	0.13309	-3.31899	0.000903429	0.007629593
AFF4	88.53266803	-0.441003	0.15627	-2.82212	0.004770798	0.031145537
BBX	147.3897051	-0.440968	0.11457	-3.84897	0.000118616	0.001338807
JUNB	4889.534171	-0.437805	0.0355	-12.3321	6.09E-35	6.85E-33
IDI1	392.4306182	-0.437695	0.07857	-5.57104	2.53E-08	5.43E-07
SMU1	96.46805065	-0.436925	0.13709	-3.18717	0.001436702	0.011387195

NFU1	68.71424565	-0.435985	0.1617	-2.69627	0.007012166	0.042010177
ZNF655	114.7067764	-0.435158	0.14176	-3.06969	0.002142844	0.016078841
RNMT	224.7409058	-0.43464	0.09621	-4.51745	6.26E-06	9.31E-05
TOR1AIP2	69.26156712	-0.434374	0.16316	-2.66219	0.007763479	0.045598896
WNK1	172.3984768	-0.43357	0.10441	-4.1526	3.29E-05	0.000421729
NUP98	76.3585393	-0.432994	0.15836	-2.73421	0.006253069	0.038541382
VPS37B	154.7922534	-0.432543	0.11459	-3.7747	0.000160203	0.001747374
RICTOR	77.68232015	-0.432325	0.15174	-2.84913	0.004383885	0.029189527
PRPF4B	214.0455797	-0.432215	0.09572	-4.5155	6.32E-06	9.39E-05
INTS6	412.535531	-0.431987	0.07741	-5.58067	2.40E-08	5.16E-07
ANKRD12	500.1953328	-0.431338	0.07164	-6.02064	1.74E-09	4.28E-08
DNAJC2	71.68250696	-0.431066	0.15911	-2.70919	0.006744791	0.040819722
RSBN1	111.0485715	-0.429382	0.13932	-3.08191	0.002056777	0.015553014
ATP1B1	82.82741253	-0.429358	0.14909	-2.8798	0.003979214	0.026930796
CCNL1	852.4630301	-0.428896	0.05572	-7.69679	1.40E-14	6.07E-13
ANKRD11	219.5150684	-0.428591	0.10178	-4.21089	2.54E-05	0.000336428
RAP2B	91.28151801	-0.428382	0.14282	-2.99942	0.002704904	0.019595444
IQGAP1	314.3750207	-0.42792	0.08307	-5.15104	2.59E-07	4.79E-06
NSMCE3	160.3539702	-0.427138	0.11023	-3.8748	0.000106711	0.001218575
SLC30A1	1011.662376	-0.425222	0.05649	-7.52729	5.18E-14	2.16E-12
PTGER2	91.35308216	-0.424685	0.15558	-2.72964	0.006340434	0.038922915
GPRIN3	116.7072441	-0.424307	0.12677	-3.34708	0.000816686	0.007005369
SRSF10	190.1932574	-0.424305	0.10745	-3.94872	7.86E-05	0.000930997
CLK1	311.1188716	-0.423176	0.08387	-5.04587	4.51E-07	8.10E-06
SNRNP200	109.8803299	-0.423053	0.13336	-3.17234	0.001512154	0.011923694
PRRC2C	494.1778662	-0.421723	0.06965	-6.05462	1.41E-09	3.53E-08
PIP4K2A	215.3103053	-0.420212	0.09696	-4.3339	1.46E-05	0.000201473
EPM2AIP1	92.82675412	-0.419326	0.14341	-2.92398	0.003455826	0.024022059
CSNK1G2	81.90195104	-0.419323	0.1481	-2.83129	0.004636006	0.030507544
RPS21	1871.336149	-0.41831	0.04105	-10.1891	2.22E-24	1.65E-22
COPA	93.09587262	-0.416197	0.14359	-2.8985	0.003749492	0.025668304
ARID1B	129.3744053	-0.415865	0.12374	-3.36081	0.000777152	0.006727769
JAML	96.27542917	-0.415493	0.14866	-2.79501	0.005189885	0.033248218
GADD45A	132.8713365	-0.414898	0.12862	-3.22579	0.001256264	0.010129631
WBP11	119.4229548	-0.414666	0.12742	-3.25422	0.001137059	0.009308687
NXF1	99.02679038	-0.414394	0.13653	-3.03521	0.002403685	0.017725315
TOP1	277.0023933	-0.41429	0.08976	-4.6153	3.93E-06	6.11E-05
CYCS	554.0805675	-0.41357	0.06328	-6.53592	6.32E-11	1.87E-09
SPAG9	94.73377176	-0.413004	0.14516	-2.84518	0.004438644	0.029372425
STK17B	496.2791646	-0.412097	0.06773	-6.0846	1.17E-09	2.95E-08
PTP4A1	170.292965	-0.411683	0.11432	-3.601	0.000316991	0.003117462
TMEM243	92.04457483	-0.41114	0.14115	-2.91286	0.003581327	0.024706768
IRF1	614.1583097	-0.411045	0.0662	-6.20885	5.34E-10	1.43E-08
NOP56	159.7011718	-0.410791	0.11391	-3.6062	0.000310711	0.003066984
ZBTB20	139.1537536	-0.409423	0.11805	-3.46809	0.000524176	0.004777415
ISCA1	225.4221637	-0.408951	0.10089	-4.05342	5.05E-05	0.000624373
UGP2	142.2408429	-0.408344	0.1142	-3.57565	0.000349362	0.003397985
PRKCB	92.02026111	-0.407167	0.14552	-2.79801	0.005141775	0.032983808
STAG2	129.883366	-0.406215	0.1204	-3.37393	0.000741042	0.006435999
MT-CO3	5273.556372	-0.403954	0.04009	-10.0753	7.10E-24	5.17E-22
RBM17	112.8401585	-0.401212	0.1362	-2.94575	0.003221744	0.022731272
SRPRA	105.8103062	-0.400878	0.13692	-2.92778	0.003413874	0.023828082

SFPQ	376.7136715	-0.400683	0.07962	-5.03248	4.84E-07	8.61E-06
BZW1	475.1506564	-0.400634	0.06798	-5.89298	3.79E-09	9.00E-08
MORF4L1	336.6384188	-0.398903	0.0781	-5.10788	3.26E-07	5.96E-06
TOB2	88.71047812	-0.398571	0.151	-2.63956	0.00830133	0.0480131
GTF2B	324.053074	-0.395689	0.08566	-4.61915	3.85E-06	6.02E-05
CHD2	165.8854497	-0.394394	0.11223	-3.51429	0.000440933	0.004145858
EIF2S3	94.76121561	-0.394285	0.13813	-2.8545	0.0043105	0.028780606
RBMX	191.8733867	-0.393268	0.10199	-3.85606	0.000115231	0.001304734
RPS17	222.9986338	-0.392754	0.09435	-4.16293	3.14E-05	0.000404549
BAZ1A	201.2466405	-0.392026	0.09823	-3.99099	6.58E-05	0.000794621
RAB5A	97.48769145	-0.391696	0.13642	-2.87115	0.004089805	0.027539906
PITPNC1	114.0189033	-0.391655	0.1295	-3.02437	0.002491545	0.018272465
SNRK	90.19494606	-0.391545	0.14304	-2.73731	0.006194378	0.038278729
ATRX	267.6560559	-0.389678	0.09411	-4.14056	3.46E-05	0.000442385
YPEL5	1029.863911	-0.387391	0.06127	-6.32231	2.58E-10	7.12E-09
AL118516.1	135.674756	-0.386807	0.12161	-3.18074	0.001468978	0.011617193
WTAP	187.5251233	-0.381818	0.10827	-3.52657	0.000420981	0.003989816
YWHAZ	913.8664345	-0.381636	0.05975	-6.38668	1.70E-10	4.84E-09
RPL36A	552.2792832	-0.377412	0.06526	-5.78288	7.34E-09	1.69E-07
EMD	116.4762543	-0.376513	0.1261	-2.98573	0.002829046	0.020329613
GATA3	93.06200831	-0.375415	0.13965	-2.68833	0.007181011	0.04287769
CD46	143.6092654	-0.374125	0.11793	-3.17233	0.001512192	0.011923694
RNF149	94.66490195	-0.371692	0.1407	-2.64182	0.008246078	0.047780181
CRIP1	561.712791	-0.371355	0.07058	-5.26141	1.43E-07	2.76E-06
PRKAR1A	105.5950318	-0.370443	0.13133	-2.82063	0.004792902	0.031251706
GPBP1	545.2290152	-0.369746	0.06722	-5.50084	3.78E-08	7.93E-07
POLR2K	172.4531639	-0.368476	0.10596	-3.47754	0.000506039	0.004643195
MT-ND1	3054.239815	-0.367728	0.04313	-8.52612	1.51E-17	7.67E-16
EIF4A1	305.0892832	-0.367209	0.08433	-4.35446	1.33E-05	0.000185123
PRNP	113.5722795	-0.367123	0.13243	-2.77222	0.005567606	0.035146541
KCNQ1OT1	163.45849	-0.365969	0.11929	-3.06794	0.002155422	0.016159246
BDP1	132.5219584	-0.36336	0.11947	-3.04153	0.002353794	0.017429477
MBNL1	496.8953024	-0.36333	0.06738	-5.39205	6.97E-08	1.40E-06
TTC19	107.9870411	-0.360296	0.13365	-2.6958	0.007021898	0.042044941
PITHD1	161.5279159	-0.359561	0.11079	-3.24542	0.001172764	0.009542641
EIF1AX	239.2501111	-0.357565	0.10436	-3.42611	0.000612302	0.005468812
KDM2A	121.7487119	-0.355868	0.12444	-2.85986	0.004238219	0.028378562
BIRC3	202.506666	-0.35422	0.10634	-3.33093	0.000865556	0.007344529
RPS16	3109.643944	-0.353228	0.03774	-9.35986	7.98E-21	5.09E-19
MECP2	113.4887806	-0.351956	0.13042	-2.69869	0.006961202	0.041845431
NUFIP2	195.3786899	-0.350264	0.10641	-3.2917	0.000995835	0.008305096
SNHG12	162.0865532	-0.350217	0.11688	-2.99649	0.00273109	0.019758389
HNRNPA2B1	1268.202523	-0.344395	0.05593	-6.15742	7.39E-10	1.93E-08
NCL	741.0070113	-0.342995	0.06108	-5.61548	1.96E-08	4.29E-07
MORF4L2	177.0560727	-0.341436	0.10582	-3.22651	0.001253081	0.010113531
C16orf72	143.1618293	-0.341349	0.11331	-3.0126	0.002590188	0.018892307
THUMPD3-AS	217.8377147	-0.340173	0.10208	-3.33253	0.000860617	0.007314218
MAPRE1	136.0776376	-0.339692	0.12012	-2.82784	0.004686393	0.030782324
MT-CO2	6852.732022	-0.339279	0.04112	-8.25145	1.56E-16	7.54E-15
PTMA	5250.022097	-0.336952	0.03734	-9.02309	1.83E-19	1.08E-17
ARL6IP1	195.2320209	-0.336051	0.10555	-3.18374	0.001453878	0.011514802
BTG3	133.2010091	-0.333176	0.12263	-2.71682	0.006591239	0.040048982

CLEC2D	305.0531809	-0.331652	0.08386	-3.9547	7.66E-05	0.000910061
JMJD6	248.8669793	-0.32951	0.08948	-3.68254	0.000230926	0.002368806
HCG18	127.3064834	-0.327725	0.1232	-2.66021	0.007809236	0.045785657
SKP1	725.1567755	-0.327144	0.06062	-5.39652	6.79E-08	1.36E-06
EVI2B	179.3742662	-0.321622	0.10861	-2.96131	0.003063312	0.021808013
TTC3	127.6624454	-0.321046	0.12134	-2.64578	0.008150398	0.047350846
SAMD9	148.1246463	-0.32085	0.11748	-2.73106	0.006313092	0.03879959
YME1L1	385.2860436	-0.319359	0.08012	-3.98615	6.72E-05	0.000806497
LUC7L3	156.6326711	-0.318811	0.11597	-2.74901	0.005977484	0.037185511
NEAT1	588.5642573	-0.318744	0.07147	-4.45997	8.20E-06	0.000119823
AMD1	358.803422	-0.318329	0.08139	-3.91133	9.18E-05	0.001068712
DHX36	218.6535287	-0.31315	0.09446	-3.31514	0.000915987	0.007717374
PNRC1	1357.491116	-0.310597	0.04754	-6.5329	6.45E-11	1.91E-09
PPP1R2	311.9397736	-0.307948	0.08204	-3.75365	0.000174281	0.001881747
NIPBL	136.2503163	-0.307782	0.11731	-2.62377	0.008696339	0.049758493
ATM	170.6156894	-0.307104	0.10462	-2.9353	0.003332281	0.023319428
ANKRD44	161.5497626	-0.306056	0.1154	-2.65218	0.007997322	0.046632884
HNRNPU	571.2980322	-0.305479	0.06522	-4.68349	2.82E-06	4.53E-05
H2AFX	302.8300944	-0.304423	0.08741	-3.48271	0.000496359	0.004578483
RBM25	296.92511	-0.304134	0.08486	-3.58389	0.000338517	0.003304864
RPLP0	1215.228952	-0.303375	0.05122	-5.92253	3.17E-09	7.59E-08
CALM2	776.3988149	-0.3033	0.05935	-5.11004	3.22E-07	5.90E-06
RBBP6	205.4653031	-0.303207	0.1036	-2.92658	0.00342709	0.023873611
GPR65	216.0273278	-0.302626	0.09855	-3.07067	0.002135756	0.016048166
H2AFJ	186.9114779	-0.302015	0.10363	-2.91439	0.003563839	0.024617865
VMP1	170.5592747	-0.301717	0.10947	-2.75625	0.0058469	0.036535133
CD8A	417.2698593	-0.301636	0.07562	-3.98883	6.64E-05	0.000800078
BOD1L1	164.647095	-0.300905	0.11415	-2.63604	0.008387862	0.048487372
YTHDC1	190.8723061	-0.300127	0.10033	-2.99129	0.002778029	0.020030266
RBL2	154.4152133	-0.299569	0.11215	-2.67126	0.007556627	0.044548713
UBE2A	226.0404618	-0.298992	0.09545	-3.13258	0.0017328	0.013391093
CDV3	288.8461909	-0.298283	0.08308	-3.59036	0.000330216	0.003229719
WAC	181.6620292	-0.296314	0.10344	-2.86464	0.004174813	0.028041742
TAF1D	194.5934933	-0.295932	0.10268	-2.88208	0.003950636	0.026771249
CHMP1B	267.9029265	-0.295843	0.09681	-3.05605	0.002242757	0.016664928
PTPRC	1252.642399	-0.29527	0.04681	-6.3082	2.82E-10	7.76E-09
ARAP2	184.9804111	-0.292638	0.10139	-2.88638	0.003896981	0.026458234
SCAND1	209.0153414	-0.292505	0.09877	-2.96149	0.003061541	0.021808013
HNRNPC	492.1231445	-0.292358	0.06697	-4.36573	1.27E-05	0.00017791
DDX21	236.7031034	-0.291034	0.09254	-3.14489	0.0016615	0.012920091
GNAS	743.8330885	-0.289749	0.05931	-4.88556	1.03E-06	1.75E-05
PAPOLA	203.2073578	-0.289716	0.10087	-2.87215	0.00407685	0.027487268
NOP58	172.313345	-0.285519	0.10524	-2.71311	0.006665517	0.040454359
RPL36	2738.390292	-0.284964	0.04074	-6.99402	2.67E-12	9.02E-11
RPS27	10452.08513	-0.284651	0.03284	-8.66856	4.38E-18	2.33E-16
EIF4G2	413.338572	-0.284484	0.07232	-3.93378	8.36E-05	0.000987556
UBE2S	819.6491999	-0.284345	0.06519	-4.36201	1.29E-05	0.000180022
BRD2	374.2850216	-0.283223	0.07778	-3.64156	0.000270996	0.002737743
ZEB2	192.5414146	-0.282121	0.10603	-2.66086	0.00779405	0.045721674
CYLD	187.8470745	-0.281862	0.10408	-2.7082	0.006765015	0.040918969
EIF3A	185.1838772	-0.281505	0.10435	-2.69766	0.006982932	0.041910992
HSP90B1	412.8509529	-0.27757	0.07981	-3.47783	0.000505482	0.004643195

MT-ATP6	9548.347748	-0.276911	0.03401	-8.14117	3.91E-16	1.86E-14
TXN	431.3125416	-0.276246	0.0714	-3.86925	0.000109171	0.001245338
ARF6	298.9845222	-0.276188	0.08364	-3.30228	0.00095903	0.008035722
SPTAN1	205.9719926	-0.273759	0.10028	-2.72992	0.006335034	0.038912094
NR4A2	872.3607262	-0.273111	0.05778	-4.72681	2.28E-06	3.69E-05
EIF3E	289.9816142	-0.270919	0.08299	-3.26453	0.001096456	0.009007633
IFITM2	840.4879983	-0.270764	0.06085	-4.45005	8.59E-06	0.000124982
BPTF	213.4280507	-0.268774	0.10136	-2.65178	0.008006779	0.046662601
ZFP36L1	393.7952552	-0.265027	0.08449	-3.13692	0.001707327	0.013228384
KTN1	214.4752484	-0.26445	0.09611	-2.75139	0.005934232	0.037002498
GADD45B	953.4629572	-0.264003	0.09978	-2.64573	0.008151426	0.047350846
CSDE1	218.9475504	-0.262573	0.10006	-2.62416	0.00868634	0.049729178
RBM8A	432.4013509	-0.260753	0.06997	-3.72642	0.000194219	0.002053499
LDHA	423.9071284	-0.258621	0.07456	-3.4688	0.000522794	0.004768881
HNRNPAB	281.7773881	-0.257884	0.08968	-2.87569	0.00403145	0.027215465
TMEM50A	287.0095513	-0.257687	0.08431	-3.0563	0.002240883	0.01666258
CNBP	549.910115	-0.257333	0.07071	-3.63947	0.000273196	0.002749946
SBDS	346.3211072	-0.257178	0.07821	-3.28847	0.001007337	0.008374905
NPM1	1098.860829	-0.256744	0.05198	-4.93949	7.83E-07	1.35E-05
HNRNPH3	223.250082	-0.256709	0.09573	-2.68172	0.007324405	0.043509411
COX7B	322.3381128	-0.256319	0.08441	-3.03668	0.002392001	0.0176635
CCNI	477.0429711	-0.253788	0.06876	-3.69109	0.000223298	0.002301815
SF3B1	367.309695	-0.253162	0.07615	-3.32463	0.000885371	0.007494832
RSL24D1	271.0155069	-0.251122	0.09105	-2.75817	0.005812651	0.036411519
EEF2	435.0255021	-0.250539	0.07143	-3.50729	0.000452698	0.004237851
RPL38	2075.114916	-0.246968	0.03978	-6.20886	5.34E-10	1.43E-08
CIRBP	504.8091096	-0.241372	0.06784	-3.55779	0.000373985	0.003598596
PMAIP1	462.8800374	-0.240979	0.06909	-3.48797	0.000486697	0.004504895
STK4	486.766333	-0.239778	0.06589	-3.63893	0.000273772	0.002753159
PPIG	257.809584	-0.239165	0.09096	-2.62939	0.008553772	0.049222361
ITM2A	297.9245418	-0.238878	0.08454	-2.82571	0.004717557	0.030873309
EIF4A2	740.388169	-0.237453	0.0577	-4.11552	3.86E-05	0.000487438
HSPA8	1669.046968	-0.235166	0.04558	-5.15982	2.47E-07	4.58E-06
LYAR	254.657538	-0.232529	0.08761	-2.6541	0.007951938	0.046444178
DDX17	289.2024117	-0.227444	0.08603	-2.6438	0.008198106	0.047544574
CALM1	2370.287111	-0.225278	0.03977	-5.66481	1.47E-08	3.29E-07
EIF5	790.1012348	-0.224686	0.05854	-3.8382	0.000123938	0.001390081
RPL39	4759.037203	-0.217757	0.03587	-6.07114	1.27E-09	3.21E-08
RPL34	5006.501491	-0.217101	0.03737	-5.80879	6.29E-09	1.46E-07
RPS20	1513.663292	-0.213846	0.04666	-4.58336	4.58E-06	7.01E-05
SQSTM1	451.4624481	-0.212653	0.07609	-2.7946	0.005196333	0.033254042
IER2	2174.04982	-0.212545	0.04132	-5.144	2.69E-07	4.95E-06
RPS29	5896.081177	-0.210692	0.03556	-5.92543	3.11E-09	7.51E-08
CD53	410.7861392	-0.20923	0.07205	-2.90413	0.003682776	0.025308735
RPL30	6620.157297	-0.208692	0.03313	-6.29869	3.00E-10	8.19E-09
RPL41	9143.288208	-0.206973	0.03394	-6.0979	1.07E-09	2.72E-08
RPS25	3582.628964	-0.197859	0.03786	-5.22542	1.74E-07	3.30E-06
H3F3B	5483.664938	-0.195922	0.03492	-5.61102	2.01E-08	4.38E-07
RPL36AL	1001.397927	-0.195023	0.05593	-3.487	0.000488468	0.004517201
ATP5IF1	412.9823486	-0.188663	0.07061	-2.67182	0.007543998	0.044498776
TUBA1B	532.2735526	-0.187486	0.06634	-2.8262	0.00471038	0.030861309
RPL32	5360.597595	-0.186375	0.03425	-5.44118	5.29E-08	1.09E-06

UBE2D3	554.2983336	-0.180399	0.06579	-2.74199	0.006106852	0.037858235
RPL35A	3201.253852	-0.179549	0.03947	-4.5493	5.38E-06	8.10E-05
S100A6	2152.70059	-0.179069	0.04241	-4.22238	2.42E-05	0.000320514
RPL5	2206.833167	-0.173013	0.04157	-4.1619	3.16E-05	0.000405886
RPL37	3496.770318	-0.172429	0.03895	-4.42747	9.53E-06	0.000137652
RPS28	4252.986733	-0.169356	0.03413	-4.96154	6.99E-07	1.21E-05
ARHGDIB	882.7665125	-0.167494	0.05407	-3.09773	0.001950086	0.014840623
TPT1	7978.497143	-0.164579	0.03137	-5.24711	1.55E-07	2.96E-06
HSPH1	1096.762276	-0.162023	0.05515	-2.93805	0.003302802	0.023185665
RPS13	2816.738125	-0.161075	0.03738	-4.30857	1.64E-05	0.000224542
ITM2B	868.5973997	-0.160937	0.05336	-3.01613	0.002560254	0.018721803
RPLP1	8131.182214	-0.16021	0.03517	-4.55581	5.22E-06	7.90E-05
RPS3	5522.657449	-0.156372	0.03536	-4.42279	9.74E-06	0.000140128
RPL31	1046.055892	-0.155954	0.05022	-3.10571	0.001898212	0.01451813
RPL14	3410.303113	-0.150588	0.03607	-4.17541	2.97E-05	0.000386721
RPS19	6674.515992	-0.15004	0.03761	-3.98893	6.64E-05	0.000800078
RPL11	4800.116694	-0.14912	0.03322	-4.48849	7.17E-06	0.000105717
RPS6	3756.518216	-0.146521	0.03507	-4.17834	2.94E-05	0.000382698
DNAJA1	2457.322441	-0.145379	0.05053	-2.87712	0.004013237	0.027126741
RPS24	4855.922791	-0.141679	0.03399	-4.16765	3.08E-05	0.000397224
RPL22	1517.668616	-0.140084	0.04442	-3.15365	0.001612436	0.012584295
RPL9	3683.230114	-0.133795	0.03848	-3.47734	0.000506412	0.004643195
EEF1A1	6473.753895	-0.133461	0.03238	-4.12227	3.75E-05	0.00047449
RPS23	4795.205121	-0.131587	0.03704	-3.5526	0.000381446	0.003657229
DUSP2	2253.071518	-0.121696	0.04334	-2.80821	0.004981823	0.032215334
RPS15A	5260.064566	-0.120937	0.03478	-3.47761	0.000505905	0.004643195
RPS8	4491.815033	-0.114987	0.037	-3.10812	0.001882786	0.014420764
S100A4	3492.191479	-0.114674	0.04093	-2.80153	0.005086047	0.032744105
RPL37A	2590.224941	-0.112486	0.04113	-2.73503	0.00623752	0.038489889
RPS18	7801.449131	-0.098688	0.03394	-2.90766	0.003641419	0.02505671
RPS14	5951.222485	-0.095313	0.03235	-2.94661	0.00321282	0.022721197
RPS27A	6639.557848	-0.092864	0.03223	-2.88155	0.003957262	0.026799179
FTL	2611.582707	0.115872	0.04295	2.697613	0.00698386	0.041910992
HLA-A	3908.620176	0.117002	0.04163	2.810593	0.004945036	0.032028989
RPS3A	4308.876406	0.144138	0.03607	3.996611	6.43E-05	0.000776878
EEF1D	1129.207039	0.154118	0.05029	3.064774	0.002178345	0.016299504
COX4I1	812.5439548	0.157601	0.05602	2.81344	0.00490146	0.0318238
ATP5F1E	1728.085687	0.169409	0.04673	3.625188	0.000288751	0.002890214
HSPD1	1483.44104	0.171138	0.05602	3.055091	0.00224992	0.016706555
SERF2	1417.84619	0.172309	0.04514	3.817371	0.000134881	0.001498679
GZMM	550.7044453	0.182772	0.0653	2.798949	0.005126929	0.032928059
UBL5	487.7345311	0.191593	0.07301	2.624371	0.008680907	0.049724681
CD52	1312.367027	0.192607	0.05469	3.521709	0.000428774	0.004049325
ARL6IP5	411.3322471	0.195922	0.07396	2.64901	0.008072786	0.046996089
PPIA	1386.071908	0.196783	0.0453	4.344367	1.40E-05	0.000193095
CYBA	1440.625363	0.203309	0.05119	3.971506	7.14E-05	0.000851941
EIF3K	483.5272794	0.203431	0.06802	2.990924	0.00278135	0.020040704
S100A11	612.6889591	0.207272	0.06197	3.344732	0.000823621	0.007050193
SLC25A6	440.3179103	0.216373	0.07288	2.968827	0.002989388	0.021364852
EDF1	465.8186285	0.219565	0.07095	3.094426	0.001971941	0.01498563
POMP	309.5595479	0.223996	0.08361	2.679095	0.007382156	0.04376064
MYL12B	892.745917	0.228093	0.05365	4.251867	2.12E-05	0.000282485

LSP1	346.9337492	0.230272	0.07887	2.91972	0.003503463	0.024279177
HSP90AB1	4225.830505	0.232204	0.05133	4.523649	6.08E-06	9.10E-05
GUK1	450.2059687	0.239504	0.07345	3.260688	0.001111423	0.009112817
AHSA1	289.6214125	0.241082	0.08989	2.682114	0.007315863	0.043488741
CAPZB	288.8860105	0.24179	0.08498	2.845168	0.004438805	0.029372425
PTGES3	575.42205	0.243993	0.06827	3.573955	0.000351629	0.003414188
DDIT3	701.5565241	0.244987	0.06604	3.709937	0.000207311	0.002164024
ARPC2	792.6515211	0.246663	0.05881	4.194297	2.74E-05	0.000359361
DBI	326.0884937	0.248411	0.08182	3.035953	0.002397771	0.017693894
CLEC2B	665.2733906	0.249843	0.06717	3.719376	0.000199715	0.002101232
ACTB	4095.062643	0.249926	0.0366	6.827813	8.62E-12	2.80E-10
ERP29	258.293235	0.249967	0.09454	2.643913	0.008195384	0.047544574
MYL12A	1675.6619	0.256708	0.04432	5.792221	6.95E-09	1.61E-07
PSMD8	212.3380882	0.261619	0.09505	2.752544	0.005913424	0.036894248
LDHB	239.8208779	0.265431	0.09432	2.814078	0.004891734	0.031779933
C9orf16	243.0541171	0.266821	0.09044	2.950162	0.003176077	0.022505976
ATP5MC2	548.2191816	0.268722	0.06566	4.09264	4.26E-05	0.000534361
CIB1	337.6004013	0.269464	0.07893	3.414169	0.000639769	0.005676225
PTMS	241.9219359	0.274548	0.09404	2.919502	0.003505909	0.024280403
LGALS1	1432.165727	0.275426	0.05072	5.429872	5.64E-08	1.15E-06
COPS9	190.0875432	0.276899	0.10259	2.699101	0.00695271	0.041817872
MYL6	1458.934365	0.281249	0.04659	6.036989	1.57E-09	3.91E-08
SEM1	199.4169726	0.28459	0.10345	2.751097	0.005939612	0.037014472
SH3BGRL3	2266.457769	0.285441	0.04059	7.033068	2.02E-12	6.91E-11
ATP5MPL	201.0483047	0.286415	0.10013	2.860337	0.004231908	0.028354046
PSME1	474.5893978	0.289462	0.0698	4.147044	3.37E-05	0.000431066
UBXN1	229.9977222	0.290479	0.09462	3.070106	0.002139827	0.016067476
CD3G	565.8721866	0.290973	0.06676	4.358686	1.31E-05	0.000182298
CFL1	1781.930782	0.293854	0.04678	6.280991	3.36E-10	9.11E-09
PSMB6	149.0674822	0.295753	0.11221	2.635659	0.008397407	0.04851504
DYNLRB1	230.613028	0.296131	0.09207	3.216491	0.001297686	0.010408728
TMSB4X	13103.11951	0.296521	0.03401	8.71801	2.83E-18	1.55E-16
BCAS2	428.7591075	0.302912	0.07402	4.092262	4.27E-05	0.000534607
RBX1	267.8251125	0.307024	0.09078	3.382181	0.000719128	0.006286495
LAMTOR5	153.5172286	0.314778	0.11284	2.789667	0.005276224	0.033604521
TBCA	243.9557871	0.316364	0.09008	3.512067	0.000444635	0.004173329
OCIAD1	150.7437712	0.317333	0.116	2.735672	0.0062253	0.038436646
UBE2L3	179.0184044	0.317761	0.10522	3.019896	0.002528616	0.018531641
MGP	264.3826497	0.318657	0.09287	3.431036	0.00060128	0.005383849
C4orf3	254.823699	0.318874	0.09315	3.423235	0.000618805	0.005522281
GNG5	238.2354706	0.318972	0.09698	3.288918	0.001005732	0.008368067
ATP6V0E1	352.151306	0.321192	0.07748	4.145711	3.39E-05	0.000433063
DYNLL1	676.3700015	0.323633	0.06049	5.350562	8.77E-08	1.72E-06
IL32	3133.697946	0.323724	0.04102	7.891036	3.00E-15	1.36E-13
CMC1	249.4248984	0.325301	0.09251	3.516258	0.000437675	0.004120271
ARPC3	640.2157644	0.325664	0.06201	5.251711	1.51E-07	2.89E-06
UQCR10	216.7887551	0.325798	0.09988	3.261986	0.001106348	0.009078158
HLA-B	3963.074973	0.326383	0.03509	9.300034	1.40E-20	8.79E-19
KLRG1	147.1032755	0.329191	0.11999	2.743592	0.0060771	0.037695636
FGFR1OP2	142.6206368	0.331336	0.12105	2.737217	0.006196146	0.038278729
CD63	605.8774623	0.332265	0.06202	5.357775	8.43E-08	1.67E-06
PDCD10	123.3881243	0.333507	0.12454	2.677894	0.007408663	0.043869779

TRIR	384.5808763	0.335176	0.07478	4.48215	7.39E-06	0.000108759
ARF5	131.6582674	0.336181	0.12604	2.667262	0.007647214	0.045008358
C8orf59	144.1869413	0.337072	0.12363	2.7265	0.006400998	0.03918231
HSPA1A	7326.632892	0.339682	0.04758	7.139158	9.39E-13	3.32E-11
BIN2	165.8079507	0.34109	0.1102	3.095195	0.001966835	0.01495745
HOPX	379.6380056	0.342603	0.07919	4.326373	1.52E-05	0.000207943
C19orf70	127.0068781	0.343036	0.12735	2.693697	0.007066429	0.042264277
TSPO	315.8586422	0.343576	0.08031	4.278384	1.88E-05	0.000253694
PSMA7	507.2551593	0.344431	0.0692	4.977193	6.45E-07	1.12E-05
NSD3	172.7462761	0.345032	0.10803	3.193939	0.001403457	0.011165047
ARPC4	212.4564124	0.34536	0.10162	3.398524	0.000677505	0.005966504
CCT4	255.0964844	0.345793	0.09486	3.645485	0.000266888	0.002699155
CBX3	226.279176	0.351474	0.09387	3.744399	0.000180826	0.001936774
ARHGAP30	110.3379168	0.355003	0.12918	2.748193	0.005992466	0.037257053
LAMTOR4	244.9338641	0.356088	0.09296	3.830457	0.000127906	0.001428591
RAC2	582.1448969	0.35691	0.06369	5.603598	2.10E-08	4.55E-07
SNHG15	201.7912214	0.35825	0.10299	3.478356	0.000504499	0.004641563
BRK1	144.5241103	0.360594	0.11884	3.034323	0.002410762	0.017763863
ATP5MC3	248.6104117	0.36153	0.08945	4.041767	5.30E-05	0.000651705
FIS1	166.811786	0.3648	0.11508	3.170018	0.001524296	0.012010287
NDUFB9	143.1769913	0.367029	0.12047	3.046687	0.002313784	0.01715696
PIN1	119.7252198	0.367325	0.12654	2.902745	0.003699075	0.025389386
NDUFB7	175.0218538	0.36776	0.10611	3.465738	0.000528779	0.004811168
DNAJB1	3521.288739	0.368442	0.05061	7.280503	3.33E-13	1.26E-11
EMP3	705.6316412	0.369221	0.05934	6.222427	4.90E-10	1.32E-08
TBCC	104.200369	0.374798	0.13885	2.699266	0.006949253	0.041817872
GPSM3	296.084714	0.376175	0.08302	4.53112	5.87E-06	8.82E-05
NEDD8	289.9585474	0.376298	0.08781	4.285376	1.82E-05	0.000246155
PDCD5	130.3535969	0.378158	0.12349	3.06231	0.002196361	0.016388465
ATP5PF	273.7924956	0.379588	0.08698	4.364287	1.28E-05	0.000178386
AIP	113.4234474	0.380314	0.13476	2.822129	0.004770603	0.031145537
TMCO1	160.3455337	0.381877	0.11146	3.426175	0.000612146	0.005468812
ISG15	314.8202577	0.383289	0.08647	4.432686	9.31E-06	0.000134751
TPM4	166.3326382	0.384883	0.10801	3.563259	0.000366279	0.003537175
WDR83OS	222.4562558	0.385485	0.09966	3.867911	0.000109772	0.001250862
PSMA4	158.5697622	0.385582	0.1152	3.346988	0.000816949	0.007005369
ARPC1B	271.6937029	0.389606	0.08896	4.379515	1.19E-05	0.000168569
CSTB	265.5977344	0.393117	0.08968	4.383516	1.17E-05	0.000166162
CD3D	867.3567378	0.395512	0.06078	6.506747	7.68E-11	2.25E-09
ITPA	84.44489769	0.395518	0.15046	2.628739	0.008570211	0.049222361
NDUFC1	91.95808479	0.396557	0.15103	2.625681	0.008647579	0.049560305
GTF2A2	131.1649622	0.397522	0.12046	3.299929	0.000967094	0.008090626
STMN1	90.48767134	0.398819	0.15023	2.65475	0.007936728	0.046431375
REEP5	187.1815465	0.400254	0.10221	3.916102	9.00E-05	0.001050073
HNRNPF	215.4897659	0.401951	0.09615	4.180572	2.91E-05	0.000379891
PGAM1	105.6247706	0.402122	0.13333	3.015973	0.002561563	0.018721803
HMOX2	83.07329655	0.402366	0.15016	2.679591	0.007371221	0.043720655
TRAPPC1	187.0296492	0.402797	0.10382	3.879601	0.000104628	0.001198628
PLAC8	147.2831832	0.403791	0.12394	3.257929	0.001122287	0.009194846
RSBN1L	118.9850623	0.407934	0.12597	3.238294	0.001202467	0.009754664
SLC9A3R1	159.7856113	0.408572	0.1105	3.697357	0.000217856	0.002254407
PGLS	85.22079189	0.409278	0.14724	2.779578	0.005442963	0.034502193

OSER1	179.1272144	0.41148	0.10919	3.768594	0.00016417	0.001786997
COX5A	136.1759899	0.411657	0.12061	3.413146	0.000642176	0.005692859
PSMC5	127.0534193	0.411674	0.12658	3.252214	0.001145099	0.009360243
C19orf24	105.3345136	0.412704	0.14304	2.885249	0.003911036	0.026536518
SCP2	139.5637243	0.413425	0.11617	3.558902	0.000372409	0.003586658
ADRM1	87.77500123	0.414441	0.155	2.673746	0.007500913	0.044281712
TNFRSF14	112.8535498	0.415195	0.12967	3.202042	0.001364572	0.010871865
GNB2	105.5274416	0.415365	0.13325	3.117153	0.001826069	0.01402065
TBCB	167.7555773	0.418461	0.10796	3.876226	0.000106089	0.001213237
SRSF9	266.0802003	0.419462	0.08757	4.790155	1.67E-06	2.75E-05
ATP5F1D	273.6480058	0.42038	0.08802	4.775946	1.79E-06	2.94E-05
PYURF	108.5118684	0.420877	0.13572	3.100996	0.001928712	0.014719839
ABHD17A	256.1480857	0.423388	0.08889	4.762812	1.91E-06	3.12E-05
CCT6A	122.9031312	0.424397	0.12608	3.36616	0.000762223	0.00660388
SUPT4H1	95.10318716	0.425659	0.14097	3.019406	0.002532707	0.018548914
SRP9	160.0614287	0.425802	0.11036	3.858475	0.000114097	0.00129326
CTSD	171.978988	0.426269	0.10658	3.999403	6.35E-05	0.00076951
ACTR3	268.3866858	0.426773	0.08664	4.925658	8.41E-07	1.45E-05
CHMP2A	105.7888383	0.428122	0.14536	2.945204	0.003227416	0.022749242
ATP5F1B	158.9917184	0.429461	0.11449	3.751201	0.00017599	0.001896016
TMEM9B	76.6566784	0.429566	0.15836	2.712546	0.006676861	0.040477289
ZYX	90.08338395	0.429718	0.14503	2.962894	0.003047612	0.021725148
NDUFC2	133.3630411	0.432317	0.12189	3.546682	0.000390114	0.003730317
RHOG	99.45881746	0.432382	0.14061	3.07494	0.002105449	0.01588738
DR1	69.13974348	0.433148	0.16383	2.643846	0.008197002	0.047544574
PSMB10	79.39169208	0.433891	0.15446	2.809169	0.004966951	0.032151468
ARPC5	325.7014479	0.434043	0.07919	5.480959	4.23E-08	8.76E-07
CENPX	74.50588239	0.435355	0.16357	2.661656	0.007775721	0.045639176
RASSF1	99.0201849	0.436962	0.1421	3.074958	0.002105319	0.01588738
RARRES3	452.8711246	0.437079	0.07417	5.893084	3.79E-09	9.00E-08
LCP2	114.4693651	0.437959	0.13124	3.337174	0.000846348	0.00721588
NDUFB10	166.8589699	0.439965	0.10982	4.006204	6.17E-05	0.000749394
RNF187	76.87867356	0.440381	0.15534	2.834932	0.00458355	0.030218104
DCN	278.5929817	0.440397	0.08797	5.006355	5.55E-07	9.76E-06
HSPA1B	3243.537591	0.440477	0.08179	5.385327	7.23E-08	1.44E-06
IDH2	85.92269565	0.440662	0.1533	2.874564	0.004045859	0.02729552
PHB2	90.21666219	0.443108	0.14557	3.043922	0.002335159	0.017303465
CNPY2	73.15730547	0.443428	0.16128	2.749459	0.005969368	0.037159338
HDAC1	79.38703129	0.44499	0.15798	2.816796	0.004850539	0.031550619
HSPE1	2697.607432	0.445112	0.04627	9.618845	6.66E-22	4.57E-20
FKBP2	116.7423961	0.446008	0.13215	3.375078	0.000737948	0.006415754
CD74	430.7405381	0.446557	0.07218	6.187019	6.13E-10	1.62E-08
PPP4C	93.06132024	0.447251	0.14411	3.10359	0.001911883	0.01461225
DEDD2	252.0562357	0.447601	0.09027	4.958597	7.10E-07	1.23E-05
NDUFS6	166.1039655	0.44976	0.10989	4.092888	4.26E-05	0.000534361
TUBB	117.9528964	0.450282	0.13111	3.434354	0.000593969	0.005330156
KIF2A	118.0020986	0.450524	0.13025	3.459031	0.000542122	0.004918473
EMC6	69.1938357	0.451587	0.16425	2.749435	0.005969804	0.037159338
CFD	511.1369935	0.452403	0.07191	6.291446	3.15E-10	8.56E-09
TRAC	725.5774322	0.453501	0.0621	7.302606	2.82E-13	1.09E-11
SNRPC	98.18476535	0.453888	0.14018	3.237908	0.001204097	0.009760483
RASAL3	130.0301127	0.455199	0.1228	3.706772	0.000209918	0.002184945

DRAP1	254.0162423	0.456601	0.09342	4.887376	1.02E-06	1.74E-05
SLF1	77.3647297	0.457351	0.16444	2.781306	0.005414063	0.034359714
CDK2AP2	129.7278662	0.459546	0.1212	3.791667	0.00014964	0.001642199
TNFAIP8	166.9532206	0.459615	0.10948	4.198344	2.69E-05	0.000353869
IRF3	89.25501313	0.461132	0.14721	3.132485	0.001733333	0.013391093
VAMP8	271.2350099	0.461677	0.08703	5.304675	1.13E-07	2.20E-06
RAC1	251.2948579	0.462178	0.09132	5.061226	4.17E-07	7.55E-06
RGS2	365.8935903	0.462343	0.07937	5.825021	5.71E-09	1.33E-07
LAMTOR1	110.136111	0.463137	0.13252	3.494832	0.00047436	0.004405948
C12orf75	250.0107807	0.463848	0.09187	5.048794	4.45E-07	8.00E-06
GSDMD	72.86540947	0.465199	0.16413	2.834253	0.004593291	0.030245057
SSNA1	109.9651711	0.468825	0.13237	3.541731	0.00039751	0.003790874
ATG101	91.64280794	0.472144	0.15392	3.067515	0.002158468	0.016162074
CD81	385.2433466	0.473264	0.07646	6.189944	6.02E-10	1.59E-08
SAMD3	198.456206	0.474691	0.10142	4.680592	2.86E-06	4.58E-05
COMMD8	70.09408688	0.475728	0.16538	2.876622	0.004019569	0.027152394
SRA1	75.04365709	0.475966	0.15826	3.007416	0.002634786	0.019139311
THYN1	60.82453645	0.47812	0.18034	2.651146	0.008021904	0.046725299
MATK	185.6877891	0.478135	0.10421	4.588013	4.47E-06	6.89E-05
FKBP1A	129.3463632	0.479407	0.12488	3.83882	0.000123627	0.001388045
CNIH4	68.7556591	0.48026	0.1713	2.8037	0.005051995	0.032564066
VKORC1	59.05915855	0.480632	0.17653	2.722703	0.006475033	0.03951315
IRF2	84.16807898	0.483497	0.15215	3.177688	0.001484543	0.011722958
CST3	112.5741017	0.483883	0.13406	3.609439	0.00030686	0.003037378
ETFA	64.64458766	0.488213	0.18485	2.641069	0.00826449	0.047851757
GZMA	1405.418797	0.488402	0.04959	9.849733	6.87E-23	4.81E-21
NDUFS4	65.87546902	0.488551	0.17295	2.824876	0.004729887	0.030935079
PTPN4	100.4849927	0.488912	0.13905	3.516166	0.000437827	0.004120271
CHCHD5	53.99958861	0.489553	0.18638	2.62663	0.008623495	0.049448767
GTF3C6	88.14189666	0.490311	0.15364	3.191383	0.001415933	0.011247574
GLRX3	53.66481165	0.490682	0.18642	2.632193	0.008483576	0.048908547
ACTG1	1903.9703	0.491248	0.04133	11.8847	1.42E-32	1.49E-30
RHOF	104.1455877	0.492858	0.1341	3.675297	0.000237573	0.002430239
TNFSF9	214.6140072	0.499018	0.10646	4.687355	2.77E-06	4.45E-05
ECHS1	65.94499724	0.499856	0.18351	2.723897	0.006451672	0.039424838
FAM89B	96.4641278	0.50051	0.14276	3.505984	0.000454923	0.00425496
SEC11A	191.8024299	0.50164	0.10302	4.869288	1.12E-06	1.89E-05
TNFSF12	60.28237801	0.502298	0.17615	2.851533	0.004350895	0.029005969
LTB	228.2263861	0.503585	0.10061	5.00556	5.57E-07	9.79E-06
MRPL18	479.6074161	0.503849	0.07179	7.018054	2.25E-12	7.64E-11
RGS14	58.81472436	0.50657	0.18039	2.808141	0.004982839	0.032215334
SP110	90.0919753	0.507788	0.14631	3.470715	0.000519074	0.004738985
TIMM8B	55.3694436	0.510522	0.19004	2.686323	0.00722432	0.043040214
APOBEC3C	145.3718346	0.51196	0.12033	4.254635	2.09E-05	0.000279361
HSD17B10	60.3246568	0.512094	0.18247	2.806482	0.005008572	0.032359969
KDELR1	75.6755158	0.513274	0.16123	3.183481	0.001455158	0.01151641
MRPL42	55.08799063	0.51337	0.18614	2.757999	0.005815634	0.036411519
ZFAND2A	426.4957421	0.515229	0.07128	7.228029	4.90E-13	1.81E-11
NDUFS8	103.7559832	0.516235	0.13751	3.754283	0.000173838	0.001878856
HSPB11	143.939768	0.517438	0.11879	4.355959	1.32E-05	0.000184104
FCRL6	59.68954016	0.518818	0.17809	2.913208	0.003577358	0.024695307
RPA2	83.68845831	0.521246	0.16574	3.14502	0.001660756	0.012920091

PMVK	50.88800179	0.523448	0.18974	2.758788	0.005801622	0.03636635
FIBP	61.78386833	0.526027	0.18471	2.847791	0.004402387	0.029250872
PRR5	54.66278137	0.526827	0.19018	2.770082	0.005604211	0.035313481
SELENOH	168.1825519	0.527048	0.11018	4.783531	1.72E-06	2.84E-05
TADA3	53.97722648	0.527095	0.18871	2.79309	0.005220719	0.033370188
TIMM10	56.51885907	0.527187	0.18437	2.859358	0.004244999	0.028406186
NDUFAB1	118.3841308	0.530316	0.13108	4.045771	5.22E-05	0.000642452
GSN	100.287806	0.531432	0.13857	3.835184	0.00012547	0.001402853
TMEM160	124.5313898	0.532296	0.12825	4.150304	3.32E-05	0.000425481
SMIM37	59.8785646	0.53275	0.18725	2.845192	0.00443847	0.029372425
PPP1CA	180.9387854	0.533857	0.10697	4.990662	6.02E-07	1.06E-05
FAM173A	74.24371512	0.53416	0.16065	3.325063	0.000883985	0.007489022
TRGC1	60.89234479	0.534208	0.19686	2.713585	0.006655953	0.040419237
FAM200B	53.1835559	0.536858	0.19408	2.766113	0.005672881	0.035663825
NDUFB3	108.6531616	0.536974	0.1383	3.882686	0.000103309	0.001188107
RNF167	91.57040774	0.537411	0.14925	3.600663	0.000317407	0.003118692
WASHC3	84.57407756	0.539935	0.15041	3.589712	0.000331044	0.003234857
GPR174	91.70410474	0.540899	0.14527	3.723287	0.000196646	0.002073688
ZNRD1	103.3931353	0.542185	0.13646	3.973156	7.09E-05	0.000847002
SELPLG	48.84052151	0.543229	0.19556	2.777871	0.005471641	0.034642933
PLEKHJ1	83.48300361	0.543958	0.15749	3.453852	0.00055264	0.005002746
C1orf35	56.27782119	0.544069	0.18995	2.864332	0.004178904	0.028051612
CGAS	58.39046491	0.544212	0.17967	3.028983	0.002453787	0.018007899
TALDO1	95.42314773	0.544354	0.14212	3.830185	0.000128047	0.001428675
TRIM8	54.48371455	0.544714	0.19182	2.839672	0.00451599	0.029846258
SYNRG	128.2412711	0.544737	0.12702	4.288666	1.80E-05	0.000243151
ARHGAP25	51.70915883	0.544996	0.19546	2.788317	0.005298266	0.033724832
POLR2G	89.48334082	0.545642	0.14628	3.730055	0.000191438	0.002032131
AKR1A1	50.58895148	0.548186	0.19259	2.846402	0.004421635	0.029331367
CKS2	264.3621573	0.550515	0.09819	5.606768	2.06E-08	4.47E-07
MRPL14	47.33518248	0.550853	0.19692	2.797304	0.005153104	0.033036675
IMMT	48.1326401	0.552611	0.20135	2.744577	0.006058902	0.037604555
CORO1A	856.258995	0.554052	0.05498	10.07685	6.99E-24	5.13E-22
CLIC1	824.473995	0.55526	0.05561	9.985657	1.76E-23	1.26E-21
ITM2C	99.00220777	0.556698	0.14024	3.96969	7.20E-05	0.00085655
CARHSP1	76.16628232	0.560269	0.16266	3.444416	0.000572294	0.0051545
PSMA5	162.2822666	0.562699	0.11155	5.044277	4.55E-07	8.15E-06
GSTP1	345.1015503	0.564344	0.08212	6.872025	6.33E-12	2.08E-10
SLAMF1	52.28289567	0.564497	0.19325	2.921009	0.003488998	0.024210294
CTSW	647.2740241	0.565232	0.06251	9.041811	1.54E-19	9.21E-18
LAGE3	46.49443547	0.566678	0.20152	2.811973	0.004923862	0.0319305
SLC25A38	48.57046966	0.566831	0.20351	2.785331	0.005347311	0.033976382
CKLF	324.0181919	0.566897	0.08503	6.667276	2.61E-11	8.13E-10
ETHE1	56.09687402	0.569543	0.18596	3.062783	0.002192893	0.016383822
PSMD4	88.58063594	0.570593	0.14693	3.883524	0.000102953	0.001185794
COTL1	274.6910606	0.570892	0.09064	6.298789	3.00E-10	8.19E-09
CCDC107	169.7771924	0.571589	0.10727	5.328349	9.91E-08	1.94E-06
PARP1	75.86969745	0.572054	0.15823	3.615328	0.000299968	0.002985723
IMP3	72.16724272	0.572692	0.16425	3.486781	0.000488871	0.004517201
CD300A	50.00630052	0.573995	0.19554	2.935447	0.003330678	0.023319428
CHST12	93.44631259	0.575688	0.14307	4.023785	5.73E-05	0.000701936
BST2	190.3382871	0.575997	0.10599	5.434465	5.50E-08	1.12E-06

TXNDC9	49.39330001	0.576214	0.20088	2.868449	0.004124892	0.027741263
CCDC85B	196.8097849	0.576535	0.10442	5.521093	3.37E-08	7.14E-07
IDH3G	70.04859106	0.576844	0.16571	3.480962	0.000499616	0.004600594
COPZ1	72.42214578	0.577659	0.16512	3.498458	0.000467956	0.004357818
CAP1	279.8998247	0.578567	0.0894	6.471573	9.70E-11	2.83E-09
SDF2L1	87.34025742	0.579463	0.15365	3.771315	0.000162389	0.001769416
UBLCP1	54.92580023	0.579729	0.1842	3.147292	0.001647901	0.012833
AP2S1	97.60550064	0.5815	0.13921	4.177172	2.95E-05	0.000384205
ARMCX6	42.511337	0.583203	0.2116	2.756127	0.005849036	0.036535133
UBE2M	114.2993538	0.585302	0.13389	4.371459	1.23E-05	0.00017445
PSMC3	61.23918189	0.585441	0.18143	3.226805	0.001251805	0.010113531
SUCLG1	69.55637078	0.589709	0.1663	3.54611	0.000390963	0.003735095
MGAT1	52.73322551	0.590774	0.1933	3.056305	0.002240835	0.01666258
CYB561D2	61.10251656	0.592046	0.17689	3.346945	0.000817075	0.007005369
MAD1L1	60.31632418	0.593059	0.17651	3.359868	0.000779797	0.006745213
SPAG7	62.76597528	0.594575	0.17775	3.344936	0.000823016	0.007050193
PIH1D1	57.98799242	0.599734	0.18184	3.298077	0.000973493	0.008131444
MRFAP1L1	35.7067087	0.599824	0.22824	2.628084	0.00858672	0.049277447
LYSMD2	58.47443863	0.603789	0.18732	3.223215	0.001267604	0.010213376
FDPS	69.00681678	0.604802	0.17912	3.376612	0.000733844	0.006389041
MPLKIP	60.59783503	0.605439	0.17764	3.408152	0.000654044	0.005778916
C1orf122	69.30766041	0.606817	0.16972	3.575437	0.000349643	0.003397985
RNF126	65.4166504	0.607051	0.1749	3.470862	0.000518791	0.004738985
PPM1G	104.4597584	0.607554	0.13878	4.377694	1.20E-05	0.000169759
NDUFS2	53.84087653	0.60911	0.19606	3.106745	0.001891593	0.014477856
TMEM141	50.48743861	0.60953	0.20339	2.996879	0.002727591	0.019746433
MPST	38.87319408	0.611738	0.22379	2.733505	0.006266414	0.038601401
CMC2	173.5018878	0.612415	0.10691	5.728066	1.02E-08	2.32E-07
SLC25A11	48.253128	0.613621	0.1995	3.075779	0.002099533	0.015865117
HDDC2	54.6318437	0.615967	0.1883	3.271272	0.001070649	0.008825843
LAT	179.2611949	0.616764	0.10954	5.630524	1.80E-08	3.98E-07
RBCK1	97.81593938	0.619805	0.14417	4.29922	1.71E-05	0.000233035
MRPL4	49.00541208	0.620257	0.19866	3.12215	0.001795352	0.013812448
PSME2	235.4343017	0.621486	0.09771	6.360626	2.01E-10	5.67E-09
CKAP2	36.12702124	0.622526	0.23453	2.654359	0.007945929	0.046434428
TTN	44.36974043	0.623514	0.21602	2.886379	0.003897026	0.026458234
PDLIM2	111.9303642	0.624286	0.13123	4.757056	1.96E-06	3.21E-05
MEA1	46.63466303	0.62605	0.20207	3.098193	0.001947049	0.014838621
CCR5	49.38477639	0.626104	0.19845	3.154936	0.001605331	0.012547144
USP28	36.31422415	0.627275	0.23041	2.722404	0.00648089	0.03951315
IFI35	42.33722153	0.629542	0.21688	2.90273	0.003699257	0.025389386
UBTF	73.96483386	0.629717	0.16111	3.908588	9.28E-05	0.001079034
PRDX3	34.71031843	0.63005	0.23095	2.72806	0.006370807	0.039051069
CD247	285.69629	0.630817	0.08674	7.272126	3.54E-13	1.33E-11
CYB5B	40.25452159	0.631706	0.22317	2.830551	0.004646785	0.030559681
PSMB9	354.1720755	0.633029	0.07681	8.241396	1.70E-16	8.17E-15
RRP36	40.94172234	0.633725	0.21648	2.927403	0.003418053	0.023841697
CASP1	70.9772622	0.634323	0.16257	3.901815	9.55E-05	0.001106794
TRBC1	656.9227072	0.635192	0.07052	9.00716	2.11E-19	1.24E-17
AK6	41.5868332	0.635415	0.21124	3.008067	0.002629155	0.019124374
CHCHD1	61.66934764	0.636436	0.17841	3.567305	0.000360672	0.003489321
PSMB8	209.4412519	0.637059	0.10416	6.116308	9.58E-10	2.45E-08

FBXW5	67.55348567	0.637245	0.17732	3.593796	0.000325896	0.003196226
MRPL34	61.52405549	0.637458	0.18397	3.464918	0.000530393	0.004821759
CASP4	111.474139	0.638933	0.13478	4.740527	2.13E-06	3.46E-05
APOD	76.15012787	0.639009	0.16384	3.90013	9.61E-05	0.001113321
RPS19BP1	130.3350286	0.639082	0.1271	5.028187	4.95E-07	8.79E-06
GOPC	64.47349952	0.640236	0.17258	3.709702	0.000207504	0.002164024
ITGB2	412.7131567	0.644502	0.07513	8.578099	9.65E-18	4.96E-16
TESC	49.44662081	0.64529	0.1969	3.277264	0.001048185	0.00866326
BANF1	115.5418524	0.646035	0.13351	4.83898	1.31E-06	2.18E-05
FKBP4	82.33974211	0.647672	0.15542	4.167172	3.08E-05	0.000397578
TRNAU1AP	33.52506738	0.647804	0.23894	2.711131	0.006705404	0.040627309
NFYB	34.79092845	0.648923	0.23703	2.737726	0.006186566	0.038263734
LCK	251.0172722	0.649674	0.09432	6.88802	5.66E-12	1.86E-10
CYC1	55.98460831	0.649925	0.19855	3.27335	0.001062808	0.008767959
PIM1	85.70680569	0.650097	0.15111	4.302195	1.69E-05	0.00023022
TMEM242	38.32679137	0.650567	0.22482	2.893757	0.003806631	0.025943278
ANXA2R	62.28942012	0.654433	0.18163	3.60317	0.000314359	0.00309728
KLRC2	49.21357648	0.6547	0.20875	3.136261	0.001711167	0.013248545
NT5C	95.83600541	0.655637	0.14888	4.403898	1.06E-05	0.000152093
FBXL15	39.36498177	0.657535	0.21682	3.032596	0.002424601	0.017842664
ACBD5	35.94320538	0.657706	0.23995	2.741018	0.00612491	0.037926234
NUDT2	30.40845144	0.659742	0.25048	2.633921	0.008440507	0.048686485
NOP10	180.6462113	0.661312	0.11961	5.528946	3.22E-08	6.87E-07
TNFSF14	66.55361004	0.664929	0.17904	3.71392	0.000204073	0.002138672
C16orf91	36.17988263	0.666238	0.22988	2.898239	0.003752645	0.025673468
ORMDL2	67.44469802	0.669888	0.17056	3.927611	8.58E-05	0.001009898
LAMTOR2	72.05443635	0.67512	0.16549	4.079501	4.51E-05	0.000562262
TIGIT	104.1263315	0.675827	0.15414	4.384376	1.16E-05	0.000165727
TRDC	118.156396	0.676189	0.14084	4.801196	1.58E-06	2.62E-05
SERTAD3	68.47139164	0.676502	0.16998	3.979844	6.90E-05	0.000826292
TWF2	77.4617124	0.67736	0.16018	4.228816	2.35E-05	0.000311872
STN1	46.97338632	0.680325	0.20819	3.267764	0.001084006	0.008929073
PRELID1	212.2889183	0.680446	0.10033	6.781974	1.19E-11	3.81E-10
PTPN7	146.3899329	0.681994	0.12374	5.511585	3.56E-08	7.51E-07
CACYBP	393.5565823	0.682647	0.07616	8.963154	3.16E-19	1.83E-17
DCP2	61.37945286	0.683801	0.18714	3.653969	0.000258218	0.002616412
TSC22D1	28.43160402	0.687256	0.25958	2.647562	0.008107449	0.047172213
CYTH4	47.16665152	0.689373	0.20387	3.381397	0.000721182	0.006294163
UBE2F	28.04487788	0.689505	0.25787	2.673892	0.007497665	0.044281712
SIRPG	28.41749968	0.692979	0.25842	2.681583	0.007327473	0.043509411
KRCC1	35.10580571	0.693927	0.23446	2.959697	0.003079414	0.021893507
HLA-DPA1	256.3292867	0.696068	0.09141	7.614915	2.64E-14	1.13E-12
TRPV2	43.44941315	0.696086	0.21623	3.219128	0.001285812	0.01032122
CARD16	115.4549871	0.696678	0.13535	5.147072	2.65E-07	4.88E-06
PFN1	1912.713389	0.699934	0.04746	14.74933	3.11E-49	5.54E-47
C8orf33	28.0530814	0.70082	0.25719	2.724951	0.006431107	0.039344106
NSL1	37.47585444	0.703992	0.22948	3.067776	0.00215658	0.016159246
NMI	53.96003	0.704522	0.18796	3.748231	0.000178086	0.00191164
TSEN34	44.35859364	0.710069	0.21073	3.369611	0.000752743	0.006532323
GSTO1	89.18625771	0.713612	0.14869	4.799485	1.59E-06	2.63E-05
HLA-DPB1	324.2690255	0.713999	0.0865	8.254353	1.53E-16	7.39E-15
CDKN2D	113.3380923	0.714689	0.13652	5.235095	1.65E-07	3.14E-06

TRAF3IP3	150.9420026	0.715158	0.11928	5.995519	2.03E-09	4.94E-08
RHOC	120.9904265	0.716038	0.13289	5.388319	7.11E-08	1.42E-06
RGS19	93.3409863	0.718012	0.14391	4.989417	6.06E-07	1.06E-05
PFDN6	48.57654936	0.719534	0.19933	3.609817	0.000306414	0.003035765
TMEM126B	48.53189594	0.719638	0.19699	3.653114	0.000259079	0.002622655
MRPL28	46.1392177	0.720084	0.20254	3.555277	0.000377582	0.003626683
MRPS6	147.1340035	0.720551	0.12064	5.972773	2.33E-09	5.65E-08
LSM6	93.40507746	0.722307	0.14476	4.989829	6.04E-07	1.06E-05
GZMH	600.2154216	0.723911	0.11817	6.125944	9.01E-10	2.33E-08
COMMD3	37.22267643	0.725577	0.22424	3.235743	0.001213266	0.009827364
SAMD9L	67.87703089	0.726104	0.16824	4.315771	1.59E-05	0.0002179
MOSPD3	27.11359238	0.726373	0.26697	2.720817	0.006512086	0.03965153
GMEB1	31.00621865	0.727898	0.25033	2.907752	0.003640369	0.02505671
BAD	38.22980182	0.728337	0.2231	3.264659	0.001095958	0.009007633
MAP7D1	40.97848649	0.729478	0.22346	3.264413	0.001096911	0.009007633
GIMAP4	182.5416933	0.730635	0.10757	6.791963	1.11E-11	3.57E-10
YDJC	31.22339968	0.73106	0.25199	2.901157	0.003717871	0.025484444
UBE2L6	67.45842589	0.733596	0.16926	4.334163	1.46E-05	0.000201473
HDDC3	25.35900004	0.737921	0.27421	2.691082	0.007122061	0.042573211
GRK6	66.32947272	0.739309	0.17167	4.306485	1.66E-05	0.000226378
MED11	30.8516063	0.739406	0.25122	2.943249	0.003247872	0.022848274
CALHM2	26.10719756	0.741296	0.28192	2.629452	0.008552257	0.049222361
NDUFA7	31.42962161	0.742185	0.25338	2.929146	0.003398942	0.023754853
C19orf12	28.10778846	0.747129	0.27437	2.723038	0.006468468	0.039504915
NUDT22	47.63927487	0.748153	0.20087	3.724521	0.000195686	0.002066975
PLA2G16	143.0876419	0.751571	0.12288	6.116369	9.57E-10	2.45E-08
DENND2D	60.2033827	0.752572	0.17962	4.189882	2.79E-05	0.000365528
ARHGEF2	25.64498476	0.752916	0.27254	2.762599	0.005734322	0.03602892
AP1S1	26.60764997	0.754141	0.27966	2.696611	0.007004901	0.041990163
SMPD3	23.7994205	0.754824	0.2839	2.658763	0.007842815	0.045932196
IFNG	294.4478225	0.757713	0.10189	7.436693	1.03E-13	4.14E-12
SLAMF7	79.42366153	0.760182	0.16013	4.74737	2.06E-06	3.35E-05
C15orf61	41.52206431	0.766004	0.21511	3.561055	0.000369368	0.003563789
C1orf162	37.37130849	0.76651	0.22711	3.375017	0.000738111	0.006415754
CSK	112.8249156	0.766536	0.13511	5.673327	1.40E-08	3.14E-07
PAFAH1B3	26.19857109	0.76709	0.27692	2.77011	0.005603744	0.035313481
UQCC3	24.68625136	0.768517	0.27638	2.780628	0.005425379	0.034411117
GBP1	44.03267175	0.770974	0.20873	3.693666	0.000221044	0.002282984
GLRX2	26.38392585	0.773264	0.26566	2.910677	0.003606464	0.024848144
PPP1R14B	95.13438021	0.773876	0.15018	5.152861	2.57E-07	4.75E-06
IL16	60.25007277	0.776958	0.18175	4.274796	1.91E-05	0.000257489
RARRES2	30.80042059	0.778559	0.2566	3.034139	0.002412233	0.017763863
LIMA1	47.30083781	0.780849	0.21286	3.668344	0.000244127	0.002487767
FGL2	21.70718061	0.782677	0.29486	2.654413	0.00794466	0.046434428
VAV1	30.01512627	0.785111	0.25324	3.100235	0.001933671	0.014747173
TEX30	24.08756883	0.785892	0.28163	2.790501	0.005262651	0.033573163
CASP7	23.58241816	0.787884	0.28941	2.722404	0.006480895	0.03951315
HLA-DRA	233.2186639	0.788046	0.1035	7.61368	2.66E-14	1.14E-12
BLVRA	27.36710987	0.788471	0.26839	2.937767	0.003305856	0.023185665
CIAPIN1	21.99772635	0.78952	0.29393	2.68611	0.007228923	0.04304367
RPF2	30.52118642	0.791101	0.26929	2.937691	0.003306666	0.023185665
PSD4	37.41320847	0.791388	0.23711	3.337599	0.000845057	0.00721588

SFT2D1	90.70752604	0.791763	0.14977	5.286533	1.25E-07	2.42E-06
TNFRSF1A	32.38225411	0.792237	0.24829	3.190771	0.001418938	0.011263081
ACAA2	90.76638348	0.792436	0.14805	5.352457	8.68E-08	1.71E-06
TUSC2	38.38105953	0.794587	0.23114	3.43776	0.000586547	0.005273996
B4GALT3	26.89837249	0.795839	0.28234	2.818712	0.004821675	0.031409824
GIMAP1	99.01683663	0.796966	0.14532	5.484266	4.15E-08	8.61E-07
S100A13	28.82247266	0.799598	0.26318	3.038215	0.002379841	0.017587607
PTPN18	46.57492864	0.799754	0.21005	3.807447	0.000140409	0.001550436
PLEKHF1	46.06962314	0.80162	0.20764	3.860583	0.000113117	0.00128487
PDZD11	32.80668814	0.801791	0.24205	3.312542	0.000924522	0.007783152
SH3BP1	72.6088821	0.802726	0.16598	4.836155	1.32E-06	2.21E-05
SELENOP	45.76814168	0.804989	0.2058	3.911581	9.17E-05	0.001068712
CKS1B	34.79433765	0.805565	0.23648	3.406486	0.000658049	0.00580472
SNRNP35	24.6054934	0.806297	0.2875	2.804471	0.005039925	0.032525451
HLA-DMB	20.72293104	0.811908	0.30249	2.684082	0.00727292	0.043272244
RPS6KA1	26.73562493	0.812155	0.26732	3.038185	0.00238008	0.017587607
CXCL14	49.16848473	0.812288	0.19931	4.075508	4.59E-05	0.000571254
TOX	63.3918557	0.813536	0.18213	4.466892	7.94E-06	0.000116169
HPF1	40.19087836	0.813827	0.23241	3.501736	0.000462238	0.0043196
ZBTB2	37.60141475	0.815807	0.23064	3.537111	0.00040453	0.003847532
BIN1	85.09682352	0.815826	0.16199	5.036294	4.75E-07	8.46E-06
CTSZ	39.07730703	0.816599	0.22073	3.699578	0.000215958	0.002236931
PTPRN2	22.07480094	0.816762	0.30188	2.705623	0.006817633	0.041190667
PYCARD	103.8830136	0.820325	0.13946	5.882128	4.05E-09	9.55E-08
DCTPP1	24.59283381	0.821038	0.27869	2.946033	0.003218783	0.022731272
MRPS18B	32.40106453	0.821657	0.26218	3.133899	0.001725001	0.013345997
GBP3	28.22838101	0.822336	0.25976	3.165814	0.001546499	0.012176258
NDUFAF4	46.26354454	0.823584	0.21327	3.861744	0.000112581	0.00128014
LSM2	78.23236441	0.826548	0.15902	5.197754	2.02E-07	3.81E-06
MRPL36	37.25805487	0.828668	0.24004	3.452177	0.000556083	0.005021169
CDK4	28.15983831	0.831338	0.26329	3.157445	0.001591583	0.01246701
ARL5B	54.57087653	0.833445	0.19546	4.264083	2.01E-05	0.000269463
MAPKAPK3	23.16480185	0.841402	0.30188	2.787247	0.005315798	0.033816312
STARD3NL	45.56200681	0.841985	0.21136	3.983632	6.79E-05	0.000814136
UROD	20.99498177	0.844529	0.30477	2.770994	0.005588541	0.035257894
NKG7	2173.38294	0.845249	0.05003	16.89623	4.80E-64	1.12E-61
GPX3	32.11897602	0.847259	0.25805	3.283344	0.001025832	0.008508843
SVBP	31.67964698	0.852159	0.24943	3.416491	0.000634338	0.005632711
GIMAP6	30.51635994	0.853455	0.26035	3.278165	0.001044843	0.00864642
CISD3	54.73068613	0.857095	0.1915	4.475706	7.62E-06	0.000111631
HES4	23.9293731	0.859388	0.29668	2.896677	0.003771383	0.025768708
MRPL16	36.56442437	0.861902	0.22953	3.755116	0.000173261	0.001874514
TBC1D10C	194.5927193	0.861928	0.10416	8.275301	1.28E-16	6.26E-15
SCNM1	32.2902676	0.863008	0.24509	3.521128	0.000429716	0.004054636
UBASH3A	29.7615644	0.863131	0.25438	3.393136	0.000690972	0.006065139
DCUN1D5	42.79075556	0.864742	0.21183	4.082219	4.46E-05	0.000556945
TSC22D4	77.74047895	0.867618	0.16333	5.311936	1.08E-07	2.12E-06
MTCH2	28.85948863	0.867933	0.25877	3.354089	0.000796266	0.006859942
POLR3K	35.08751779	0.86801	0.23713	3.660433	0.000251789	0.002556114
TTC38	26.9707625	0.868776	0.27568	3.151354	0.001625155	0.01267431
CCM2	73.11108483	0.870883	0.16309	5.339778	9.31E-08	1.83E-06
C9orf85	23.04142483	0.872541	0.29899	2.918322	0.003519203	0.024356706

CDKN2C	26.06005251	0.876309	0.27385	3.199925	0.001374632	0.010943869
TMEM101	21.70924535	0.88117	0.29715	2.965407	0.00302283	0.021562853
NTPCR	16.83728266	0.881707	0.33611	2.623257	0.008709353	0.049781022
DUSP23	54.70762553	0.883015	0.19073	4.629776	3.66E-06	5.74E-05
GIMAP7	271.4235323	0.88719	0.0946	9.378222	6.71E-21	4.30E-19
COL6A2	50.13211401	0.887286	0.20301	4.370616	1.24E-05	0.000174894
CCL3	432.3867295	0.889096	0.32607	2.726694	0.006397231	0.039181665
CCNG2	18.5201617	0.889628	0.32687	2.721655	0.006495588	0.03958018
TNFSF10	56.45489792	0.893205	0.19154	4.663353	3.11E-06	4.94E-05
MRPL12	19.15606847	0.893386	0.31599	2.827224	0.004695346	0.030811075
MRPS12	48.6447523	0.894037	0.20279	4.408779	1.04E-05	0.000149104
HSPB1	247.3400872	0.896297	0.09889	9.063371	1.26E-19	7.60E-18
MRPS34	82.27244797	0.897349	0.15744	5.699676	1.20E-08	2.72E-07
PVT1	16.70944023	0.89973	0.34224	2.628982	0.008564085	0.049222361
MIF4GD	29.72028245	0.90199	0.25278	3.568335	0.000359257	0.003478777
SMIM15	32.65948205	0.905584	0.25155	3.600038	0.000318171	0.003123329
CCDC80	45.75225004	0.906063	0.20797	4.356768	1.32E-05	0.000183663
MRPL51	124.274737	0.908882	0.12898	7.046689	1.83E-12	6.30E-11
MAD2L1	17.05446588	0.910874	0.34645	2.629157	0.008559677	0.049222361
ISCA2	31.58273804	0.913504	0.24808	3.682316	0.000231125	0.002368806
PYM1	26.49029974	0.925205	0.27253	3.394858	0.000686642	0.006032075
VPS72	22.97738621	0.927945	0.28798	3.222242	0.001271915	0.010240402
NELFCD	50.60226809	0.931563	0.20289	4.591452	4.40E-06	6.79E-05
JAKMIP2	23.8133695	0.932833	0.30262	3.082557	0.002052304	0.015530165
SNX20	21.83473664	0.933735	0.3026	3.085672	0.00203093	0.015390192
TIMM13	41.69820727	0.934348	0.22413	4.168724	3.06E-05	0.000396314
FAM217B	23.56934382	0.935374	0.28651	3.264684	0.001095862	0.009007633
ZMAT5	25.12042362	0.935909	0.28068	3.334487	0.00085457	0.00727535
GADD45G	85.89735046	0.936575	0.1527	6.133435	8.60E-10	2.23E-08
EMC9	19.57378271	0.940347	0.314	2.994688	0.002747257	0.019848518
APOBEC3G	323.4767465	0.94867	0.08785	10.79856	3.50E-27	3.07E-25
BARD1	23.05643965	0.950179	0.29374	3.234745	0.001217515	0.009854316
AGTRAP	117.3541171	0.950899	0.1336	7.117498	1.10E-12	3.86E-11
DNTTIP1	17.30243361	0.951285	0.33973	2.800153	0.005107833	0.032844839
HSPA6	1295.177689	0.953753	0.05913	16.13026	1.56E-58	3.35E-56
LUM	103.7580689	0.95615	0.14428	6.627175	3.42E-11	1.06E-09
CD244	23.74566499	0.957824	0.29168	3.283831	0.001024063	0.008500752
EOMES	42.98989735	0.958383	0.21648	4.427045	9.55E-06	0.000137652
EIF4EBP1	16.08198	0.958505	0.34621	2.76857	0.005630291	0.035437714
DAXX	31.41969348	0.960007	0.25031	3.835216	0.000125454	0.001402853
ACP5	23.3317653	0.962767	0.29187	3.298638	0.00097155	0.008121551
VAMP5	87.29247351	0.967224	0.15236	6.348258	2.18E-10	6.09E-09
PLPP3	15.68452623	0.969113	0.35478	2.731612	0.00630253	0.038756937
ARL14EP	44.04169586	0.971044	0.21467	4.523365	6.09E-06	9.10E-05
PTTG1	96.1068725	0.975299	0.15365	6.347445	2.19E-10	6.10E-09
RPS26	2084.825042	0.975715	0.0439	22.22695	1.88E-109	9.17E-107
ABI3	59.74969472	0.98128	0.18167	5.401465	6.61E-08	1.33E-06
PPP1R18	133.3396394	0.985799	0.13576	7.261322	3.83E-13	1.44E-11
ARPC5L	168.7644683	0.986962	0.11118	8.876777	6.88E-19	3.94E-17
WDR18	16.89304945	0.98782	0.33822	2.92062	0.003493352	0.024224798
TCEAL8	32.67929716	0.989644	0.24439	4.049435	5.13E-05	0.000633627
SMPD1	17.51195189	0.990911	0.33532	2.955117	0.003125506	0.022176999

SKA2	46.29444907	0.994505	0.2087	4.765206	1.89E-06	3.09E-05
TRG-AS1	73.08047857	0.995465	0.17249	5.771193	7.87E-09	1.81E-07
HLA-DRB1	640.7497229	0.99891	0.1171	8.530485	1.46E-17	7.43E-16
C6orf136	16.46119555	0.999332	0.34985	2.85649	0.004283531	0.028628219
ZNF683	37.81909145	1.003467	0.24038	4.174494	2.99E-05	0.000387185
DOK2	247.9613051	1.011641	0.09792	10.33118	5.09E-25	3.86E-23
BAK1	28.31639634	1.014532	0.26293	3.858552	0.000114061	0.00129326
C11orf21	30.60859402	1.015922	0.25373	4.003875	6.23E-05	0.000755956
PRR5L	27.38792939	1.017432	0.27012	3.766558	0.000165514	0.001797967
LACTB2	15.57019306	1.019659	0.35872	2.842497	0.004476167	0.029601353
CENPK	46.17826516	1.022816	0.2077	4.924447	8.46E-07	1.46E-05
GPNMB	14.59488504	1.028013	0.37549	2.737802	0.006185137	0.038263734
TOX2	14.35249197	1.033672	0.38326	2.697067	0.006995322	0.041956247
ISOC2	15.98141324	1.037315	0.35278	2.940403	0.003277859	0.023044079
SLC15A4	27.13674735	1.038027	0.27483	3.776975	0.000158745	0.001736502
EPS8L2	24.14410103	1.038449	0.29263	3.548625	0.000387248	0.003706224
SURF6	17.07058954	1.038994	0.35828	2.899985	0.00373181	0.025563613
FGFBP2	172.0526945	1.039959	0.11971	8.687366	3.71E-18	2.00E-16
CORO1B	81.79601817	1.041382	0.16039	6.493011	8.41E-11	2.46E-09
FKBPL	12.96823027	1.047004	0.39329	2.662138	0.007764597	0.045598896
NT5C2	16.88577412	1.049459	0.35248	2.977322	0.002907781	0.020811546
GBP4	34.8493005	1.051437	0.25159	4.179203	2.93E-05	0.000381719
HSCB	21.72596132	1.053058	0.31744	3.317305	0.000908904	0.007669216
CD27	120.4619203	1.053951	0.13617	7.740029	9.94E-15	4.41E-13
SNAPC2	20.92134206	1.055394	0.31163	3.386653	0.000707507	0.006189963
OGG1	19.27298533	1.05578	0.32525	3.246073	0.001170088	0.009528941
FBXO6	25.24664061	1.057451	0.28221	3.747075	0.000178909	0.001918159
MRPL35	25.83456395	1.057549	0.28086	3.765455	0.000166246	0.001804089
CTSC	287.1779076	1.061455	0.08948	11.86237	1.86E-32	1.93E-30
CPE	18.64923073	1.061688	0.33052	3.212213	0.001317169	0.010533413
SNX18	14.53790884	1.063588	0.38928	2.732204	0.00629122	0.038727288
COA3	34.70242561	1.067609	0.24646	4.331707	1.48E-05	0.000203228
ARL3	27.02377362	1.075602	0.27204	3.953812	7.69E-05	0.000912416
NDFIP2	16.90685116	1.076345	0.36102	2.981412	0.002869224	0.020563093
PHF23	27.49552186	1.077693	0.27104	3.976115	7.01E-05	0.00083841
ZNRF1	12.34489128	1.084856	0.40066	2.707697	0.006775178	0.040957288
C1R	35.34797715	1.084913	0.2407	4.507386	6.56E-06	9.73E-05
BCL2L1	29.18931553	1.087791	0.25943	4.193064	2.75E-05	0.000360878
EFEMP1	20.74171976	1.088607	0.31823	3.420822	0.000624322	0.005562234
ZFYVE21	15.21770262	1.08953	0.35957	3.030104	0.002444693	0.017978152
HLA-DMA	70.04869947	1.093404	0.17817	6.136707	8.42E-10	2.19E-08
FCGR3A	67.20368083	1.105359	0.18507	5.972774	2.33E-09	5.65E-08
ADH1B	34.88040035	1.119827	0.24577	4.556389	5.20E-06	7.89E-05
PTGDS	67.07842544	1.119872	0.32627	3.432386	0.000598296	0.005361615
C7	32.25862052	1.129116	0.25841	4.369403	1.25E-05	0.000175636
LAT2	13.96296204	1.131252	0.38637	2.927926	0.003412315	0.023828082
SEMA3C	13.45273293	1.134605	0.40514	2.800539	0.005101735	0.032825353
COL1A1	18.1899499	1.147998	0.34952	3.284544	0.001021474	0.008485846
MFAP4	28.96814136	1.151831	0.27053	4.257739	2.07E-05	0.000276546
MOSPD2	16.41256543	1.154658	0.36909	3.128418	0.001757499	0.013548444
CETN3	13.68206462	1.160144	0.3966	2.925241	0.0034419	0.023945599
HAVCR2	12.26471193	1.167996	0.41005	2.848436	0.004393474	0.029217013

CAMK1	10.06282982	1.185715	0.45104	2.628874	0.008566808	0.049222361
ACKR3	11.64948656	1.185869	0.41839	2.834364	0.004591699	0.030245057
RFC2	15.3217163	1.186822	0.36856	3.220152	0.001281225	0.010292126
LGALS9	13.25034404	1.190898	0.40282	2.956423	0.003112295	0.022097911
LAMB1	11.70893561	1.193479	0.43998	2.712587	0.006676031	0.040477289
VCAM1	17.31249886	1.19602	0.34355	3.481326	0.000498938	0.00459831
ARL4D	22.21521962	1.204093	0.31476	3.825388	0.000130566	0.001455269
CCDC141	12.18816066	1.211926	0.4343	2.790501	0.00526265	0.033573163
KIFC1	17.33727109	1.213722	0.3446	3.522168	0.000428033	0.004045898
CX3CR1	36.49851072	1.214639	0.25027	4.853317	1.21E-06	2.04E-05
APP	12.15569407	1.217975	0.40725	2.990696	0.002783423	0.020042147
SIT1	111.1495528	1.221476	0.14344	8.515513	1.66E-17	8.37E-16
CLIC3	108.0759091	1.222794	0.22559	5.42044	5.95E-08	1.21E-06
MCM3	16.55047749	1.227747	0.37166	3.303447	0.00095504	0.008008568
HMOX1	10.47205261	1.2356	0.44237	2.79314	0.005219903	0.033370188
LAMA4	16.35063215	1.238332	0.37633	3.290565	0.000999865	0.008332211
HMG20A	18.18234491	1.245556	0.33906	3.673533	0.00023922	0.002444751
FGR	16.78654676	1.246791	0.36172	3.446847	0.000567169	0.005112644
KLRF1	55.64932486	1.249201	0.33669	3.710191	0.000207103	0.002164024
GPR18	13.28404421	1.252267	0.39679	3.156029	0.001599327	0.01251851
RGS3	19.32238169	1.254525	0.33537	3.740729	0.000183488	0.001959399
TFPT	27.37990507	1.265284	0.28102	4.502431	6.72E-06	9.93E-05
PTPN6	57.41857641	1.26619	0.19266	6.572122	4.96E-11	1.50E-09
LAIR2	25.32414869	1.270645	0.30436	4.174858	2.98E-05	0.000387185
PLAC9	29.68022435	1.274981	0.26657	4.782878	1.73E-06	2.84E-05
EIF2B2	17.45418536	1.280199	0.34345	3.727418	0.000193451	0.002047408
B3GALT4	16.22917784	1.306239	0.36363	3.592242	0.000327845	0.003209459
RPP25L	16.3317729	1.313284	0.35552	3.693994	0.000220759	0.002282244
FMO2	10.68780471	1.314897	0.47625	2.760924	0.005763809	0.036150502
SFRP2	14.56062686	1.325809	0.38059	3.483583	0.000494749	0.004567573
CCDC28B	16.1882504	1.332584	0.36362	3.664743	0.000247588	0.002515846
CXCL12	31.40607903	1.333305	0.25809	5.165986	2.39E-07	4.46E-06
C3	64.47086183	1.342947	0.18774	7.153119	8.48E-13	3.04E-11
SLPI	19.48209139	1.345508	0.33095	4.065627	4.79E-05	0.000594627
WISP2	9.741750539	1.346157	0.47426	2.838442	0.004533437	0.029943073
PDGFRA	9.656057951	1.354006	0.47535	2.848464	0.004393078	0.029217013
TMED1	13.26571881	1.354721	0.39953	3.390763	0.000696982	0.006112877
FASLG	25.05889215	1.354836	0.29469	4.597473	4.28E-06	6.61E-05
AC004865.2	25.35671994	1.361784	0.29149	4.671881	2.98E-06	4.77E-05
SPSB2	17.34040375	1.362426	0.34887	3.905248	9.41E-05	0.001092384
IRF8	12.44098077	1.36294	0.43655	3.122107	0.001795618	0.013812448
NUPR1	14.13313025	1.374785	0.38664	3.555707	0.000376963	0.003623996
SASH3	52.5919465	1.381624	0.20402	6.772022	1.27E-11	4.06E-10
ZCCHC24	7.740208804	1.39029	0.52753	2.635485	0.008401717	0.04851504
LRP1	13.3404446	1.392908	0.39706	3.508065	0.000451379	0.004229209
ACADS	13.15538296	1.402885	0.42036	3.337376	0.000845733	0.00721588
CD79B	23.89117055	1.405213	0.30101	4.66825	3.04E-06	4.84E-05
GEMIN7	12.37505173	1.409131	0.41721	3.377511	0.00073145	0.006373383
AKR1C3	13.39237489	1.41179	0.4234	3.33445	0.000854684	0.00727535
SOX4	9.500389475	1.425117	0.49255	2.89337	0.003811324	0.025958729
SRPX	9.411262516	1.432005	0.48057	2.979793	0.002884433	0.020658258
GGCT	22.87090514	1.436934	0.31229	4.601291	4.20E-06	6.51E-05

DAB2	10.40927743	1.455523	0.45606	3.191522	0.001415253	0.011247574
DNAL4	8.081729975	1.456553	0.52474	2.775766	0.005507176	0.034826702
PLEK	108.4547956	1.459629	0.14642	9.9688	2.09E-23	1.49E-21
RTP4	9.597031136	1.466603	0.47712	3.073845	0.002113189	0.015923325
ALDH3A2	12.28506951	1.466902	0.42004	3.492293	0.000478892	0.004440337
AIF1	17.73669954	1.471335	0.35669	4.124962	3.71E-05	0.000470085
ABHD11	8.638937655	1.48205	0.51066	2.902214	0.003705352	0.025414913
PAFAH2	11.34005449	1.486327	0.43995	3.378383	0.000729136	0.006358396
GEM	7.413733477	1.487131	0.55723	2.668781	0.007612718	0.044829982
CISH	37.07780791	1.509419	0.24495	6.162076	7.18E-10	1.88E-08
FSTL1	36.24036198	1.539877	0.25188	6.113503	9.75E-10	2.48E-08
MFAP5	27.17657557	1.582141	0.29088	5.439087	5.36E-08	1.10E-06
PRG4	12.72762739	1.588464	0.42609	3.728026	0.000192986	0.002044503
MARCKS	19.07146142	1.589511	0.35805	4.439377	9.02E-06	0.000130984
COL3A1	19.27875969	1.599726	0.34668	4.614443	3.94E-06	6.13E-05
TMEM204	23.06453768	1.601715	0.31986	5.007616	5.51E-07	9.71E-06
FBLN1	40.25395921	1.606342	0.23926	6.713896	1.89E-11	6.00E-10
TNXB	27.40751331	1.609325	0.29876	5.386639	7.18E-08	1.43E-06
LAMB2	6.932291843	1.614332	0.56419	2.861315	0.004218873	0.028284425
SLC2A8	9.762854913	1.627329	0.47804	3.404202	0.000663576	0.005848652
NFIB	9.159544115	1.633547	0.50991	3.203588	0.001357267	0.010821722
TRIB3	8.166329308	1.636646	0.52956	3.090585	0.001997627	0.01514855
MYOM2	43.84789567	1.6487	0.2343	7.036637	1.97E-12	6.75E-11
TRIO	7.210426494	1.651102	0.5871	2.812279	0.004919182	0.031919496
MINPP1	5.937106247	1.65207	0.61475	2.687406	0.007200942	0.042935317
C1S	34.50610774	1.656029	0.26262	6.305884	2.87E-10	7.86E-09
PLXDC2	6.586769671	1.663977	0.59887	2.778535	0.005460456	0.034592587
ZNF595	8.31828906	1.688919	0.52425	3.221588	0.001274823	0.010256093
STYXL1	8.335703259	1.694372	0.52198	3.246048	0.00117019	0.009528941
AP003774.4	15.13438864	1.719009	0.39347	4.368877	1.25E-05	0.000175827
ANKRD35	9.675995835	1.72402	0.4882	3.531385	0.00041339	0.003924823
EBF1	6.21466239	1.742106	0.62168	2.802263	0.005074548	0.032689742
AKAP12	6.630922966	1.744518	0.5924	2.944812	0.003231506	0.02276308
THBS2	5.495861709	1.744585	0.66039	2.641759	0.008247663	0.047780181
PMP22	14.61536338	1.791208	0.39858	4.493959	6.99E-06	0.000103179
SDC2	5.788799059	1.796197	0.64054	2.804175	0.005044549	0.03253567
COL12A1	5.864805946	1.799781	0.6528	2.757026	0.005832978	0.036477417
RAB33A	12.81871424	1.818841	0.4364	4.167821	3.08E-05	0.000397224
FCER1G	36.12767867	1.835225	0.47116	3.89516	9.81E-05	0.001133943
COL1A2	39.09640895	1.83959	0.24696	7.449043	9.40E-14	3.81E-12
CPNE2	12.89965387	1.840408	0.42845	4.295483	1.74E-05	0.000236095
SLC25A29	18.30982696	1.869861	0.3643	5.132783	2.85E-07	5.25E-06
CD200R1	12.48689472	1.872005	0.45276	4.13469	3.55E-05	0.000451971
ECM2	6.576727042	1.882403	0.62457	3.013901	0.002579116	0.018824379
SVEP1	19.70219333	1.888665	0.36193	5.218293	1.81E-07	3.41E-06
LINC02384	11.22038104	1.890026	0.46829	4.036024	5.44E-05	0.000667088
GFRA1	4.654857306	1.915229	0.72064	2.657691	0.007867808	0.046053359
ASB2	8.829230616	1.918076	0.53086	3.613117	0.000302538	0.003002249
ASCL2	9.422628847	1.934754	0.52081	3.714864	0.000203313	0.002132791
PI16	8.230139251	1.940741	0.55056	3.525019	0.000423453	0.004009685
MEG3	16.61778111	1.944403	0.41141	4.726138	2.29E-06	3.69E-05
CEP41	5.676239131	1.964367	0.66357	2.96032	0.003073198	0.021863839

CPNE7	6.260342956	1.968731	0.63135	3.118278	0.001819114	0.013983131
IGHG4	13.32676574	1.972592	0.43129	4.573746	4.79E-06	7.30E-05
COX7A1	7.008713891	1.985591	0.60515	3.281143	0.001033875	0.008568907
ABCA1	4.869815948	1.986541	0.71929	2.761795	0.005748462	0.036075391
HP	25.04647007	2.009726	0.31754	6.328973	2.47E-10	6.84E-09
LAMC1	11.60181244	2.019594	0.46988	4.298117	1.72E-05	0.0002339
CXXC5	8.731703908	2.034075	0.54475	3.733942	0.000188506	0.002006976
LAMA2	9.675978377	2.060579	0.52501	3.924868	8.68E-05	0.001019227
ABCA9	14.91234013	2.068238	0.42467	4.870238	1.11E-06	1.88E-05
DLC1	4.433736376	2.068778	0.75654	2.734515	0.006247228	0.038527576
TCF4	8.778818872	2.096676	0.55445	3.781509	0.00015588	0.001708934
CPB1	12.25383901	2.120066	0.48572	4.364826	1.27E-05	0.00017818
FBLN2	12.45381887	2.13803	0.4673	4.575306	4.76E-06	7.26E-05
AL512625.3	13.27979327	2.149358	0.45301	4.744614	2.09E-06	3.39E-05
FBN1	32.23822174	2.151927	0.28662	7.508045	6.00E-14	2.47E-12
ABCA8	20.50446773	2.174451	0.36708	5.923655	3.15E-09	7.55E-08
JCHAIN	10.29212655	2.192807	0.50925	4.305966	1.66E-05	0.00022662
F10	3.946872181	2.203006	0.82292	2.67705	0.007427349	0.043935392
DST	11.97644882	2.214868	0.47789	4.634652	3.58E-06	5.63E-05
MMP2	17.86686038	2.215336	0.39219	5.648642	1.62E-08	3.60E-07
FN1	7.145794934	2.216234	0.613	3.615376	0.000299912	0.002985723
AC004585.1	6.607613418	2.269078	0.63862	3.553098	0.000380723	0.003653574
IGFBP5	46.47960712	2.303935	0.24526	9.393679	5.79E-21	3.76E-19
ABCA6	9.352562121	2.315526	0.55022	4.208367	2.57E-05	0.00033937
CD248	6.795998199	2.344283	0.64823	3.616445	0.000298676	0.00298119
TK1	3.729238878	2.440712	0.90547	2.695531	0.00702765	0.042055845
VIT	3.773752978	2.456818	0.91064	2.697902	0.0069778	0.041910992
FLRT2	5.58555138	2.493736	0.73588	3.388781	0.000702042	0.006147174
LIFR	5.661069294	2.504941	0.72132	3.472715	0.000515222	0.004719927
ABCA10	10.24831017	2.515497	0.54118	4.648169	3.35E-06	5.30E-05
DCLK1	3.995253961	2.541294	0.87906	2.890924	0.003841104	0.026111698
COL6A3	16.82087774	2.575659	0.42881	6.00659	1.89E-09	4.63E-08
IGHG1	17.84333515	2.579454	0.42567	6.059733	1.36E-09	3.42E-08
S100B	39.64170712	2.692632	0.42101	6.39561	1.60E-10	4.57E-09
ADAMTS5	7.527441747	2.708208	0.65536	4.132414	3.59E-05	0.000455635
HSPG2	6.397462955	2.709699	0.70847	3.824699	0.000130932	0.001457824
PLAU	4.396200471	2.727475	0.86908	3.138348	0.00169903	0.013173638
CD38	10.80532176	2.737853	0.56243	4.867865	1.13E-06	1.90E-05
ZNF48	4.535895378	2.771179	0.86919	3.188231	0.001431462	0.011354073
IGHG3	14.5281236	2.777531	0.48361	5.743276	9.29E-09	2.13E-07
KRT86	6.785463187	2.790729	0.74586	3.741645	0.00018282	0.001954218
CATSPER1	3.409504087	2.84249	1.0055	2.826947	0.004699409	0.030811075
IGKC	140.2993544	2.860603	0.22361	12.79298	1.79E-37	2.26E-35
IGHG2	6.071465335	2.918766	0.76545	3.813137	0.000137214	0.001518295
CLDN11	6.225277942	2.994082	0.77992	3.838952	0.000123561	0.001388045
IGHM	9.5804919	3.049184	0.87394	3.488996	0.000484839	0.004491584
ZNF93	4.0088581	3.062028	0.97028	3.155808	0.001600541	0.012518848
CLNK	6.675927341	3.389869	0.82414	4.113203	3.90E-05	0.000491201
CCL23	5.261752771	3.526344	1.30515	2.70187	0.00689508	0.041541302
AC006504.5	3.634190677	3.560266	1.13678	3.1319	0.001736791	0.01340813
IGLC2	48.2751019	3.993306	0.35626	11.20887	3.69E-29	3.43E-27
IGHA1	22.15205505	4.031514	0.5291	7.619638	2.54E-14	1.10E-12

LRRN3	3.773282106	4.390402	1.14407	3.837514	0.000124286	0.001392528
IGLC3	12.69182224	4.40601	0.77894	5.65645	1.55E-08	3.44E-07
CXCL13	25.14327587	4.488802	1.10405	4.065765	4.79E-05	0.000594627
PCLAF	6.03838638	5.057168	1.13511	4.455236	8.38E-06	0.000122329
AC004556.1	6.416700907	5.930508	1.10462	5.368806	7.93E-08	1.57E-06

Supplementary Table S4: Primers used for quantitative real-time PCR.

Actin F	5'-GGTGGGAATGGGTCAGAAG-3'
Actin R	5'-AGCTCATTGTAGAAGGTGTGG-3'
Ccl2 F	5'-TAAAAACCTGGATCGGAACCAAA-3'
Ccl2 R	5'-GCATTAGCTTCAGATTTACGGGT-3'
Cxcl2 F	5'-CAGAAGTCATAGCCACTCTCAAG-3'
Cxcl2 R	5'-CTCCTTTCCAGGTCAGTTAGC-3'
Tnfaip6 F	5'-TCTCGCAACCTACAAGCAG-3'
Tnfaip6 R	5'-CCGTTTTCCCAAATCCACAG-3'
Ifng F	5'-ATGAACGCTACACACTGCATC-3'
Ifng R	5'-CCATCCTTTTGCCAGTTCCTC-3'
Tnf F	5'-CCCTCACACTCAGATCATCTTCT-3'
Tnf R	5'-GCTACGACGTGGGCTACAG-3'
Il6 F	5'-TAGTCCTTCCTACCCCAATTTCC-3'
Il6 R	5'-TTGGTCCTTAGCCACTCCTTC-3'
Plin1 F	5'-GGGACCTGTGAGTGCTTCC-3'
Plin1 R	5'-GTATTGAAGAGCCGGGATCTTTT-3'
Fasn F	5'-GGAGGTGGTGATAGCCGGTAT-3'
Fasn R	5'-TGGGTAATCCATAGAGCCCAG-3'
Atgl F	5'-GGATGGCGGCATTTCAGACA-3'
Atgl R	5'-CAAAGGGTTGGGTTGGTTCAG-3'
Hsl F	5'-CCAGCCTGAGGGCTTACTG-3'
Hsl R	5'-CTCCATTGACTGTGACATCTCG-3'
Mgl F	5'-CGGACTTCCAAGTTTTTGTCAGA-3'
Mgl R	5'-GCAGCCACTAGGATGGAGATG-3'
Bax F	5'-TGAAGACAGGGGCCTTTTTG-3'
Bax R	5'-AATTCGCCGGAGACACTCG-3'
Bcl2l1 F	5'-GACAAGGAGATGCAGGTATTGG-3'
Bcl2l1 R	5'-TCCCGTAGAGATCCACAAAAGT-3'
Casp3 F	5'-ATGGAGAACAACAAAACCTCAGT-3'
Casp3 R	5'-TTGCTCCCATGTATGGTCTTTAC-3'
Casp6 F	5'-GGAAGTGTTCGATCCAGCCG-3'
Casp6 R	5'-GGAGGGTCAGGTGCCAAAAG-3'
Capn1 F	5'-ATGACAGAGGAGTTAATCACCCC-3'
Capn1 R	5'-GGCTATGAGAAACCGGAGGG-3'

Supplementary Table S5: Macrophage polarization and T cell signature gene list

M1_Polarization	M2_Polarization	Cytolytics effector pathway	TCA cycle	Glycolysis	CD4/CD8 resting
IL12	ARG1	EOMES	ACLY	ALDOA	WDR86
IL23	ARG2	TBX21	ACO1	ALDOB	IL7R
TNF	IL10	GZMB	ACO2	ALDOC	TSPAN2
IL6	CD32	PRF1	CS	BPGM	LRRC2
CD86	CD163	FASL	DLAT	ENO1	SNAI3
IL1B	CD23	GZMH	DLD	ENO2	CD52
MARCO	CD200R1	GZMA	DLST	GALM	TIMP1
NOS2	PDCD1LG2		FH	GCK	YPEL4
CD64	CD274		IDH1	GPI	CALHM2
CD80	MARCO		IDH2	HK2	S100A4
CXCR10	CSF1R		IDH3A	HK3	TSC22D3
CXCL9	CD206		IDH3B	PFKL	EPHA4
CXCL10	IL1RN		IDH3G	PGAM2	ZNF831
CXCL11	IL1R2		MDH1	PGK1	ZCCHC18
IL1A	IL4R		MDH1B	PGK2	GLIPR1
CCL5	CCL4		MDH2	PGM1	AIM1L
IRF5	CCL13		OGDH	PGM2	KLRB1
IRF1	CCL20		PC	PGM3	CNPY4
CD40	CCL17		PCK1PCK2	PKLR	GDPD5
IDO1	CCL18		PDHA1	TPI1	TC2N
KYNU	CCL22		PDHB		AHNAK
CCR7	CCL24		SDHA		PBXIP1
	LYVE1		SDHB		HIST1H2BD
	VEGFA		SDHD		BCL9L
	VEGFB		SUCLA2		EEPD1
	VEGFC		SUCLG1		ADAM23
	VEGFD		SUCLG2		THRA
	EGF				KCTD7
	CTSA				CTSF
	CTSB				CAMK2N1
	CSTC				DCP1B
	CTSD				SEPT4
	TGFB1				FXYD2
	TGFB2				CECR1
	TGFB3				DPYSL2
	MMP14				CCDC65
	MMP19				CDC25B
	MMP9				FAM229A
	CLEC7A				RP11-111M22.2
	WNT7B				BCO2
	FASL				HHAT
	TNFSF12				TGFB3
	TNFSF8				ANTXR2
	CD276				AQP3
	VTCN1				CRIP2
	MSR1				MYO1F
	FN1				MPP7

	IRF4				NMT2<br>UTRN<br>NLRP3<br>BTD<br>KLF2<br>ZFP36L2<br>SUN2<br>FXYD1<br>BEST4<br>IGFBP6<br>SFXN3<br>CEP128<br>PLCD1<br>CYB561<br>ANKMY2<br>NBPF11<br>BAZ2B<br>IL11RA<br>ITGB7<br>ACSF2<br>ATXN7L1<br>PINK1<br>PDCD4
